# Practical synthesis of allylic amines *via* nickel-catalysed multicomponent coupling of alkenes, aldehydes, and amides†

**DOI:** 10.1039/d3sc03233g

**Published:** 2023-07-25

**Authors:** Wei-Guo Xiao, Bin Xuan, Li-Jun Xiao, Qi-Lin Zhou

**Affiliations:** a State Key Laboratory, Institute of Elemento-Organic Chemistry, College of Chemistry, Frontiers Science Center for New Organic Matter, Nankai University Tianjin 300071 China ljxiao@nankai.edu.cn

## Abstract

Molecules with an allylic amine motif provide access to important building blocks and versatile applications of biologically relevant chemical space. The need for diverse allylic amines requires the development of increasingly general and modular multicomponent reactions for allylic amine synthesis. Herein, we report an efficient catalytic multicomponent coupling reaction of simple alkenes, aldehydes, and amides by combining nickel catalysis and Lewis acid catalysis, thus providing a practical, environmentally friendly, and modular protocol to build architecturally complex and functionally diverse allylic amines in a single step. The method is remarkably simple, shows broad functional-group tolerance, and facilitates the synthesis of drug-like allylic amines that are not readily accessible by other methods. The utilization of accessible starting materials and inexpensive Ni(ii) salt as the alternative precatalyst offers a significant practical advantage. In addition, the practicality of the process was also demonstrated in an efficient, gram-scale preparation of the prostaglandin agonist.

## Abstract 2

A practical and efficient protocol is reported for synthesizing valuable allylic amines *via* a catalytic multicomponent coupling reaction of alkenes, aldehydes, and amides using nickel and Lewis acid catalysts.

## Graphical abstract

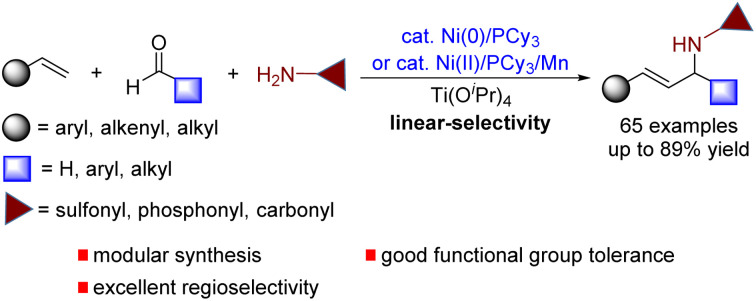

## Introduction

**Scheme 1:**
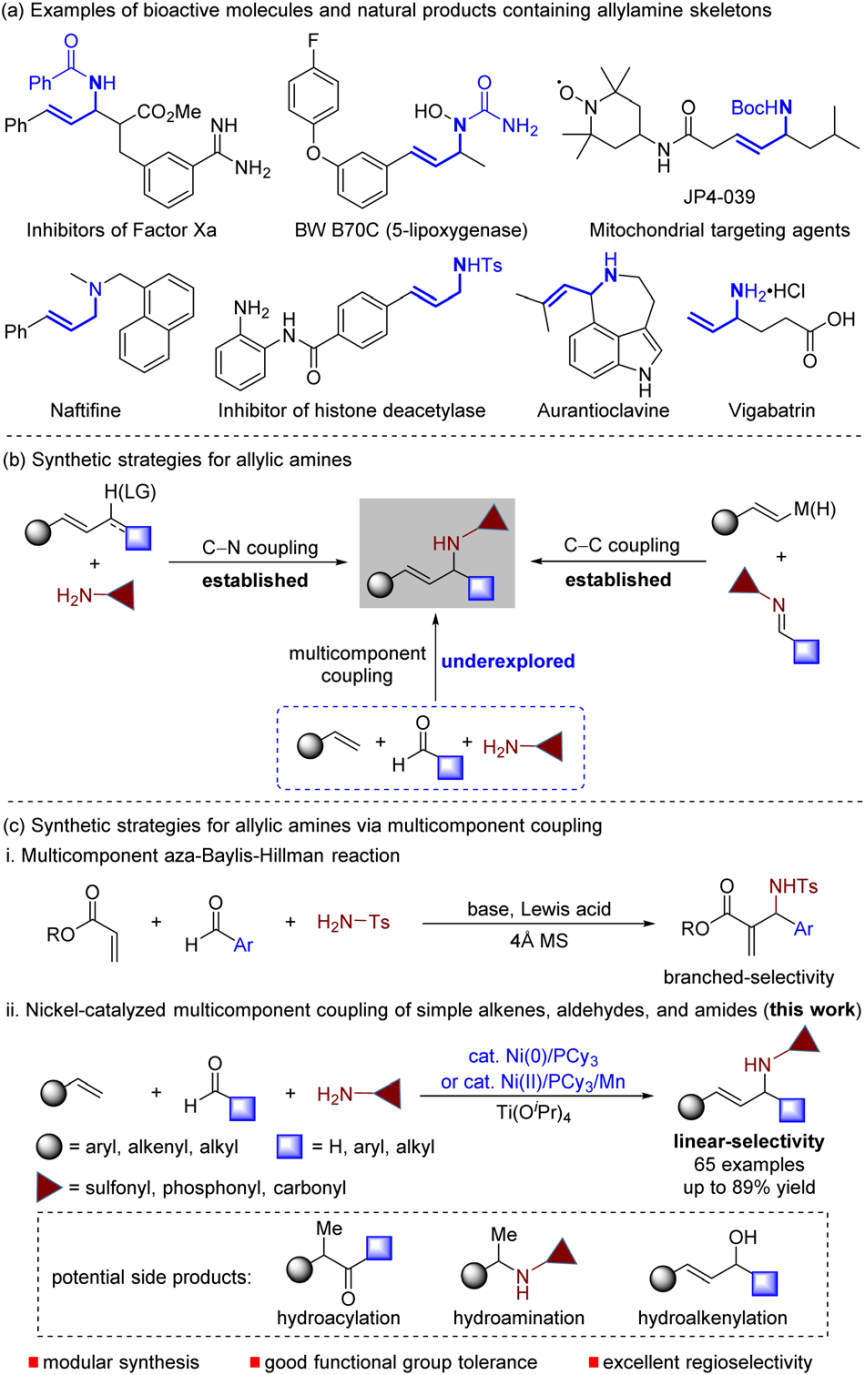
Pharmaceuticals displaying allylic amine motifs and the synthetic methods of allylic amines. LG = leaving group.

Allylic amines are not only important building blocks used in the synthesis of heterocycles and bioactive amines,^1^ but are also crucial structural motifs in biologically relevant agents, drugs, and natural products (Scheme 1a).^2^ Traditionally, two methods are used to synthesize allylic amines by forming C–N or C–C bonds between coupling partners (Scheme 1b). The first method involves the amination of allylating reagents,^3^ while the other is achieved by adding alkenylmetals to imines.^4^ Both methods are limited by the need for preparing allylating reagents or using stoichiometric organometallic reagents. To circumvent these limitations, new catalytic approaches, such as allylic C–H amination,^5^ hydroamination of 1,3-dienes,^6^ reductive coupling of alkynes with imines,^7^ and direct coupling of alkenes with imines,^8^ have recently been developed. Although these catalytic methods have significantly improved allylic amine synthesis efficiency, they still have limitations in expanding the diversity of allylic amines through two-component coupling. Therefore, there is a need to develop increasingly general and modular multicomponent reactions for allylic amine synthesis *via* sequential C–C and C–N bond formation, which would increase the diversity of allylic amines (Scheme 1b).

A multicomponent reaction is a powerful tool that generates molecular diversity with chemical and biological applications.^9^ Multicomponent coupling reactions use a minimum of three independent variable reactants in a single step. Among the various multicomponent coupling reactions, the aza-Baylis–Hillman reaction combines activated alkenes, aldehydes, and amides to produce branched allylic amines (Scheme 1c).^8*a*,*b*,10^ Despite its numerous synthetic applications, this method is limited by its substrate scope. Specifically, the alkene coupling partner is primarily restricted to α,β-unsaturated esters. Thus, discovering a general and practical multicomponent coupling method that allows streamlined access to linear allylic amines through joining three abundant chemical feedstocks – simple alkene, aldehyde, and amide – is still highly desirable.^11^

Nickel(0)-catalysed multicomponent coupling reaction of unsaturated hydrocarbons with either carbonyls or imines has become a powerful protocol for C–C bond formation.^12^ Recently, we have developed nickel-catalysed coupling reactions of alkenes with aldehydes and imines for the synthesis of ketones^13^ and allylic amines,^8*f*^ respectively. We wondered whether we could control the chemoselectivity of multicomponent coupling between alkenes, aldehydes, and amides for the synthesis of allylic amines, which can suppress interference from the nickel-catalysed hydrofunctionalization of alkenes^13,14^ or hydroalkenylation of aldehydes^15^ (Scheme 1c). If successful, this strategy would open up new opportunities in multicomponent reactions and avoid the challenges associated with the utilization of preformed imines in allylic amine synthesis. Imines, particularly aliphatic ones, are unstable and reactive, undergoing imine–enamine tautomerization, decomposition, and self-condensation during preparation and storage, limiting their usefulness.^16^ Herein, we report the first catalytic multicomponent coupling reaction of simple alkenes, common aldehydes, and amides by combining nickel catalysis and Lewis acid catalysis, providing a modular protocol for the synthesis of architecturally complex and functionally diverse allylic amines in a single step (Scheme 1c). Our method uses *in situ*-generated imines from a wide range of aldehydes and amides, significantly increasing the reaction's practicality by avoiding the need for inaccessible imines. It is remarkably simple, shows broad functional-group tolerance, and facilitates the synthesis of drug-like amines with the functionalities present in pharmaceuticals, which are challenging to access with other C–C or C–N bond-forming reactions.

## Results and discussion

We initiated our investigation by examining nickel catalysis and Lewis catalysis in the multicomponent coupling reaction, as shown in Table 1. After conducting significant optimizations (for detail, see the ESI†), we discovered that a combination of Ni(COD)_2_, PCy_3_, and Ti(O^i^Pr)_4_ was capable of promoting the target reaction at 100 °C and generating product 1 in an isolated yield of 89% (entry 1). Control experiments conducted without either metal, ligand, or Ti(O^i^Pr)_4_ confirmed that these components play a crucial role in the multicomponent coupling reaction (entries 2–4). While several Lewis acids, including Sc(OTf)_3_, Yb(OTf)_3_, and Mg(OTf)_2_, are commonly used as catalysts in imine formation,^10^ only Ti(O^i^Pr)_4_ (ref. 10b) has been proven to be compatible and effective in this case, highlighting the subtleties of our catalytic protocol (entries 5–7). The use of Ni(ii) precursors other than Ni(COD)_2_ resulted in only trace amounts of the desired products (entry 8). However, when Zn and Mn were used as reducing agents, the reaction proceeded with 66% (entry 9) and 83% (entry 10) yields, respectively. We identified bulky and electron-rich monodentate PCy_3_ to be critical for the success of this reaction. Under our reaction conditions, no other ligands, such as the bisphosphine ligand dcype (1,2-bis(dicyclohexylphosphino)ethane, entry 11) or the N-heterocyclic carbene ligand IPr (1,3-bis(2,6-diisopropylphenyl)imidazol-2-ylidene, entry 12), produced the desired coupling products (see Table S2 in the ESI† for ligand optimization). The reaction could also be conducted using less polar solvents than MeCN, such as THF and toluene, but this resulted in decreased yields (entries 13 and 14).

**Table 1:** Optimization of the reaction conditions and variation from standard conditions^a^

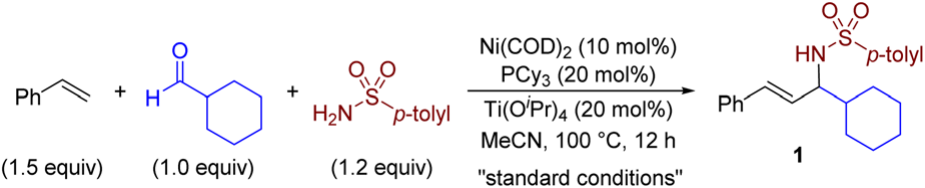		
Entry	Variation from standard conditions	Yield of 1^b^ (%)
**1**	**None**	**94 (89)**
2	Without Ni(COD)_2_	0
3	Without PCy_3_	0
4	Without Ti(O^i^Pr)_4_	50
5	Sc(OTf)_3_ instead of Ti(O^i^Pr)_4_	0
6	Yb(OTf)_3_ instead of Ti(O^i^Pr)_4_	0
7	Mg(OTf)_2_ instead of Ti(O^i^Pr)_4_	0
8	Ni(OAc)_2_ instead of Ni(COD)_2_	Trace
9^c^	Ni(OAc)_2_/Zn instead of Ni(COD)_2_	66
**10** d	**Ni(OAc)**_**2**_ **/Mn instead of Ni(COD)**_**2**_	**83**
11	dcype instead of PCy_3_	33
12	IPr instead of PCy_3_	0
13	THF instead of MeCN	75
14	Toluene instead of MeCN	46

a Reaction conditions: Ni(COD)_2_ (10 mol%), PCy_3_ (20 mol%), alkene (0.15 mmol), aldehyde (0.1 mmol), amide (0.12 mmol), Ti(O^i^Pr)_4_ (0.2 equiv.), MeCN (0.5 mL).

b Yields were determined by ^1^H NMR analysis using dibromomethane as an internal standard. The isolated yield was given in parentheses.

c Ni(OAc)_2_ (10 mol%), Zn powder (20 mol%).

d Ni(OAc)_2_ (10 mol%), Mn powder (20 mol%).

After establishing optimal reaction conditions with Ni(0) (Table 1, entry 1) and Ni(ii) precatalyst (Table 1, entry 10), we aimed to determine the substrate scope of the nickel-catalysed multicomponent coupling process (Table 2). Our initial focus was on assessing the appropriateness of all types of amides. As shown in Table 2a, a wide variety of amides including aromatic, heteroaromatic, aliphatic sulfonamides, and *N*,*N*-dimethyl sulfonamide, could be employed as substrates with complete linear selectivity to produce the targeted products (yielding 1–9). We also assessed the feasibility of utilizing phosphoramides (yielding 10 and 11) and *tert*-butyl carbamate (yielding 12) as reaction substrates and achieved remarkably respectable yields ranging from 41% to 58% in generating the desired allylic amines. Notably, the reaction could be extended to a series of carboxamides with good yields (yielding 13–16). Subsequently, a diverse range of aldehydes, including those bearing heteroarenes, efficiently gave allylic amines 17–24 in good yields (Table 2b). The reaction also produced the expected allylic amines 25–35 in good yields for aliphatic aldehydes, including those with saturated heterocycles or strained ring features typical of pharmaceutical agents. Notably, in the presence of hemiacetals, the reaction provided amino alcohol 33 in comparable yield with the same sense of regioselectivity. Interestingly, bulky aldehydes, such as pivaldehyde, and even the simplest aldehyde, formaldehyde,^17^ were effective coupling partners, giving the desired allylic amines 34 and 36 in 53% and 70% yields, respectively. Moreover, we observed that the nickel-catalysed multicomponent coupling process was effective with a wide range of alkenes tested using piperidine-4-carbaldehyde as the coupling partner (Table 2c). Styrene derivatives, including those bearing heteroarenes, showed efficient conversion, yielding products 37–44 in moderate to good yields. The reaction also accommodated challenging 1,3-diene (yielding 45) and nitrogen-containing alkene substrates (yielding 46), which are prone to homocoupling in metal-catalysed reactions. Aliphatic alkenes with unbiased electrophilicity and low activity were also viable coupling partners, with complete linear regioselectivity observed to form an array of homoallylic and allylic amine mixtures (yielding 47–52).^8*f*^ In addition, vinylcyclohexane and allylbenzene derivatives proved to be viable substrates, enabling facile access to homoallylic amines with excellent selectivity (yielding 50–52).

**Table 2:** Substrate scope of nickel-catalysed multicomponent coupling of simple alkenes, aldehydes, and amides^a^

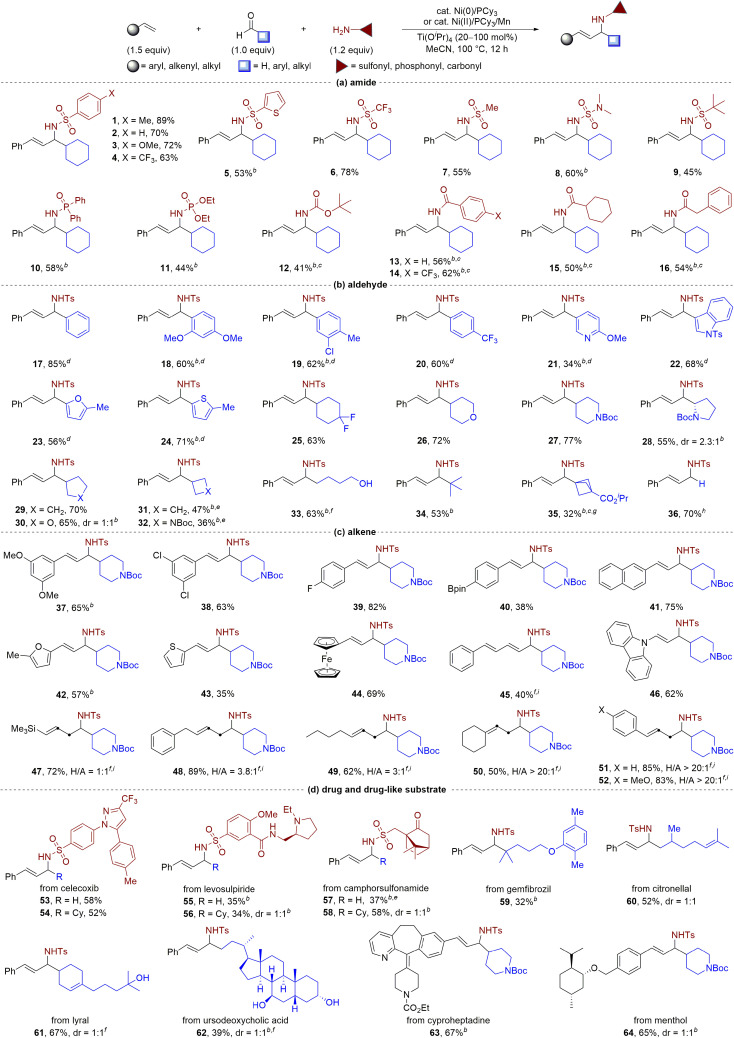

a Standard conditions unless noted, isolated yields.

b Alkene (2.0 equiv.), Ti(O^i^Pr)_4_ (1.0 equiv.).

c Ni(COD)_2_ (20 mol%), PCy_3_ (40 mol%).

d Adding 30 mg 4 Å M.S., using toluene as solvent.

e Using Ni(OAc)_2_ (10 mol%) and Mn (20 mol%) instead of Ni(COD)_2_.

f Using EtOH as solvent.

g Using ^i^PrOH as solvent.

h Without Ti(O^i^Pr)_4_, using EtOH as solvent.

i Alkene (3.0 equiv.), Ti(O^i^Pr)_4_ (1.0 equiv.), the isolated yield of the mixture of two isomers, H/A = ratio of homoallylic amine to allylic amine.

Given the abundance of allylic amine motifs in natural products and drug molecules, efficient three-component coupling reactions are essential for increasing the diversity of bioactive molecules. We investigated the reaction's performance using a series of substrates from pharmaceutical agents and bioactive molecules, such as celecoxib, levosulpiride, camphorsulfonamide, gemfibrozil, citronellal, lyral, ursodeoxycholic acid, cyproheptadine, and menthol (Table 2d). Under standard conditions, these compounds effectively underwent the three-component coupling to afford the corresponding allylamine derivatives (53–64) in satisfactory yields (32–67%) with excellent regioselectivities. Our results demonstrated the potential to construct drug-like molecules in a single step from readily available materials.

**Scheme 2:**
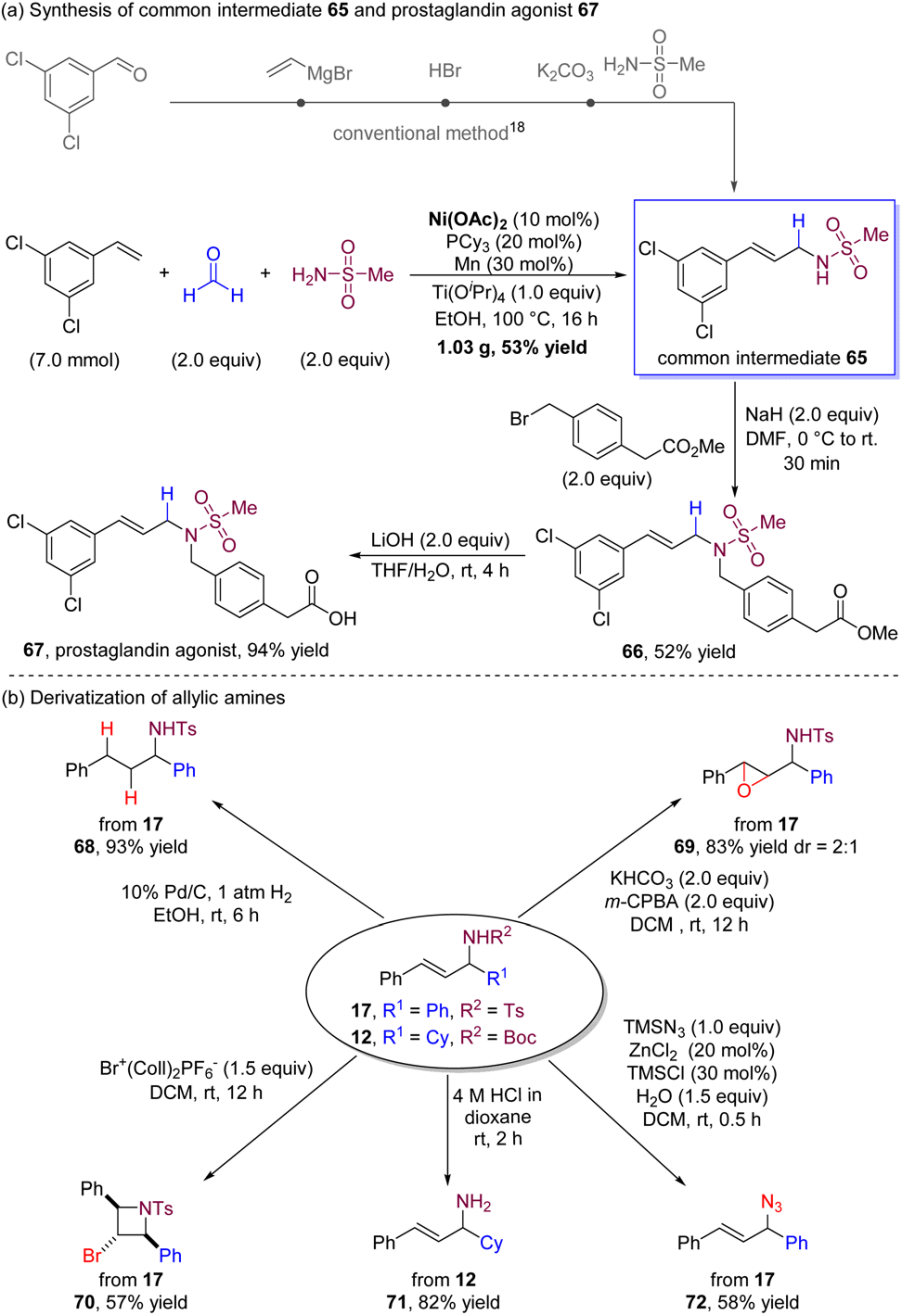
Synthetic applications.

To further demonstrate the synthetic applicability of our nickel-catalysed multicomponent coupling process, we conducted a gram-scale reaction of 3,5-dichlorostyrene with formaldehyde and methanesulfonamide (Scheme 2a). The reaction yielded multicomponent coupling product 65 in a single step with a good yield using inexpensive Ni(ii) salt as the alternative precatalyst. We note that, according to the literature,^18^ producing this product using conventional methods requires multiple steps. The resulting product 65 can serve as a common intermediate for producing a range of prostaglandin agonists,^18^ which possess the potential to reduce intraocular pressure and prevent loss and restoration of bone mass. Specifically, we demonstrate one example where compound 65 is readily benzylated and hydrolyzed to generate prostaglandin agonist 67 (for details, see ESI†).

Derivatization of the allylic amine products could also be successfully accomplished (Scheme 2b). The palladium-catalysed hydrogenation of 17 led to the corresponding saturated alkylamine 68 with a high yield. Epoxypropyl amine 69 was obtained in a high yield in the presence of *m*-CPBA and KHCO_3_. By utilizing bis(collidine)bromonium(i) hexafluorophosphate as an electrophile, the cyclization process of 17 yielded cyclic product 70 in which the two phenyl groups were in a *cis*-disubstituted azetidine. The *N-tert*-butoxycarbonyl group in 12 was successfully deprotected by treatment with 4 M HCl in dioxane to provide allylic primary amine 71. Additionally, the sulfonamide group could be easily substituted by the azide group with TMSN_3_ to deliver allylic azide 72.

**Scheme 3:**
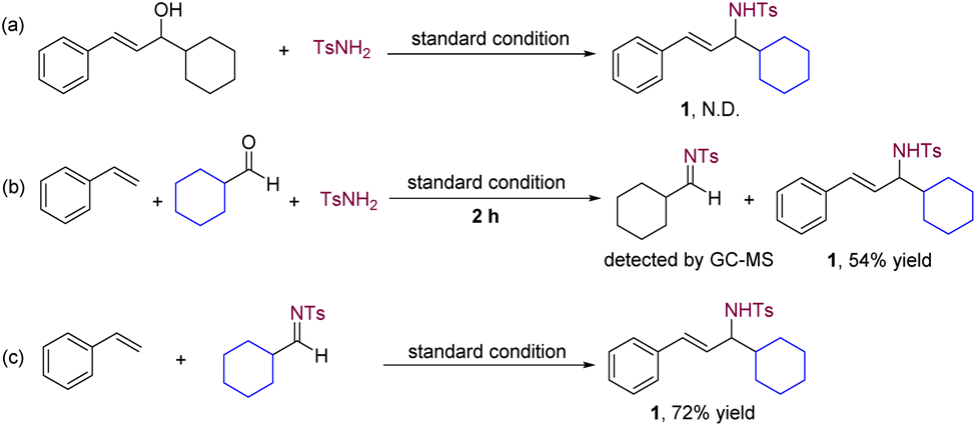
Control experiments. N.D. = not detected.

To enhance our understanding of the transformation pathway, we conducted several control experiments (Scheme 3). The use of allyl alcohol as the starting material did not yield the intended allyl amine product under the standard reaction conditions (Scheme 3a). The result excludes the possibility of allylic amination of allylic alcohol with an amide as a viable pathway.^19^ Furthermore, GC-MS analysis of the standard reaction revealed the *in situ* formation of an imine intermediate (Scheme 3b). By directly employing an imine instead of an aldehyde and an amide under the standard reaction conditions, we successfully obtained allylic amine product 1 (Scheme 3c). These findings effectively demonstrate that the multicomponent reaction involves the utilization of *in situ*-generated imines, formed from aldehydes and amides, that participate in C–C bond formation with alkenes.

**Scheme 4:**
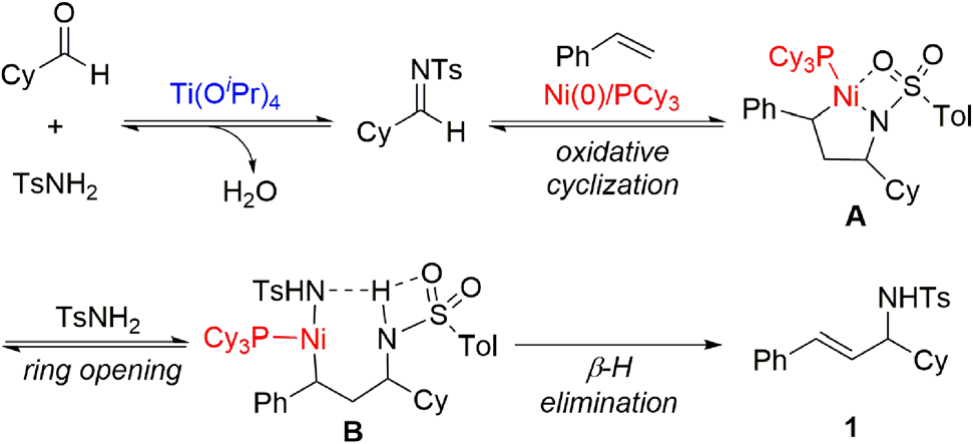
Proposed mechanism.

Based on our previous studies^8*f*,20^ and experimental results, we propose a mechanism for the multicomponent coupling reaction of alkenes, aldehydes, and imines, as illustrated in Scheme 4. The imine is generated *via* the condensation reaction of an aldehyde and an amide catalysed by Ti(O^i^Pr)_4_. Subsequently, the nickel(0) complex with both the alkene and the imine undergoes oxidative cyclization, leading to the generation of intermediate A, an aza-nickelacycle. The TsNH_2_ then promotes the ring-opening of aza-nickelacycle intermediate A by means of protonation at the N atom, followed by coordination to the Ni atom. Finally, β-hydride elimination of B releases the allylic amine product and regenerates the nickel catalyst.

## Conclusions

In summary, we have described an efficient nickel-catalysed multicomponent coupling reaction that produces structurally diverse allylic amines from easily accessible starting materials: simple alkenes, aldehydes, and amides. This one-step process does not require organometallic reagents or harshly acidic or basic conditions, making it advantageous for synthesizing drug-like amines that contain functionalities commonly found in pharmaceuticals. Furthermore, the use of readily available substrates and inexpensive Ni(ii) salt as the alternative precatalyst offers significant practical advantages. We anticipate that this nickel-catalysed multicomponent coupling reaction, which is highly selective, operationally simple, effective, and has a broad scope, will be widely adopted by researchers in both academic and industrial settings. We are currently investigating the asymmetric version of this approach in our laboratory.

## Data availability

General information, detailed experimental procedures, characterization data for all new compounds, and NMR spectra are in the ESI.†

## Author contributions

L. J. X. and Q. L. Z. conceived the study; W. G. X. performed the experiments, and prepared the ESI;† B. X. made some of the substrates; W. G. X., L. J. X., and Q. L. Z. wrote the manuscript.

## Conflicts of interest

There are no conflicts to declare.

## Supplementary Material

SC-014-D3SC03233G-s001
