# Supplementary material for: Practical synthesis of allylic amines via nickel-catalysed multicomponent coupling of alkenes, aldehydes, and amides
Source: Chem Sci. 2023 Jul 25;14(32):8644–50. doi: 10.1039/d3sc03233g (PMC10430692; doi:10.1039/d3sc03233g)
Supplement: SC-014-D3SC03233G-s001 [file SC-014-D3SC03233G-s001.pdf]

Electronic Supplementary Material (ESI) for Chemical Science.
This journal is © The Royal Society of Chemistry 2023

Supporting Information for:

Practical synthesis of allylic amines via nickel-catalysed multicomponent coupling of alkenes, aldehydes, and amides

Wei-Guo Xiao, Bin Xuan, Li-Jun Xiao,* Qi-Lin Zhou
State Key Laboratory and Institute of Elemento-Organic Chemistry, College of Chemistry, Frontiers Science Center for New Organic Matter, Nankai University, Tianjin 300071, China
E-mail: ljxiao@nankai.edu.cn

CONTENTS:

1. **General Information S2**
2. **Optimization of Reaction Conditions S3**
3. **General Procedure and the Data for Products …………………………..S6**
4. **Synthetic Applications S42**
5. **Transformations of Products S44**
6. **NMR Spectra of New Compounds S47**
7. **Control Experiments S121**
8. **Reference S122**

1. General Information

Unless mentioned otherwise, all manipulations were performed in an argon-filled glove box (MBRAUN LABstar) or using standard Schlenk techniques. NMR spectra were recorded on a Bruker AV 400 spectrometer at 400 MHz (^{1}H NMR), 101 MHz (^{13}C NMR), 376 MHz (^{19}F NMR). Chemical shifts were reported in ppm relative to internal TMS for ^{1}H NMR data, deuterated solvent for ^{13}C NMR data, respectively. Data are presented in the following space: chemical shift, multiplicity, coupling constant in hertz (Hz), and signal area integration in natural numbers. Melting points were measured on a RY-I apparatus and uncorrected. High-resolution mass spectra were recorded on an IonSpec FT-ICR mass spectrometer with ESI resource. All the solvents used for reactions were distilled after drying over an appropriate drying agent. [Ni(COD)$_2$] was purchased from Strem Chemicals. Other commercially available reagents were purchased from Acros, Sigma-Aldrich and Alfa Aesar Chemical Company. All of the liquid substrates were distilled before used.

2. Optimization of Reaction Conditions

Table S1: Evaluation of the solvents[a]

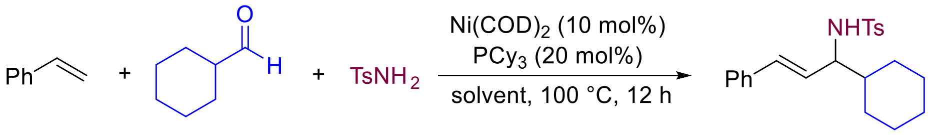

1

entry	solvent	yield of **1** (%)[b]
1	MeOH	44
2	EtOH	26
3	^{i}PrOH	33
4	^{t}BuOH	33
5	toluene	18
6	THF	trace
7	1,4-dioxane	30
**8**	**MeCN**	**50**
9	DMF	trace
10	DMSO	11
11	DMA	30

[a] Reaction conditions: alkene (0.3 mmol), aldehyde (0.2 mmol), amide (0.24 mmol), $Ni(COD)_2$ (10 mol%), PCy_3 (20 mol%), solvent (0.5 mL). [b] The yields were determined by ^1H NMR analysis using dibromomethane as an internal standard.

Table S2: Evaluation of the ligands[a]

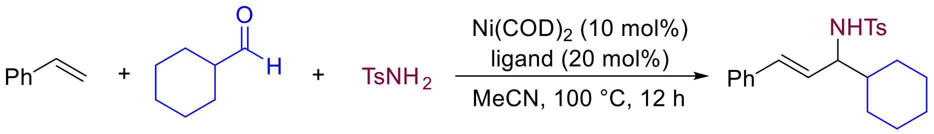

1

entry	ligand	yield of **1** (%)[b]
**1**	**PCy_3**	**50**
2	PPh_3	trace
3	PBu_3	33
4	P^tBu_3	0
5	dppe	0
6	dcype	trace
7	dppf	trace
8	BINAP	0
9	IPr	0

[a] Reaction conditions: alkene (0.3 mmol), aldehyde (0.2 mmol), amide (0.24 mmol), $Ni(COD)_2$ (10 mol%), ligand (20 mol%), solvent (0.5 mL). [b] The yields were determined by ^1H NMR analysis using dibromomethane as an internal standard.

Table S3: Evaluation of additives[a]

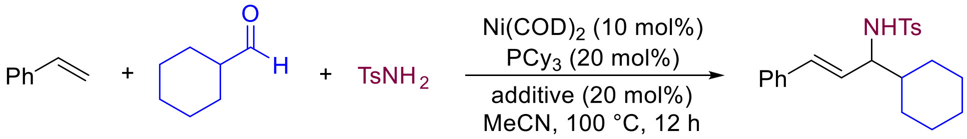

1

entry	additive	yield of **1** (%)[b]
1	–	50
2	$PhB(OH)_2$	72
3	PhCOOH	trace
4	LiCl	10
5	$MgCl_2$	trace
6	$MgSO_4$	48
**7**	**$Ti(^iOPr)_4$**	**94**
8	4Å MS (30 mg)	81

[a] Reaction conditions: alkene (0.3 mmol), aldehyde (0.2 mmol), amide (0.24 mmol), $Ni(COD)_2$ (10 mol%), PCy_3 (20 mol%), additive (20 mol%), solvent (0.5 mL). [b] The yields were determined by ^1H NMR analysis using dibromomethane as an internal standard.

Table S4: Evaluation of temperature[a]

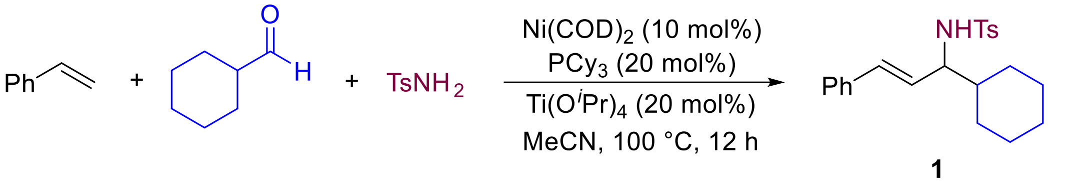

entry	temp. (°C)	yield of **1** (%)
**1**	**100**	**94**
2	80	84
3	60	76

[a] Reaction conditions: alkene (0.3 mmol), aldehyde (0.2 mmol), amide (0.24 mmol), $Ni(COD)_2$ (10 mol%), PCy_3 (20 mol%), additive (20 mol%), solvent (0.5 mL). [b] The yields were determined by ^1H NMR analysis using dibromomethane as an internal standard.

Table S5: Evaluation of the Ni(II) precursors[a]

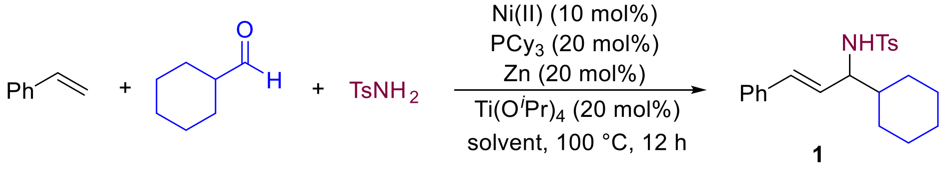

entry	Ni(II) precursor	yield of **1** (%)[b]
1	$NiBr_2$•DME	36
2	$Ni(BF)_4$•$6H_2O$	38
3	$Ni(OTf)_2$	28
4	$Ni(OAc)_2$	66
**5**[c]	**$Ni(OAc)_2$**	**83**
6	Ni(OAc)•$4H_2O$	39
7	$NiSO_4$	15
8	$NiCl_2$	33
9	$NiCl_2$•$6H_2O$	36
10	$NiCl_2$•DME	43
11	$Ni(acac)_2$	trace

[a] Reaction conditions: alkene (0.3 mmol), aldehyde (0.2 mmol), amide (0.24 mmol), Ni(II) (10 mol%), PCy_3 (20 mol%), Zn (20 mol%), $Ti(O^iPr)_4$ (20 mol%), solvent (0.5 mL). [b] The yields were determined by ^1H NMR analysis using dibromomethane as an internal standard. [c] Using Mn (20 mol%) instead of Zn (20 mol%).

3. General Procedure and the Data for Products:

General Procedure A:

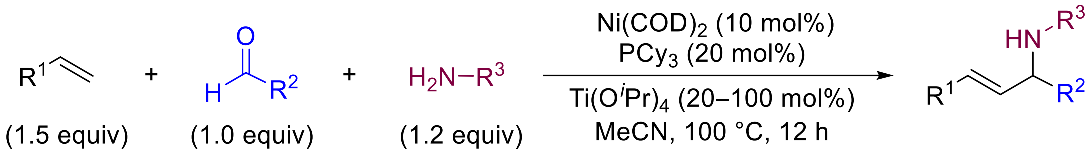

In an N_2-filled glove-box, an oven-dried sealed tube was charged with a stir bar, $Ni(COD)_2$ (5.5 mg, 0.02 mmol), PCy_3 (11.2 mg, 0.04 mmol), amide (0.24 mmol), anhydrous acetonitrile (0.5 mL) and aldehyde (0.2 mmol), $Ti(O^iPr)_4$ (20–100 mol%), alkene (0.3 mmol) were injected into the tube. The tube was then sealed with a Teflon-lined screw cap, removed from the glove box, and stirred at 100 °C for 12 h. After cooled to room temperature, the mixture was concentrated and purified by column chromatography on silica gel. In some cases, the crude product was examined by ^1H NMR to obtain an isomeric ratio (dr) before further purification.

General Procedure B:

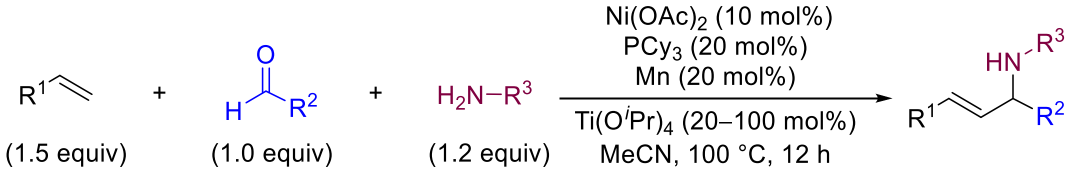

In an N_2-filled glove-box, an oven-dried sealed tube was charged with a stir bar, $Ni(OAc)_2$ (3.5 mg, 0.02 mmol), PCy_3 (11.2 mg, 0.04 mmol), Mn (2.2 mg, 20 mol%), amide (0.24 mmol), anhydrous acetonitrile (0.5 mL) and aldehyde (0.2 mmol), $Ti(O^iPr)_4$ (20–100 mol%), alkene (0.3 mmol). The tube was then sealed with a Teflon-lined screw cap, removed from the glove box, and stirred at 100 °C for 12 h. After cooled to room temperature, the mixture was concentrated and purified by column chromatography on silica gel. In some cases, the crude product was examined by ^1H NMR to obtain an isomeric ratio (dr) before further purification.

(*E*)-N-(1-cyclohexyl-3-phenylallyl)-4-methylbenzenesulfonamide (1)[1]

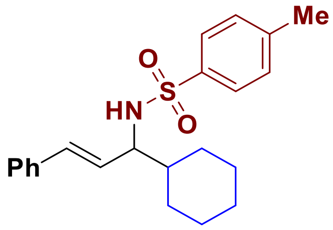

From styrene (35 µL, 0.30 mmol, 1.5 equiv), cyclohexanecarboxaldehyde (24 µL, 0.2 mmol, 1.0 equiv) and *p*-toluenesulfonamide (41.0 mg, 0.24 mmol, 1.2 equiv) the title compound was prepared following the general procedure **A** using Ni(COD)$_2$ (5.5 mg, 10 mol%), PCy$_3$ (11.2 mg, 20 mol%), Ti(O^{i}Pr)$_4$(12 µL, 20 mol%), anhydrous acetonitrile (0.5 mL). The reaction mixture was stirred for 12 h at 100 °C. The crude material was purified by flash column chromatography (petroleum ether/EtOAc = 6:1) to provide the title compound as a white solid in 89% yield (65.7 mg).
^{1}H NMR (400 MHz, CDCl$_3$) δ 7.71 (d, *J* = 7.9 Hz, 2H), 7.26 – 7.17 (m, 3H), 7.13 (d, *J* = 7.9 Hz, 2H), 7.05 (d, *J* = 7.3 Hz, 2H), 6.03 (d, *J* = 15.8 Hz, 1H), 5.69 (dd, *J* = 15.8, 8.1 Hz, 1H), 4.97 (d, *J* = 8.5 Hz, 1H), 3.70 (q, *J* = 7.8 Hz, 1H), 2.24 (s, 3H), 1.81 (d, *J* = 13.0 Hz, 1H), 1.65 (dd, *J* = 27.4, 12.1 Hz, 4H), 1.50 – 1.39 (m, 1H), 1.13 (tt, *J* = 21.0, 10.6 Hz, 3H), 1.04 – 0.90 (m, 2H).
^{13}C NMR (101 MHz, CDCl$_3$) δ 250.8, 143.0, 138.1, 136.3, 132.0, 129.4, 128.3, 127.5, 127.3, 127.2, 126.2, 77.3, 77.0, 76.7, 61.4, 42.9, 29.0, 29.0, 26.2, 25.9, 25.6, 21.3.
HRMS (ESI) calcd. for [$C_{22}H_{227}NO_2S$, M+Na]$^+$: 392.1660, found: 392.1656.

(*E*)-N-(1-cyclohexyl-3-phenylallyl)benzenesulfonamide (2)

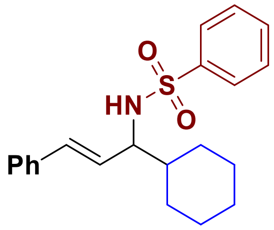

From styrene (35 µL, 0.30 mmol, 1.5 equiv), cyclohexanecarboxaldehyde (24 µL, 0.20 mmol, 1.0 equiv) and benzenesulfonamide (37.7 mg, 0.24 mmol, 1.2 equiv) the title compound was prepared following the general procedure **A** using Ni(COD)$_2$ (5.5 mg, 10 mol%), PCy$_3$ (11.2 mg, 20 mol%), Ti(O^{i}Pr)$_4$ (12 µL, 20 mol%), anhydrous acetonitrile (0.5 mL). The reaction mixture was stirred for 12 h at 100 °C. The crude material was purified by flash column chromatography (petroleum ether/EtOAc = 6:1) to provide the title compound as a white solid in 70% yield (49.7 mg). m.p.: 114−115 °C.
^{1}H NMR (400 MHz, CDCl$_3$) δ 7.83 – 7.71 (m, 2H), 7.35 – 7.21 (m, 3H), 7.19 – 7.04 (m, 3H), 7.01 – 6.92 (m, 2H), 6.01 (d, *J* = 15.8 Hz, 1H), 5.66 (dd, *J* = 15.8, 8.0 Hz, 1H), 5.13 (d, *J* = 8.4 Hz, 1H), 3.75 – 3.59 (m, 1H), 1.73 (d, *J* = 12.0 Hz, 1H), 1.69 – 1.47 (m, 4H), 1.42 – 1.32 (m, 1H), 1.12 – 0.84 (m, 5H).
^{13}C NMR (101 MHz, CDCl$_3$) δ 141.2, 132.2, 132.1, 128.8, 128.3, 127.6, 127.3, 127.2, 126.3, 61.4, 42.9, 29.0, 29.0, 26.2, 26.0, 25.9.
HRMS (ESI) calcd. for [$C_{21}H_{25}NO_2S$, M-H]$^-$: 354.1536, found: 354.1530.

(*E*)-N-(1-cyclohexyl-3-phenylallyl)-4-methoxybenzenesulfonamide (3)

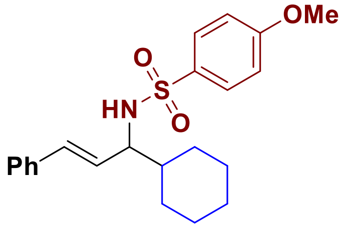

From styrene (35 μL, 0.30 mmol, 1.5 equiv), cyclohexanecarboxaldehyde (24 μL, 0.20 mmol, 1.0 equiv) and 4-methoxybenzenesulfonamide (44.9 mg, 0.24 mmol, 1.2 equiv) the title compound was prepared following the general procedure **A** using Ni(COD)$_2$ (5.5 mg, 10 mol%), PCy$_3$ (11.2 mg, 20 mol%), Ti(O^{i}Pr)$_4$ (12 μL, 20 mol%), anhydrous acetonitrile (0.5 mL). The reaction mixture was stirred for 12 h at 100 °C. The crude material was purified by flash column chromatography (petroleum ether/EtOAc = 5:1) to provide the title compound as a white solid in 72% yield (55.5 mg). m.p.: 148−149 °C.
^{1}H NMR (400 MHz, CDCl$_3$) δ 7.81 – 7.71 (m, 2H), 7.22 (td, *J* = 12.1, 6.0 Hz, 3H), 7.09 – 7.05 (m, 2H), 6.81 – 6.75 (m, 2H), 6.04 (d, *J* = 15.8 Hz, 1H), 5.70 (dd, *J* = 15.9, 8.2 Hz, 1H), 5.12 (d, *J* = 8.4 Hz, 1H), 3.66 (s, 3H), 1.82 (d, *J* = 12.9 Hz, 1H), 1.75 – 1.59 (m, 4H), 1.49 – 1.39 (m, 1H), 1.15 (ddd, *J* = 27.0, 13.2, 6.0 Hz, 3H), 1.04 – 0.92 (m, 2H).
^{13}C NMR (101 MHz, CDCl$_3$) δ 162.5, 136.4, 132.0, 129.4, 128.3, 127.5, 127.4, 126.2, 113.9, 61.5, 55.4, 42.9, 29.1, 29.0, 26.2, 25.9.
HRMS (ESI) calcd. for [$C_{22}H_{27}NO_3S$, M-H]$^-$: 384.1642, found: 384.1635.

(*E*)-N-(1-cyclohexyl-3-phenylallyl)-4-(trifluoromethyl)benzenesulfonamide (4)

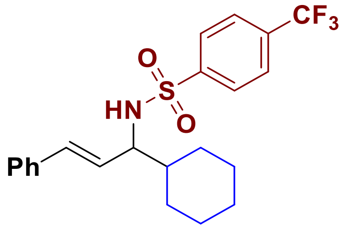

From styrene (35 μL, 0.30 mmol, 1.5 equiv), cyclohexanecarboxaldehyde (24 μL, 0.20 mmol, 1.0 equiv) and 4-(trifluoromethyl)benzenesulfonamide (54.0 mg, 0.24 mmol, 1.2 equiv) the title compound was prepared following the general procedure **A** using Ni(COD)$_2$ (5.5 mg, 10 mol%), PCy$_3$ (11.2 mg, 20 mol%), Ti(O^{i}Pr)$_4$ (12 μL, 20 mol%), anhydrous acetonitrile (0.5 mL). The reaction mixture was stirred for 12 h at 100 °C. The crude material was purified by flash column chromatography (petroleum ether/EtOAc = 5:1) to provide the title compound as a white solid in 63% yield (53.3 mg). m.p.: 111−112 °C.
^{1}H NMR (400 MHz, CDCl$_3$) δ 7.95 (d, *J* = 8.1 Hz, 2H), 7.59 (d, *J* = 8.2 Hz, 2H), 7.22 (s, 1H), 7.21 (s, 2H), 7.00 (dd, *J* = 7.2, 2.2 Hz, 2H), 6.07 (d, *J* = 15.8 Hz, 1H), 5.63 (dd, *J* = 15.8, 8.4 Hz, 1H), 5.15 (d, *J* = 8.3 Hz, 1H), 3.78 (q, *J* = 7.9 Hz, 1H), 1.83 (d, *J* = 12.8 Hz, 1H), 1.74 – 1.59 (m, 4H), 1.47 (ddt, *J* = 15.0, 11.3, 5.2 Hz, 1H), 1.30 – 1.05 (m, 3H), 1.00 (qd, *J* = 12.1, 6.2 Hz, 2H).

^{13}C NMR (101 MHz, $CDCl_3$) δ 144.8, 135.7, 132.7, 129.5(q, *J* = 272 Hz), 128.5, 127.9, 127.8, 126.7, 126.1, 125.9(q, *J* = 3.8 Hz), 61.9, 29.1(d, *J* = 4.9 Hz), 26.1, 25.8.
^{19}F NMR (376 MHz, $CDCl_3$) δ -63.2.
HRMS (ESI) calcd. for $[C_{22}H_{24}F_3NO_2S, M-H]^-$: 422.1407, found: 422.1405.

(*E*)-N-(1-cyclohexyl-3-phenylallyl)thiophene-2-sulfonamide (5)

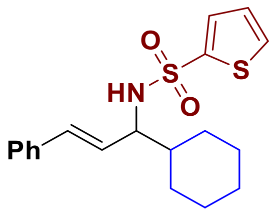

From styrene (46 µL, 0.40 mmol, 2.0 equiv), cyclohexanecarboxaldehyde (24 µL, 0.20 mmol, 1.0 equiv) and thiophene-2-sulfonamide (39.1 mg, 0.24 mmol, 1.2 equiv) the title compound was prepared following the general procedure **A** using $Ni(COD)_2$ (5.5 mg, 10 mol%), PCy_3 (11.2 mg, 20 mol%), $Ti(O^iPr)_4$ (60 µL, 100 mol%), anhydrous acetonitrile (0.5 mL). The reaction mixture was stirred for 12 h at 100 °C. The crude material was purified by flash column chromatography (petroleum ether/EtOAc = 6:1) to provide the title compound as a white solid in 53% yield (38.3 mg). m.p.: 101−102 °C.
^{1}H NMR (400 MHz, $CDCl_3$) δ 7.95 (d, *J* = 8.1 Hz, 2H), 7.59 (d, *J* = 8.2 Hz, 2H), 7.22 (s, 1H), 7.21 (s, 2H), 7.00 (dd, *J* = 7.2, 2.2 Hz, 2H), 6.07 (d, *J* = 15.8 Hz, 1H), 5.63 (dd, *J* = 15.8, 8.4 Hz, 1H), 5.15 (d, *J* = 8.3 Hz, 1H), 3.78 (q, *J* = 7.9 Hz, 1H), 1.83 (d, *J* = 12.8 Hz, 1H), 1.74 – 1.59 (m, 4H), 1.47 (ddt, *J* = 15.0, 11.3, 5.2 Hz, 1H), 1.30 – 1.05 (m, 3H), 1.00 (qd, *J* = 12.1, 6.2 Hz, 2H).
^{13}C NMR (101 MHz, $CDCl_3$) δ 144.8, 135.7, 132.7, 128.5, 127.9, 127.8, 126.7, 126.1, 125.9, 125.9, 61.9, 42.7, 29.1, 29.1, 26.1, 25.8.
HRMS (ESI) calcd. for $[C_{19}H_{23}NO_2S2, M-H]^-$: 360.1097, found: 360.1095.

(*E*)-N-(1-cyclohexyl-3-phenylallyl)-1,1,1-trifluoromethanesulfonamide (6)

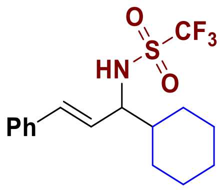

From styrene (35 µL, 0.30 mmol, 1.5 equiv), cyclohexanecarboxaldehyde (24 µL, 0.20 mmol, 1.0 equiv) and trifluoromethanesulfonamide (35.8 mg, 0.24 mmol, 1.2 equiv) the title compound was prepared following the general procedure **A** using $Ni(COD)_2$ (5.5 mg, 10 mol%), PCy_3 (11.2 mg, 20 mol%), $Ti(O^iPr)_4$ (12 µL, 20 mol%), anhydrous acetonitrile (0.5 mL). The reaction mixture was stirred for 12 h at 100 °C. The crude material was purified by flash column chromatography (petroleum ether/EtOAc = 6:1) to provide the title compound as a white solid in 78% yield (54.1 mg). m.p.: 123−124 °C.
^{1}H NMR (400 MHz, $CDCl_3$) δ 7.28 – 7.10 (m, 4H), 6.41 (d, *J* = 15.8 Hz, 1H), 5.93 (dd, *J* = 15.9, 7.7 Hz, 1H), 3.89 (t, *J* = 7.1 Hz, 1H), 1.74 (d, *J* = 13.6 Hz, 1H), 1.64 (t, *J* = 10.0 Hz, 2H), 1.55 (dd, *J* = 9.7, 6.1 Hz, 1H), 1.44 (dddt, *J* = 12.2, 10.0, 6.6, 3.2 Hz, 1H),

1.14 (s, 1H), 1.14 – 0.84 (m, 4H).
^{13}C NMR (101 MHz, $CDCl_3$) δ 136.0, 132.8, 128.6, 128.1, 126.5, 126.4, 119.53 (q, *J* = 323 Hz), 63.1, 43.0, 29.2, 28.8, 26.0, 25.8.
^{19}F NMR (376 MHz, $CDCl_3$) δ -77.2.
HRMS (ESI) calcd. for $[C_{16}H_{20}F_3NO_2S, M-H]^-$: 346.1094, found: 346.1097.

(*E*)-N-(1-cyclohexyl-3-phenylallyl)methanesulfonamide (7)

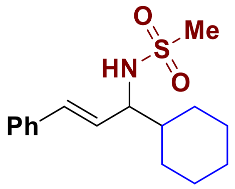

From styrene (35 μL, 0.30 mmol, 1.5 equiv), cyclohexanecarboxaldehyde (24 μL, 0.20 mmol, 1.0 equiv) and methanesulfonamide (22.8 mg, 0.24 mmol, 1.2 equiv) the title compound was prepared following the general procedure **A** using $Ni(COD)_2$ (5.5 mg, 10 mol%), PCy_3 (11.2 mg, 20 mol%), $Ti(O^iPr)_4$ (12 μL, 20 mol%), anhydrous acetonitrile (0.5 mL). The reaction mixture was stirred for 12 h at 100 °C. The crude material was purified by flash column chromatography (petroleum ether/EtOAc = 6:1) to provide the title compound as a white solid in 55% yield (32.2 mg). m.p.: 122−123 °C.
^{1}H NMR (400 MHz, $CDCl_3$) δ 7.25 (ddd, *J* = 27.7, 19.4, 7.1 Hz, 5H), 6.49 (d, *J* = 15.9 Hz, 1H), 6.00 (dd, *J* = 15.9, 8.3 Hz, 1H), 4.81 (d, *J* = 8.6 Hz, 1H), 3.79 (q, *J* = 8.0 Hz, 1H), 2.85 (s, 3H), 1.82 (d, *J* = 12.8 Hz, 1H), 1.68 (h, *J* = 7.0, 5.3 Hz, 4H), 1.59 (d, *J* = 11.9 Hz, 1H), 1.44 (dddt, *J* = 11.5, 9.5, 6.5, 3.2 Hz, 1H), 1.19 – 0.88 (m, 6H).
^{13}C NMR (101 MHz, $CDCl_3$) δ 136.1, 132.4, 128.7, 128.2, 128.0, 126.4, 61.5, 42.9, 42.1, 29.3, 29.2, 26.2, 25.9.
HRMS (ESI) calcd. for $[C_{16}H_{23}NO_2S, M-H]^-$: 292.1376, found: 292.1377.

(*E*)-N-(1-cyclohexyl-3-phenylallyl))-N,N-dimethylsulfonamide (8)

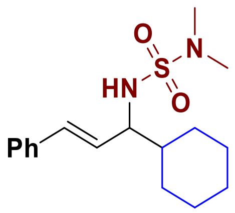

From styrene (46 μL, 0.40 mmol, 2.0 equiv), cyclohexanecarboxaldehyde (24 μL, 0.20 mmol, 1.0 equiv) and N,N-dimethylsulfamide (29.8 mg, 0.24 mmol, 1.2 equiv) the title compound was prepared following the general procedure **A** using $Ni(COD)_2$ (5.5 mg, 10 mol%), PCy_3 (11.2 mg, 20 mol%), $Ti(O^iPr)_4$ (60 μL, 100 mol%), anhydrous acetonitrile (0.5 mL). The reaction mixture was stirred for 12 h at 100 °C. The crude material was purified by flash column chromatography (petroleum ether/EtOAc = 6:1) to provide the title compound as a white solid in 60% yield (38.6 mg). m.p.: 88−89 °C.
^{1}H NMR (400 MHz, $CDCl_3$) δ 7.41 – 7.28 (m, 4H), 7.30 – 7.20 (m, 1H), 6.51 (d, *J* = 15.8 Hz, 1H), 6.06 (dd, *J* = 15.9, 8.1 Hz, 1H), 4.44 (d, *J* = 8.0 Hz, 1H), 3.77 (td, *J* = 8.1, 6.2 Hz, 1H), 2.76 (s, 6H), 1.86 (d, *J* = 12.9 Hz, 1H), 1.73 (d, *J* = 3.4 Hz, 1H), 1.66 (d,

J = 12.1 Hz, 2H), 1.53 (tdt, J = 11.7, 6.0, 3.0 Hz, 1H), 1.31 – 0.98 (m, 4H).
^{13}C NMR (101 MHz, $CDCl_3$) δ 136.5, 131.9, 128.6, 128.6, 127.7, 126.4, 61.5, 43.1, 38.0, 29.3, 29.0, 26.2, 26.0.
HRMS (ESI) calcd. for $[C_{17}H_{26}N_2O_2S, M+Na]^+$: 345.1607, found: 345.1610.

(*E*)-N-(1-cyclohexyl-3-phenylallyl)-2-methylpropane-2-sulfonamide (9)

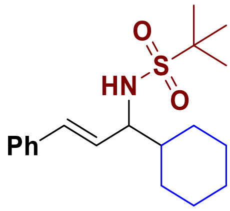

From styrene (35 μL, 0.30 mmol, 1.5 equiv), cyclohexanecarboxaldehyde (24 μL, 0.20 mmol, 1.0 equiv) and *tert*-butylsulfonamide (32.9 mg, 0.24 mmol, 1.2 equiv) the title compound was prepared following the general procedure **A** using Ni(COD)$_2$ (5.5 mg, 10 mol%), PCy$_3$ (11.2 mg, 20 mol%), Ti(O^{i}Pr)$_4$ (12 μL, 20 mol%), anhydrous acetonitrile (0.5 mL). The reaction mixture was stirred for 12 h at 100 °C. The crude material was purified by flash column chromatography (petroleum ether/EtOAc = 6:1) to provide the title compound as a white solid in 45% yield (30.2 mg). m.p.: 175−177 °C.
^{1}H NMR (400 MHz, $CDCl_3$) δ 7.28 – 7.08 (m, 5H), 6.38 (d, J = 15.9 Hz, 1H), 5.97 (dd, J = 15.9, 7.6 Hz, 1H), 4.00 (d, J = 9.4 Hz, 1H), 3.80 (dt, J = 8.9, 6.6 Hz, 1H), 1.76 (d, J = 13.1 Hz, 1H), 1.64 (d, J = 11.4 Hz, 3H), 1.54 (d, J = 8.9 Hz, 2H), 1.42 (ddt, J = 11.6, 8.8, 4.3 Hz, 1H), 1.26 (s, 9H), 1.23 – 0.87 (m, 4H).
^{13}C NMR (101 MHz, $CDCl_3$) δ 131.6, 128.9, 128.6, 127.7, 126.4, 61.9, 59.8, 44.1, 29.4, 29.1, 26.2, 26.1, 26.1, 24.3.
HRMS (ESI) calcd. for $[C_{19}H_{29}NO_2S, M-H]^-$: 334.1846, found: 334.1844.

(*E*)-N-(1-cyclohexyl-3-phenylallyl)-P,P-diphenylphosphinicamide (10)

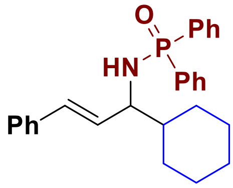

From styrene (46 μL, 0.40 mmol, 2.0 equiv), cyclohexanecarboxaldehyde (24 μL, 0.20 mmol, 1.0 equiv) and diphenylphosphinamide (52 mg, 0.24 mmol, 1.2 equiv) the title compound was prepared following the general procedure **A** using Ni(COD)$_2$ (5.5 mg, 10 mol%), PCy$_3$ (11.2 mg, 20 mol%), Ti(O^{i}Pr)$_4$ (60 μL, 100 mol%), anhydrous acetonitrile (0.5 mL). The reaction mixture was stirred for 12 h at 100 °C. The crude material was purified by flash column chromatography (petroleum ether/EtOAc = 1:1) to provide the title compound as a white solid in 58% yield (48.1 mg). m.p.: 193−194 °C.
^{1}H NMR (400 MHz, $CDCl_3$) δ 7.93 (td, J = 11.3, 7.4 Hz, 4H), 7.57 – 7.35 (m, 6H), 7.36 – 7.19 (m, 2H), 6.27 (d, J = 15.8 Hz, 1H), 6.10 (dd, J = 15.9, 7.5 Hz, 1H), 3.70 – 3.57 (m, 1H), 3.12 (dd, J = 9.9, 6.6 Hz, 1H), 1.87 (d, J = 12.8 Hz, 1H), 1.70 – 1.55 (m, 2H), 1.33 – 1.00 (m, 5H).

^{13}**C NMR** (101 MHz, $CDCl_3$) 136.9, 134.0, 133.2, 132.7 (d, J = 9.8 Hz), 132.1 (d, J = 10.3 Hz), 131.9 (d, J = 9.2 Hz), 131.6 (d, J = 7.8 Hz), 130.9, 130.4 (d, J = 5.0 Hz), 128.5, 128.4 (d, J = 7.5 Hz), 128.2, 127.3, 126.3, 58.8, 44.4 (d, J = 4.4 Hz), 29.6, 28.7, 26.4, 26.2.
^{31}**P NMR** (162 MHz, $CDCl_3$) δ 22.03.
HRMS (ESI) calcd. for $[C_{27}H_{30}NOP, M+Na]^+$: 438.1957, found: 438.1960.

(*E*)-diethyl-(1-cyclohexyl-3-phenylallyl)phosphoramidate (11)

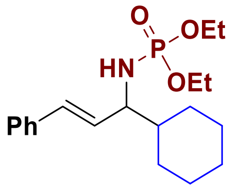

From styrene (46 µL, 0.40 mmol, 2.0 equiv), cyclohexanecarboxaldehyde (24 µL, 0.20 mmol, 1.0 equiv) and diethylphosphoramidate (36.7 mg, 0.24 mmol, 1.2 equiv) the title compound was prepared following the general procedure **A** using $Ni(COD)_2$ (5.5 mg, 10 mol%), PCy_3 (11.2 mg, 20 mol%), $Ti(O^iPr)_4$ (60 µL, 100 mol%), anhydrous acetonitrile (0.5 mL). The reaction mixture was stirred for 12 h at 100 °C. The crude material was purified by flash column chromatography (petroleum ether/EtOAc = 1:1) to provide the title compound as a white solid in 44% yield (30.8 mg). m.p.: 108−110 °C.
^{1}**H NMR** (400 MHz, $CDCl_3$) δ 7.28 – 7.16 (m, 3H), 7.18 – 7.09 (m, 1H), 6.39 (d, J = 15.7 Hz, 1H), 5.98 (dd, J = 15.9, 7.3 Hz, 1H), 3.94 (ddh, J = 24.0, 16.6, 9.4, 8.5 Hz, 4H), 3.47 (p, J = 8.1 Hz, 1H), 2.63 (t, J = 10.4 Hz, 1H), 1.73 (d, J = 12.6 Hz, 1H), 1.65 (s, 1H), 1.35 (s, 1H), 1.21 (t, J = 7.2 Hz, 3H), 1.11 (q, J = 13.5, 10.3 Hz, 4H), 1.09 – 0.75 (m, 3H).
^{13}**C NMR** (101 MHz, $CDCl_3$) δ 136.9, 130.6 (d, J = 3.2 Hz), 130.4, 128.5, 127.4, 126.2, 62.2 (t, J = 6.0 Hz), 58.9, 43.9 (d, J = 6.5 Hz), 29.0 (d, J = 7.7 Hz), 26.4, 26.1, 16.2 (dd, J = 13.2, 7.4 Hz).
^{31}**P NMR** (162 MHz, $CDCl_3$) δ 8.15.
HRMS (ESI) calcd. for $[C_{19}H_{30}NO_3P, M+Na]^+$: 374.1855, found: 374.1860.

(*E*)-*tert*-butyl-(1-cyclohexyl-3-phenylallyl)carbamate (12)

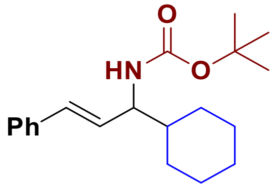

From styrene (46 µL, 0.40 mmol, 2.0 equiv), cyclohexanecarboxaldehyde (24 µL, 0.20 mmol, 1.0 equiv) and *tert*-butyl-carbamate (28.1 mg, 0.24 mmol, 1.2 equiv) the title compound was prepared following the general procedure **A** using $Ni(COD)_2$ (11.0 mg, 20 mol%), PCy_3 (22.4 mg, 40 mol%), $Ti(O^iPr)_4$ (60 µL, 100 mol%), anhydrous acetonitrile (0.5 mL). The reaction mixture was stirred for 12 h at 100 °C. The crude material was purified by flash column chromatography (petroleum ether/EtOAc = 10:1) to provide the title compound as a white solid in 41% yield (25.8 mg). m.p.: 121−122 °C.

^{1}H NMR (400 MHz, $CDCl_3$) δ 7.40 – 7.25 (m, 4H), 7.26 – 7.14 (m, 1H), 6.48 (d, *J* = 15.9 Hz, 1H), 6.07 (dd, *J* = 15.9, 6.9 Hz, 1H), 4.62 (s, 1H), 1.77 (td, *J* = 12.2, 9.6, 4.4 Hz, 4H), 1.70 – 1.61 (m, 1H), 1.47 (s, 1H), 1.45 (s, 8H), 1.31 – 0.94 (m, 4H).
^{13}C NMR (101 MHz, $CDCl_3$) δ 130.5, 129.3, 128.5, 127.3, 126.3, 79.2, 42.8, 29.4, 28.9, 28.4, 26.3, 26.1, 26.1.
HRMS (ESI) calcd. for $[C_{20}H_{29}NO_2, M+Na]^+$: 338.2090, found: 338.2095.

(*E*)-N-(1-cyclohexyl-3-phenylallyl)benzamide (13)

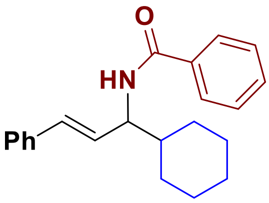

From styrene (46 µL, 0.40 mmol, 2.0 equiv), cyclohexanecarboxaldehyde (24 µL, 0.20 mmol, 1.0 equiv) and benzamide (29.0 mg, 0.24 mmol, 1.2 equiv) the title compound was prepared following the general procedure **A** using $Ni(COD)_2$ (11 mg, 20 mol%), PCy_3 (22.4 mg, 40 mol%), $Ti(O^iPr)_4$ (60 µL, 100 mol%). anhydrous acetonitrile (0.5 mL). The reaction mixture was stirred for 12 h at 100 °C. The crude material was purified by flash column chromatography (petroleum ether/acetone = 7:1) to provide the title compound as a white solid in 56% yield (35.8 mg). m.p.: 153−155 °C
^{1}H NMR (600 MHz, $CDCl_3$) δ 7.80 (d, *J* = 7.5 Hz, 2H), 7.48 (dt, *J* = 36.3, 7.5 Hz, 3H), 7.39 – 7.28 (m, 4H), 7.22 (t, *J* = 7.3 Hz, 1H), 6.58 (d, *J* = 15.8 Hz, 1H), 6.19 (dd, *J* = 16.0, 7.1 Hz, 2H), 4.71 (q, *J* = 7.5 Hz, 1H), 1.89 – 1.74 (m, 4H), 1.70 – 1.62 (m, 2H), 1.25 (tt, *J* = 12.7, 3.4 Hz, 2H), 1.20 – 1.08 (m, 3H).
^{13}C NMR (151 MHz, $CDCl_3$) δ 166.7, 136.7, 134.9, 131.5, 131.4, 128.6, 128.5, 128.4, 127.5, 126.9, 126.4, 56.3, 42.7, 29.6, 29.1, 26.3, 26.1.
HRMS (ESI) calcd. for $[C_{22}H_{25}NO, M+Na]^+$: 342.1834, found: 342.1823.

(*E*)-N-(1-cyclohexyl-3-phenylallyl)-4-(trifluoromethyl)-benzamide (14)

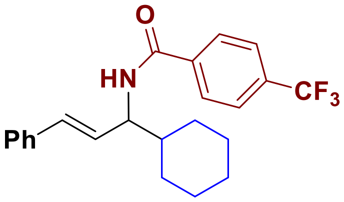

From styrene (46 µL, 0.40 mmol, 2.0 equiv), cyclohexanecarboxaldehyde (24 µL, 0.20 mmol, 1.0 equiv) and 4-(trifluoromethyl)benzamide (45.4 mg, 0.24 mmol, 1.2 equiv) the title compound was prepared following the general procedure **A** using $Ni(COD)_2$ (11 mg, 20 mol%), PCy_3 (22.4 mg, 40 mol%), $Ti(O^iPr)_4$ (60 µL, 100 mol%). anhydrous acetonitrile (0.5 mL). The reaction mixture was stirred for 12 h at 100 °C. The crude material was purified by flash column chromatography (petroleum ether/acetone = 7:1) to provide the title compound as a white solid in 62% yield (48.6 mg). m.p.: 173−175 °C
^{1}H NMR (600 MHz, $CDCl_3$) δ 7.90 (d, *J* = 7.9 Hz, 2H), 7.71 (d, *J* = 8.0 Hz, 2H), 7.37 (d, *J* = 7.6 Hz, 2H), 7.31 (t, *J* = 7.5 Hz, 2H), 7.23 (t, *J* = 7.3 Hz, 1H), 6.59 (d, *J* = 15.8

Hz, 1H), 6.18 (dt, J = 15.9, 8.1 Hz, 2H), 4.69 (q, J = 7.6 Hz, 1H), 1.88 – 1.76 (m, 4H), 1.71 – 1.63 (m, 2H), 1.26 (d, J = 12.4 Hz, 2H), 1.19 – 1.07 (m, 3H).

^{13}C NMR (151 MHz, $CDCl_3$) δ 165.5, 138.1, 136.5, 133.2 (q, J = 32.8 Hz), 132.0, 128.6, 127.9, 127.7, 127.4, 126.4, 125.6 (q, J = 4.1 Hz), 123.7 (q, J = 272.5 Hz), 56.7, 42.6, 29.6, 29.2, 26.3, 26.0.

^{19}F NMR (376 MHz, $CDCl_3$) δ -62.92.

HRMS (ESI) calcd. for $[C_{23}H_{24}F_3NO, M+H]^+$: 388.1888, found: 388.1882.

(*E*)-N-(1-cyclohexyl-3-phenylallyl)cyclohexanecarboxamide (15)

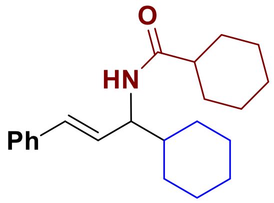

From styrene (46 μL, 0.40 mmol, 2.0 equiv), cyclohexanecarboxaldehyde (24 μL, 0.20 mmol, 1.0 equiv) and cyclohexanecarboxamide (30.5 mg, 0.24 mmol, 1.2 equiv) the title compound was prepared following the general procedure **A** using $Ni(COD)_2$ (11 mg, 20 mol%), PCy_3 (22.4 mg, 40 mol%), $Ti(O^iPr)_4$ (60 μL, 100 mol%). anhydrous acetonitrile (0.5 mL). The reaction mixture was stirred for 12 h at 100 °C. The crude material was purified by flash column chromatography (petroleum ether/acetone = 7:1) to provide the title compound as a white solid in 50% yield (32.8 mg). m.p.: 147−149 °C

^{1}H NMR (600 MHz, $CDCl_3$) δ 7.37 – 7.27 (m, 4H), 7.22 (t, J = 7.3 Hz, 1H), 6.47 (d, J = 15.8 Hz, 1H), 6.09 (dd, J = 15.8, 6.9 Hz, 1H), 5.46 (d, J = 9.0 Hz, 1H), 4.49 (q, J = 7.4 Hz, 1H), 2.12 (tt, J = 11.9, 3.6 Hz, 1H), 1.90 (dd, J = 11.2, 6.4 Hz, 2H), 1.84 – 1.75 (m, 5H), 1.67 (t, J = 12.4 Hz, 2H), 1.49 (ddp, J = 21.0, 12.4, 4.4, 3.7 Hz, 3H), 1.32 – 1.11 (m, 7H), 1.03 (dtd, J = 25.2, 13.2, 12.3, 5.9 Hz, 2H).

^{13}C NMR (151 MHz, $CDCl_3$) δ 175.2, 136.9, 131.0, 128.8, 128.5, 127.4, 126.3, 55.2, 45.9, 42.6, 30.0, 29.7, 29.5, 28.9, 26.3, 26.1, 26.1, 25.8, 25.7.

HRMS (ESI) calcd. for $[C_{22}H_{31}NO, M+Na]^+$: 348.2303, found: 348.2295.

(*E*)-N-(1-cyclohexyl-3-phenylallyl)-2-phenylacetamide (16)

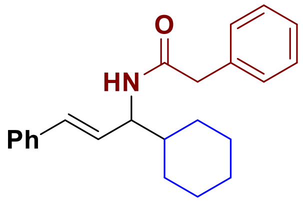

From styrene (46 μL, 0.40 mmol, 2.0 equiv), cyclohexanecarboxaldehyde (24 μL, 0.20 mmol, 1.0 equiv) and 2-phenylacetamide (32.4 mg, 0.24 mmol, 1.2 equiv) the title compound was prepared following the general procedure **A** using $Ni(COD)_2$ (11 mg, 20 mol%), PCy_3 (22.4 mg, 40 mol%), $Ti(O^iPr)_4$ (60 μL, 100 mol%). anhydrous acetonitrile (0.5 mL). The reaction mixture was stirred for 12 h at 100 °C. The crude material was purified by flash column chromatography (petroleum ether/acetone = 7:1) to provide the title compound as a white solid in 54% yield (36.1 mg). m.p.: 145−147 °C

^{1}H NMR (600 MHz, $CDCl_3$) δ 7.39 (t, *J* = 7.4 Hz, 2H), 7.34 – 7.26 (m, 7H), 7.21 (q, *J* = 4.4 Hz, 1H), 6.31 (d, *J* = 15.9 Hz, 1H), 5.99 (dd, *J* = 15.9, 6.7 Hz, 1H), 5.36 (d, *J* = 9.2 Hz, 1H), 4.47 (d, *J* = 7.7 Hz, 1H), 3.64 (s, 2H), 1.73 – 1.67 (m, 2H), 1.62 (d, *J* = 13.4 Hz, 3H), 1.46 – 1.39 (m, 1H), 1.16 (dddd, *J* = 16.1, 12.3, 7.4, 3.5 Hz, 2H), 1.08 (tt, *J* = 13.0, 3.5 Hz, 1H), 0.98 – 0.92 (m, 1H), 0.83 (tt, *J* = 12.8, 6.2 Hz, 1H).
^{13}C NMR (151 MHz, $CDCl_3$) δ 170.21 , 136.74 , 135.09 , 130.90 , 129.50 , 129.14 , 128.52 , 128.45 , 127.52 , 127.49 , 126.33 , 55.65 , 44.15 , 42.42 , 29.45 , 28.70 , 26.31 , 26.03 .
HRMS (ESI) calcd. for $[C_{23}H_{27}NO, M+Na]^+$: 356.1990, found: 356.1979.

(*E*)-N-(1,3-diphenylallyl)-4-methylbenzenesulfonamide (17)[1]

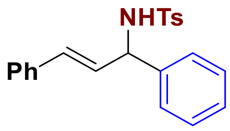

From styrene (46 μL, 0.40 mmol, 2.0 equiv), benzaldehyde (21 μL, 0.20 mmol, 1.0 equiv) and *p*-toluenesulfonamide (41.0 mg, 0.24 mmol, 1.2 equiv) the title compound was prepared following the general procedure **A** using $Ni(COD)_2$ (5.5 mg, 10 mol%), PCy_3 (11.2 mg, 20 mol%), $Ti(O^iPr)_4$ (12 μL, 20 mol%) and 4Å MS (30 mg). anhydrous toluene (0.5 mL). The reaction mixture was stirred for 4 h at 100 °C. The crude material was purified by flash column chromatography (petroleum ether/EtOAc = 10:1) to provide the title compound as a white solid in 85% yield (61.7 mg).
^{1}H NMR (400 MHz, $CDCl_3$) δ 7.69 (d, *J* = 8.0 Hz, 2H), 7.32 – 7.07 (m, 12H), 6.37 (d, *J* = 15.8 Hz, 1H), 6.10 (dd, *J* = 15.8, 6.8 Hz, 1H), 5.30 (d, *J* = 7.4 Hz, 1H), 5.14 (t, *J* = 7.1 Hz, 1H), 2.34 (s, 3H).
^{13}C NMR (101 MHz, $CDCl_3$) δ 143.2, 139.6, 137.7, 136.0, 132.0, 129.7, 129.4, 128.7, 128.4, 128.1, 127.8, 127.2, 127.0, 126.5, 59.8, 21.5.
HRMS (ESI) calcd. for $[C_{22}H_{21}NO_2S, M+Na]^+$: 386.1191, found: 386.1189.

(*E*)-N-(1-(2,4-dimethoxyphenyl)-3-phenylallyl)-4-methylbenzenesulfonamide (18)

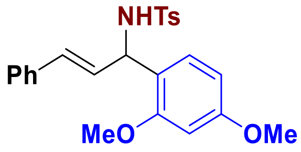

From styrene (46 μL, 0.40 mmol, 2.0 equiv), 2,4-dimethoxybenzaldehyde (33.2 mg, 0.20 mmol, 1.0 equiv) and *p*-toluenesulfonamide (41.0 mg, 0.24 mmol, 1.2 equiv) the title compound was prepared following the general procedure **A** using $Ni(COD)_2$ (5.5 mg, 10 mol%), PCy_3 (11.2 mg, 20 mol%), $Ti(O^iPr)_4$ (60 μL, 100 mol%) and 4Å MS (30 mg). anhydrous toluene (0.5 mL). The reaction mixture was stirred for 12 h at 100 °C. The crude material was purified by flash column chromatography (petroleum ether/EtOAc = 2:1) to provide the title compound as a white solid in 60% yield (50.8 mg). m.p.: 132−133 °C.
^{1}H NMR (400 MHz, $CDCl_3$) δ 7.57 (d, *J* = 8.3 Hz, 2H), 7.29 – 7.13 (m, 5H), 7.07 (d,

J = 8.0 Hz, 2H), 6.92 (d, J = 8.3 Hz, 1H), 6.36 – 6.26 (m, 2H), 6.28 – 6.15 (m, 2H), 5.59 (d, J = 9.1 Hz, 1H), 5.12 (ddd, J = 9.1, 5.9, 1.3 Hz, 1H), 3.75 (s, 3H), 3.68 (s, 3H), 2.30 (s, 3H).
^{13}C NMR (101 MHz, $CDCl_3$) δ 142.7, 137.9, 136.5, 130.7, 129.6, 129.0, 128.7, 128.3, 127.5, 127.0, 126.4, 119.8, 104.1, 98.9, 57.8, 55.4, 55.3, 21.3.
HRMS (ESI) calcd. for [$C_{24}H_{25}NO_4S$, M-H]$^-$: 422.1431, found: 422.1431.

(*E*)-N-(1-(3-chloro-4-methylphenyl)-3-phenylallyl)-4-methylbenzenesulfonamide (19)

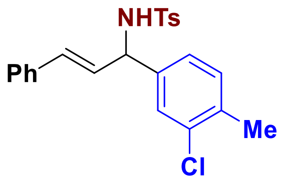

From styrene (46 μL, 0.40 mmol, 2.0 equiv), 3-chloro-4-methylbenzaldehyde (30.8 mg, 0.20 mmol, 1.0 equiv) and *p*-toluenesulfonamide (41.0 mg, 0.24 mmol, 1.2 equiv) the title compound was prepared following the general procedure **A** using Ni(COD)$_2$ (5.5 mg, 10 mol%), PCy$_3$ (11.2 mg, 20 mol%), Ti(O^{i}Pr)$_4$ (60 μL, 100 mol%) and 4Å MS (30 mg). anhydrous toluene (0.5 mL). The reaction mixture was stirred for 12 h at 100 °C. The crude material was purified by flash column chromatography (petroleum ether/EtOAc = 2:1) to provide the title compound as a white solid in 62% yield (51.0 mg). m.p.: 113−114 ℃.
^{1}H NMR (400 MHz, $CDCl_3$) δ 7.68 (d, J = 7.9 Hz, 2H), 7.31 (q, J = 5.6, 3.8 Hz, 3H), 7.27 – 7.03 (m, 8H), 6.39 (d, J = 15.8 Hz, 1H), 6.09 (dd, J = 15.8, 6.7 Hz, 1H), 5.27 (d, J = 7.4 Hz, 1H), 5.10 (t, J = 7.1 Hz, 1H), 2.37 (d, J = 10.2 Hz, 6H).
^{13}C NMR (101 MHz, $CDCl_3$) δ 143.4, 135.6, 132.4, 131.1, 129.4, 128.5, 128.0, 127.7, 127.5, 127.2, 126.5, 125.3, 59.0, 21.4, 19.6.
HRMS (ESI) calcd. for [$C_{23}H_{22}ClNO_2S$, M-H]$^-$: 410.0987, found: 410.0985.

(*E*)-4-methyl-N-(3-phenyl-1-(4-(trifluoromethyl)phenyl)allyl)benzenesulfonamide(20)[1]

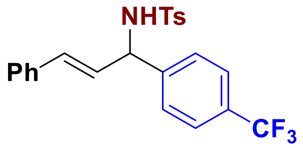

From styrene (46 μL, 0.4 mmol, 2.0 equiv), 4-(trifluoromethyl)benzaldehyde (27 μL, 0.2 mmol, 1.0 equiv) and *p*-toluenesulfonamide (41.0 mg, 0.24 mmol, 1.2 equiv) the title compound was prepared following the general procedure **A** using Ni(COD)$_2$ (5.5 mg, 10 mol%), PCy$_3$ (11.2 mg, 20 mol%), Ti(O^{i}Pr)$_4$ (12 μL, 20 mol%) and 4Å MS (30 mg). anhydrous toluene (0.5 mL). The reaction mixture was stirred for 12 h at 100 °C. The crude material was purified by flash column chromatography (petroleum

ether/EtOAc = 5:1) to provide the title compound as a white solid in 60% yield (51.8 mg).

^{1}H NMR (400 MHz, $CDCl_3$) δ 7.59 (d, *J* = 8.3, 2H), 7.43 (d, *J* = 8.3 Hz, 2H), 7.32 – 7.24 (m, 5H), 7.16 (dt, *J* = 6.3, 2.6 Hz, 2H), 7.07 (d, *J* = 8.2, 2H), 6.31 (d, *J* = 15.9, 1H), 6.06 (dd, *J* = 15.9, 6.9, 1H), 5.86 (dd, *J* = 7.9, 1H), 5.16 (t, *J* = 7.5, 1H), 2.28 (S, 3H).

^{13}C NMR (101 MHz, $CDCl_3$) δ 143.7, 143.5, 137.3, 135.7, 132.8, 129.7 (q, *J* = 24.1 Hz), 129.4, 128.5, 128.1, 127.5, 127.2, 127.1, 126.5, 125.4 (d, *J* = 3.8 Hz), 124.0 (q, *J* = 272.2 Hz), 59.4, 21.3.

^{19}F NMR (376 MHz, $CDCl_3$) δ -62.6.

HRMS (ESI) calcd. for $[C_{23}H_{20}F_3NO_2S, M+Na]^+$: 454.1065, found: 454.1060.

(*E*)-N-(1-(6-methoxypyridin-3-yl)-3-phenylallyl)-4-methylbenzenesulfonamide (21)

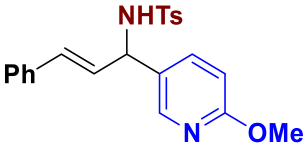

From styrene (46 μL, 0.40 mmol, 2.0 equiv), 6-methoxynicotinaldehyde (27.4 mg, 0.20 mmol, 1.0 equiv) and *p*-toluenesulfonamide (41.0 mg, 0.24 mmol, 1.2 equiv) the title compound was prepared following the general procedure **A** using $Ni(COD)_2$ (5.5 mg, 10 mol%), PCy_3 (11.2 mg, 20 mol%), $Ti(O^iPr)_4$ (60 μL, 100 mol%) and 4Å MS (30 mg). anhydrous toluene (0.5 mL). The reaction mixture was stirred for 12 h at 100 °C. The crude material was purified by flash column chromatography (petroleum ether/EtOAc = 4:1) to provide the title compound as a white solid in 34% yield (26.8 mg). m.p.: 117−118 °C.

^{1}H NMR (400 MHz, $CDCl_3$) δ 8.04 (s, 1H), 7.74 (d, *J* = 7.8 Hz, 2H), 7.52 (d, *J* = 8.7 Hz, 1H), 7.37 – 6.98 (m, 7H), 6.68 (d, *J* = 8.7 Hz, 1H), 6.41 (d, *J* = 15.8 Hz, 1H), 6.16 (dd, *J* = 16.0, 6.6 Hz, 1H), 5.60 (d, *J* = 7.3 Hz, 1H), 5.17 (t, *J* = 7.0 Hz, 1H), 3.97 (s, 3H), 2.41 (s, 3H).

^{13}C NMR (101 MHz, $CDCl_3$) δ 163.7, 145.6, 143.4, 137.5, 137.5, 135.8, 132.4, 129.5, 128.5, 128.0, 127.5, 127.2, 126.5, 110.9, 57.1, 53.5, 21.4.

HRMS (ESI) calcd. for $[C_{22}H_{24}F_3NO_2S, M-H]^-$: 393.1278, found: 393.1275.

(*E*)-4-methyl-N-(3-phenyl-1-(1-tosyl-1H-indol-3-yl)allyl)benzenesulfonamide (22)

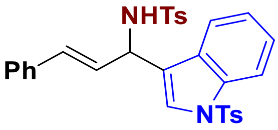

From styrene (46 μL, 0.40 mmol, 2.0 equiv), 1-[(4-methylphenyl)sulfonyl]-1H-indole-3-carboxaldehyde (59.8 mg, 0.20 mmol, 1.0 equiv) and *p*-toluenesulfonamide (41.0 mg, 0.24 mmol, 1.2 equiv) the title compound was prepared following the general procedure **A** using $Ni(COD)_2$ (5.5 mg, 10 mol%), PCy_3 (11.2 mg, 20 mol%), $Ti(O^iPr)_4$ (12 μL, 20 mol%) and 4Å MS (30 mg). anhydrous toluene (0.5 mL). The reaction mixture was

stirred for 12 h at 100 °C. The crude material was purified by flash column chromatography (petroleum ether/EtOAc = 2:1) to provide the title compound as a white solid in 68% yield (75.6 mg). m.p.: 177−178 °C.

^{1}H NMR (400 MHz, $CDCl_3$) δ 7.92 (d, J = 8.3 Hz, 1H), 7.75 (d, J = 8.1 Hz, 2H), 7.62 (d, J = 7.9 Hz, 2H), 7.54 (d, J = 7.9 Hz, 1H), 7.24 (ddt, J = 36.5, 21.5, 7.4 Hz, 10H), 7.05 (d, J = 7.9 Hz, 2H), 6.39 (d, J = 15.8 Hz, 1H), 6.13 (dd, J = 15.8, 6.7 Hz, 1H), 5.38 (t, J = 7.2 Hz, 1H), 5.10 (d, J = 7.5 Hz, 1H), 2.36 (s, 3H), 2.28 (s, 3H).

^{13}C NMR (101 MHz, $CDCl_3$) δ 145.1, 143.4, 137.4, 135.8, 135.2, 135.0, 132.8, 129.9, 129.3, 128.4, 128.3, 128.0, 127.1, 126.8, 126.6, 126.0, 124.9, 124.2, 123.3, 120.9, 120.4, 113.5, 52.7, 21.5, 21.3.

HRMS (ESI) calcd. for $[C_{31}H_{28}N_2O_4S_2, M\text{-}H]^-$: 555.1417, found: 438.1415.

(*E*)-4-methyl-N-(1-(5-methylfuran-2-yl)-3-phenylallyl)benzenesulfonamide (23)

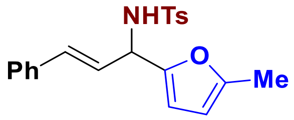

From styrene (46 μL, 0.40 mmol, 2.0 equiv), 5-methyl furfural (20 μL, 0.20 mmol, 1.0 equiv) and *p*-toluenesulfonamide (41.0 mg, 0.24 mmol, 1.2 equiv) the title compound was prepared following the general procedure **A** using $Ni(COD)_2$ (5.5 mg, 10 mol%), PCy_3 (11.2 mg, 20 mol%), $Ti(O^iPr)_4$ (12 μL, 20 mol%) and 4Å MS (30 mg). anhydrous toluene (0.5 mL). The reaction mixture was stirred for 12 h at 100 °C. The crude material was purified by flash column chromatography (petroleum ether/EtOAc = 6:1) to provide the title compound as a white solid in 56% yield (41.1 mg). m.p.: 105−106 °C.

^{1}H NMR (400 MHz, $CDCl_3$) δ 7.70 – 7.62 (m, 2H), 7.28 – 7.19 (m, 5H), 7.16 (d, J = 8.1 Hz, 2H), 6.48 – 6.31 (m, 1H), 6.09 (dd, J = 15.8, 6.3 Hz, 1H), 5.97 (d, J = 3.1 Hz, 1H), 5.80 – 5.69 (m, 1H), 5.16 (dt, J = 14.0, 7.5 Hz, 2H), 2.32 (s, 3H), 2.11 (s, 3H).

^{13}C NMR (101 MHz, $CDCl_3$) δ 152.2, 149.7, 143.1, 137.8, 136.0, 132.5, 129.3, 128.4, 127.9, 127.2, 126.6, 125.7, 108.4, 106.1, 53.7, 21.4, 13.3.

HRMS (ESI) calcd. for $[C_{21}H_{21}NO_3S, M\text{-}H]^-$: 366.1169, found: 366.1169.

(*E*)-4-methyl-N-(1-(5-methylthiophen-2-yl)-3-phenylallyl)benzenesulfonamide (24)

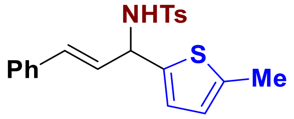

From styrene (46 μL, 0.40 mmol, 2.0 equiv), 5-methylthiophene-2-carboxaldehyde (22 μL, 0.20 mmol, 1.0 equiv) and *p*-toluenesulfonamide (41.0 mg, 0.24 mmol, 1.2 equiv) the title compound was prepared following the general procedure **A** using $Ni(COD)_2$ (5.5 mg, 10 mol%), PCy_3 (11.2 mg, 20 mol%), $Ti(O^iPr)_4$ (60 μL, 100 mol%) and 4Å MS (30 mg). anhydrous toluene (0.5 mL). The reaction mixture was stirred for 12 h at 100 °C. The crude material was purified by flash column chromatography (petroleum ether/EtOAc = 6:1) to provide the title compound as a white solid in 71% yield (54.4 mg). m.p.: 122−124 °C.

^{1}H NMR (400 MHz, $CDCl_3$) δ 7.73 – 7.60 (m, 2H), 7.29 – 7.21 (m, 3H), 7.20 – 7.12 (m, 3H), 6.63 (dd, *J* = 3.4, 0.9 Hz, 1H), 6.52 (dd, *J* = 3.4, 1.3 Hz, 1H), 6.43 (dd, *J* = 15.8, 1.2 Hz, 1H), 6.05 (dd, *J* = 15.8, 6.9 Hz, 1H), 5.27 (t, *J* = 7.2 Hz, 1H), 4.93 (d, *J* = 7.5 Hz, 1H), 2.39 (d, *J* = 1.1 Hz, 3H), 2.33 (s, 3H).
^{13}C NMR (101 MHz, $CDCl_3$) δ 143.3, 140.9, 140.4, 137.7, 135.9, 132.3, 129.5, 128.4, 128.0, 127.4, 127.3, 126.6, 125.3, 124.9, 55.6, 21.4, 15.3.
HRMS (ESI) calcd. for $[C_{21}H_{21}NO_2S_2, M-H]^-$: 382.0941, found: 382.0938.

(*E*)-N-(1-(4,4-difluorocyclohexyl)-3-phenylallyl)-4-methylbenzenesulfonamide (25)

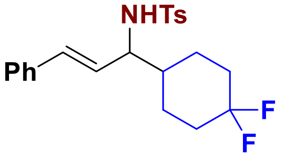

From styrene (46 μL, 0.40 mmol, 2.0 equiv), 4,4-difluorocyclohexane-1-carbaldehyde (29.6 mg, 0.20 mmol, 1.0 equiv) and *p*-toluenesulfonamide (41.0 mg, 0.24 mmol, 1.2 equiv) the title compound was prepared following the general procedure **A** using $Ni(COD)_2$ (5.5 mg, 10 mol%), PCy_3 (11.2 mg, 20 mol%), $Ti(O^iPr)_4$ (12 μL, 20 mol%), anhydrous acetonitrile (0.5 mL). The reaction mixture was stirred for 12 h at 100 °C. The crude material was purified by flash column chromatography (petroleum ether/EtOAc = 5:1) to provide the title compound as a white solid in 63% yield (51.0 mg). m.p.: 187−188 °C.
^{1}H NMR (400 MHz, $CDCl_3$) δ 7.64 (d, *J* = 8.0 Hz, 2H), 7.20 – 7.12 (m, 3H), 7.09 (d, *J* = 8.0 Hz, 2H), 7.01 – 6.91 (m, 2H), 5.94 (d, *J* = 15.8 Hz, 1H), 5.59 (dd, *J* = 15.8, 8.3 Hz, 1H), 4.89 (d, *J* = 8.7 Hz, 1H), 3.67 (q, *J* = 8.0 Hz, 1H), 2.18 (s, 3H), 2.06 – 1.91 (m, 2H), 1.86 (d, *J* = 13.6 Hz, 1H), 1.72 – 1.59 (m, 2H), 1.48 (d, *J* = 7.8 Hz, 1H), 1.29 (qd, *J* = 11.1, 7.4, 4.9 Hz, 2H).
^{13}C NMR (101 MHz, $CDCl_3$) δ 142.4, 136.7, 134.8, 131.9, 128.5, 127.3, 126.8, 126.2, 125.2, 124.9, 122.7 (q, *J* = 243.4 Hz), 59.3, 39.9, 32.0 (t, *J* = 24.5 Hz), 24.1 (dd, *J* = 18.7, 9.6 Hz), 20.3.
^{19}F NMR (376 MHz, $CDCl_3$) δ -92.2(d, J = 236.5 Hz), -101.9(d, J = 236.1 Hz).
HRMS (ESI) calcd. for $[C_{22}H_{25}F_2NO_2S, M-H]^-$: 404.1501, found: 404.1496.

(*E*)-4-methyl-N-(3-phenyl-1-(tetrahydro-2H-pyran-4-yl)allyl)benzenesulfonamide (26)[2]

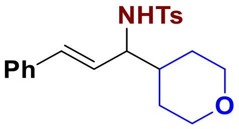

From styrene (46 μL, 0.40 mmol, 2.0 equiv), tetrahydropyran-4-carbaldehyde (21 μL, 0.20 mmol, 1.0 equiv) and *p*-toluenesulfonamide (41.0 mg, 0.24 mmol, 1.2 equiv) the title compound was prepared following the general procedure **A** using $Ni(COD)_2$ (5.5 mg, 10 mol%), PCy_3 (11.2 mg, 20 mol%), $Ti(O^iPr)_4$ (12 μL, 20 mol%), anhydrous

acetonitrile (0.5 mL). The reaction mixture was stirred for 12 h at 100 °C. The crude material was purified by flash column chromatography (petroleum ether/EtOAc = 4:1) to provide the title compound as a white solid in 72% yield (53.4 mg).

^{1}H NMR (400 MHz, $CDCl_3$) δ 7.74 (d, *J* = 7.9 Hz, 2H), 7.32 – 7.14 (m, 5H), 7.08 (d, *J* = 7.2 Hz, 2H), 6.11 – 6.02 (m, 1H), 5.75 – 5.63 (m, 1H), 5.15 (d, *J* = 8.8 Hz, 1H), 3.96 (s, 2H), 3.71 (q, *J* = 8.8 Hz, 1H), 3.32 (q, *J* = 11.6 Hz, 2H), 2.27 (d, *J* = 2.9 Hz, 3H), 1.77 (d, *J* = 13.6 Hz, 1H), 1.54 (d, *J* = 13.5 Hz, 1H), 1.46 – 1.25 (m, 3H).

^{13}C NMR (101 MHz, $CDCl_3$) δ 136.0, 132.9, 129.5, 128.4, 127.8, 127.3, 126.3, 126.2, 67.6, 67.5, 61.1, 40.2, 29.3, 29.2, 21.3.

HRMS (ESI) calcd. for $[C_{21}H_{25}NO_3S, M+Na]^+$: 394.1453, found: 394.1450.

tert-butyl-(_E_)-4-(1-((4-methylphenyl)sulfonamido)-3-phenylallyl)piperidine-1-carboxylate (27)

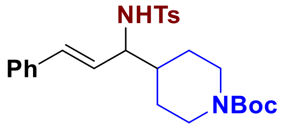

From styrene (46 µL, 0.40 mmol, 2.0 equiv), 1-*tert*-butoxycarbonyl-4-piperidinecarboxaldehyde (42.6 mg, 0.20 mmol, 1.0 equiv) and *p*-toluenesulfonamide (41.0 mg, 0.24 mmol, 1.2 equiv) the title compound was prepared following the general procedure **A** using $Ni(COD)_2$ (5.5 mg, 10 mol%), PCy_3 (11.2 mg, 20 mol%), $Ti(O^iPr)_4$ (12 µL, 20 mol%), anhydrous acetonitrile (0.5 mL). The reaction mixture was stirred for 12 h at 100 °C. The crude material was purified by flash column chromatography (petroleum ether/EtOAc = 4:1) to provide the title compound as a white solid in 77% yield (72.4 mg). m.p.: 130−131 °C.

^{1}H NMR (400 MHz, $CDCl_3$) δ 7.71 (d, *J* = 7.9 Hz, 2H), 7.32 – 6.81 (m, 7H), 5.98 (d, *J* = 15.8 Hz, 1H), 5.66 (dd, *J* = 15.9, 8.4 Hz, 1H), 5.55 (d, *J* = 8.8 Hz, 1H), 4.07 (s, 2H), 3.76 – 3.55 (m, 1H), 2.61 (d, *J* = 21.4 Hz, 2H), 2.22 (s, 3H), 1.82 (d, *J* = 12.9 Hz, 1H), 1.59 (d, *J* = 11.4 Hz, 2H), 1.43 (s, 9H), 1.15 (td, *J* = 12.2, 4.2 Hz, 1H).

^{13}C NMR (101 MHz, $CDCl_3$) δ 154.6, 143.2, 137.9, 136.0, 132.7, 129.4, 128.3, 127.7, 127.2, 126.2, 126.2, 79.4, 61.0, 43.7, 41.2, 29.2, 28.4, 21.3.

HRMS (ESI) calcd. for $[C_{26}H_{34}N_2O_4S, M-H]^-$: 469.2166, found: 469.2160.

tert-butyl-(2S)-2-((_E_)-1-((4-methylphenyl)sulfonamido)-3-phenylallyl)pyrrolidine-1-carboxylate (28)

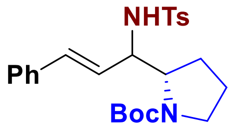

From styrene (46 µL, 0.40 mmol, 2.0 equiv), N-Boc-*L*-prolinal (39.8 mg, 0.20 mmol, 1.0 equiv) and *p*-toluenesulfonamide (41.0 mg, 0.24 mmol, 1.2 equiv) the title compound was prepared following the general procedure **A** using $Ni(COD)_2$ (5.5 mg, 10 mol%), PCy_3 (11.2 mg, 20 mol%), $Ti(O^iPr)_4$ (60 µL, 100 mol%), anhydrous acetonitrile (0.5 mL). The reaction mixture was stirred for 12 h at 100 °C. The crude

material was purified by flash column chromatography (petroleum ether/EtOAc = 3:1) to provide the title compound as a yellow sticky oil in 55% yield with a mixture containing diastereoisomers. (2.3:1 dr, 50.1 mg).

^{1}H NMR (400 MHz, $CDCl_3$) δ 7.67 (d, J = 7.6 Hz, 2H), 7.49 (d, J = 7.5 Hz, 1H), 7.31 – 6.96 (m, 8H), 6.49 – 6.20 (m, 1H), 5.60 (dd, J = 15.8, 8.2 Hz, 1H), 4.24 – 3.68 (m, 2H), 3.42 (d, J = 8.9 Hz, 1H), 3.23 – 2.92 (m, 1H), 2.29 (d, J = 17.0 Hz, 3H), 2.11 – 1.89 (m, 1H), 1.81 – 1.58 (m, 3H), 1.48 (d, J = 16.9 Hz, 10H).

^{13}C NMR (101 MHz, $CDCl_3$) δ 156.4, 142.5, 139.1, 136.4, 136.2, 133.4, 129.2, 128.3, 128.3, 127.7, 127.6, 127.2, 127.1, 126.4, 126.4, 124.6, 61.5, 61.3, 60.7, 48.0, 29.5, 28.4, 28.3, 28.1, 23.8, 23.4, 21.3.

HRMS (ESI) calcd. for $[C_{25}H_{32}N_2O_4S, M-H]^-$: 455.2010, found: 455.2008.

(*E*)-N-(1-cyclopentyl-3-phenylallyl)-4-methylbenzenesulfonamide (29)

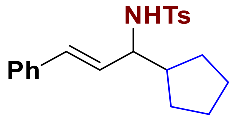

From styrene (46 µL, 0.40 mmol, 2.0 equiv), cyclopentanecarbaldehyde (21 µL, 0.20 mmol, 1.0 equiv) and *p*-toluenesulfonamide (41.0 mg, 0.24 mmol, 1.2 equiv) the title compound was prepared following the general procedure **A** using $Ni(COD)_2$ (5.5 mg, 10 mol%), PCy_3 (11.2 mg, 20 mol%), $Ti(O^iPr)_4$ (12 µL, 20 mol%), anhydrous acetonitrile (0.5 mL). The reaction mixture was stirred for 12 h at 100 °C. The crude material was purified by flash column chromatography (petroleum ether/EtOAc = 5:1) to provide the title compound as a white solid in 70% yield (49.7 mg). m.p.: 119−120 °C.

^{1}H NMR (400 MHz, $CDCl_3$) δ 7.76 – 7.69 (m, 2H), 7.31 – 7.14 (m, 5H), 7.12 – 7.05 (m, 2H), 6.19 (d, J = 15.9 Hz, 1H), 5.62 (dd, J = 15.9, 7.6 Hz, 1H), 4.71 – 4.42 (m, 1H), 3.81 (q, J = 8.0 Hz, 1H), 2.45 – 2.30 (m, 1H), 2.29 (s, 3H), 2.06 – 1.66 (m, 5H).

^{13}C NMR (101 MHz, $CDCl_3$) δ 143.2, 138.1, 136.3, 131.8, 129.4, 128.3, 127.6, 127.3, 126.9, 126.3, 61.1, 39.4, 25.0, 24.7, 21.4, 17.4.

HRMS (ESI) calcd. for $[C_{21}H_{25}NO_2S, M-H]^-$: 354.1533, found: 354.1530.

(*E*)-4-methyl-N-(3-phenyl-1-(tetrahydrofuran-2-yl)allyl)benzenesulfonamide (30)

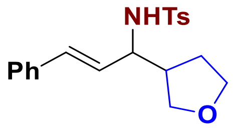

From styrene (46 µL, 0.40 mmol, 2.0 equiv), tetrahydrofuran-2-carbaldehyde (20 mg, 0.20 mmol, 1.0 equiv) and *p*-toluenesulfonamide (41.0 mg, 0.24 mmol, 1.2 equiv) the title compound was prepared following the general procedure **A** using $Ni(COD)_2$ (5.5 mg, 10 mol%), PCy_3 (11.2 mg, 20 mol%), $Ti(O^iPr)_4$ (60 µL, 100 mol%), anhydrous acetonitrile (0.5 mL). The reaction mixture was stirred for 12 h at 100 °C. The crude material was purified by flash column chromatography (petroleum ether/EtOAc = 3:1)

to provide the title compound as a white solid in 65% yield with a mixture containing diastereoisomers. (1:1 dr, 46.4 mg).

^{1}H NMR (400 MHz, $CDCl_3$) δ 7.79 (d, *J* = 8.0 Hz, 2H), 7.35 – 7.27 (m, 3H), 7.23 (d, *J* = 8.0 Hz, 2H), 7.13 (dt, *J* = 7.6, 2.1 Hz, 2H), 6.19 (dd, *J* = 15.8, 12.0 Hz, 1H), 5.74 (ddd, *J* = 20.2, 15.8, 8.0 Hz, 1H), 5.16 (dd, *J* = 31.0, 8.3 Hz, 1H), 3.99 – 3.58 (m, 5H), 2.47 (s, 1H), 2.33 (s, 3H), 2.17 – 1.73 (m, 2H).

^{13}C NMR (101 MHz, $CDCl_3$) δ 143.4, 143.3, 137.9, 137.8, 136.0, 135.9, 132.6, 132.3, 129.5, 128.3, 127.8, 127.8, 127.3, 127.2, 126.9, 126.7, 126.3, 126.3, 70.5, 70.0, 68.1, 68.0, 59.4, 59.2, 44.2, 44.0, 29.2, 29.1, 21.3.

HRMS (ESI) calcd. for $[C_{20}H_{23}NO_3S, M-H]^-$: 356.1326, found: 356.1324.

(*E*)-N-(1-cyclobutyl-3-phenylallyl)-4-methylbenzenesulfonamide (31)

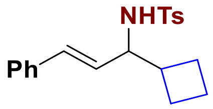

From styrene (46 µL, 0.40 mmol, 2.0 equiv), cyclobutanecarboxaldehyde (18 µL, 0.20 mmol, 1.0 equiv) and *p*-toluenesulfonamide (41.0 mg, 0.24 mmol, 1.2 equiv) the title compound was prepared following the general procedure **B** using $Ni(OAc)_2$ (3.5 mg, 10 mol%), PCy_3 (11.2 mg, 20 mol%), $Ti(O^iPr)_4$ (60 µL, 100 mol%), Mn (2.2 mg, 20 mol%), anhydrous acetonitrile (0.5 mL). The reaction mixture was stirred for 12 h at 100 °C. The crude material was purified by flash column chromatography (petroleum ether/EtOAc = 4:1) to provide the title compound as a white solid in 47% yield (32.1 mg). m.p.: 113−114 °C.

^{1}H NMR (400 MHz, $CDCl_3$) δ 7.78 – 7.67 (m, 2H), 7.30 – 7.14 (m, 5H), 7.12 – 7.01 (m, 2H), 6.19 (d, *J* = 15.9 Hz, 1H), 5.65 – 5.51 (m, 1H), 4.67 – 4.49 (m, 1H), 3.81 (q, *J* = 8.0 Hz, 1H), 2.43 – 2.33 (m, 1H), 2.29 (s, 3H), 2.04 – 1.88 (m, 2H), 1.87 – 1.68 (m, 4H), 1.62 – 1.55 (m, 1H)..

^{13}C NMR (101 MHz, $CDCl_3$) δ 143.2, 138.1, 136.3, 131.8, 129.4, 128.3, 127.6, 127.3, 126.9, 126.3, 61.1, 39.4, 25.0, 24.7, 21.4, 17.4.

HRMS (ESI) calcd. for $[C_{20}H_{23}NO_2S, M-H]^-$: 340.1376, found: 340.1371.

tert-butyl-(*E*)-3-(1-((4-methylphenyl)sulfonamido)-3-phenylallyl)azetidine-1-carboxylate (32)

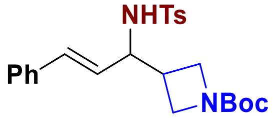

From styrene (46 µL, 0.40 mmol, 2.0 equiv), *tert*-butyl-3-formylazetidine-1-carboxylate (37 mg, 0.20 mmol, 1.0 equiv) and *p*-toluenesulfonamide (41.0 mg, 0.24 mmol, 1.2 equiv) the title compound was prepared following the general procedure **B** using $Ni(OAc)_2$ (3.5 mg, 10 mol%), PCy_3 (11.2 mg, 20 mol%), $Ti(O^iPr)_4$ (60 µL, 100 mol%), Mn (2.2 mg, 20 mol%)anhydrous acetonitrile (0.5 mL). The reaction mixture was stirred for 12 h at 100 °C. The crude material was purified by flash column

chromatography (petroleum ether/EtOAc = 4:1) to provide the title compound as a white solid in 36% yield (31.8 mg). m.p.: 137−138 °C.

^{1}H NMR (400 MHz, $CDCl_3$) δ 7.75 (d, *J* = 8.1 Hz, 2H), 7.28 – 7.22 (m, 3H), 7.19 (d, *J* = 8.0 Hz, 2H), 7.09 – 7.02 (m, 2H), 6.16 (d, *J* = 15.8 Hz, 1H), 5.61 (dd, *J* = 16.3, 7.7 Hz, 1H), 5.32 (s, 1H), 4.04 (q, *J* = 8.5 Hz, 1H), 3.97 (t, *J* = 8.6 Hz, 1H), 3.89 (t, *J* = 8.7 Hz, 1H), 3.79 (dd, *J* = 9.1, 5.3 Hz, 1H), 3.65 (s, 1H), 2.64 (d, *J* = 8.9 Hz, 1H), 2.28 (s, 3H), 1.44 (s, 9H).

^{13}C NMR (101 MHz, $CDCl_3$) δ 156.2, 143.5, 137.79, 135.7, 133.2, 129.6, 128.4, 128.0, 127.3, 126.4, 124.8, 79.5, 59.0, 51.1, 32.9, 28.3, 21.3.

HRMS (ESI) calcd. for [$C_{24}H_{30}N_2O_4S$, M-H]$^-$: 441.1853, found: 441.1851.

(*E*)-N-(7-hydroxy-1-phenylhept-1-en-3-yl)-4-methylbenzenesulfonamide (33)

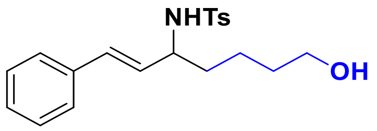

From styrene (46 μL, 0.40 mmol, 2.0 equiv), 2-hydroxytetrahydropyran (19 μL, 0.20 mmol, 1.0 equiv) and *p*-toluenesulfonamide (41.0 mg, 0.24 mmol, 1.2 equiv) the title compound was prepared following the general procedure **A** using $Ni(COD)_2$ (5.5 mg, 10 mol%), PCy_3 (11.2 mg, 20 mol%), $Ti(O^iPr)_4$ (60 μL, 100 mol%), anhydrous EtOH (0.5 mL). The reaction mixture was stirred for 12 h at 100 °C. The crude material was purified by flash column chromatography (petroleum ether/EtOAc = 2:1) to provide the title compound as colorless oil in 63% yield (45.2 mg)

^{1}H NMR (400 MHz, $CDCl_3$) δ 7.73 (d, *J* = 8.3 Hz, 2H), 7.26 – 7.14 (m, 5H), 7.09 – 7.02 (m, 2H), 6.15 (d, *J* = 15.9 Hz, 1H), 5.70 (dd, *J* = 15.9, 7.6 Hz, 1H), 5.22 (d, *J* = 8.0 Hz, 1H), 3.96 – 3.85 (m, 1H), 3.58 (t, *J* = 6.3 Hz, 2H), 2.27 (s, 3H), 1.68 – 1.50 (m, 4H), 1.40 (ddt, *J* = 15.6, 13.4, 6.0 Hz, 2H).

^{13}C NMR (101 MHz, $CDCl_3$) δ 143.2, 138.1, 136.2, 131.3, 129.5, 128.7, 128.3, 127.6, 127.3, 126.3, 62.4, 56.3, 35.6, 32.1, 21.7, 21.3.

HRMS (ESI) calcd. for [$C_{20}H_{25}NO_3S$, M-H]$^-$: 358.1482, found: 358.1480.

(*E*)-N-(4,4-dimethyl-1-phenylpent-1-en-3-yl)-4-methylbenzenesulfonamide (34)[1]

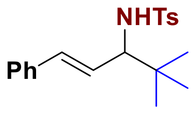

From styrene (46 μL, 0.40 mmol, 2.0 equiv), pivaldehyde (22 μL, 0.20 mmol, 1.0 equiv) and *p*-toluenesulfonamide (41.0 mg, 0.24 mmol, 1.2 equiv) the title compound was prepared following the general procedure **A** using $Ni(COD)_2$ (5.5 mg, 10 mol%), PCy_3 (11.2 mg, 20 mol%), $Ti(O^iPr)_4$ (60 μL, 100 mol%), anhydrous acetonitrile (0.5 mL). The reaction mixture was stirred for 12 h at 100 °C. The crude material was purified by flash column chromatography (petroleum ether/EtOAc = 6:1) to provide the title compound as a white solid in 53% yield (36.4 mg).

^{1}H NMR (400 MHz, $CDCl_3$) δ 7.68 (d, *J* = 7.9 Hz, 2H), 7.22 (d, *J* = 7.2 Hz, 3H), 7.12 (d, *J* = 7.9 Hz, 2H), 7.03 – 6.96 (m, 2H), 5.95 (d, *J* = 15.8 Hz, 1H), 5.70 (d, *J* = 8.5 Hz, 1H), 4.57 (d, *J* = 9.3 Hz, 1H), 3.58 (t, *J* = 8.8 Hz, 1H), 2.21 (s, 3H), 0.93 (s, 9H).
^{13}C NMR (101 MHz, $CDCl_3$) δ 143.1, 138.0, 136.3, 132.8, 129.3, 128.2, 127.5, 127.3, 126.2, 125.5, 65.5, 34.9, 26.4, 21.2.
HRMS (ESI) calcd. for [$C_{20}H_{25}NO_2S$, M+Na]$^+$: 366.1504, found: 366.1502.

Isopropyl-(*E*)-3-(1-((4-methylphenyl)sulfonamido)-3-phenylallyl)bicyclo[1.1.1]pentane-1-carboxylate (35)

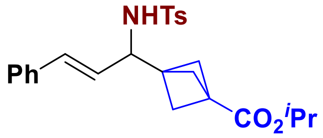

From styrene (46 μL, 0.40 mmol, 2.0 equiv), methyl-3-formylbicyclo[1.1.1]pentane-1-carboxylate (30.8 mg, 0.20 mmol, 1.0 equiv) and *p*-toluenesulfonamide (41 mg, 0.24 mmol, 1.2 equiv) the title compound was prepared following the general procedure **A** using Ni(COD)$_2$ (11 mg, 20 mol%), PCy$_3$ (22.4 mg, 40 mol%), Ti(O^{i}Pr)$_4$ (60 μL, 100 mol%), anhydrous ^{i}PrOH (0.5 mL). The reaction mixture was stirred for 12 h at 100 °C. The crude material was purified by flash column chromatography (petroleum ether/EtOAc = 5:1) to provide the title compound as colorless oil in 32% yield (28.2 mg).
^{1}H NMR (400 MHz, $CDCl_3$) δ 7.73 (d, *J* = 6.6 Hz, 2H), 7.27 – 7.18 (m, 5H), 7.13 (d, *J* = 7.2 Hz, 2H), 6.26 (d, *J* = 15.8 Hz, 1H), 5.68 (dd, *J* = 15.8, 7.4 Hz, 1H), 4.95 (dt, *J* = 10.5, 6.2 Hz, 1H), 4.82 (d, *J* = 7.7 Hz, 1H), 4.01 (t, *J* = 7.5 Hz, 1H), 2.31 (s, 3H), 1.88 (q, *J* = 9.4 Hz, 6H), 1.20 (d, *J* = 4.3 Hz, 6H).
^{13}C NMR (101 MHz, $CDCl_3$) δ 169.2, 143.4, 137.8, 136.0, 132.4, 129.5, 128.4, 127.8, 127.2, 126.4, 125.2, 67.9, 56.1, 49.5, 41.0, 38.4, 21.7, 21.4.
HRMS (ESI) calcd. for [$C_{15}H_{21}N$, M-NH$_2$]$^-$: 438.1744, found: 438.1740.

(*E*)-N-cinnamyl-4-methylbenzenesulfonamide (36)

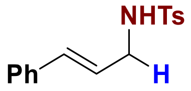

From styrene (46 μL, 0.40 mmol, 2.0 equiv), paraformaldehyde (6.0 mg, 0.20 mmol, 1.0 equiv) and *p*-toluenesulfonamide (41.0 mg, 0.24 mmol, 1.2 equiv) the title compound was prepared following the general procedure **A** using Ni(COD)$_2$ (5.5 mg, 10 mol%), PCy$_3$ (11.2 mg, 20 mol%), anhydrous EtOH (0.5 mL). The reaction mixture was stirred for 12 h at 100 °C. The crude material was purified by flash column chromatography (petroleum ether/EtOAc = 5:1) to provide the title compound as a white solid in 70% yield (40.2 mg). m.p.: 100−101 °C.
^{1}H NMR (400 MHz, $CDCl_3$) δ 7.81 – 7.74 (m, 2H), 7.33 – 7.18 (m, 7H), 6.47 – 6.36 (m, 1H), 6.00 (dt, *J* = 15.8, 6.4 Hz, 1H), 4.75 (t, *J* = 6.3 Hz, 1H), 3.74 (td, *J* = 6.3, 1.5 Hz, 2H), 2.40 (s, 3H).

^{13}**C NMR** (101 MHz, $CDCl_3$) δ 143.5, 137.0, 136.1, 133.0, 129.7, 128.5, 127.9, 127.2, 126.4, 124.1, 45.5, 21.5.
HRMS (ESI) calcd. for $[C_{16}H_{17}NO_2S, M-H]^-$: 286.0907, found: 286.0905.

***tert*-butyl-(*E*)-4-(1-((4-methylphenyl)sulfonamido)-3-(3,5-trimethoxyphenyl)allyl)piperidine-1-carboxylate (37)**

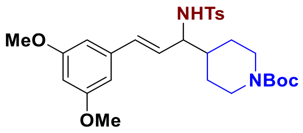

From 1,3-dimethoxy-5-vinylbenzene (65.6 mg, 0.40 mmol, 2.0 equiv), *tert*-butyl-4-formylpiperidine-1-carboxylate (42.6 mg, 0.20 mmol, 1.0 equiv) and *p*-toluenesulfonamide (41.0 mg, 0.24 mmol, 1.2 equiv) the title compound was prepared following the general procedure **A** using $Ni(COD)_2$ (5.5 mg, 10 mol%), PCy_3 (11.2 mg, 20 mol%), $Ti(O^iPr)_4$ (60 µL, 100 mol%), anhydrous acetonitrile (0.5 mL). The reaction mixture was stirred for 12 h at 100 °C. The crude material was purified by flash column chromatography (petroleum ether/acetone = 2:1) to provide the title compound as a white solid in 65% yield (68.9 mg). m.p.: 188−189 °C.
^{1}**H NMR** (400 MHz, $CDCl_3$) δ 7.74 (d, *J* = 7.9 Hz, 2H), 7.23 (d, *J* = 7.9 Hz, 2H), 6.37 (d, *J* = 2.1 Hz, 1H), 6.25 (d, *J* = 1.9 Hz, 2H), 6.02 (d, *J* = 15.8 Hz, 1H), 5.69 (dd, *J* = 15.8, 8.1 Hz, 1H), 4.74 (d, *J* = 8.5 Hz, 1H), 4.15 (s, 2H), 3.81 (d, *J* = 1.5 Hz, 7H), 2.66 (d, *J* = 16.0 Hz, 2H), 2.35 (s, 3H), 1.88 – 1.76 (m, 1H), 1.63 (d, *J* = 13.3 Hz, 2H), 1.48 (s, 9H), 1.21 (d, *J* = 12.5 Hz, 2H).
^{13}**C NMR** (101 MHz, $CDCl_3$) δ 160.7, 154.6, 143.3, 138.0, 137.9, 132.8, 129.5, 127.2, 126.7, 104.4, 99.8, 79.4, 60.8, 55.3, 43.5, 41.2, 29.2, 28.4, 21.3.
HRMS (ESI) calcd. for $[C_{28}H_{38}N_2O_6S, M-H]^-$: 529.2378, found: 529.2370.

***tert*-butyl-(*E*)-4-(3-(3,5-dichlorophenyl)-1-((4-methylphenyl)sulfonamido)allyl)piperidine-1-carboxylate (38)**

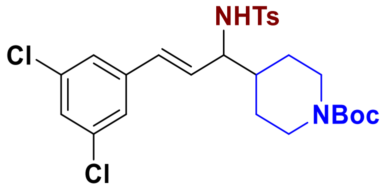

From 1,3-dichloro-5-vinylbenzene (68.4 mg, 0.40 mmol, 2.0 equiv), *tert*-butyl-4-formylpiperidine-1-carboxylate (42.6 mg, 0.20 mmol, 1.0 equiv) and *p*-toluenesulfonamide (41.0 mg, 0.24 mmol, 1.2 equiv) the title compound was prepared following the general procedure **A** using $Ni(COD)_2$ (5.5 mg, 10 mol%), PCy_3 (11.2 mg, 20 mol%), $Ti(O^iPr)_4$ (60 µL, 100 mol%), anhydrous acetonitrile (0.5 mL). The reaction mixture was stirred for 12 h at 100 °C. The crude material was purified by flash column chromatography (petroleum ether/acetone = 3:1) to provide the title compound as a white solid in 63% yield (67.8 mg). m.p.: 166−167 °C.

^{1}H NMR (400 MHz, $CDCl_3$) δ 7.70 (d, *J* = 7.9 Hz, 2H), 7.20 (d, *J* = 8.4 Hz, 3H), 6.86 (d, *J* = 1.9 Hz, 2H), 5.91 (d, *J* = 15.9 Hz, 1H), 5.68 (dd, *J* = 15.8, 8.1 Hz, 1H), 5.11 (d, *J* = 8.7 Hz, 1H), 4.11 (d, *J* = 14.6 Hz, 2H), 3.77 – 3.60 (m, 1H), 2.60 (d, *J* = 12.8 Hz, 2H), 2.33 (s, 3H), 1.79 (d, *J* = 13.0 Hz, 1H), 1.56 (d, *J* = 12.8 Hz, 2H), 1.44 (s, 9H), 1.16 (dd, *J* = 12.3, 4.2 Hz, 2H).
^{13}C NMR (101 MHz, $CDCl_3$) δ 143.6, 139.0, 137.9, 134.9, 130.3, 129.6, 129.5, 127.5, 127.3, 124.6, 79.5, 43.6, 41.1, 28.4, 28.3, 21.4.
HRMS (ESI) calcd. for $[C_{26}H_{32}Cl_2N_2O_4S, M\text{-}H]^-$: 537.1387, found: 537.1385.

***tert*-butyl-(*E*)-4-(3-(4-fluorophenyl)-1-((4-methylphenyl)sulfonamido)allyl)piperidine-1-carboxylate (39)**

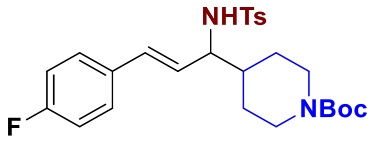

From 1-fluoro-4-vinylbenzene (36 μL, 0.30 mmol, 1.5 equiv), *tert*-butyl-4-formylpiperidine-1-carboxylate (42.6 mg, 0.20 mmol, 1.0 equiv) and *p*-toluenesulfonamide (41.0 mg, 0.24 mmol, 1.2 equiv) the title compound was prepared following the general procedure **A** using $Ni(COD)_2$ (5.5 mg, 10 mol%), PCy_3 (11.2 mg, 20 mol%), $Ti(O^iPr)_4$ (12 μL, 20 mol%), anhydrous acetonitrile (0.5 mL). The reaction mixture was stirred for 12 h at 100 °C. The crude material was purified by flash column chromatography (petroleum ether/acetone = 3:1) to provide the title compound as a white solid in 82% yield (80.0 mg). m.p.: 143−144 °C.
^{1}H NMR (400 MHz, $CDCl_3$) δ 7.72 (d, *J* = 8.0 Hz, 2H), 7.17 (d, *J* = 8.2 Hz, 2H), 7.10 – 6.99 (m, 2H), 6.99 – 6.90 (m, 2H), 6.01 (d, *J* = 15.4 Hz, 1H), 5.60 (dd, *J* = 15.8, 8.2 Hz, 1H), 4.11 (s, 2H), 3.71 (s, 1H), 2.62 (s, 2H), 2.30 (d, *J* = 11.4 Hz, 3H), 1.81 (d, *J* = 13.0 Hz, 1H), 1.63 – 1.56 (m, 2H), 1.45 (d, *J* = 2.0 Hz, 9H), 1.24 – 1.11 (m, 2H).
^{13}C NMR (101 MHz, $CDCl_3$) δ 162.2 (d, *J* = 247.3 Hz), 154.6, 143.2, 138.0, 132.2 (d, *J* = 3.6 Hz), 131.5, 129.4, 127.8 (d, *J* = 7.7 Hz), 127.2, 126.0, 115.2 (d, *J* = 21.7 Hz), 79.4, 60.9, 43.7, 41.1, 28.4, 28.2, 21.3.
^{19}F NMR (376 MHz, $CDCl_3$) δ -113.9.
HRMS (ESI) calcd. for $[C_{26}H_{33}FN_2O_4S, M\text{-}H]^-$: 487.2072, found: 487.2070.

***tert*-butyl-(*E*)-4-(1-((4-methylphenyl)sulfonamido)-3-(4-(4,4,5,5-tetramethyl-1,3,2-dioxaborolan-2-yl)phenyl)allyl)piperidine-1-carboxylate (40)**

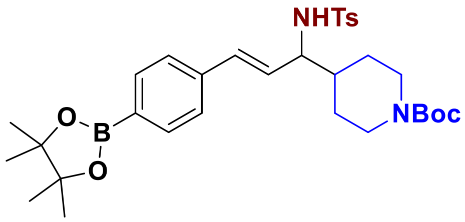

From 4,4,5,5-tetramethyl-2-(4-vinylphenyl)-1,3,2-dioxaborolane (69.0 mg, 0.30 mmol, 1.5 equiv), *tert*-butyl-4-formylpiperidine-1-carboxylate (42.6 mg, 0.20 mmol, 1.0 equiv) and *p*-toluenesulfonamide (41.0 mg, 0.24 mmol, 1.2 equiv) the title compound was prepared following the general procedure **A** using Ni(COD)$_2$ (5.5 mg, 10 mol%), PCy$_3$ (11.2 mg, 20 mol%), Ti(O^{i}Pr)$_4$ (12 μL, 20 mol%), anhydrous acetonitrile (0.5 mL). The reaction mixture was stirred for 12 h at 100 °C. The crude material was purified by flash column chromatography (petroleum ether/acetone = 5:1) to provide the title compound as a white solid in 38% yield (45.9 mg). m.p.: 184−186 °C.
^{1}H NMR (400 MHz, CDCl$_3$) δ 7.68 (dd, *J* = 10.9, 7.9 Hz, 4H), 7.13 (d, *J* = 7.9 Hz, 2H), 7.02 (d, *J* = 7.6 Hz, 2H), 5.98 (d, *J* = 15.8 Hz, 1H), 5.73 (dd, *J* = 15.8, 8.2 Hz, 1H), 5.23 (d, *J* = 9.1 Hz, 1H), 4.22 – 3.96 (m, 2H), 3.70 (s, 1H), 2.60 (s, 2H), 2.24 (s, 3H), 1.81 (d, *J* = 13.0 Hz, 1H), 1.59 (d, *J* = 11.9 Hz, 2H), 1.43 (s, 9H), 1.34 (s, 12H), 1.19 – 1.09 (m, 1H).
^{13}C NMR (101 MHz, CDCl$_3$) δ 154.6, 143.4, 138.6, 137.8, 134.8, 132.7, 129.5, 127.2, 125.5, 83.8, 79.4, 60.9, 41.3, 28.4, 24.8, 21.3.
^{11}B NMR (128 MHz, CDCl$_3$) δ 32.8.
HRMS (ESI) calcd. for [$C_{32}H_{45}BN_2O_6S$, M-H]$^-$: 595.3018, found: 595.3017.

tert-butyl-(_E_)-4-(1-((4-methylphenyl)sulfonamido)-3-(naphthalen-2-yl)allyl)piperidine-1-carboxylate (41)

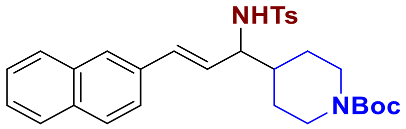

From 2-vinylnaphthalene (61.6 mg, 0.40 mmol, 2.0 equiv), *tert*-butyl-4-formylpiperidine-1-carboxylate (42.6 mg, 0.20 mmol, 1.0 equiv) and *p*-toluenesulfonamide (41.0 mg, 0.24 mmol, 1.2 equiv) the title compound was prepared following the general procedure **A** using Ni(COD)$_2$ (5.5 mg, 10 mol%), PCy$_3$ (11.2 mg, 20 mol%), Ti(O^{i}Pr)$_4$ (12 μL, 20 mol%), anhydrous acetonitrile (0.5 mL). The reaction mixture was stirred for 12 h at 100 °C. The crude material was purified by flash column chromatography (petroleum ether/acetone = 5:1) to provide the title compound as a white solid in 75% yield (78.1 mg). m.p.: 138−139 °C.
^{1}H NMR (400 MHz, CDCl$_3$) δ 7.80 – 7.67 (m, 5H), 7.49 – 7.42 (m, 2H), 7.41 (s, 1H), 7.23 (dd, *J* = 8.5, 1.7 Hz, 1H), 7.14 (d, *J* = 7.9 Hz, 2H), 6.17 (d, *J* = 15.8 Hz, 1H), 5.78 (dd, *J* = 15.8, 8.2 Hz, 1H), 4.76 (d, *J* = 8.6 Hz, 1H), 4.12 (s, 2H), 3.87 – 3.71 (m, 1H), 2.64 (s, 2H), 2.19 (s, 3H), 1.84 (d, *J* = 13.0 Hz, 1H), 1.68 – 1.57 (m, 2H), 1.44 (s, 9H), 1.21 (d, *J* = 15.4 Hz, 2H).
^{13}C NMR (101 MHz, CDCl$_3$) δ 154.7, 143.3, 137.9, 133.4, 133.3, 132.9, 132.9, 129.5, 127.8, 127.6, 127.3, 126.6, 126.3, 126.0, 123.3, 79.4, 61.0, 43.5, 41.3, 29.3, 28.4, 21.3.
HRMS (ESI) calcd. for [$C_{30}H_{36}N_2O_4S$, M-H]$^-$: 519.2323, found: 519.2320.

tert-butyl-(_E_)-4-(3-(5-methylfuran-2-yl)-1-((4-

methylphenyl)sulfonamido)allyl)piperidine-1-carboxylate (42)

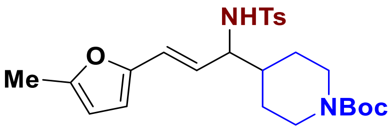

From 2-methyl-5-vinylfuran (43.2 mg, 0.40 mmol, 2.0 equiv), *tert*-butyl-4-formylpiperidine-1-carboxylate (42.6 mg, 0.20 mmol, 1.0 equiv) and *p*-toluenesulfonamide (41.0 mg, 0.24 mmol, 1.2 equiv) the title compound was prepared following the general procedure **A** using $Ni(COD)_2$ (5.5 mg, 10 mol%), PCy_3 (11.2 mg, 20 mol%), $Ti(O^iPr)_4$ (60 μL, 100 mol%), anhydrous acetonitrile (0.5 mL). The reaction mixture was stirred for 12 h at 100 °C. The crude material was purified by flash column chromatography (petroleum ether/acetone = 3:1) to provide the title compound as a white solid in 57% yield (54.0 mg). m.p.: 89−91 °C.

^{1}H NMR (400 MHz, $CDCl_3$) δ 7.72 (d, *J* = 7.9 Hz, 2H), 7.21 (d, *J* = 7.9 Hz, 2H), 5.92 (q, *J* = 3.3 Hz, 2H), 5.78 (d, *J* = 15.6 Hz, 1H), 5.64 – 5.49 (m, 1H), 4.82 (d, *J* = 8.5 Hz, 1H), 4.11 (s, 2H), 3.66 (d, *J* = 9.7 Hz, 1H), 2.70 – 2.54 (m, 2H), 2.36 (s, 3H), 2.26 (s, 3H), 1.77 (d, *J* = 13.2 Hz, 1H), 1.64 – 1.56 (m, 2H), 1.45 (s, 9H), 1.16 (d, *J* = 13.3 Hz, 2H).

^{13}C NMR (101 MHz, $CDCl_3$) δ 154.6, 152.1, 149.9, 143.2, 137.8, 129.4, 127.2, 122.9, 121.1, 109.7, 107.3, 79.4, 60.6, 43.5, 41.4, 28.4, 28.0, 21.4, 13.6.

HRMS (ESI) calcd. for $[C_{25}H_{34}N_2O_5S, M+Na]^+$: 497.2086, found: 497.2085.

***tert*-butyl-(*E*)-4-(1-((4-methylphenyl)sulfonamido)-3-(thiophen-2-yl)allyl)piperidine-1-carboxylate (43)**

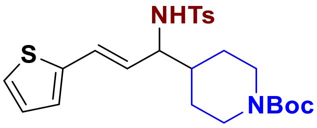

From 2-vinylthiophene (44.0 mg, 0.40 mmol, 2.0 equiv), *tert*-butyl-4-formylpiperidine-1-carboxylate (42.6 mg, 0.20 mmol, 1.0 equiv) and *p*-toluenesulfonamide (41.0 mg, 0.24 mmol, 1.2 equiv) the title compound was prepared following the general procedure **A** using $Ni(COD)_2$ (5.5 mg, 10 mol%), PCy_3 (11.2 mg, 20 mol%), $Ti(O^iPr)_4$ (12 μL, 20 mol%), anhydrous acetonitrile (0.5 mL). The reaction mixture was stirred for 12 h at 100 °C. The crude material was purified by flash column chromatography (petroleum ether/acetone = 3:1) to provide the title compound as a white solid in 35% yield (33.3 mg). m.p.: 150−151 °C.

^{1}H NMR (400 MHz, $CDCl_3$) δ 7.77 – 7.66 (m, 2H), 7.22 (s, 1H), 7.13 (d, *J* = 5.0 Hz, 1H), 6.92 (dd, *J* = 5.1, 3.5 Hz, 1H), 6.74 (d, *J* = 3.5 Hz, 1H), 6.16 (d, *J* = 15.6 Hz, 1H), 5.52 (dd, *J* = 15.6, 8.2 Hz, 1H), 4.91 (d, *J* = 8.7 Hz, 1H), 4.25 – 3.97 (m, 2H), 3.69 (d, *J* = 8.2 Hz, 1H), 2.63 (d, *J* = 12.4 Hz, 2H), 2.31 (s, 3H), 1.81 (d, *J* = 13.0 Hz, 1H), 1.67 – 1.53 (m, 1H), 1.46 (s, 10H), 1.18 (tt, *J* = 15.9, 8.0 Hz, 2H).

^{13}C NMR (101 MHz, $CDCl_3$) δ 154.6, 143.3, 140.9, 137.8, 129.5, 127.2, 125.9, 125.7, 124.4, 79.4, 60.7, 43.5, 41.2, 28.4, 28.2, 21.4.

HRMS (ESI) calcd. for $[C_{24}H_{32}N_2O_4S_2, M+Na]^+$: 499.1695, found: 499.1700.

***tert*-butyl-(*E*)-4-(1-((4-methylphenyl)sulfonamido)-3-((ferrocenyl)allyl)piperidine-1-carboxylate (44)**

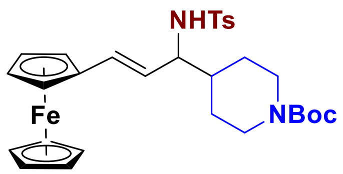

From vinylferrocene (84.8 mg, 0.40 mmol, 2.0 equiv), *tert*-butyl-4-formylpiperidine-1-carboxylate (42.6 mg, 0.20 mmol, 1.0 equiv) and *p*-toluenesulfonamide (41.0 mg, 0.24 mmol, 1.2 equiv) the title compound was prepared following the general procedure **A** using $Ni(COD)_2$ (5.5 mg, 10 mol%), PCy_3 (11.2 mg, 20 mol%), $Ti(O^iPr)_4$ (12 μL, 20 mol%), anhydrous acetonitrile (0.5 mL). The reaction mixture was stirred for 12 h at 100 °C. The crude material was purified by flash column chromatography (petroleum ether/acetone = 5:1) to provide the title compound as an orange solid in 69% yield (79.7 mg). m.p.: 156−157 °C.
^{1}H NMR (400 MHz, $CDCl_3$) δ 7.77 (d, *J* = 8.0 Hz, 2H), 7.32 – 7.18 (m, 2H), 5.89 (d, *J* = 15.6 Hz, 1H), 5.39 (dd, *J* = 15.8, 8.0 Hz, 1H), 4.89 (d, *J* = 8.3 Hz, 1H), 4.20 – 4.07 (m, 6H), 4.02 (s, 5H), 3.63 (d, *J* = 7.7 Hz, 1H), 2.63 (m, 2H), 2.39 (s, 3H), 1.76 (d, *J* = 12.5 Hz, 1H), 1.66 – 1.52 (m, 1H), 1.46 (s, 9H), 1.17 (t, *J* = 12.4 Hz, 2H).
^{13}C NMR (101 MHz, $CDCl_3$) δ 154.6, 143.3, 138.0, 130.6, 129.6, 127.1, 123.3, 81.7, 79.4, 69.0, 68.8, 68.7, 67.1, 66.2, 60.7, 44.0, 41.4, 28.4, 28.0, 21.5.
HRMS (ESI) calcd. for $[C_{30}H_{38}FeN_2O_4S, M-H]^-$: 577.1829, found: 577.1826.

***tert*-butyl-4-((2*E*,4*E*)-1-((4-methylphenyl)sulfonamido)-5-phenylpenta-2,4-dien-1-yl)piperidine-1-carboxylate (45)**

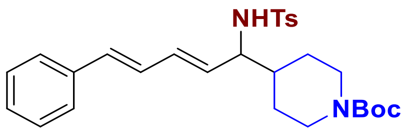

From buta-1,3-dien-1-ylbenzene (78 mg, 0.60 mmol, 3.0 equiv), *tert*-butyl-4-formylpiperidine-1-carboxylate (42.6 mg, 0.20 mmol, 1.0 equiv) and *p*-toluenesulfonamide (41.0 mg, 0.24 mmol, 1.2 equiv) the title compound was prepared following the general procedure **A** using $Ni(COD)_2$ (5.5 mg, 10 mol%), PCy_3 (11.2 mg, 20 mol%), $Ti(O^iPr)_4$ (60 μL, 100 mol%), anhydrous EtOH (0.5 mL). The reaction mixture was stirred for 12 h at 100 °C. The crude material was purified by flash column chromatography (petroleum ether/acetone = 3:1) to provide the title compound as a white solid in 40% yield (39.7 mg). m.p.: 159−160 °C.
^{1}H NMR (400 MHz, $CDCl_3$) δ 7.71 (d, *J* = 8.0 Hz, 2H), 7.35 – 7.29 (m, 4H), 7.24 (d, *J* = 5.8 Hz, 2H), 6.46 (dd, *J* = 15.6, 10.3 Hz, 1H), 6.30 (d, *J* = 15.6 Hz, 1H), 5.82 (dd, *J* = 15.2, 10.3 Hz, 1H), 5.32 (dd, *J* = 15.2, 8.1 Hz, 1H), 4.72 (d, *J* = 8.6 Hz, 1H), 4.11 (d, *J* = 6.1 Hz, 2H), 3.64 (d, *J* = 7.9 Hz, 1H), 2.60 (d, *J* = 12.1 Hz, 2H), 2.34 (s, 3H), 1.75 (d, *J* = 13.0 Hz, 1H), 1.61 – 1.51 (m, 2H), 1.44 (s, 9H), 1.15 (td, *J* = 12.4, 4.4 Hz,

2H).
^{13}C NMR (101 MHz, $CDCl_3$) δ 154.6, 143.4, 137.8, 136.8, 133.2, 129.9, 129.5, 128.6, 127.8, 127.3, 127.3, 126.3, 60.4, 43.8, 41.3, 28.4, 28.1, 21.4.
HRMS (ESI) calcd. for $[C_{28}H_{36}N_2O_4S, M\text{-}H]^-$: 495.2323, found: 495.2324.

***tert*-butyl-(*E*)-4-(3-(9H-carbazol-9-yl)-1-((4-methylphenyl)sulfonamido)allyl)piperidine-1-carboxylate (46)**

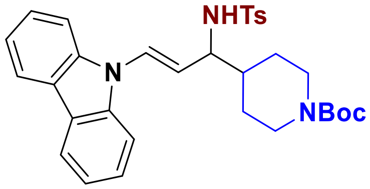

From 9-Vinylcarbazole (77.2 mg, 0.40 mmol, 2.0 equiv), *tert*-butyl-4-formylpiperidine-1-carboxylate (42.6 mg, 0.20 mmol, 1.0 equiv) and *p*-toluenesulfonamide (41.0 mg, 0.24 mmol, 1.2 equiv) the title compound was prepared following the general procedure **A** using $Ni(COD)_2$ (5.5 mg, 10 mol%), PCy_3 (11.2 mg, 20 mol%), $Ti(O^iPr)_4$ (60 μL, 100 mol%), anhydrous acetonitrile (0.5 mL). The reaction mixture was stirred for 12 h at 100 °C. The crude material was purified by flash column chromatography (petroleum ether/acetone = 3:1) to provide the title compound as a white solid in 62% yield (69.3 mg). m.p.: 119−120 ℃.
^{1}H NMR (400 MHz, $CDCl_3$) δ 8.01 (d, *J* = 7.7 Hz, 2H), 7.73 (d, *J* = 7.9 Hz, 2H), 7.39 (t, *J* = 7.7 Hz, 2H), 7.29 – 7.19 (m, 4H), 6.96 (d, *J* = 7.9 Hz, 2H), 6.71 (d, *J* = 14.2 Hz, 1H), 5.56 (dd, *J* = 14.2, 9.1 Hz, 1H), 5.45 (d, *J* = 8.4 Hz, 1H), 4.17 (d, *J* = 36.9 Hz, 2H), 3.80 (d, *J* = 8.8 Hz, 1H), 2.70 – 2.55 (m, 2H), 1.95 (s, 3H), 1.72 (q, *J* = 12.3 Hz, 3H), 1.43 (s, 9H), 1.25 (q, *J* = 10.9, 8.6 Hz, 2H).
^{13}C NMR (101 MHz, $CDCl_3$) δ 154.7, 143.5, 138.9, 137.9, 129.5, 127.2, 126.4, 126.2, 123.9, 120.8, 120.1, 114.4, 110.4, 60.3, 41.5, 28.6, 28.4, 21.0.
HRMS (ESI) calcd. for $[C_{32}H_{37}N_3O_4S, M\text{-}H]^-$: 558.2432, found: 558.2430.

***tert*-butyl-(*E*)-4-(1-((4-methylphenyl)sulfonamido)-4-(trimethylsilyl)but-3-en-1-yl)piperidine-1-carboxylate (47)**

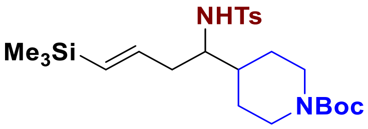

From allyltrimethylsilane (68.4 mg, 0.60 mmol, 3.0 equiv), *tert*-butyl-4-formylpiperidine-1-carboxylate (42.6 mg, 0.20 mmol, 1.0 equiv) and *p*-toluenesulfonamide (41.0 mg, 0.24 mmol, 1.2 equiv) the title compound was prepared following the general procedure **A** using $Ni(COD)_2$ (5.5 mg, 10 mol%), PCy_3 (11.2 mg, 20 mol%), $Ti(O^iPr)_4$ (60 μL, 100 mol%), anhydrous EtOH (0.5 mL). The reaction mixture was stirred for 12 h at 100 °C. The crude material was purified by flash column

chromatography (petroleum ether/acetone = 4:1) to provide the title compound as a white solid in 35% yield (33.5 mg). m.p.: 143−145 °C.

^{1}H NMR (400 MHz, $CDCl_3$) δ 7.75 (d, *J* = 7.9 Hz, 2H), 7.36 – 7.23 (m, 2H), 5.78 – 5.42 (m, 2H), 4.40 (d, *J* = 8.0 Hz, 1H), 4.13 (s, 2H), 3.17 (p, *J* = 6.2 Hz, 1H), 2.63 (d, *J* = 20.7 Hz, 2H), 2.45 (s, 3H), 2.13 (s, 2H), 1.70 – 1.57 (m, 3H), 1.46 (s, 9H), 1.14 (ddd, *J* = 29.8, 12.9, 6.7 Hz, 2H), 0.01 (s, 9H).

^{13}C NMR (101 MHz, $CDCl_3$) δ 156.0, 144.7, 141.9, 139.3, 137.4, 131.1, 128.4, 80.7, 58.4, 45.2, 41.0, 39.8, 29.8, 29.1, 22.9, -0.0.

HRMS (ESI) calcd. for [$C_{24}H_{40}N_2O_5SSi$, M-H]$^-$: 479.2405, found: 479.2403.

***tert*-butyl-(*E*)-4-(1-((4-methylphenyl)sulfonamido)-4-(trimethylsilyl)but-2-en-1-yl)piperidine-1-carboxylate (47′)**

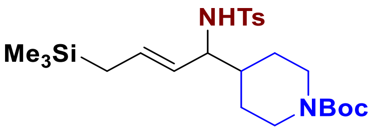

White solid in 37% yield (35.6 mg). m.p.: 148−150 °C.

^{1}H NMR (400 MHz, $CDCl_3$) δ 7.72 (d, *J* = 8.1 Hz, 2H), 7.31 – 7.27 (m, 2H), 5.21 (dt, *J* = 15.8, 8.1 Hz, 1H), 4.91 (dd, *J* = 15.2, 8.1 Hz, 1H), 4.59 (d, *J* = 8.3 Hz, 1H), 4.10 (s, 2H), 3.52 (d, *J* = 7.6 Hz, 1H), 2.63 (d, *J* = 21.3 Hz, 2H), 2.42 (s, 3H), 1.69 (s, 1H), 1.64 – 1.49 (m, 3H), 1.45 (s, 9H), 1.25 (dd, *J* = 13.8, 5.4 Hz, 2H), 1.15 – 1.06 (m, 2H), -0.10 (d, *J* = 0.9 Hz, 9H).

^{13}C NMR (101 MHz, $CDCl_3$) δ 156.6, 145.0, 140.1, 133.1, 131.4, 129.1, 126.9, 81.3, 62.8, 46.0, 43.5, 30.4, 29.8, 24.8, 23.5, -0.00.

HRMS (ESI) calcd. for [$C_{24}H_{40}N_2O_5SSi$, M-H]$^-$: 479.2405, found: 479.2403.

***tert*-butyl-(*E*)-4-(1-((4-methylphenyl)sulfonamido)-5-phenylpent-3-en-1-yl)piperidine-1-carboxylate (mixture H/A = 3.8:1) (48 and 48′)**

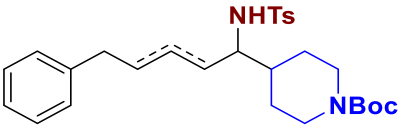

From but-3-en-1-ylbenzene (79.2 mg, 0.60 mmol, 3.0 equiv), *tert*-butyl-4-formylpiperidine-1-carboxylate (42.6 mg, 0.20 mmol, 1.0 equiv) and *p*-toluenesulfonamide (41.0 mg, 0.24 mmol, 1.2 equiv) the title compound was prepared following the general procedure **A** using Ni(COD)$_2$ (5.5 mg, 10 mol%), PCy$_3$ (11.2 mg, 20 mol%), Ti(O^{i}Pr)$_4$ (60 μL, 100 mol%), anhydrous EtOH (0.5 mL). The reaction mixture was stirred for 12 h at 100 °C. The crude material was purified by flash column chromatography (petroleum ether/acetone = 4:1) to provide the title compound as a white solid in 89% yield with a mixture containing isomers. (3.8:1 homoallylic amine/allylic amine, 88.6 mg). m.p.: 105−107 °C.

^{1}H NMR (400 MHz, $CDCl_3$) δ 7.72 (d, *J* = 8.0 Hz, 2H), 7.26 (dd, *J* = 8.1, 6.4 Hz, 5H), 7.11 – 7.03 (m, 2H), 5.61 – 5.37 (m, 1H), 5.15 (ddt, *J* = 22.4, 14.8, 7.5 Hz, 1H), 4.76

(d, J = 8.6 Hz, 1H), 4.10 (s, 2H), 3.16 (dd, J = 28.1, 7.6 Hz, 3H), 2.55 (s, 2H), 2.39 (d, J = 9.1 Hz, 3H), 2.02 (s, 2H), 1.67 – 1.51 (m, 3H), 1.44 (s, 9H), 1.20 – 1.03 (m, 2H).
^{13}C NMR (101 MHz, $CDCl_3$) δ 154.6, 143.3, 143.3, 140.2, 140.1, 138.1, 138.0, 133.6, 131.6, 129.6, 129.6, 129.4, 129.1, 128.5, 128.4, 128.3, 128.1, 127.2, 127.0, 127.0, 126.1, 126.1, 125.9, 124.8, 79.3, 67.5, 57.8, 57.5, 43.6, 39.6, 39.4, 38.9, 38.8, 35.0, 34.3, 33.3, 29.2, 28.4, 28.1, 21.5.
HRMS (ESI) calcd. for $[C_{28}H_{38}N_2O_4S, M\text{-}H]^-$: 497.2479, found: 497.2477.

***tert*-butyl-(*E*)-4-(1-((4-methylphenyl)sulfonamido)oct-3-en-1-yl)piperidine-1-carboxylate (mixture A/H= 3:1) (49 and 49′)**

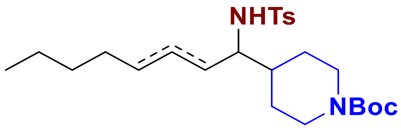

From hept-1-ene (58.8 mg, 0.60 mmol, 3.0 equiv), *tert*-butyl-4-formylpiperidine-1-carboxylate (42.6 mg, 0.20 mmol, 1.0 equiv) and *p*-toluenesulfonamide (41.0 mg, 0.24 mmol, 1.2 equiv) the title compound was prepared following the general procedure **A** using $Ni(COD)_2$ (5.5 mg, 10 mol%), PCy_3 (11.2 mg, 20 mol%), $Ti(O^iPr)_4$ (60 μL, 100 mol%), anhydrous EtOH (0.5 mL). The reaction mixture was stirred for 12 h at 100 °C. The crude material was purified by flash column chromatography (petroleum ether/acetone = 4:1) to provide the title compound as a white solid in 62% yield with a mixture containing isomers. (3:1 homoallylic amine/allylic amine, 57.5 mg). m.p.: 119−120 °C.
^{1}H NMR (400 MHz, $CDCl_3$) δ 7.73 (dd, J = 8.3, 2.7 Hz, 2H), 7.29 (t, J = 6.8 Hz, 2H), 5.33 (ddd, J = 28.9, 16.1, 7.3 Hz, 1H), 4.98 (tt, J = 14.9, 8.4 Hz, 1H), 4.47 (d, J = 8.3 Hz, 1H), 4.12 (dd, J = 15.5, 7.9 Hz, 2H), 3.17 – 2.97 (m, 1H), 2.59 (s, 2H), 2.43 (s, 3H), 2.11 – 1.79 (m, 4H), 1.68 – 1.48 (m, 3H), 1.45 (s, 9H), 1.29 – 1.04 (m, 6H), 0.91 – 0.80 (m, 3H).
^{13}C NMR (101 MHz, $CDCl_3$) δ 154.6, 143.2, 138.0, 135.6, 129.5, 127.1, 127.0, 123.9, 79.3, 57.8, 57.4, 43.8, 39.5, 39.3, 34.2, 32.2, 31.6, 31.4, 28.4, 28.0, 27.0 , 22.2, 22.2, 21.5, 13.9, 13.8.
HRMS (ESI) calcd. for $[C_{25}H_{40}N_2O_4S, M\text{-}H]^-$: 463.2636, found: 463.2634.

***tert*-butyl-4-(3-cyclohexylidene-1-((4-methylphenyl)sulfonamido)propyl)piperidine-1-carboxylate (50)**

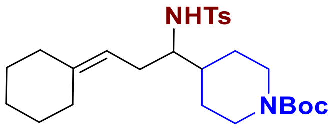

From vinylcyclohexane (66.0 mg, 0.60 mmol, 3.0 equiv), *tert*-butyl-4-formylpiperidine-1-carboxylate (42.6 mg, 0.20 mmol, 1.0 equiv) and *p*-toluenesulfonamide (41.0 mg, 0.24 mmol, 1.2 equiv) the title compound was prepared

following the general procedure **A** using Ni(COD)$_2$ (5.5 mg, 10 mol%), PCy$_3$ (11.2 mg, 20 mol%), Ti(O^{i}Pr)$_4$ (60 µL, 100 mol%), anhydrous EtOH (0.5 mL). The reaction mixture was stirred for 12 h at 100 °C. The crude material was purified by flash column chromatography (petroleum ether/acetone = 4:1) to provide the title compound as a white solid in 50% yield (47.6 mg). m.p.: 131−132 °C.

^{1}H NMR (400 MHz, CDCl$_3$) δ 7.74 (d, *J* = 8.0 Hz, 2H), 7.32 (d, *J* = 8.0 Hz, 2H), 4.69 (t, *J* = 7.4 Hz, 1H), 4.29 (d, *J* = 8.3 Hz, 1H), 4.14 (s, 2H), 3.08 (p, *J* = 6.0 Hz, 1H), 2.63 (d, *J* = 17.2 Hz, 2H), 2.45 (s, 3H), 2.01 (d, *J* = 6.6 Hz, 2H), 1.94 (dd, *J* = 14.0, 8.3 Hz, 4H), 1.59 (dd, *J* = 25.5, 11.5 Hz, 7H), 1.47 (s, 9H), 1.34 – 0.87 (m, 4H).

^{13}C NMR (101 MHz, CDCl$_3$) δ 154.6, 143.9, 143.2, 137.9, 129.5, 127.1, 114.9, 79.3, 58.0, 43.8, 39.3, 37.2, 28.6, 28.6, 28.4, 28.4, 28.2, 27.8, 26.6, 21.5.

HRMS (ESI) calcd. for [$C_{26}H_{40}N_2O_4S$, M-H]$^-$: 475.2636, found: 475.2633.

***tert*-butyl-(*E*)-4-(1-((4-methylphenyl)sulfonamido)-4-phenylbut-3-en-1-yl)piperidine-1-carboxylate (51)**

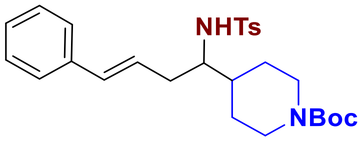

From allylbenzene (47.2 mg, 0.40 mmol, 2.0 equiv), *tert*-butyl-4-formylpiperidine-1-carboxylate (42.6 mg, 0.20 mmol, 1.0 equiv) and *p*-toluenesulfonamide (41.0 mg, 0.24 mmol, 1.2 equiv) the title compound was prepared following the general procedure **A** using Ni(COD)$_2$ (5.5 mg, 10 mol%), PCy$_3$ (11.2 mg, 20 mol%), Ti(O^{i}Pr)$_4$ (60 µL, 100 mol%), anhydrous EtOH (0.5 mL). The reaction mixture was stirred for 12 h at 100 °C. The crude material was purified by flash column chromatography (petroleum ether/acetone = 3:1) to provide the title compound as a white solid in 85% yield (82.3 mg). m.p.: 95−97 °C.

^{1}H NMR (400 MHz, CDCl$_3$) δ 7.78 – 7.64 (m, 2H), 7.35 – 7.24 (m, 4H), 7.19 (t, *J* = 7.0 Hz, 3H), 6.25 (d, *J* = 15.7 Hz, 1H), 5.71 (dt, *J* = 15.3, 7.4 Hz, 1H), 4.42 (d, *J* = 8.3 Hz, 1H), 4.14 (d, *J* = 7.1 Hz, 2H), 3.23 (p, *J* = 6.1 Hz, 1H), 2.64 (s, 2H), 2.39 (s, 3H), 2.30 – 2.16 (m, 2H), 1.67 (d, *J* = 13.2 Hz, 3H), 1.47 (s, 9H), 1.29 – 1.13 (m, 2H).

^{13}C NMR (101 MHz, CDCl$_3$) δ 154.6, 143.2, 137.9, 136.7, 133.7, 129.6, 128.4, 127.4, 127.0, 126.1, 124.6, 79.4, 57.7, 43.4, 40.2, 34.9, 28.4, 27.9, 21.5.

HRMS (ESI) calcd. for [$C_{27}H_{36}N_2O_4S$, M-H]$^-$: 483.2323, found: 483.2320.

***tert*-butyl-(*E*)-4-(4-(4-methoxyphenyl)-1-((4-methylphenyl)sulfonamido)but-3-en-1-yl)piperidine-1-carboxylate (52)**

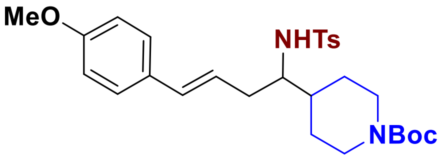

From 1-allyl-4-methoxybenzene (59.2 mg, 0.40 mmol, 2.0 equiv), *tert*-butyl-4-

formylpiperidine-1-carboxylate (42.6 mg, 0.20 mmol, 1.0 equiv) and *p*-toluenesulfonamide (41.0 mg, 0.24 mmol, 1.2 equiv) the title compound was prepared following the general procedure **A** using $Ni(COD)_2$ (5.5 mg, 10 mol%), PCy_3 (11.2 mg, 20 mol%), $Ti(O^iPr)_4$ (60 µL, 100 mol%), anhydrous EtOH (0.5 mL). The reaction mixture was stirred for 12 h at 100 °C. The crude material was purified by flash column chromatography (petroleum ether/acetone = 3:1) to provide the title compound as a white solid in 83% yield (85.3 mg). m.p.: 131−132 °C.

^{1}H NMR (400 MHz, $CDCl_3$) δ 7.74 – 7.63 (m, 2H), 7.23 – 7.04 (m, 4H), 6.83 (t, *J* = 5.0 Hz, 2H), 6.17 (d, *J* = 15.7 Hz, 1H), 5.62 – 5.50 (m, 1H), 4.40 (d, *J* = 7.9 Hz, 1H), 4.13 (s, 2H), 3.82 (d, *J* = 3.6 Hz, 3H), 3.18 (s, 1H), 2.62 (s, 2H), 2.38 (d, *J* = 3.5 Hz, 3H), 2.17 (s, 2H), 1.64 (d, *J* = 12.9 Hz, 3H), 1.45 (d, *J* = 3.7 Hz, 9H), 1.25 – 1.09 (m, 2H).

^{13}C NMR (101 MHz, $CDCl_3$) δ 159.0, 154.6, 143.1, 138.0, 133.0, 129.6, 127.3, 126.9, 122.4, 113.8, 79.3, 57.8, 55.3, 43.7, 40.1, 34.8, 28.4, 27.9, 21.5.

HRMS (ESI) calcd. for $[C_{28}H_{38}N_2O_5S, M-H]^-$: 513.2428, found: 513.2428.

(*E*)-N-cinnamyl-4-(5-(*p*-tolyl)-3-(trifluoromethyl)-1H-pyrazol-1-yl)-benzenesulfonamide (53)

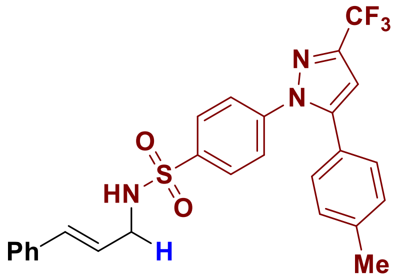

From styrene (35 µL, 0.30 mmol, 1.5 equiv), paraformaldehyde (6.0 mg, 0.20 mmol, 1.0 equiv) and celecoxib (91.4 mg, 0.24 mmol, 1.2 equiv) the title compound was prepared following the general procedure **A** using $Ni(COD)_2$ (5.5 mg, 10 mol%), PCy_3 (11.2 mg, 20 mol%), $Ti(O^iPr)_4$ (12 µL, 20 mol%), anhydrous acetonitrile (0.5 mL). The reaction mixture was stirred for 12 h at 100 °C. The crude material was purified by flash column chromatography (petroleum ether/EtOAc = 4:1) to provide the title compound as a white solid in 58% yield (57.6 mg). m.p.: 167−169 °C.

^{1}H NMR (400 MHz, $CDCl_3$) δ 7.92 – 7.82 (m, 2H), 7.49 – 7.43 (m, 2H), 7.32 – 7.26 (m, 3H), 7.26 – 7.20 (m, 2H), 7.15 (d, *J* = 8.0 Hz, 2H), 7.09 (d, *J* = 8.2 Hz, 2H), 6.74 (s, 1H), 6.46 (dt, *J* = 15.9, 1.6 Hz, 1H), 6.01 (dt, *J* = 15.8, 6.4 Hz, 1H), 4.68 (s, 1H), 3.77 (td, *J* = 6.3, 1.5 Hz, 2H), 2.37 (s, 3H).

^{13}C NMR (101 MHz, $CDCl_3$) δ 145.2, 144.1 (q, *J* = 38.7 Hz), 142.6, 139.8, 139.5, 135.8, 133.6, 129.8, 129.7, 128.68 (d, *J* = 3.3 Hz), 128.1, 128.1, 126.5, 125.6, 125.5, 123.5, 121.1 (q, *J* = 269.6 Hz), 106.3, 45.5, 21.3.

^{19}F NMR (376 MHz, $CDCl_3$) δ -62.4.

HRMS (ESI) calcd. for $[C_{26}H_{22}F_3N_3O_2S, M-H]^-$: 496.1312, found: 496.1310.

(*E*)-N-(1-cyclohexyl-3-phenylallyl)-4-(5-(*p*-tolyl)-3-(trifluoromethyl)-1H-pyrazol-1-yl)-benzenesulfonamide (54)

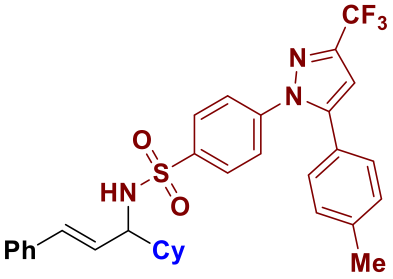

From styrene (35 µL, 0.30 mmol, 1.5 equiv), cyclohexanecarboxaldehyde (24 µL, 0.20 mmol, 1.0 equiv) and celecoxib (91.4 mg, 0.24 mmol, 1.2 equiv) the title compound was prepared following the general procedure **A** using $Ni(COD)_2$ (5.5 mg, 10 mol%), PCy_3 (11.2 mg, 20 mol%), $Ti(O^iPr)_4$ (12 µL, 20 mol%), anhydrous acetonitrile (0.5 mL). The reaction mixture was stirred for 12 h at 100 °C. The crude material was purified by flash column chromatography (petroleum ether/EtOAc = 4:1) to provide the title compound as a white solid in 52% yield (60.2 mg). m.p.: 173−174 °C.

^{1}H NMR (400 MHz, $CDCl_3$) δ 8.09 – 7.96 (m, 2H), 7.57 (d, *J* = 8.5 Hz, 2H), 7.48 – 7.40 (m, 3H), 7.37 – 7.33 (m, 2H), 7.30 (d, *J* = 7.9 Hz, 2H), 7.22 (s, 2H), 6.93 (s, 1H), 6.39 (d, *J* = 15.8 Hz, 1H), 5.99 (dd, *J* = 15.8, 7.9 Hz, 1H), 5.15 (d, *J* = 8.1 Hz, 1H), 3.99 (q, *J* = 7.6 Hz, 1H), 2.57 (s, 3H), 2.06 – 1.91 (m, 3H), 1.69 (tdd, *J* = 11.7, 6.0, 3.0 Hz, 1H), 1.46 – 1.29 (m, 3H), 1.21 (pd, *J* = 12.1, 3.0 Hz, 2H).

^{13}C NMR (151 MHz, $CDCl_3$) δ 145.0, 143.9 (q, *J* = 38.4 Hz), 142.2, 140.6, 139.6, 136.0, 132.4, 129.7, 129.6, 128.6 (d, *J* = 11.6 Hz), 128.2, 127.8, 127.2, 126.3, 125.7, 125.1, 121.1 (q, *J* = 269.0 Hz), 106.2, 61.5, 42.9, 29.0, 26.2, 25.9, 21.3.

^{19}F NMR (376 MHz, $CDCl_3$) δ -62.37.

HRMS (ESI) calcd. for $[C_{32}H_{32}F_3N_3O_2S, M\text{-}H]^-$: 578.2094, found: 578.2090.

(*S*)-5-(N-cinnamylsulfamoyl)-N-((1-ethylpyrrolidin-2-yl)methyl)-2-methoxybenzamide (55)

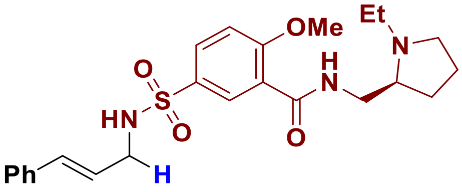

From styrene (46 µL, 0.40 mmol, 2.0 equiv), paraformaldehyde (6.0 mg, 0.20 mmol, 1.0 equiv) and levosulpiride (81.8 mg, 0.24 mmol, 1.2 equiv) the title compound was prepared following the general procedure **A** using $Ni(COD)_2$ (5.5 mg, 10 mol%), PCy_3 (11.2 mg, 20 mol%), $Ti(O^iPr)_4$ (60 µL, 100 mol%), anhydrous acetonitrile (0.5 mL). The reaction mixture was stirred for 12 h at 100 °C. The crude material was purified by flash column chromatography (DCM/MeOH = 10:1) to provide the title compound as a white solid in 35% yield (32.0 mg). m.p.: 152−153 °C.

^{1}H NMR (400 MHz, $CDCl_3$) δ 8.74 (dt, *J* = 5.4, 1.9 Hz, 1H), 8.31 (s, 1H), 7.97 (dd, *J* = 8.7, 2.5 Hz, 1H), 7.25 – 7.17 (m, 4H), 7.03 – 6.94 (m, 1H), 6.42 (dd, *J* = 16.7, 6.1 Hz,

1H), 6.03 (dt, J = 15.9, 6.3 Hz, 1H), 4.00 – 3.90 (m, 3H), 3.75 (d, J = 7.2 Hz, 3H), 3.32 (d, J = 13.9 Hz, 1H), 3.21 (s, 1H), 2.88 – 2.80 (m, 1H), 2.64 (s, 1H), 2.21 (d, J = 13.0 Hz, 2H), 1.89 (s, 1H), 1.67 (d, J = 42.9 Hz, 4H), 1.12 (p, J = 4.3 Hz, 3H).
^{13}C NMR (101 MHz, $CDCl_3$) δ 164.0, 160.2, 136.3, 133.1, 132.7, 131.8, 131.5, 128.4, 128.3, 127.6, 126.3, 124.4, 122.7, 111.6, 62.0, 56.2, 53.5, 47.8, 45.5, 41.4, 28.3, 22.9, 14.1.
HRMS (ESI) calcd. for $[C_{24}H_{31}N_3O_4S, M-H]^-$: 456.1962, found: 456.1963.

5-(N-((*E*)-1-cyclohexyl-3-phenylallyl)sulfamoyl)-N-(((*S*)-1-ethylpyrrolidin-2-yl)methyl)-2-methoxybenzamide (56)

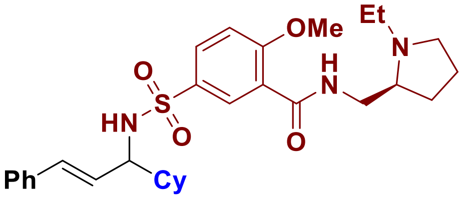

From styrene (46 µL, 0.40 mmol, 2.0 equiv), cyclohexanecarboxaldehyde (24 µL, 0.20 mmol, 1.0 equiv) and levosulpiride (81.8 mg, 0.24 mmol, 1.2 equiv) the title compound was prepared following the general procedure **A** using $Ni(COD)_2$ (5.5 mg, 10 mol%), PCy_3 (11.2 mg, 20 mol%), $Ti(O^iPr)_4$ (60 µL, 100 mol%), anhydrous acetonitrile (0.5 mL). The reaction mixture was stirred for 12 h at 100 °C. The crude material was purified by flash column chromatography (DCM/MeOH = 10:1) to provide the title compound as a white solid in 34% yield with a mixture containing diastereoisomers. (1:1 dr, 36.6 mg). m.p.: 160−161 °C.
^{1}H NMR (400 MHz, $CDCl_3$) δ 8.65 (d, J = 2.3 Hz, 1H), 8.16 (d, J = 6.9 Hz, 1H), 7.80 (dd, J = 8.7, 2.3 Hz, 1H), 7.15 (dd, J = 10.4, 7.0 Hz, 3H), 7.01 (d, J = 7.2 Hz, 2H), 6.73 (d, J = 8.7 Hz, 1H), 5.99 (dd, J = 16.0, 7.6 Hz, 1H), 5.59 (dd, J = 15.9, 8.4 Hz, 1H), 4.89 (d, J = 39.5 Hz, 1H), 3.72 (s, 5H), 3.29 (d, J = 13.9 Hz, 1H), 3.19 (d, J = 7.9 Hz, 1H), 2.85 (dd, J = 12.5, 7.2 Hz, 1H), 2.67 (s, 1H), 2.23 (dq, J = 26.2, 8.6, 7.6 Hz, 2H), 1.90 – 1.80 (m, 2H), 1.71 (d, J = 12.3 Hz, 4H), 1.63 – 1.54 (m, 3H), 1.42 (s, 1H), 1.11 (q, J = 6.5 Hz, 6H), 0.97 (d, J = 12.1 Hz, 2H).
^{13}C NMR (101 MHz, $CDCl_3$) δ 163.8, 159.9, 136.4, 134.1, 131.9, 131.8, 128.1, 127.3, 127.3, 127.2, 126.2, 122.3, 111.3, 62.3, 61.8, 56.0, 53.5, 48.0, 42.7, 41.4, 41.3, 29.2, 29.2, 28.4, 26.2, 25.9, 22.9, 14.0, 13.9.
HRMS (ESI) calcd. for $[C_{30}H_{41}N_3O_4S, M-H]^-$: 538.2745, found: 538.2740.

N-cinnamyl-1-((1*R*,4*S*)-7,7-dimethyl-2-oxobicyclo[2.2.1]heptan-1-yl)methanesulfonamide (57)

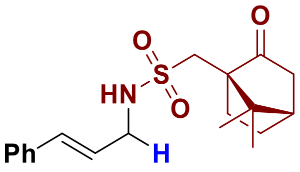

From styrene (46 µL, 0.40 mmol, 2.0 equiv), paraformaldehyde (6.0 mg, 0.20 mmol,

1.0 equiv) and (1*R*)-10-camphorsulfonamide (55.4 mg, 0.24 mmol, 1.2 equiv) the title compound was prepared following the general procedure **B** using $Ni(OAc)_2$ (3.5 mg, 10 mol%), PCy_3 (11.2 mg, 20 mol%), Mn (2.2 mg, 20 mol%), $Ti(O^iPr)_4$ (60 μL, 100 mol%), anhydrous acetonitrile (0.5 mL). The reaction mixture was stirred for 12 h at 100 °C. The crude material was purified by flash column chromatography (petroleum ether/acetone = 4:1) to provide the title compound as colorless sticky oil in 37% yield (25.7 mg).

^{1}H NMR (600 MHz, $CDCl_3$) δ 7.38 – 7.25 (m, 5H), 6.64 (d, *J* = 15.8 Hz, 1H), 6.27 – 6.17 (m, 1H), 5.39 (t, *J* = 6.4 Hz, 1H), 3.97 (hept, *J* = 7.6, 6.8 Hz, 2H), 3.44 (d, *J* = 15.1 Hz, 1H), 2.97 (d, *J* = 15.1 Hz, 1H), 2.40 (dt, *J* = 18.6, 3.9 Hz, 1H), 2.21 (td, *J* = 13.7, 5.5 Hz, 1H), 2.12 (d, *J* = 4.5 Hz, 1H), 2.05 – 1.91 (m, 3H), 1.48 – 1.42 (m, 1H), 0.99 (s, 3H), 0.84 (s, 3H).

^{13}C NMR (151 MHz, $CDCl_3$) δ 217.1, 136.2, 133.2, 128.6, 127.9, 126.5, 124.7, 59.3, 50.6, 48.8, 45.9, 43.0, 42.7, 27.0, 26.8, 19.8, 19.4.

HRMS (ESI) calcd. for $[C_{19}H_{25}NO_3S, M-H]^-$: 346.1482, found: 346.1480.

N-((*E*)-1-cyclohexyl-3-phenylallyl)-1-((1*R*,4*S*)-7,7-dimethyl-2-oxobicyclo[2.2.1]heptan-1-yl)methanesulfonamide (58)

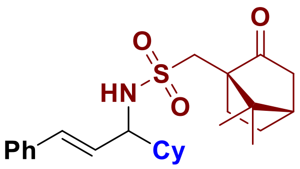

From styrene (46 μL, 0.40 mmol, 2.0 equiv), cyclohexanecarboxaldehyde (24 μL, 0.20 mmol, 1.0 equiv) and (1*R*)-10-camphorsulfonamide (55.4 mg, 0.24 mmol, 1.2 equiv) the title compound was prepared following the general procedure **A** using $Ni(COD)_2$ (5.5 mg, 10 mol%), PCy_3 (11.2 mg, 20 mol%), $Ti(O^iPr)_4$ (60 μL, 100 mol%), anhydrous acetonitrile (0.5 mL). The reaction mixture was stirred for 12 h at 100 °C. The crude material was purified by flash column chromatography (petroleum ether/acetone = 4:1) to provide the title compound as colorless sticky oil in 58% yield with a mixture containing diastereoisomers. (1:1 dr, 49.8 mg).

^{1}H NMR (400 MHz, $CDCl_3$) δ 7.43 – 7.27 (m, 4H), 7.22 (td, *J* = 7.1, 4.7 Hz, 1H), 6.57 (dd, *J* = 33.0, 15.9 Hz, 1H), 6.10 (ddd, *J* = 19.0, 15.9, 8.7 Hz, 1H), 5.91 (d, *J* = 9.6 Hz, 0H), 5.22 (d, *J* = 8.0 Hz, 0H), 4.02 – 3.83 (m, 1H), 3.49 – 3.31 (m, 1H), 2.94 (dd, *J* = 22.5, 15.0 Hz, 1H), 2.46 – 2.16 (m, 2H), 2.13 – 1.81 (m, 6H), 1.79 – 1.50 (m, 6H), 1.41 (td, *J* = 9.4, 4.1 Hz, 1H), 1.23 – 1.06 (m, 4H), 1.02 (s, 1H), 0.84 (d, *J* = 14.3 Hz, 3H), 0.49 (s, 1H).

^{13}C NMR (101 MHz, $CDCl_3$) δ 217.7, 216.0, 136.5, 136.0, 132.8, 132.2, 128.6, 128.5, 128.4, 127.9, 127.6, 126.5, 126.3, 62.6, 61.3, 59.8, 59.1, 52.4, 51.8, 48.8, 48.3, 43.1, 43.1, 42.8, 42.8, 42.7, 29.3, 29.2, 29.2, 29.1, 28.0, 27.0, 26.9, 26.4, 26.3, 26.3, 26.0, 26.0, 26.0, 19.8, 19.6, 19.6, 19.2.

HRMS (ESI) calcd. for $[C_{25}H_{35}NO_3S, M-H]^-$:428.2265, found: 428.2260.

(*E*)-N-(7-(2,5-dimethylphenoxy)-4,4-dimethyl-1-phenylhept-1-en-3-yl)-4-methylbenzenesulfonamide (59)

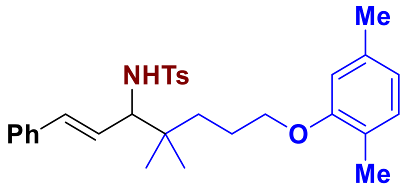

From styrene (46 μL, 0.40 mmol, 2.0 equiv), 5-(2,5-dimethylphenoxy)-2,2-dimethylpentanal (46.8 mg, 0.20 mmol, 1.0 equiv) and *p*-toluenesulfonamide (41.0 mg, 0.24 mmol, 1.2 equiv) the title compound was prepared following the general procedure **A** using Ni(COD)$_2$ (5.5 mg, 10 mol%), PCy$_3$ (11.2 mg, 20 mol%), Ti(O^{i}Pr)$_4$ (60 μL, 100 mol%), anhydrous EtOH (0.5 mL). The reaction mixture was stirred for 12 h at 100 °C. The crude material was purified by flash column chromatography (petroleum ether/EtOAc = 5:1) to provide the title compound as a white solid in 32% yield (31.4 mg). m.p.: 105−106 °C.

^{1}H NMR (400 MHz, CDCl$_3$) δ 7.68 (d, *J* = 8.0 Hz, 2H), 7.20 (dd, *J* = 10.9, 7.0 Hz, 3H), 7.09 (d, *J* = 8.0 Hz, 2H), 6.99 (t, *J* = 7.6 Hz, 3H), 6.65 (d, *J* = 7.5 Hz, 1H), 6.59 (s, 1H), 5.94 (d, *J* = 15.8 Hz, 1H), 5.69 (dd, *J* = 15.8, 8.5 Hz, 1H), 4.65 (d, *J* = 9.4 Hz, 1H), 3.86 (qd, *J* = 9.0, 4.6 Hz, 2H), 3.69 (t, *J* = 9.0 Hz, 1H), 2.30 (s, 3H), 2.17 (s, 3H), 2.13 (s, 3H), 1.77 (td, *J* = 15.3, 13.6, 6.4 Hz, 2H), 1.45 (t, *J* = 8.4 Hz, 2H), 0.96 (s, 3H), 0.91 (s, 3H).

^{13}C NMR (101 MHz, CDCl$_3$) δ 156.9, 143.2, 137.9, 136.4, 136.2, 133.1, 130.2, 129.4, 128.2, 127.6, 127.3, 126.2, 125.0, 123.5, 120.6, 111.9, 68.2, 64.1, 37.3, 35.5, 23.8, 23.5, 23.4, 21.4, 21.2, 15.7.

HRMS (ESI) calcd. for [$C_{30}H_{37}NO_3S$, M-H]$^-$: 490.2421, found: 490.2418.

(*E*)-N-(5,9-dimethyl-1-phenyldeca-1,8-dien-3-yl)-4-methylbenzenesulfonamide (60)

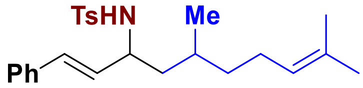

From styrene (46 μL, 0.40 mmol, 2.0 equiv), citronellal (30.8 mg, 0.20 mmol, 1.0 equiv) and *p*-toluenesulfonamide (41.0 mg, 0.24 mmol, 1.2 equiv) the title compound was prepared following the general procedure **A** using Ni(COD)$_2$ (5.5 mg, 10 mol%), PCy$_3$ (11.2 mg, 20 mol%), Ti(O^{i}Pr)$_4$ (12 μL, 20 mol%), anhydrous acetonitrile (0.5 mL). The reaction mixture was stirred for 12 h at 100 °C. The crude material was purified by flash column chromatography (petroleum ether/EtOAc = 5:1) to provide the title compound as a white solid in 52% yield with a mixture containing diastereoisomers. (1:1 dr, 43.6 mg). m.p.: 92−94 °C.

^{1}H NMR (400 MHz, CDCl$_3$) δ 7.72 (d, *J* = 8.0 Hz, 2H), 7.26 – 7.14 (m, 5H), 7.11 – 7.05 (m, 2H), 6.19 (dd, *J* = 15.9, 6.2 Hz, 1H), 5.71 – 5.58 (m, 1H), 5.03 (q, *J* = 7.5 Hz, 1H), 4.67 (dd, *J* = 25.1, 7.8 Hz, 1H), 4.00 (p, *J* = 7.3 Hz, 1H), 2.27 (s, 3H), 1.91 (ddd, *J* = 24.4, 15.7, 8.1 Hz, 2H), 1.65 (d, *J* = 10.2 Hz, 3H), 1.57 (d, *J* = 8.7 Hz, 3H), 1.50 –

1.18 (m, 4H), 1.17 – 1.08 (m, 1H), 0.85 (dd, *J* = 6.3, 3.3 Hz, 3H).
^{13}C NMR (101 MHz, $CDCl_3$) δ 143.1, 138.2, 136.3, 136.2, 131.6, 131.4, 131.3, 131.0, 129.4, 129.4, 129.4, 128.7, 128.3, 127.6, 127.5, 127.3, 127.3, 126.3, 126.3, 124.4, 54.7, 54.4, 43.3, 43.2, 36.9, 36.7, 28.9, 28.6, 25.7, 25.6, 25.2, 21.3, 19.3, 19.2, 17.6, 17.6.
HRMS (ESI) calcd. for $[C_{25}H_{33}NO_2S, M\text{-}H]^-$: 410.2159, found: 410.2155.

(*E*)-N-(1-(4-(4-hydroxy-4-methylpentyl)cyclohex-3-en-1-yl)-3-phenylallyl)-4-methylbenzenesulfonamide (61)

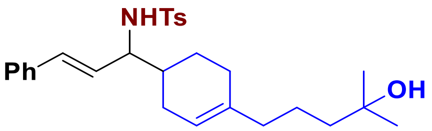

From styrene (35 μL, 0.30 mmol, 1.5 equiv), lyral (42 mg, 0.20 mmol, 1.0 equiv) and *p*-toluenesulfonamide (41.0 mg, 0.24 mmol, 1.2 equiv) the title compound was prepared following the general procedure **A** using $Ni(COD)_2$ (5.5 mg, 10 mol%), PCy_3 (11.2 mg, 20 mol%), $Ti(O^iPr)_4$ (12 μL, 20 mol%), anhydrous EtOH (0.5 mL). The reaction mixture was stirred for 12 h at 100 °C. The crude material was purified by flash column chromatography (petroleum ether/EtOAc = 2:1) to provide the title compound as a white solid in 67% yield with a mixture containing diastereoisomers. (1:1 dr, 62.5 mg). m.p.: 122−123 °C.
^{1}H NMR (400 MHz, $CDCl_3$) δ 7.73 – 7.67 (m, 2H), 7.26 – 7.18 (m, 3H), 7.14 (dd, *J* = 8.2, 2.1 Hz, 2H), 7.06 (ddt, *J* = 8.0, 5.7, 1.6 Hz, 2H), 6.12 – 6.01 (m, 1H), 5.68 (ddd, *J* = 15.8, 8.2, 6.0 Hz, 1H), 5.35 (d, *J* = 18.4 Hz, 1H), 4.63 (dd, *J* = 8.5, 6.3 Hz, 1H), 3.79 (q, *J* = 8.1, 7.6 Hz, 1H), 2.25 (d, *J* = 5.2 Hz, 3H), 2.18 – 1.67 (m, 9H), 1.41 – 1.21 (m, 4H), 1.21 (s, 6H).
^{13}C NMR (101 MHz, $CDCl_3$) δ 143.1, 138.1, 137.7, 137.4, 136.2, 132.4, 132.1, 132.1, 129.4, 128.3, 127.6, 127.3, 127.1, 127.0, 126.3, 120.8, 120.6, 119.6, 119.4, 70.9, 60.9, 60.7, 60.4, 43.5, 43.4, 38.9, 38.0, 37.8, 31.0, 29.3, 29.2, 29.2, 29.1, 28.0, 28.0, 27.8, 25.2, 25.1, 24.7, 24.7, 22.4, 22.3, 22.3, 21.3.
HRMS (ESI) calcd. for $[C_{28}H_{37}NO_3S, M\text{-}H]^-$: 466.2421, found: 466.2420.

N-((6*S*, *E*)-6-((3*S*, 5*R*, 7*R*, 8*S*, 9*R*, 10*R*, 13*S*, 14*R*, 17*S*)-3,7-dihydroxy-10,13-dimethylhexadecahydro-1H-cyclopenta[a]phenanthren-17-yl)-1-phenylhept-1-en-3-yl)-4-methylbenzenesulfonamide (62)

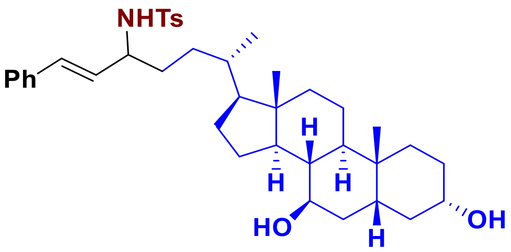

From styrene (46 μL, 0.40 mmol, 2.0 equiv), (S)-4-((3*S*, 5*R*, 7*R*, 8*S*, 9*R*, 10*R*, 13*S*, 14*R*, 17*S*)-3,7-dihydroxy-10,13-dimethylhexadecahydro-1H-cyclopenta[a]phenanthren-17-

yl)pentanal (75.2 mg, 0.20 mmol, 1.0 equiv) and *p*-toluenesulfonamide (41.0 mg, 0.24 mmol, 1.2 equiv) the title compound was prepared following the general procedure **A** using $Ni(COD)_2$ (5.5 mg, 10 mol%), PCy_3 (11.2 mg, 20 mol%), $Ti(O^iPr)_4$ (60 μL, 100 mol%), anhydrous EtOH (0.5 mL). The reaction mixture was stirred for 12 h at 100 °C. The crude material was purified by flash column chromatography (petroleum ether/acetone = 2:1) to provide the title compound as a white solid in 39% yield with a mixture containing diastereoisomers. (1:1 dr, 49.3 mg). m.p.: 188−189 °C.

^{1}H NMR (400 MHz, $CDCl_3$) δ 7.78 – 7.71 (m, 2H), 7.28 – 7.20 (m, 3H), 7.18 (dd, *J* = 8.3, 2.9 Hz, 2H), 7.09 (tt, *J* = 7.8, 1.5 Hz, 2H), 6.19 (dd, *J* = 15.8, 8.3 Hz, 1H), 5.70 (ddd, *J* = 15.8, 7.6, 1.5 Hz, 1H), 5.11 (dd, *J* = 58.7, 8.2 Hz, 1H), 3.88 (h, *J* = 7.5 Hz, 1H), 3.59 (tt, *J* = 10.8, 4.8 Hz, 2H), 2.29 (d, *J* = 6.3 Hz, 3H), 2.01 – 1.96 (m, 1H), 1.85 – 1.75 (m, 5H), 1.71 – 1.57 (m, 6H), 1.53 – 1.38 (m, 8H), 1.21 (tdd, *J* = 18.8, 9.4, 4.8 Hz, 5H), 1.07 – 0.98 (m, 3H), 0.95 (s, 3H), 0.89 (dd, *J* = 12.1, 6.5 Hz, 3H), 0.65 (s, 3H).

^{13}C NMR (101 MHz, $CDCl_3$) δ 143.1, 143.1, 138.3, 138.2, 136.3, 136.3, 131.5, 131.0, 129.6, 129.4, 129.2, 128.8, 128.3, 128.3, 127.6, 127.5, 127.2, 126.4, 126.3, 126.3, 71.4, 71.3, 71.2, 56.8, 56.8, 55.8, 55.7, 54.9, 54.8, 43.7, 43.7, 42.4, 42.4, 39.3, 39.2, 36.9, 35.2, 34.9, 34.0, 32.6, 32.5, 31.7, 31.6, 30.3, 28.7, 28.6, 26.9, 26.9, 23.4, 21.5, 21.4, 21.1, 21.1, 18.7, 18.7, 12.1, 12.1.

HRMS (ESI) calcd. for $[C_{39}H_{55}NO_4S, M-H]^-$: 632.3779, found: 632.3776.

***tert*-butyl-(*E*)-4-(3-(11-(1-(ethoxycarbonyl)piperidin-4-ylidene)-6,11-dihydro-5H-benzo[5,6]cyclohepta[1,2-b]pyridin-8-yl)-1-((4-methylphenyl)sulfonamido)allyl)piperidine-1-carboxylate (63)**

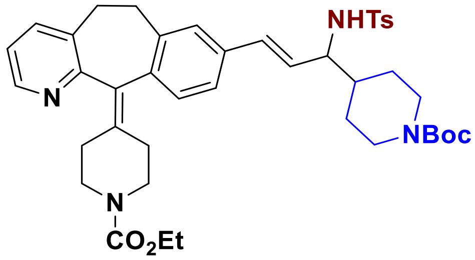

From ethyl-4-(8-vinyl-5,6-dihydro-11H-benzo[5,6]cyclohepta[1,2-b]pyridin-11-ylidene)piperidine-1-carboxylate (149.6 mg, 0.40 mmol, 2.0 equiv), *tert*-butyl-4-formylpiperidine-1-carboxylate (42.6 mg, 0.20 mmol, 1.0 equiv) and *p*-toluenesulfonamide (41.0 mg, 0.24 mmol, 1.2 equiv) the title compound was prepared following the general procedure **A** using $Ni(COD)_2$ (5.5 mg, 10 mol%), PCy_3 (11.2 mg, 20 mol%), $Ti(O^iPr)_4$ (60 μL, 100 mol%), anhydrous acetonitrile (0.5 mL). The reaction mixture was stirred for 12 h at 100 °C. The crude material was purified by flash column chromatography (petroleum ether/acetone = 2:1) to provide the title compound as a white solid in 67% yield (99.1 mg). m.p.: 161−163 °C.

^{1}H NMR (400 MHz, $CDCl_3$) δ 8.40 (dd, *J* = 4.8, 1.6 Hz, 1H), 7.73 – 7.62 (m, 2H), 7.45 (dd, *J* = 7.7, 1.7 Hz, 1H), 7.15 – 7.03 (m, 4H), 6.91 – 6.76 (m, 2H), 5.97 (d, *J* = 15.6 Hz, 1H), 5.65 (dd, *J* = 15.4, 8.0 Hz, 1H), 5.23 (d, *J* = 8.5 Hz, 1H), 4.14 (q, *J* = 7.1 Hz, 4H), 3.83 (s, 2H), 3.68 (s, 1H), 3.43 – 3.27 (m, 2H), 3.22 – 3.07 (m, 2H), 2.88 – 2.70

(m, 2H), 2.65 – 2.41 (m, 3H), 2.39 – 2.24 (m, 3H), 2.17 (s, 3H), 1.78 (d, *J* = 13.0 Hz, 1H), 1.58 (d, *J* = 11.4 Hz, 2H), 1.43 (s, 9H), 1.25 (t, *J* = 7.1 Hz, 3H), 1.20 – 1.05 (m, 2H).

^{13}C NMR (101 MHz, $CDCl_3$) δ 157.4, 155.5, 154.6, 146.6, 143.1, 138.7, 137.9, 137.9, 137.6, 137.3, 137.3, 136.8, 135.1, 134.9, 133.5, 132.3, 129.4, 129.4, 127.2, 127.2, 126.9, 126.1, 124.0, 123.8, 122.1, 79.4, 61.3, 60.8, 44.8, 43.4, 41.3, 31.8, 31.6, 30.7, 30.5, 28.4, 28.1, 21.2, 14.7.

HRMS (ESI) calcd. for $[C_{42}H_{52}N_4O_6S, M\text{-}H]^-$: 739.3535, found: 739.3530.

***tert*-butyl-4-((*E*)-3-(4-((((1*R*, 2*S*, 5*R*)-2-isopropyl-5-methylcyclohexyl)oxy)methyl)phenyl)-1-((4-methylphenyl)sulfonamido)allyl)piperidine-1-carboxylate (64)**

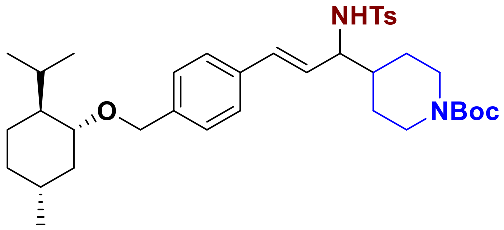

From 1-((((1*R*, 2*S*, 5*R*)-2-isopropyl-5-methylcyclohexyl)oxy)methyl)-4-vinylbenzene (108.8 mg, 0.40 mmol, 2.0 equiv), *tert*-butyl-4-formylpiperidine-1-carboxylate (42.6 mg, 0.20 mmol, 1.0 equiv) and *p*-toluenesulfonamide (41.0 mg, 0.24 mmol, 1.2 equiv) the title compound was prepared following the general procedure **A** using $Ni(COD)_2$ (5.5 mg, 10 mol%), PCy_3 (11.2 mg, 20 mol%), $Ti(O^iPr)_4$ (60 μL, 100 mol%), anhydrous acetonitrile (0.5 mL). The reaction mixture was stirred for 12 h at 100 °C. The crude material was purified by flash column chromatography (petroleum ether/acetone = 2:1) to provide the title compound as a white solid in 65% yield with a mixture containing diastereoisomers. (1:1 dr, 82.9 mg). m.p.: 132−133 °C.

^{1}H NMR (400 MHz, $CDCl_3$) δ 7.72 (d, *J* = 7.9 Hz, 2H), 7.19 (dd, *J* = 26.6, 7.9 Hz, 4H), 7.02 (d, *J* = 7.7 Hz, 2H), 6.00 (d, *J* = 15.8 Hz, 1H), 5.67 (dd, *J* = 15.8, 8.3 Hz, 1H), 5.35 (d, *J* = 8.4 Hz, 1H), 4.63 (d, *J* = 11.7 Hz, 1H), 4.36 (d, *J* = 11.7 Hz, 1H), 4.10 (s, 2H), 3.71 (t, *J* = 8.4 Hz, 1H), 3.17 (td, *J* = 10.6, 4.1 Hz, 1H), 2.63 (d, *J* = 19.4 Hz, 2H), 2.27 (s, 4H), 2.19 (t, *J* = 6.4 Hz, 1H), 1.83 (d, *J* = 12.5 Hz, 1H), 1.69 – 1.57 (m, 4H), 1.45 (s, 9H), 1.38 – 1.33 (m, 1H), 1.18 (dt, *J* = 12.0, 6.0 Hz, 2H), 0.93 (dq, *J* = 14.3, 7.5, 6.2 Hz, 10H), 0.73 (d, *J* = 6.9 Hz, 3H).

^{13}C NMR (101 MHz, $CDCl_3$) δ 154.6, 143.2, 138.8, 137.9, 135.1, 132.5, 129.5, 127.8, 127.2, 126.2, 125.9, 79.4, 78.8, 70.0, 60.9, 48.3, 43.6, 41.3, 40.3, 34.5, 31.5, 29.3, 28.4, 28.2, 25.5, 23.2, 22.4, 21.3, 21.0, 16.1.

HRMS (ESI) calcd. for $[C_{37}H_{54}N_2O_5S, M\text{-}H]^-$: 637.3680, found: 637.3678.

4. Synthetic Applications:

(*E*)-N-(3-(3,5-dichlorophenyl)allyl)methanesulfonamide (65)

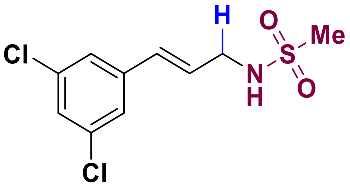

From 1,3-dichloro-5-vinylbenzene (1.2 g, 7 mmol, 1.0 equiv), paraformaldehyde (0.42 g, 14 mmol, 2.0 equiv) and methanesulfonamide (1.33 g, 14 mmol, 2.0 equiv) the title compound was prepared following the general procedure **B** using $Ni(OAc)_2$ (123.2 mg, 10 mol%), PCy_3 (392 mg, 20 mol%),Mn (115.5 mg, 2.1 mmol, 0.3 equiv), $Ti(O^iPr)_4$ (2 mL, 100 mol%), anhydrous EtOH (15 mL). The reaction mixture was stirred for 16 h at 100 °C. The crude material was purified by flash column chromatography (dichloromethane as eluent) to provide the title compound as a white solid in 53% yield (1.03 g). m.p.: 63−64 °C.

^{1}H NMR (400 MHz, $CDCl_3$) δ 7.29 – 7.20 (m, 3H), 6.51 (dd, J = 15.8, 1.5 Hz, 1H), 6.25 (dt, J = 15.8, 6.1 Hz, 1H), 5.05 (t, J = 6.2 Hz, 1H), 3.94 (td, J = 6.2, 1.5 Hz, 2H), 3.02 (s, 3H).

^{13}C NMR (101 MHz, $CDCl_3$) δ 139.1, 135.2, 130.3, 127.8, 127.7, 124.8, 44.9, 41.0.

HRMS (ESI) calcd. for $[C_{10}H_{11}Cl_2NO_2S, M\text{-}H]^-$: 277.9815, found: 277.9812.

Methyl-(*E*)-2-(4-((N-(3-(3,5-dichlorophenyl)allyl)methylsulfonamido)methyl)phenyl)acetate (66)

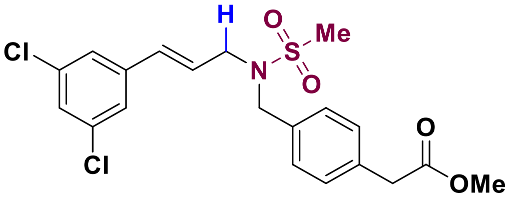

(*E*)-N-(3-(3,5-dichlorophenyl)allyl)methanesulfonamide (**65**) (55.7 mg, 0.2 mmol, 1.0 equiv) was added to a stirred suspension of NaH (9.6 mg, 0.4 mmol, 2.0 equiv) in DMF(1.0 mL) at ice bath, then warm room temperature to for 30 min. Then the residue was added to a stirred solution of methyl 2-(4-(bromomethyl)phenyl)acetate (96.7 mg , 0.4 mmol, 2.0 equiv) in DMF (1.0 mL) at ice bath, then warm room temperature to for other 30 min. The reaction mixture was quenched with sat. aq. NaCl solution, extracted by EtOAc (3 x 20.0 mL). The organic layer was dried ($MgSO_4$) and solvent evaporated in vacuo. The residue was purified by column chromatography (petroleum ether/EtOAc = 2:1) to provide the title compound as a white solid in 52% yield (46.1 mg). m.p.: 91−92 °C.

^{1}H NMR (400 MHz, $CDCl_3$) δ 7.27 – 7.19 (m, 4H), 7.17 (d, *J* = 1.8 Hz, 1H), 7.10 (d, *J* = 1.8 Hz, 2H), 6.29 (d, *J* = 15.8 Hz, 1H), 6.06 (dt, *J* = 15.8, 6.6 Hz, 1H), 4.35 (s, 2H), 3.88 (d, *J* = 6.6 Hz, 2H), 3.64 (s, 3H), 3.57 (s, 2H), 2.84 (s, 3H).

^{13}C NMR (101 MHz, $CDCl_3$) δ 171.7, 139.0, 135.2, 134.5, 134.0, 131.8, 129.7, 128.7, 127.8, 126.7, 124.8, 52.1, 50.2, 48.6, 40.7, 40.0.
HRMS (ESI) calcd. for [$C_{20}H_{21}Cl_2NO_4S$, M+Na]$^+$: 464.0466, found: 464.0465.

(*E*)-2-(4-((N-(3-(3,5-dichlorophenyl)allyl)methylsulfonamido)methyl)phenyl)acetic acid (67)[3]

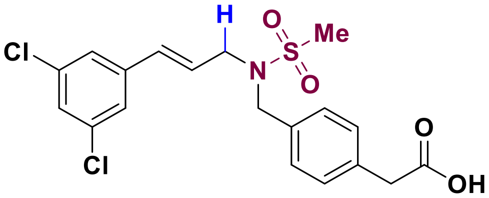

Lithium hydroxide (9.6 mg, 0.4 mmol, 4.0 equiv) was added to a stirred mixture of methyl-(*E*)-2-(4-((N-(3-(3,5-dichlorophenyl)allyl)methylsulfonamido)methyl)phenyl)acetate (**66**) (42.7 mg, 0.1 mmol, 1.0 euiqv) in a 1:1 mixture of THF and H_2O (1.0 mL). By small aliquots of HCl(aq) 6N the pH was brought to 2.0 and the volatiles evaporated. The crude was washed by H_2O and extracted by EtOAc (3 x 20.0 mL). The organic layer was dried ($MgSO_4$) and solvent evaporated in vacuo. No need further purification, the title compound as a white solid in 94% yield (40.1 mg).
^{1}H NMR (400 MHz, $CDCl_3$) δ 7.35 – 7.21 (m, 6H), 7.15 (s, 1H), 6.34 (d, J = 15.8 Hz, 1H), 6.11 (dt, J = 15.3, 6.7 Hz, 1H), 4.41 (s, 2H), 3.94 (d, J = 6.9 Hz, 3H), 3.66 (s, 2H), 2.91 (s, 3H).
^{13}C NMR (101 MHz, $CDCl_3$) δ 177.0, 139.0, 135.2, 134.8, 133.2, 131.8, 129.8, 128.7, 127.8, 126.7, 124.8, 50.2, 48.6, 40.5, 39.9, 29.7.
HRMS (ESI) calcd. for [$C_{19}H_{19}Cl_2NO_4S$, M-H]$^-$: 426.0334, found: 426.0332.

5. Transformations of Products:

N-(1,3-diphenylpropyl)-4-methylbenzenesulfonamide (68)[4]

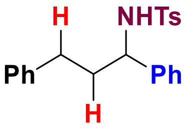

Add 10% palladium carbon into a round-bottomed flask, replace the gas with nitrogen three times, then replace the gas with hydrogen three times, then insert a hydrogen balloon, inject methanol as a solvent, and add (*E*)-N-(1,3-diphenylallyl)-4-methylbenzenesulfonamide (**17**) (36.3 mg, 0.10 mmol, 1.0 equiv) was stirred at room temperature for 4 h. After the reaction, suction filtration under reduced pressure and residue was purified by column chromatography (petroleum ether/EtOAc = 6:1) to provide the title compound as a white solid in 93% yield (33.9 mg).

^{1}H NMR (400 MHz, $CDCl_3$) δ 7.45 (d, *J* = 7.9 Hz, 2H), 7.17 – 6.89 (m, 12H), 5.50 (d, *J* = 7.9 Hz, 1H), 4.20 (q, *J* = 7.4 Hz, 1H), 2.53 – 2.34 (m, 2H), 1.96 (ddd, *J* = 48.0, 14.9, 7.3 Hz, 2H).

^{13}C NMR (101 MHz, $CDCl_3$) δ 142.9, 140.9, 140.6, 137.6, 129.3, 128.4, 128.4, 127.3, 127.0, 126.6, 125.9, 57.9, 39.0, 32.1, 21.4.

HRMS (ESI) calcd. for $[C_{22}H_{23}NO_2S, M+Na]^+$: 388.1347, found: 388.1345.

4-methyl-N-(phenyl(3-phenyloxiran-2-yl)methyl)benzenesulfonamide (69)

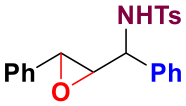

To a solution of (*E*)-N-(1,3-diphenylallyl)-4-methylbenzenesulfonamide **(13)** (72.6 mg, 0.20 mmol, 1.0 equiv) in CH_2Cl_2 (2.0 mL) was added $NaHCO_3$ (33.6 mg, 0.40 mmol, 2.0 equiv) and *m*-CPBA (68.8 mg, 0.40 mmol, 2.0 equiv) and the reaction was then stirred at rt for 12 h. The reaction mixture was washed with sat. aq. Na_2SO_3 solution (2 x 15 mL) and then sat. aq. Na_2CO_3 solution (2 x 15.0 mL). The organic layer was dried ($MgSO_4$) and solvent evaporated in vacuo. The residue was purified by column chromatography (petroleum ether/EtOAc = 4:1) to provide the title compound as a white solid in 83% yield (62.9 mg). m.p.: 128−129 °C.

^{1}H NMR (400 MHz, $CDCl_3$) δ 7.71 (dd, *J* = 12.9, 8.0 Hz, 2H), 7.35 (ddd, *J* = 16.5, 5.5, 2.8 Hz, 5H), 7.30 – 7.17 (m, 7H), 5.49 (dd, *J* = 14.9, 7.6 Hz, 1H), 4.90 – 4.50 (m, 1H), 3.87 (dd, *J* = 81.5, 2.0 Hz, 1H), 3.37 – 3.30 (m, 1H), 2.45 (d, *J* = 3.9 Hz, 3H).

^{13}C NMR (101 MHz, $CDCl_3$) δ 143.4, 143.3, 138.1, 137.7, 137.3, 136.5, 135.9, 135.9, 129.5, 129.4, 128.7, 128.4, 128.4, 128.3, 128.0, 127.3, 127.1, 127.0, 126.9, 125.7, 64.3, 63.6, 58.3, 57.7, 56.4, 55.7, 21.4.

HRMS (ESI) calcd. for $[C_{22}H_{21}NO_3S, M-H]^-$: 402.1134, found: 402.1140.

3-bromo-2,4-diphenyl-1-tosylazetidine (70)[5]

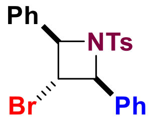

A solution of bis(collidine)bromonium(I) hexafluorophosphate (BBH) (140 mg, 0.30 mmol, 1.5 equiv) in dichloromethane (5 mL) was added at room temp. over 6 h to a solution of (*E*)-N-(1,3-diphenylallyl)-4-methylbenzenesulfonamide (**17**) (72.6 mg, 0.20 mmol, 1.0 equiv) in dichloromethane (5.0 mL). After complete addition, the mixture was stirred for 12 h. Silica gel was added and the solvent removed under reduced pressure. The residue was purified by column chromatography (petroleum ether/EtOAc = 4:1) to provide the title compound as a white solid in 57% yield (50.2 mg).

^{1}H NMR (400 MHz, $CDCl_3$) δ 7.65 – 7.59 (m, 2H), 7.55 – 7.49 (m, 4H), 7.45 – 7.36 (m, 6H), 7.31 (s, 2H), 5.11 (d, *J* = 6.8 Hz, 2H), 3.97 (t, *J* = 6.8 Hz, 1H), 2.46 (s, 3H).

^{13}C NMR (101 MHz, $CDCl_3$) δ 144.5, 137.4, 132.6, 129.6, 128.9, 128.8, 128.3, 126.4, 72.0, 47.6, 21.6.

HRMS (ESI) calcd. for $[C_{22}H_{20}BrNO_2S, M+Na]^+$: 464.2096, found: 464.0295.

(*E*)-1-cyclohexyl-3-phenylpro*p*-2-en-1-amine (71)

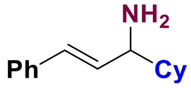

(*E*)-tert-butyl-(1-cyclohexyl-3-phenylallyl)carbamate (**12**) (63 mg, 0.20 mmol, 1.0 equiv) was added at room temp. to a solution of 4 M HCl in dioxane (3.0 mL), the mixture was stirred for 6 h. Adding NaOH until pH was brought to 12. The crude was washed by H_2O and extracted by EtOAc. The organic layer was dried ($MgSO_4$) and solvent evaporated in vacuo. The residue was purified by column chromatography (petroleum ether/EtOAc = 1:1, with Et_3N (2%)) to provide the title compound as a Colorless oil in 82% yield (35.3 mg).

^{1}H NMR (400 MHz, $CDCl_3$) δ 7.39 – 7.19 (m, 5H), 6.44 (d, *J* = 15.8 Hz, 1H), 6.16 (dd, *J* = 15.9, 7.6 Hz, 1H), 3.23 (t, *J* = 6.9 Hz, 1H), 1.88 – 1.63 (m, 5H), 1.35 – 0.95 (m, 6H).

^{13}C NMR (101 MHz, $CDCl_3$) δ 137.3, 133.8, 129.6, 128.5, 127.1, 126.2, 59.2, 44.2, 29.5, 29.3, 26.5, 26.3.

HRMS (ESI) calcd. for $[C_{15}H_{21}N, M-NH_2]^-$: 199.1487, found: 199.1485.

(*E*)-(3-azidopro*p*-1-ene-1,3-diyl)dibenzene (72)[6]

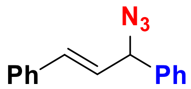

To a solution of (*E*)-N-(1,3-diphenylallyl)-4-methylbenzenesulfonamide (**17**) (36.3 mg, 0.1 mmol, 1.0 equiv) in DCM (0.2 mL) were added $TMSN_3$ (20 μL, 0.15 mmol, 1.5 equiv), TMSCl (2.2 mg, 0.02 mmol, 0.2 equiv), $ZnCl_2$ (1.4 mg, 0.01 mmol, 0.1 equiv), and H_2O (3.3 μL, 0.2 mmol, 2 equiv). The resulting mixture was stirred at room temperature for 0.5 h and then purified by flash column chromatography (n-hexane/EtOAc = 30:1) to provide the title compound as colorless liquid in 58% yield

(13.6 mg).

^{1}H NMR (400 MHz, $CDCl_3$) δ 7.43 – 7.36 (m, 6H), 7.36 – 7.30 (m, 3H), 7.29 – 7.25 (m, 1H), 6.72 (dd, *J* = 15.7, 1.1 Hz, 1H), 6.29 (dd, *J* = 15.7, 7.3 Hz, 1H), 5.21 (dd, *J* = 7.3, 1.2 Hz, 1H).

^{13}C NMR (101 MHz, $CDCl_3$) δ 138.5, 135.9, 132.9, 128.8, 128.6, 128.2, 128.2, 127.1, 126.9, 126.7, 67.2.

HRMS (ESI) calcd. for $[C_{15}H_{13}N_3, M\text{-}N_3]^+$: 193.1017, found: 193.1014.

6. NMR Spectra of New Compounds:

(*E*)-N-(1-cyclohexyl-3-phenylallyl)benzenesulfonamide (2)

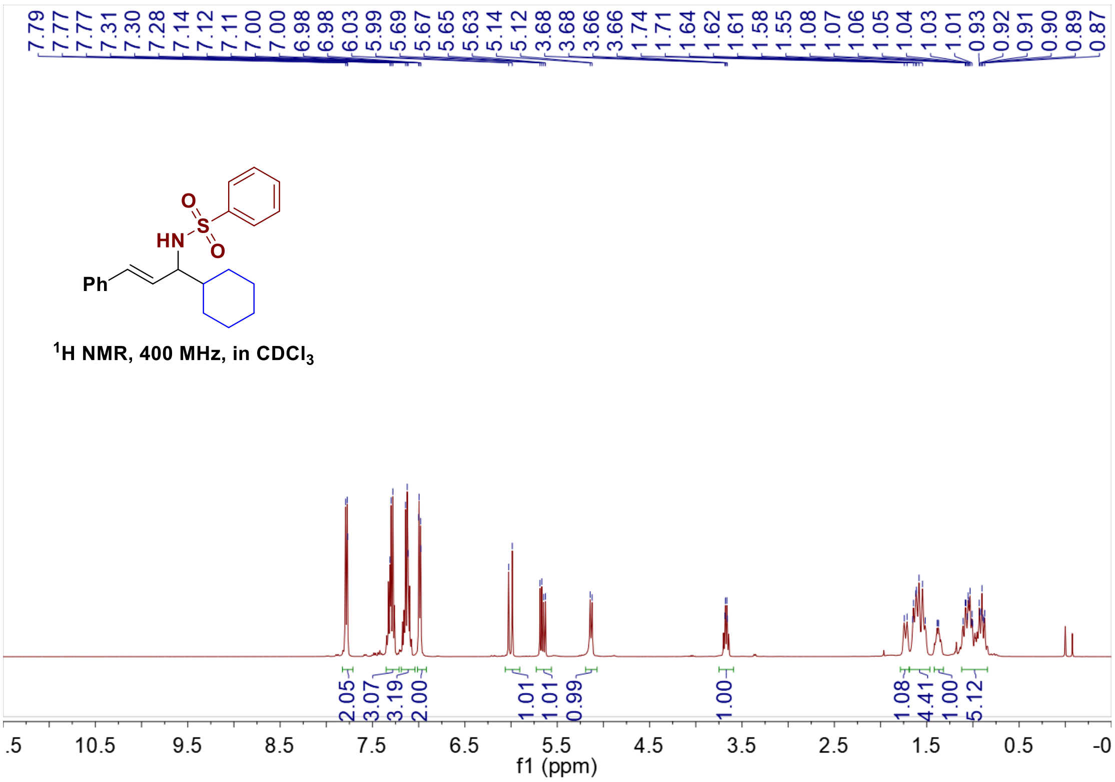

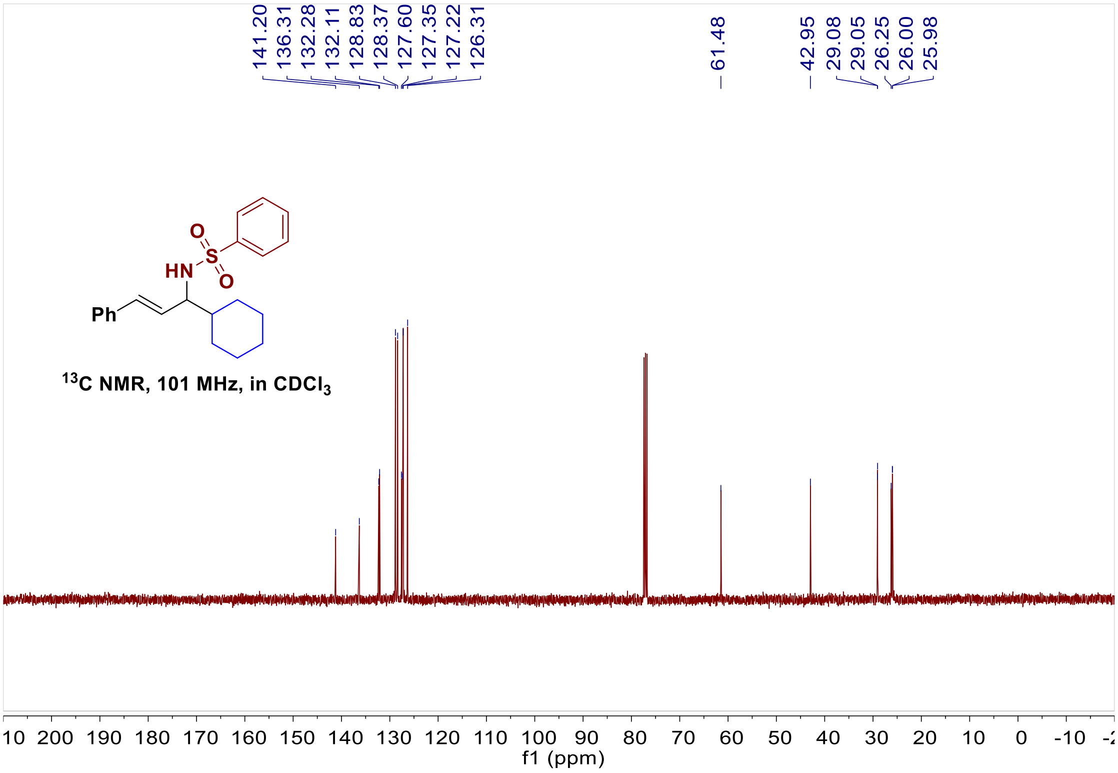

(*E*)-N-(1-cyclohexyl-3-phenylallyl)-4-methoxybenzenesulfonamide (3)

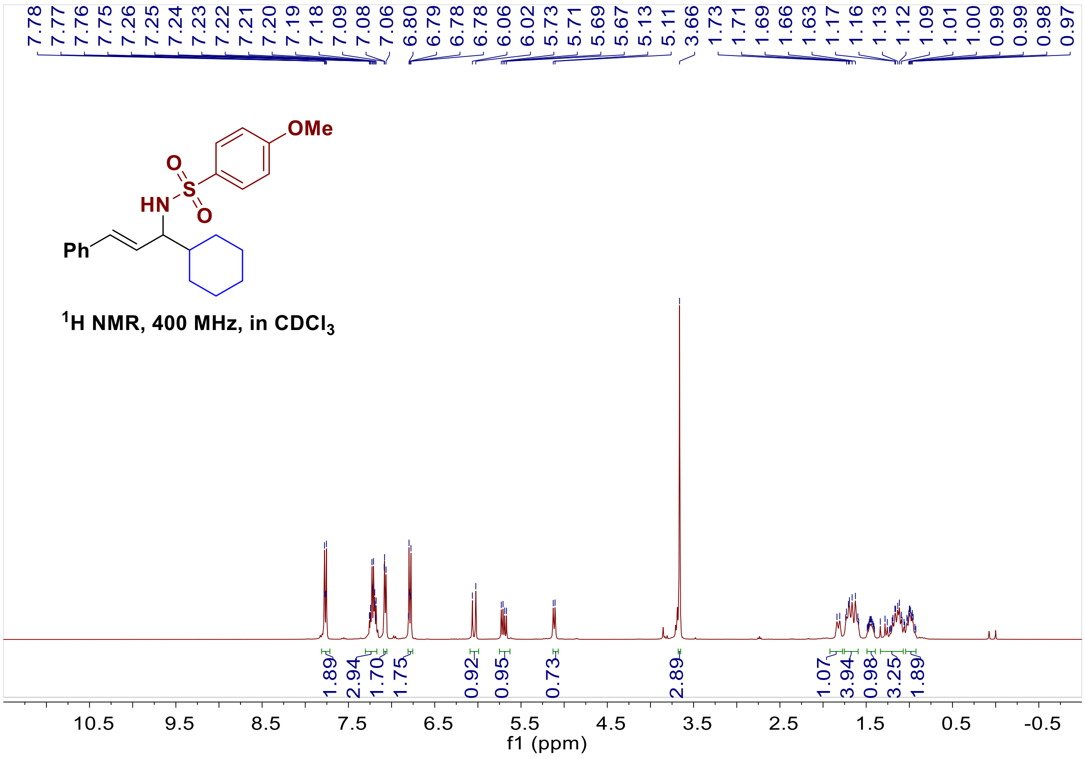

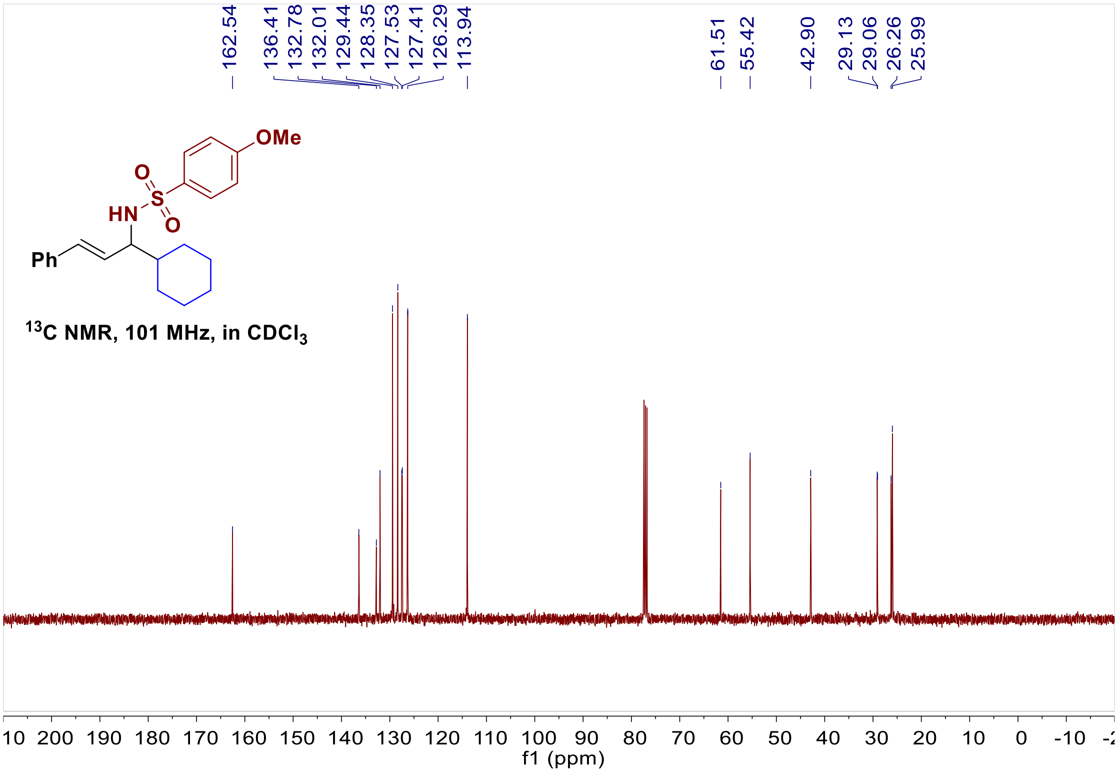

(*E*)-N-(1-cyclohexyl-3-phenylallyl)-4-(trifluoromethyl)benzenesulfonamide (4)

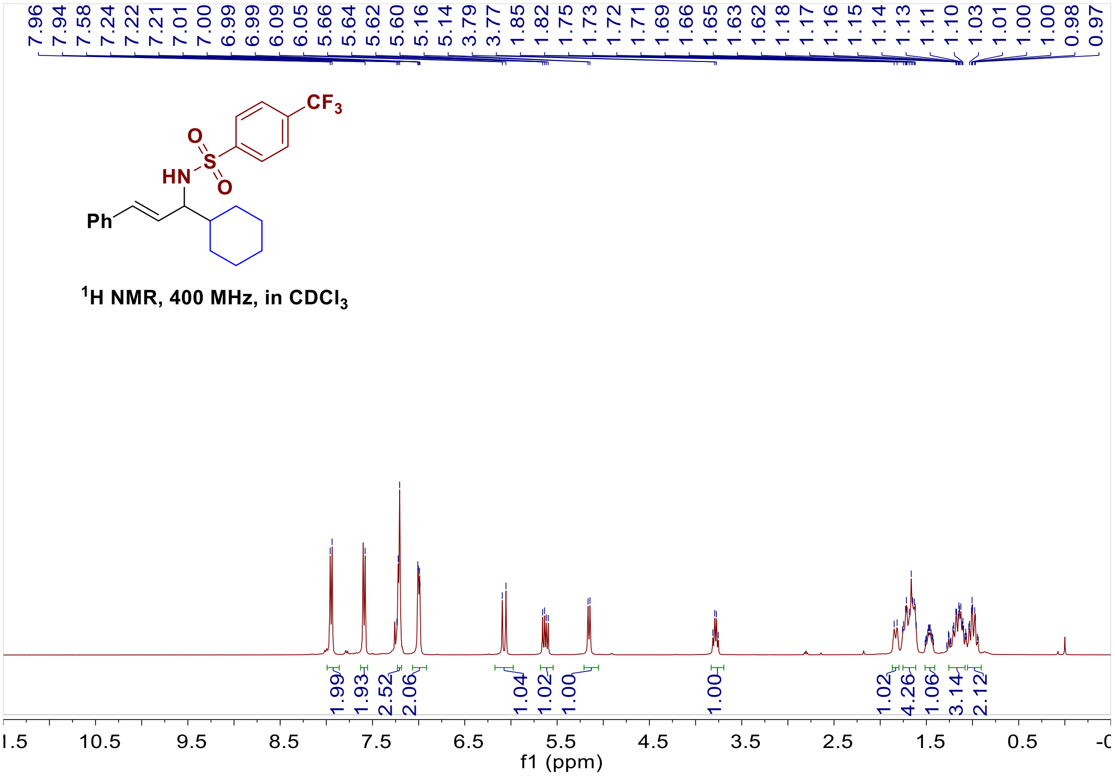

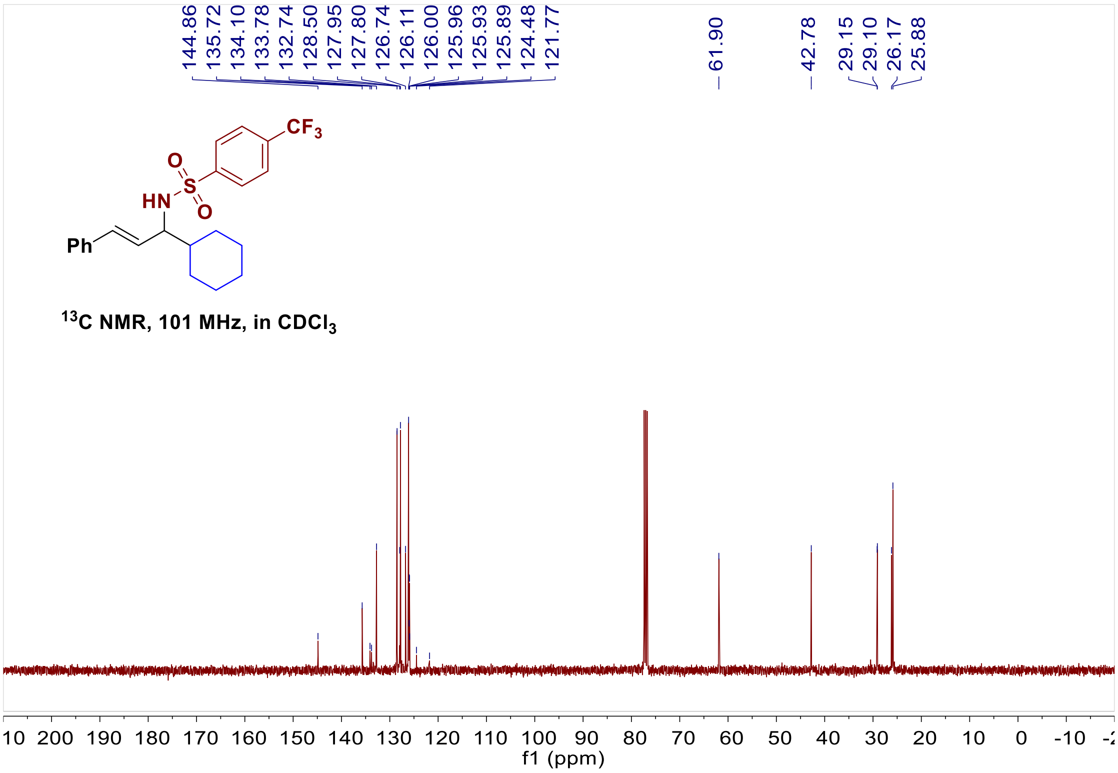

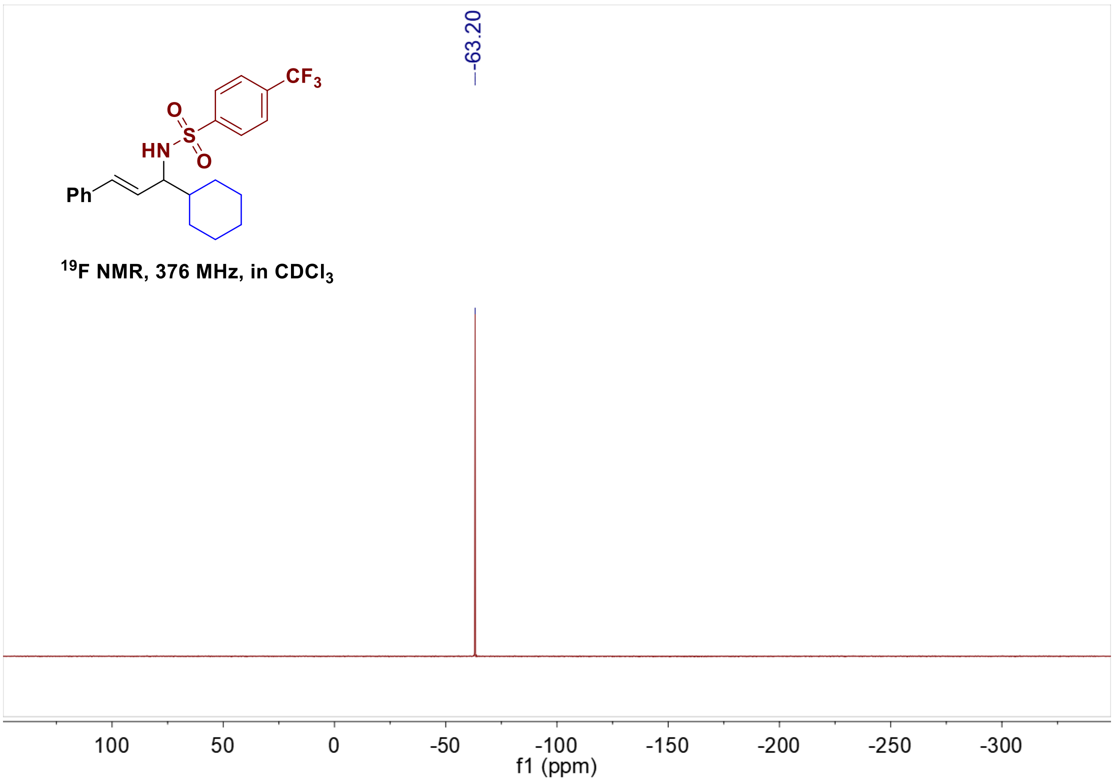

-63.20
CF3
O
S
O
HN
Ph
19F NMR, 376 MHz, in CDCl3
100
50
0
-50
-100
-150
-200
-250
-300
f1 (ppm)

(*E*)-N-(1-cyclohexyl-3-phenylallyl)thiophene-2-sulfonamide (5)

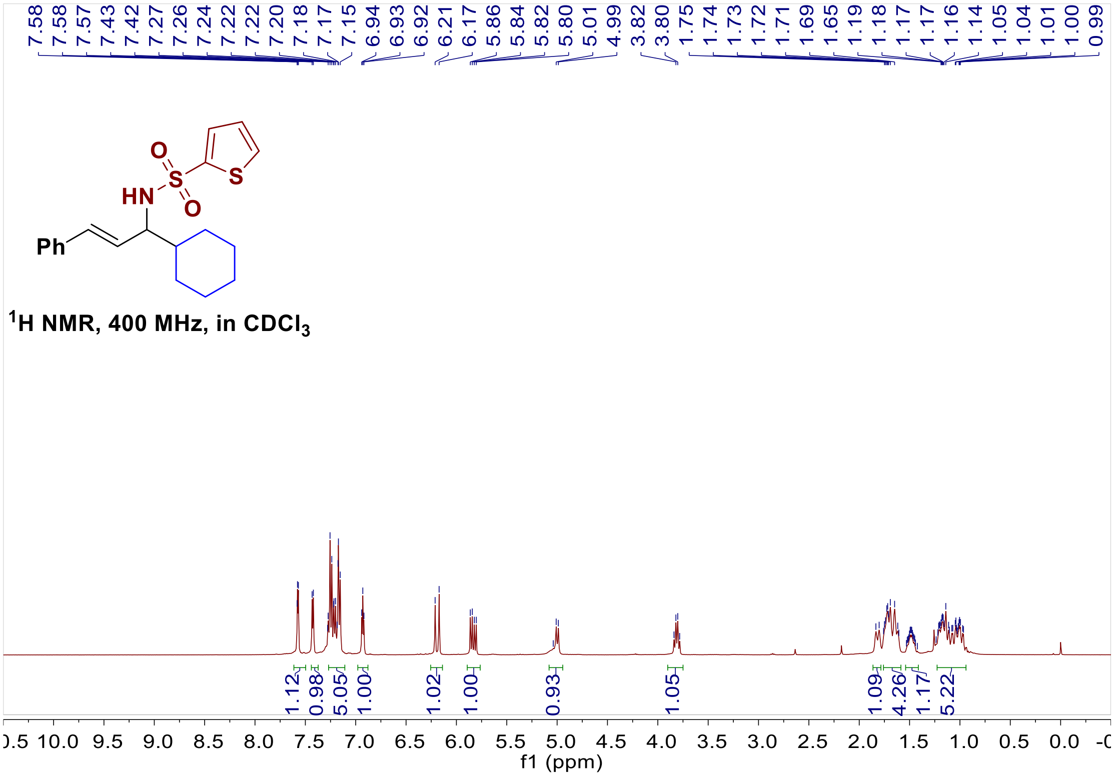

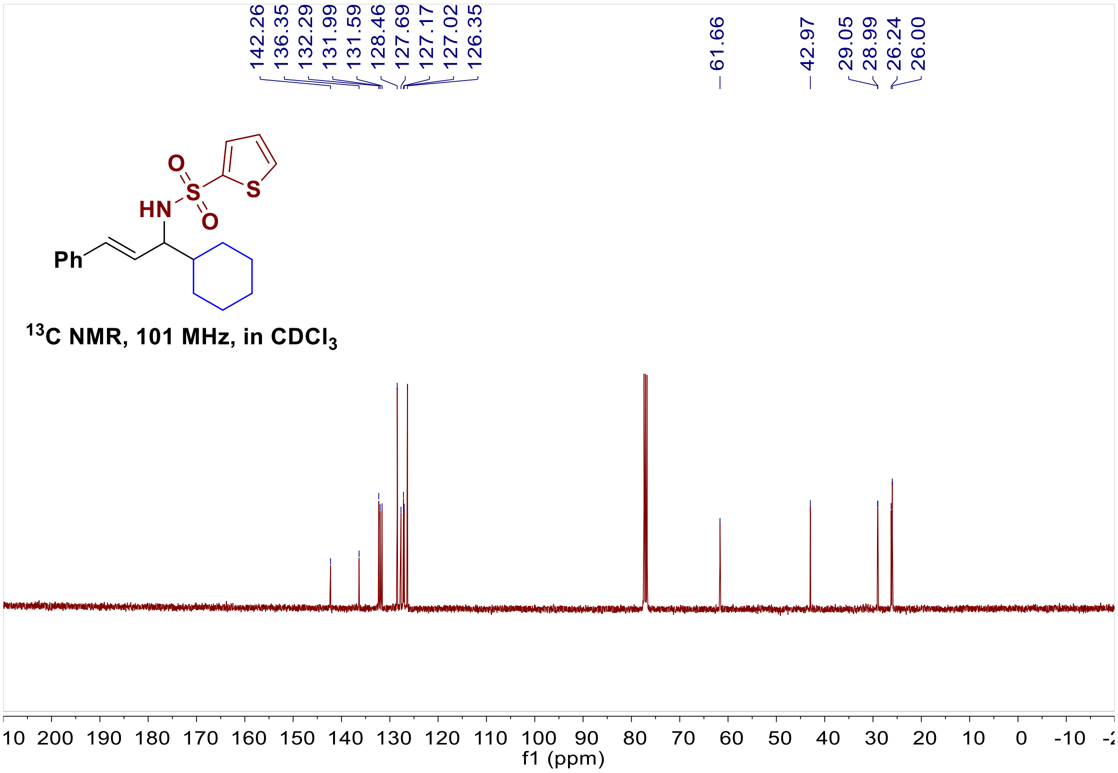

(*E*)-N-(1-cyclohexyl-3-phenylallyl)-1,1,1-trifluoromethanesulfonamide (6)

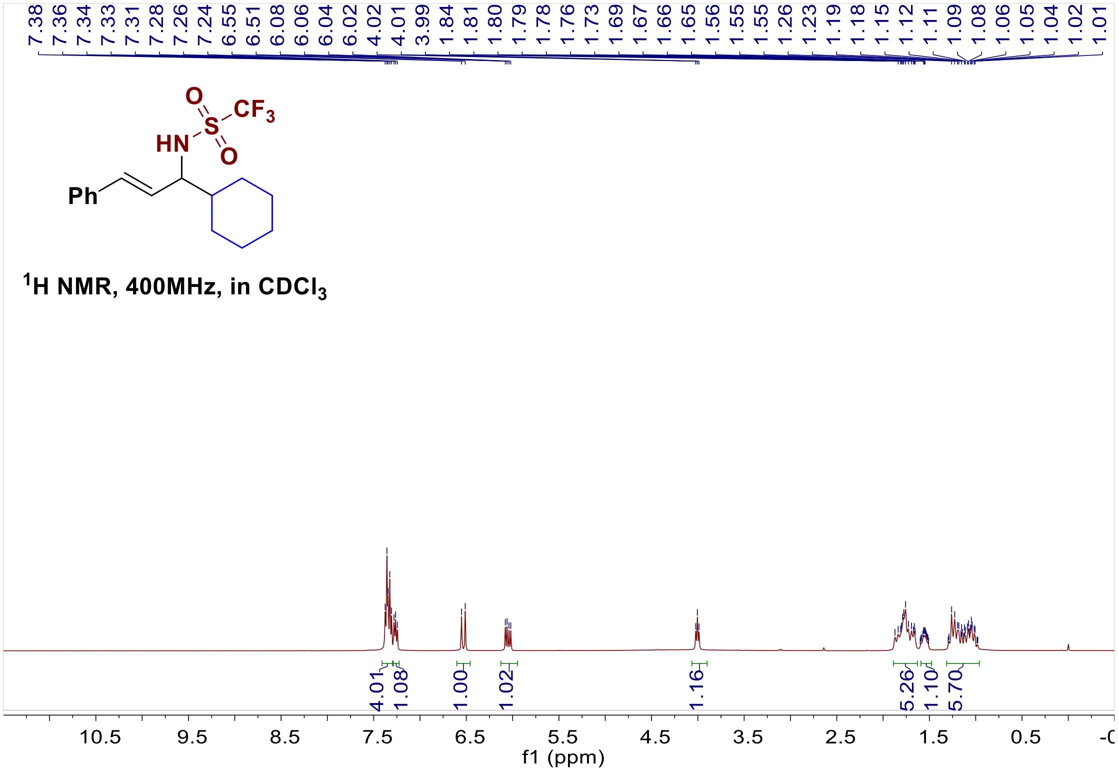

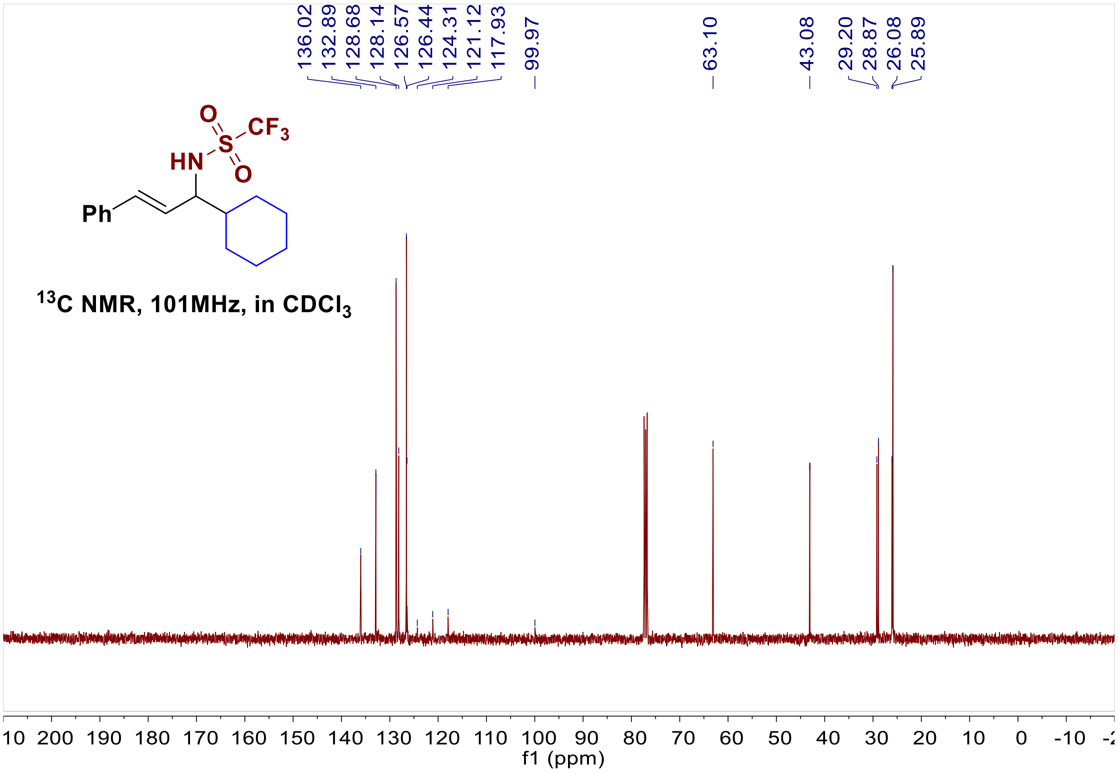

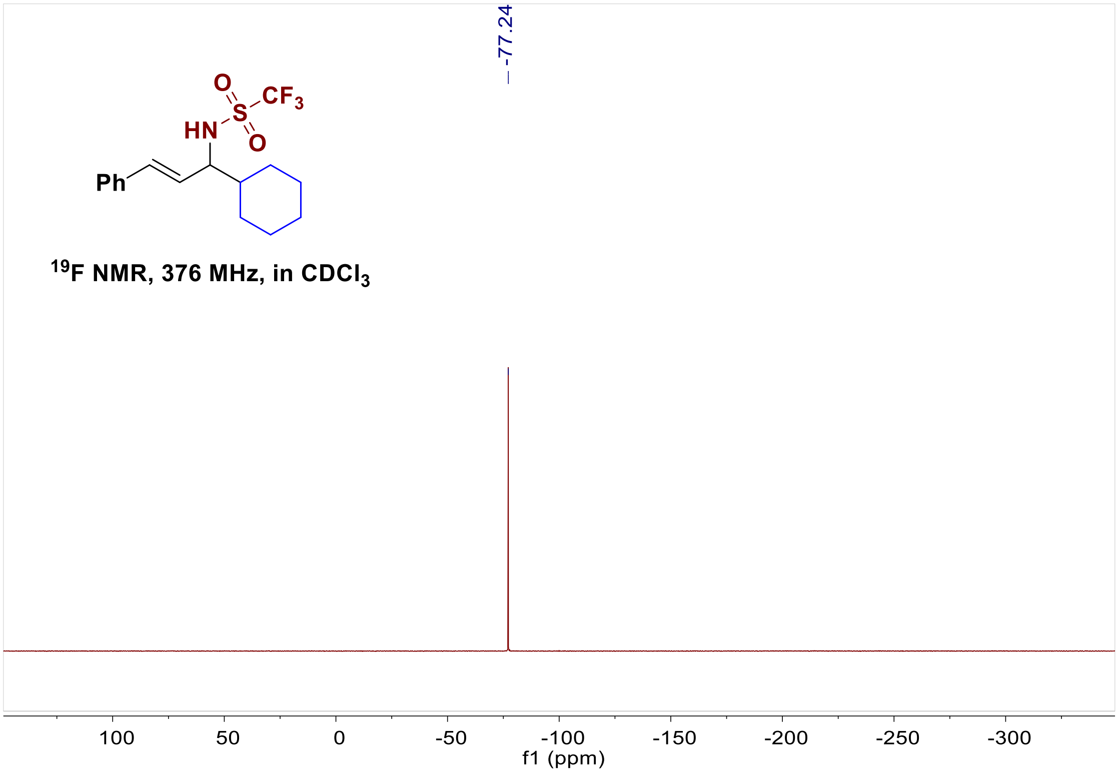

-77.24
O
CF3
HN
S
O
Ph
19F NMR, 376 MHz, in CDCl3
100
50
0
-50
-100
-150
-200
-250
-300
f1 (ppm)

(*E*)-N-(1-cyclohexyl-3-phenylallyl)methanesulfonamide (7)

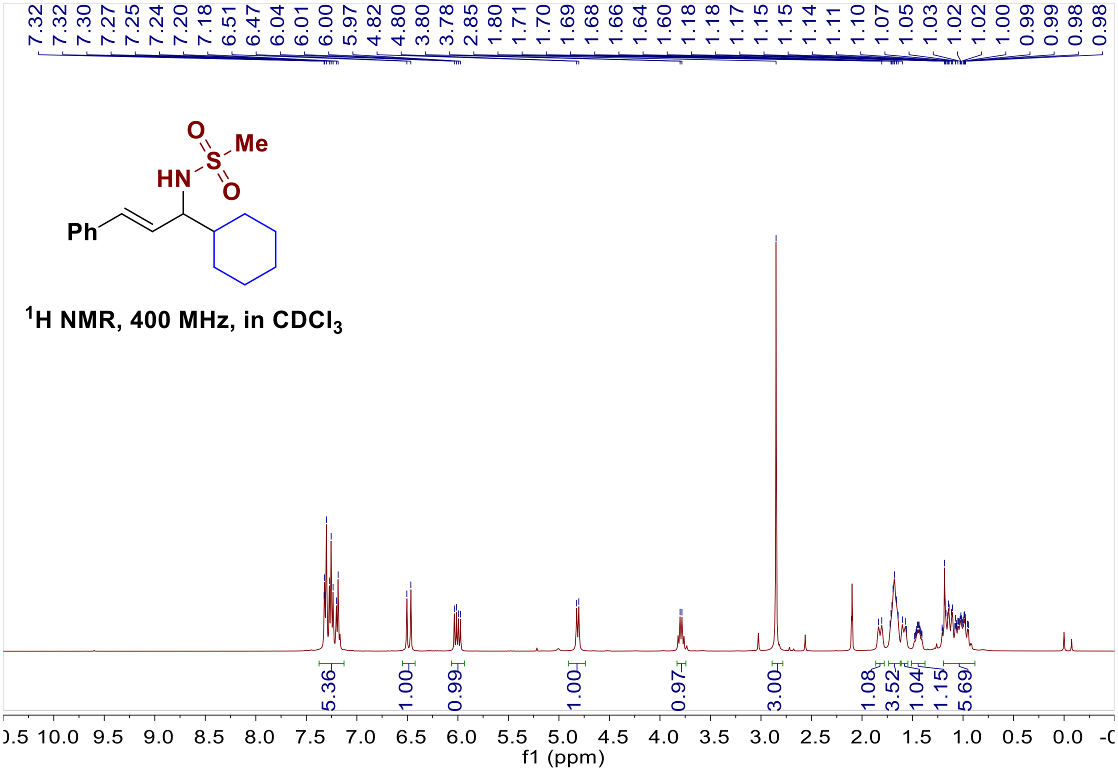

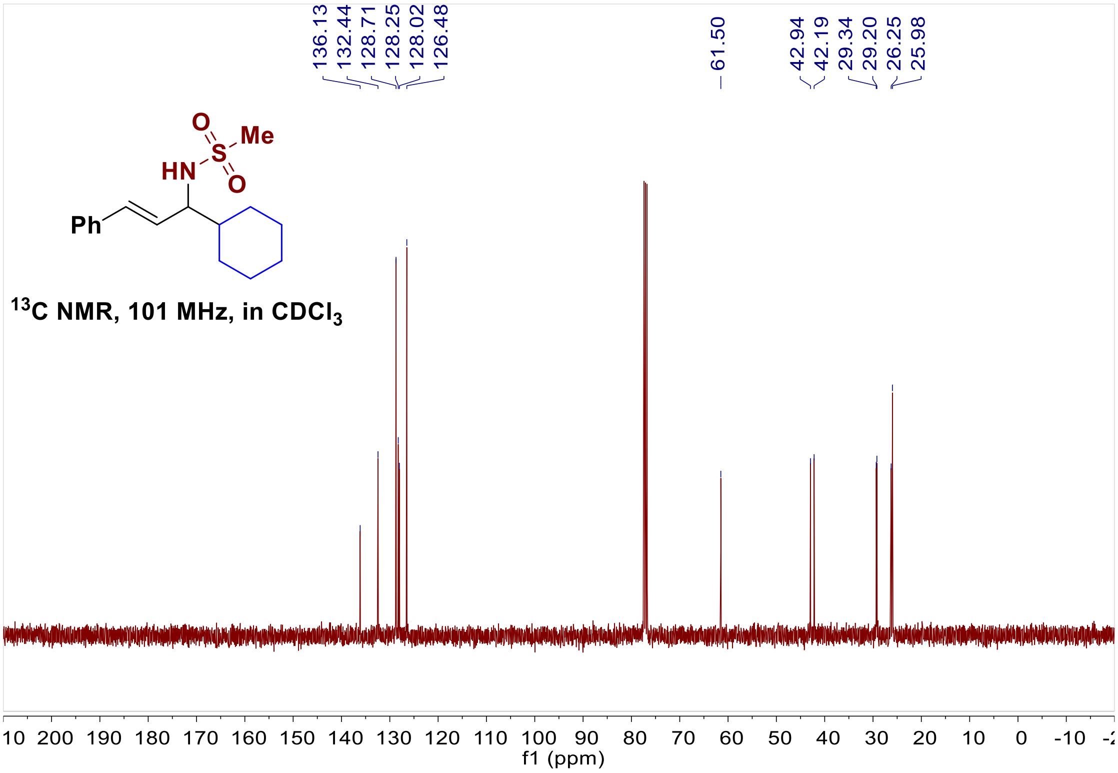

(*E*)-N-(1-cyclohexyl-3-phenylallyl))-N,N-dimethylsulfonamide (8)

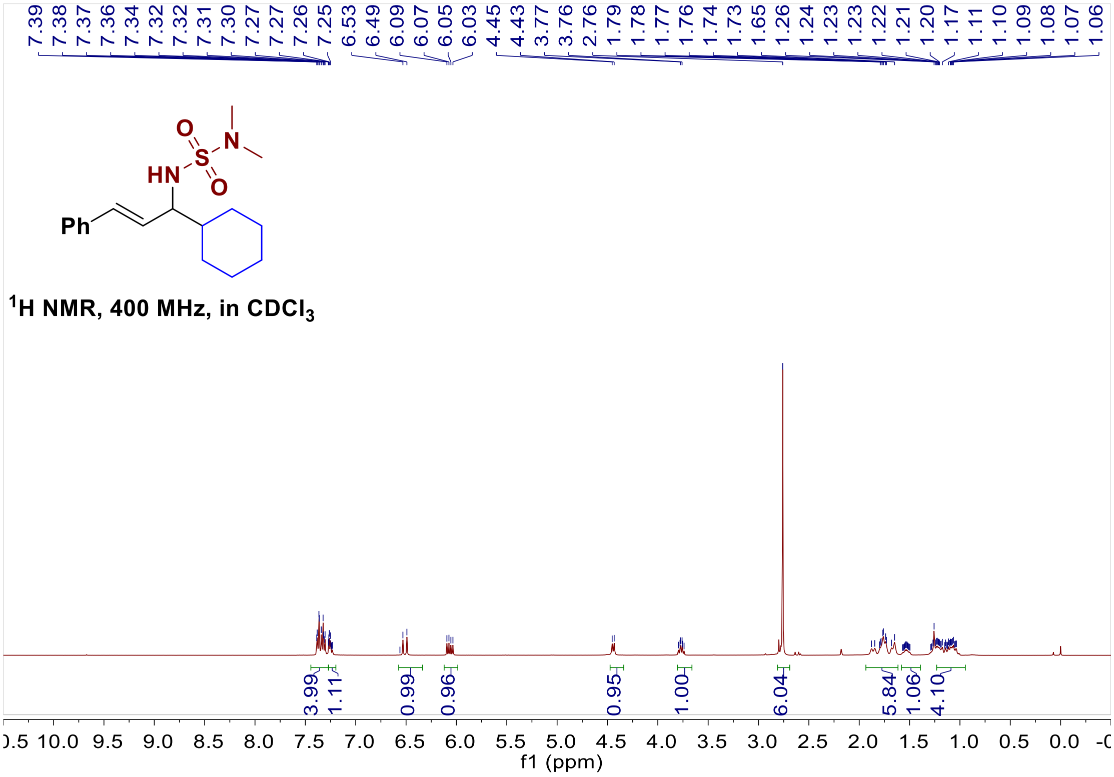

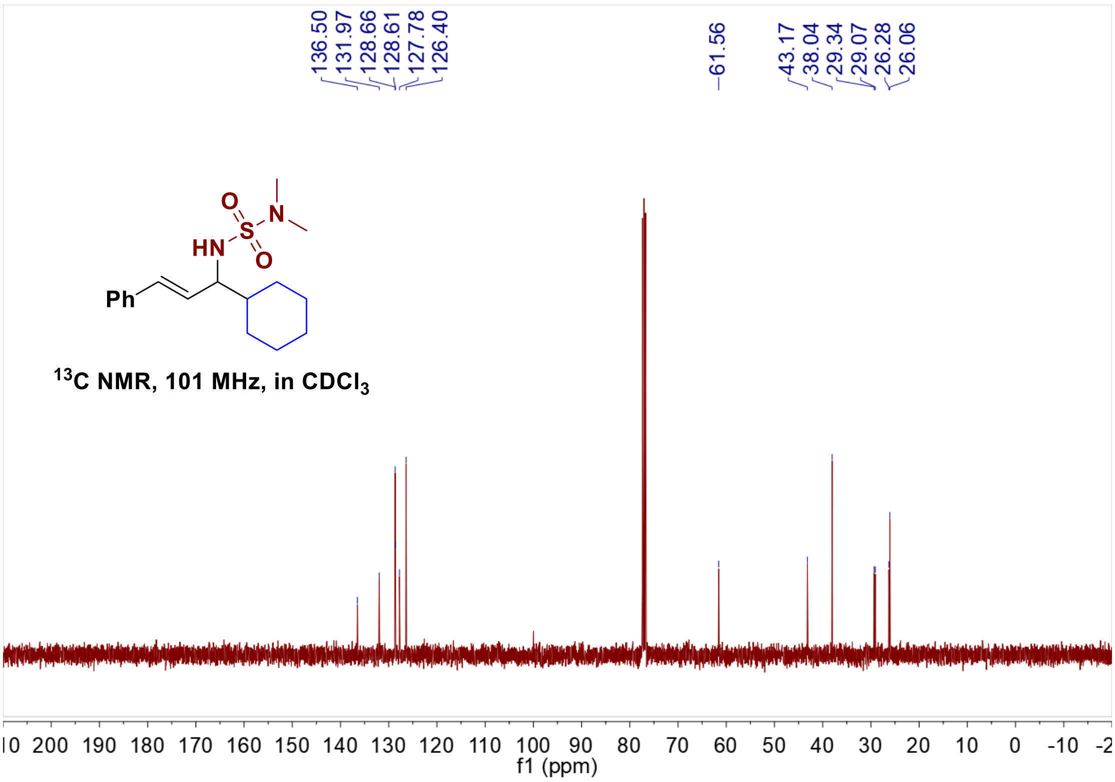

(*E*)-N-(1-cyclohexyl-3-phenylallyl)-2-methylpropane-2-sulfonamide (9)

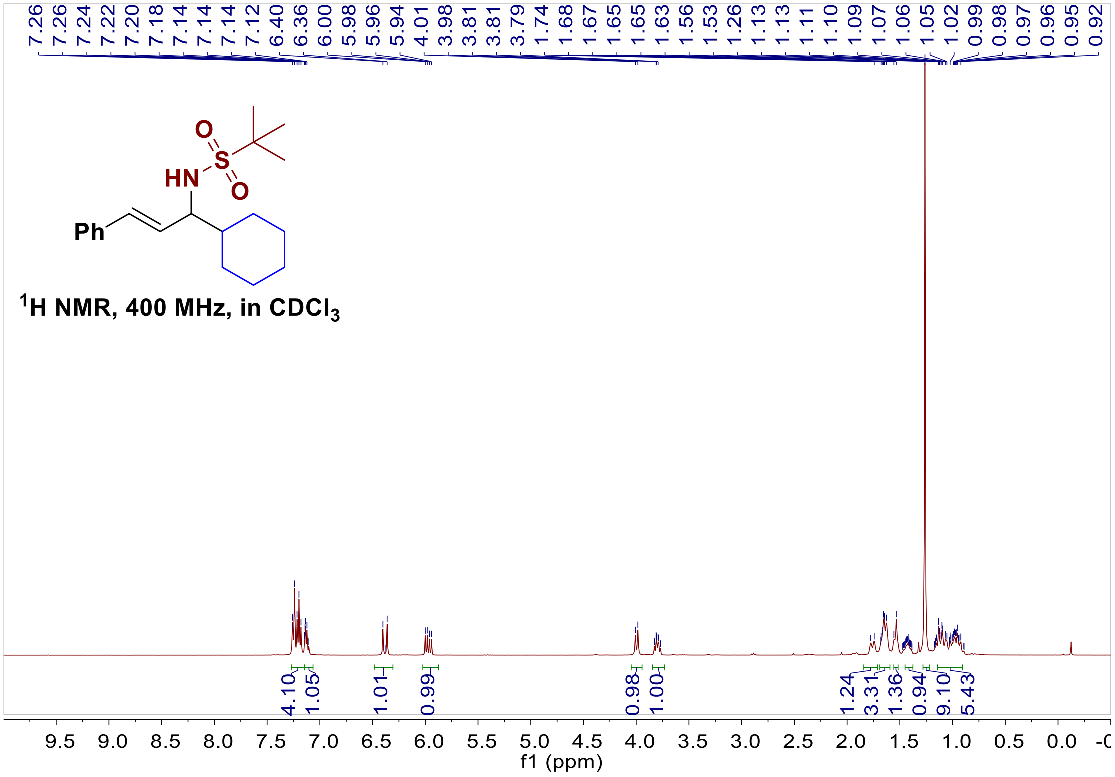

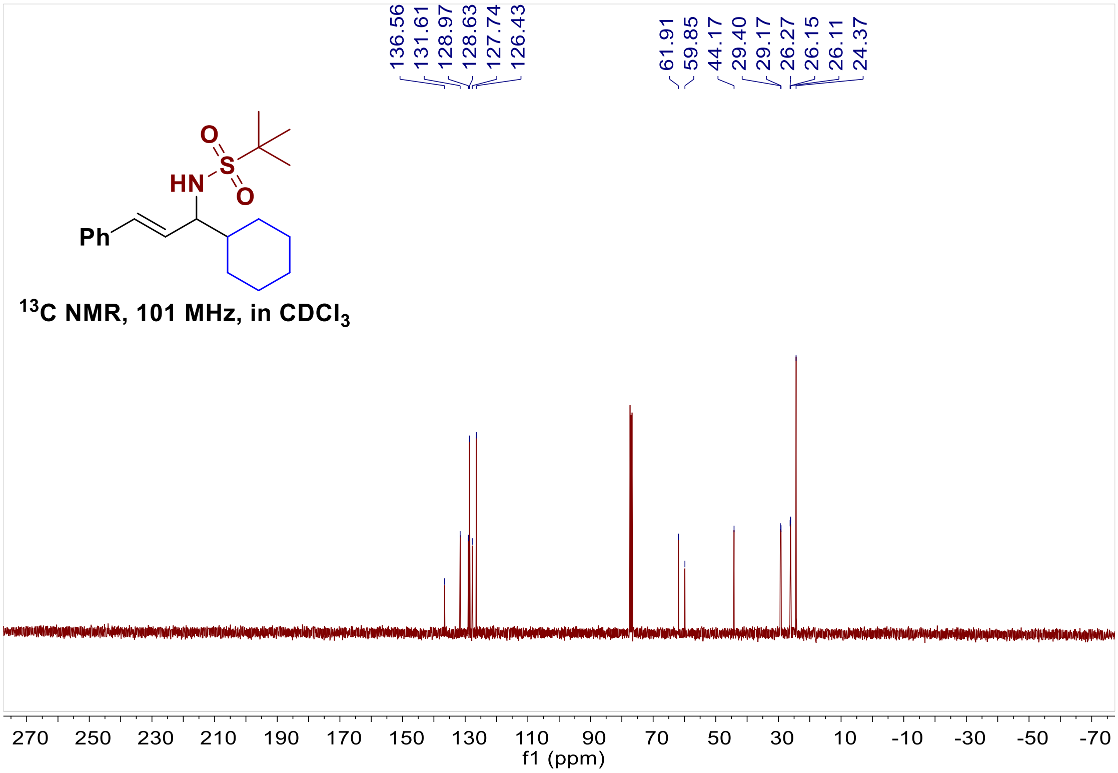

(*E*)-N-(1-cyclohexyl-3-phenylallyl)-P,P-diphenylphosphinicamide (10)

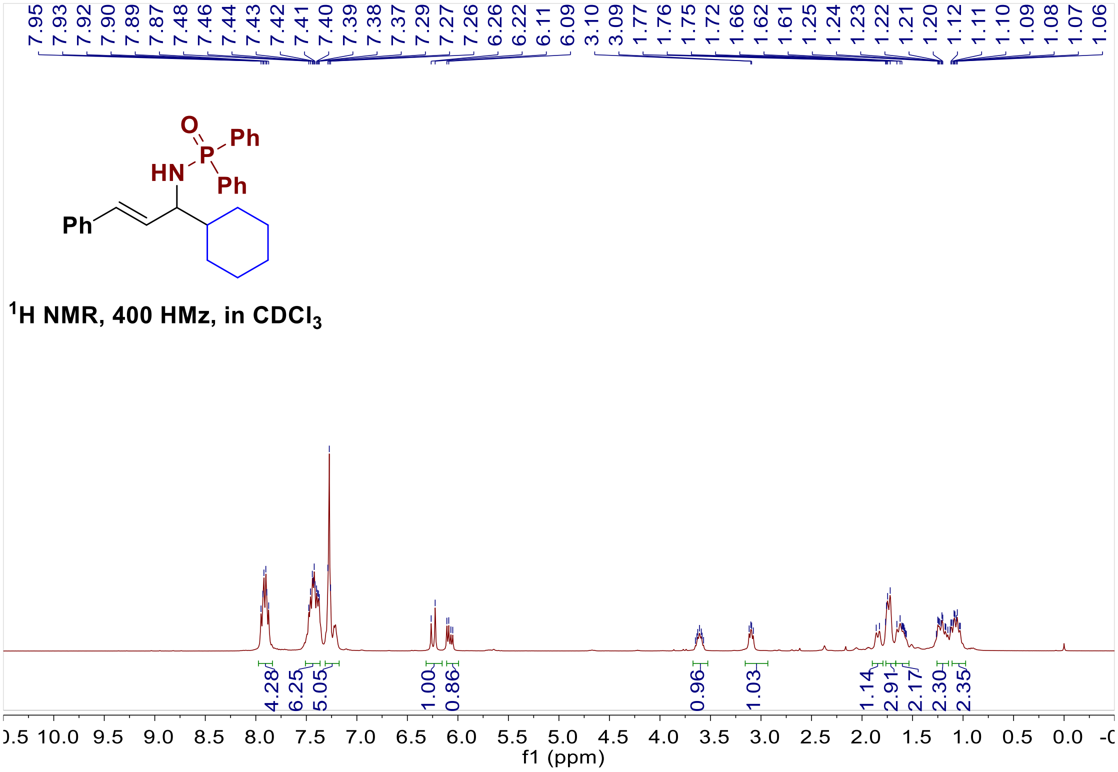

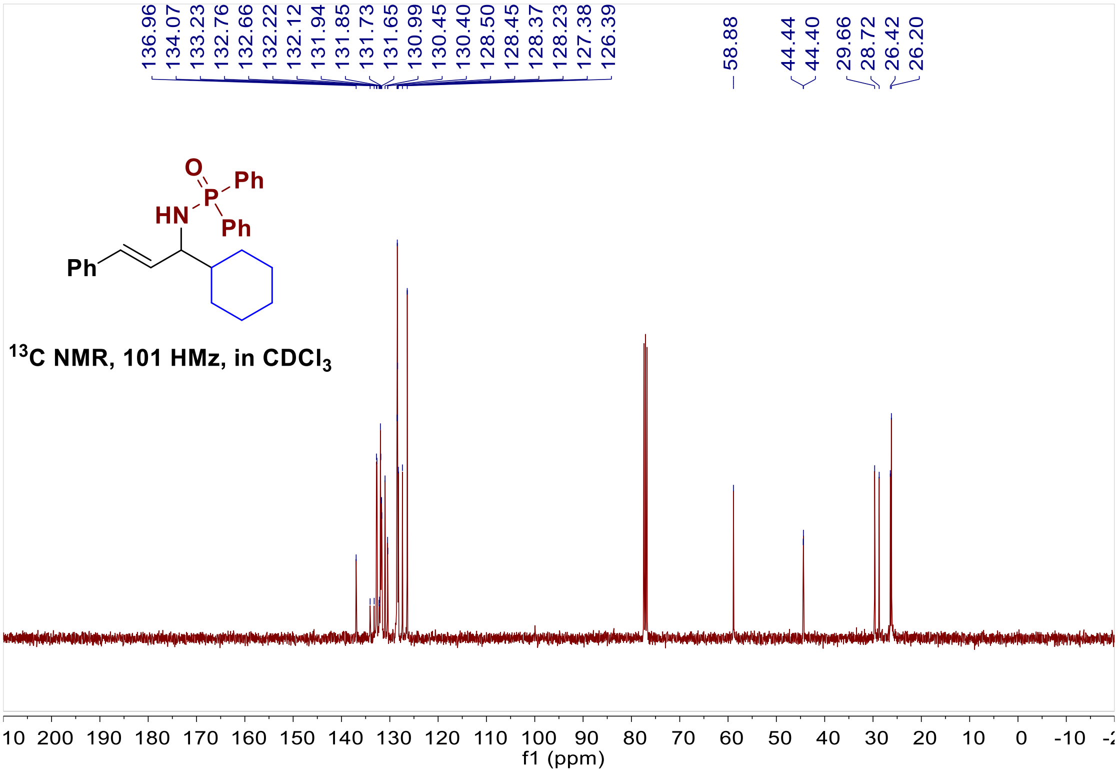

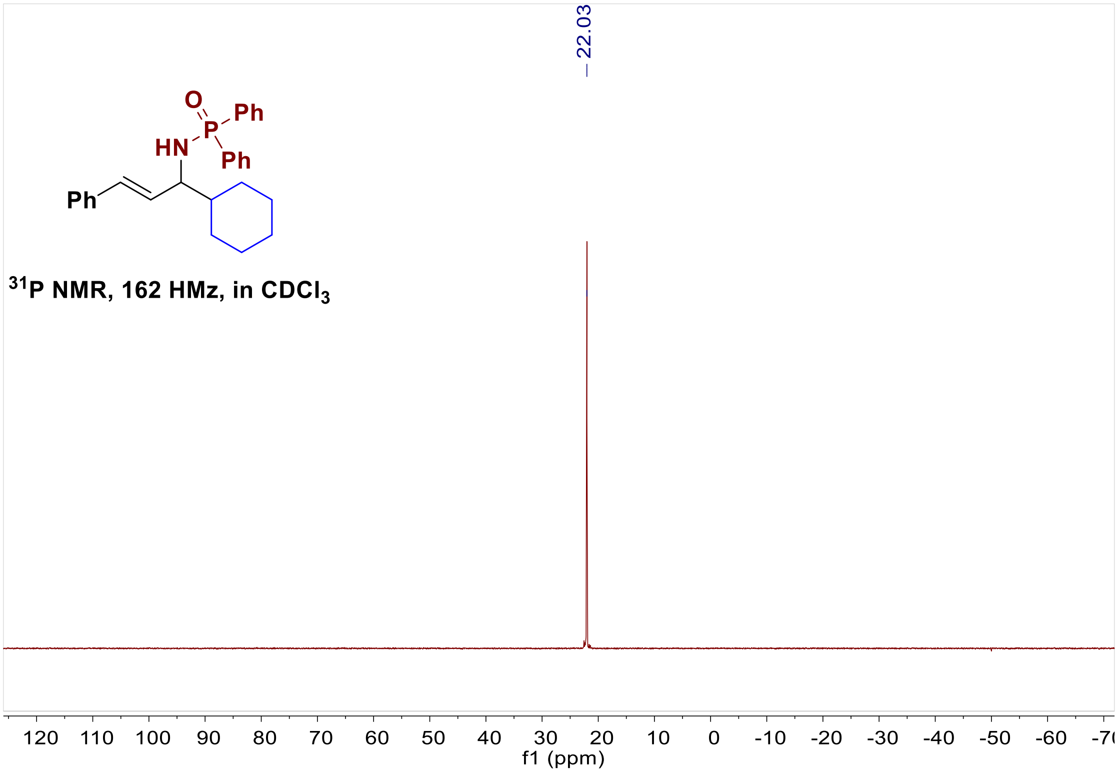
22.03
O
Ph
P
HN
Ph
Ph
31P NMR, 162 HMz, in CDCl3
120 110 100 90 80 70 60 50 40 30 20 10 0 -10 -20 -30 -40 -50 -60
f1 (ppm)

diethyl-(*E*)-(1-cyclohexyl-3-phenylallyl)phosphoramidate (11)

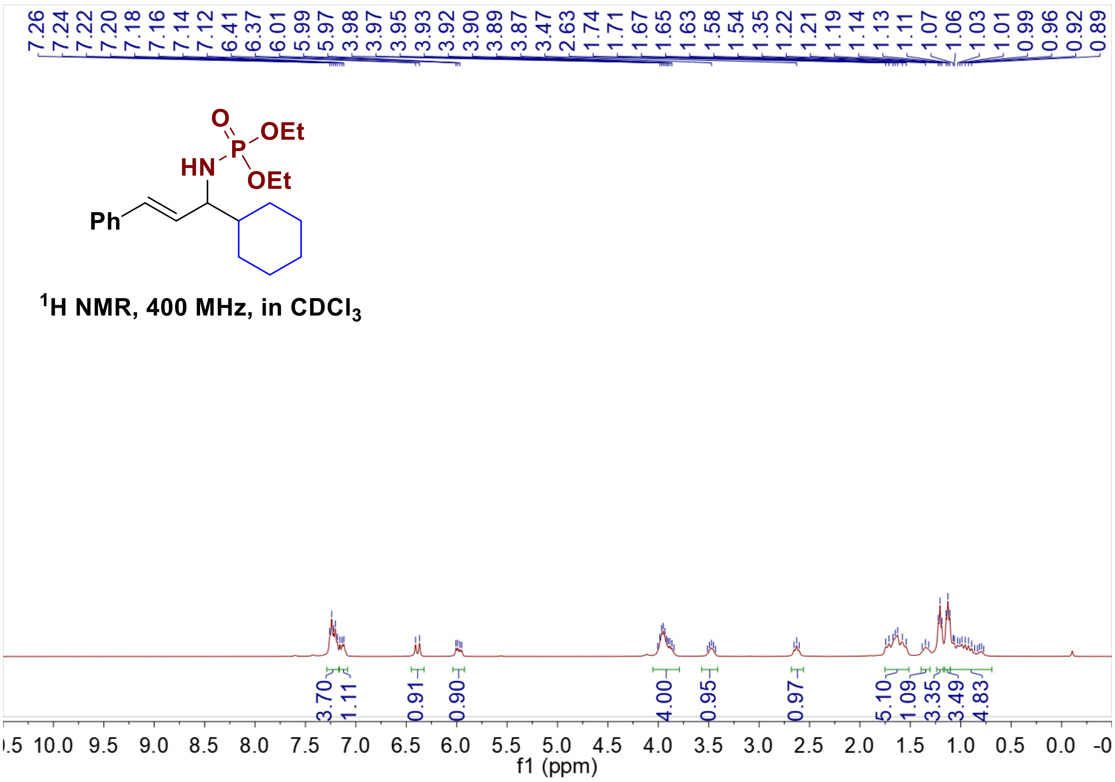

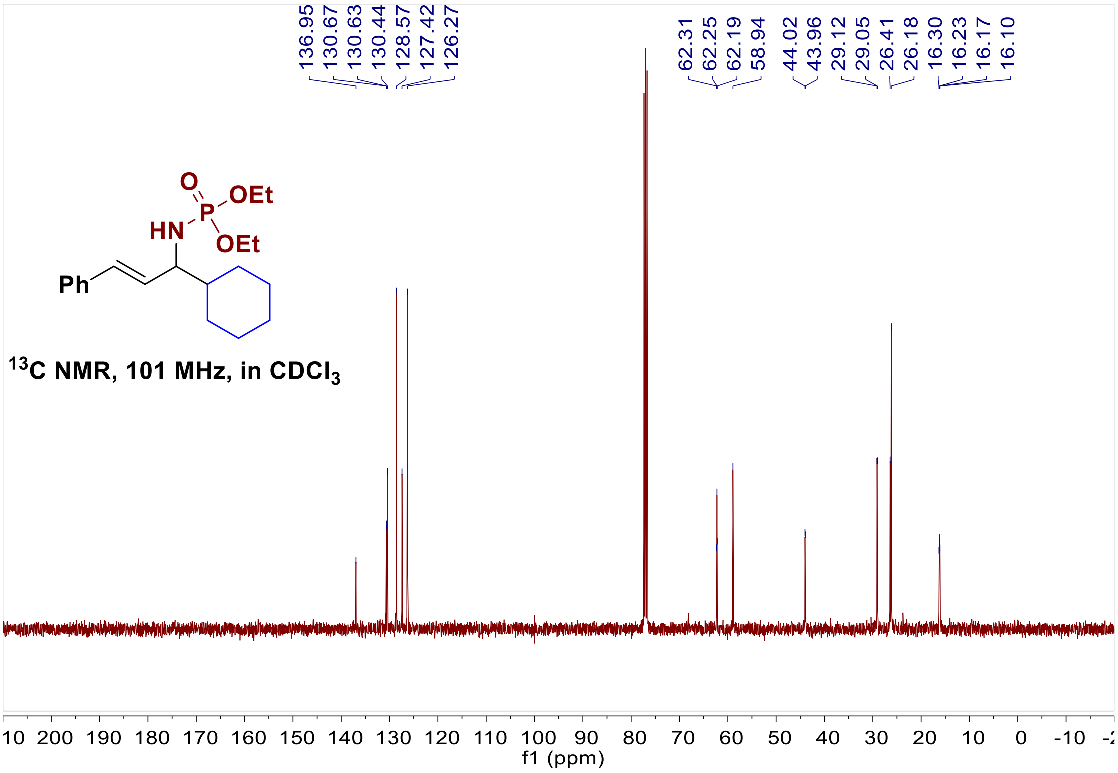

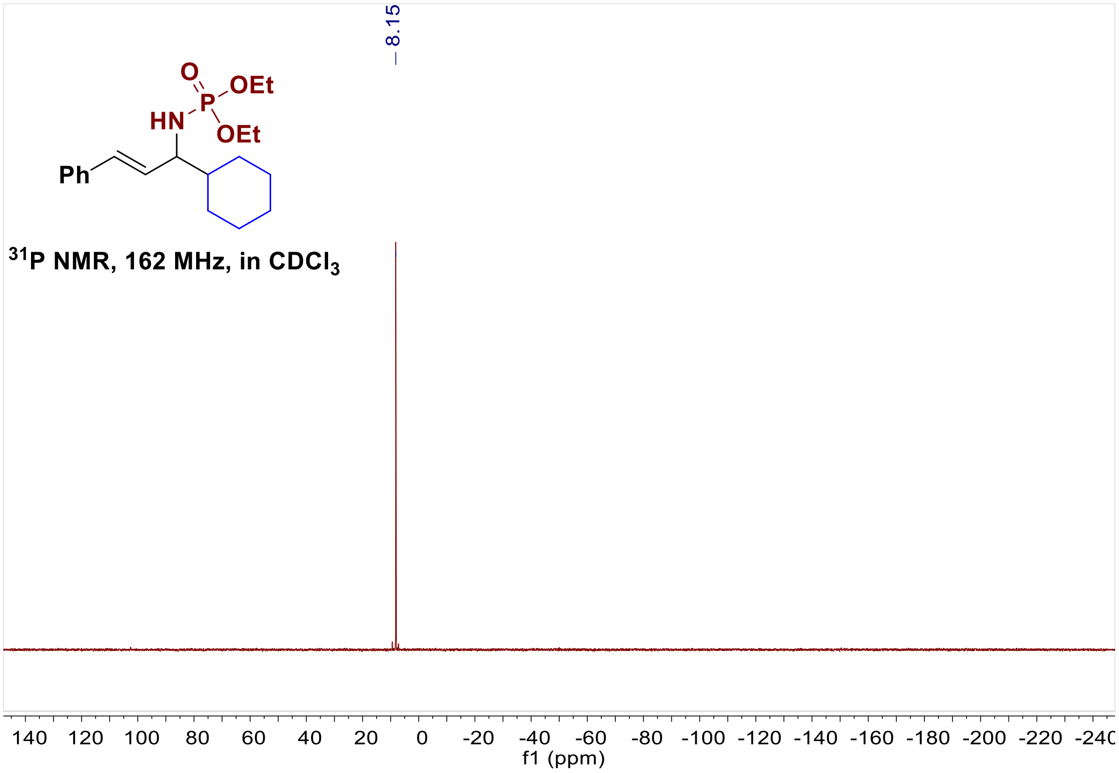

8.15
O
OEt
HN
P
OEt
Ph
31P NMR, 162 MHz, in CDCl3
140 120 100 80 60 40 20 0 -20 -40 -60 -80 -100 -120 -140 -160 -180 -200 -220 -240
f1 (ppm)

tert-butyl-(_E_)-(1-cyclohexyl-3-phenylallyl)carbamate (12)

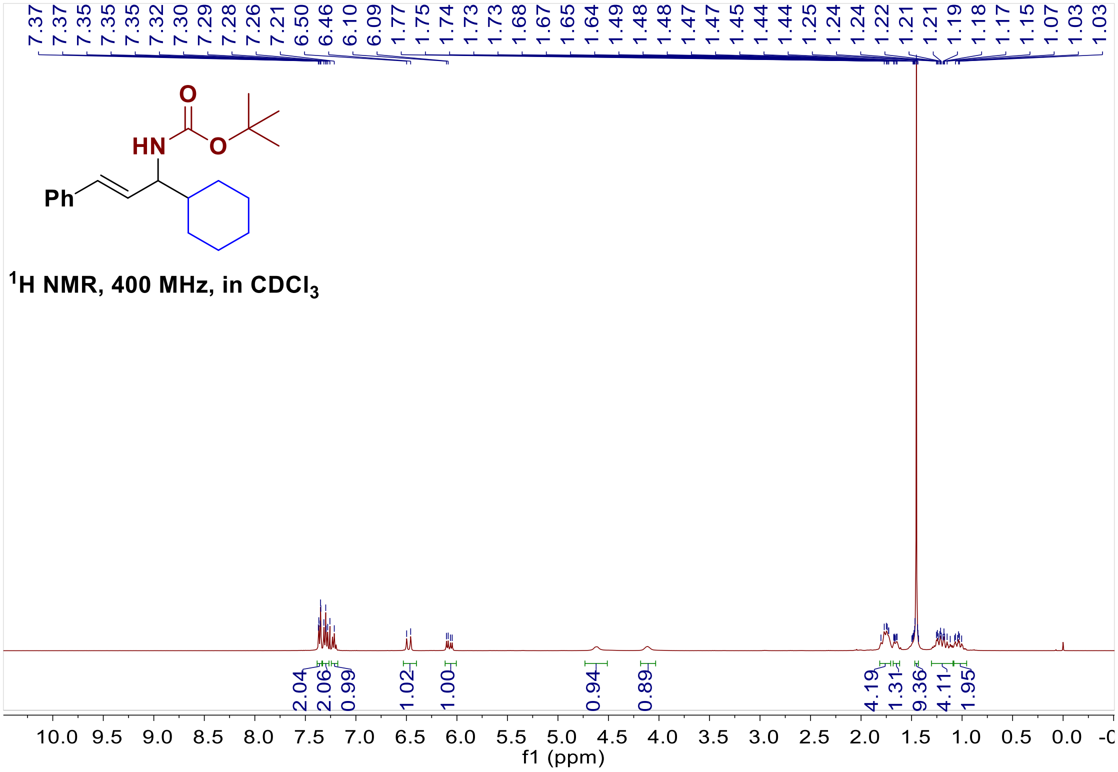

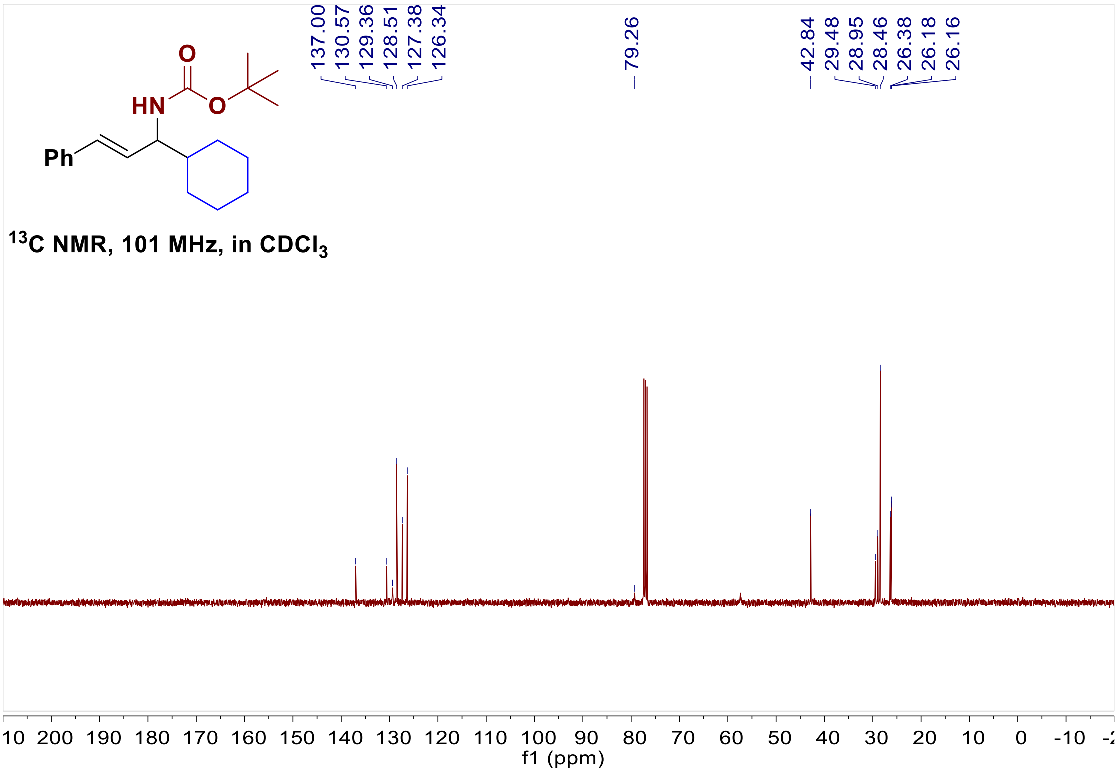

(*E*)-N-(1-cyclohexyl-3-phenylallyl)benzamide (13)

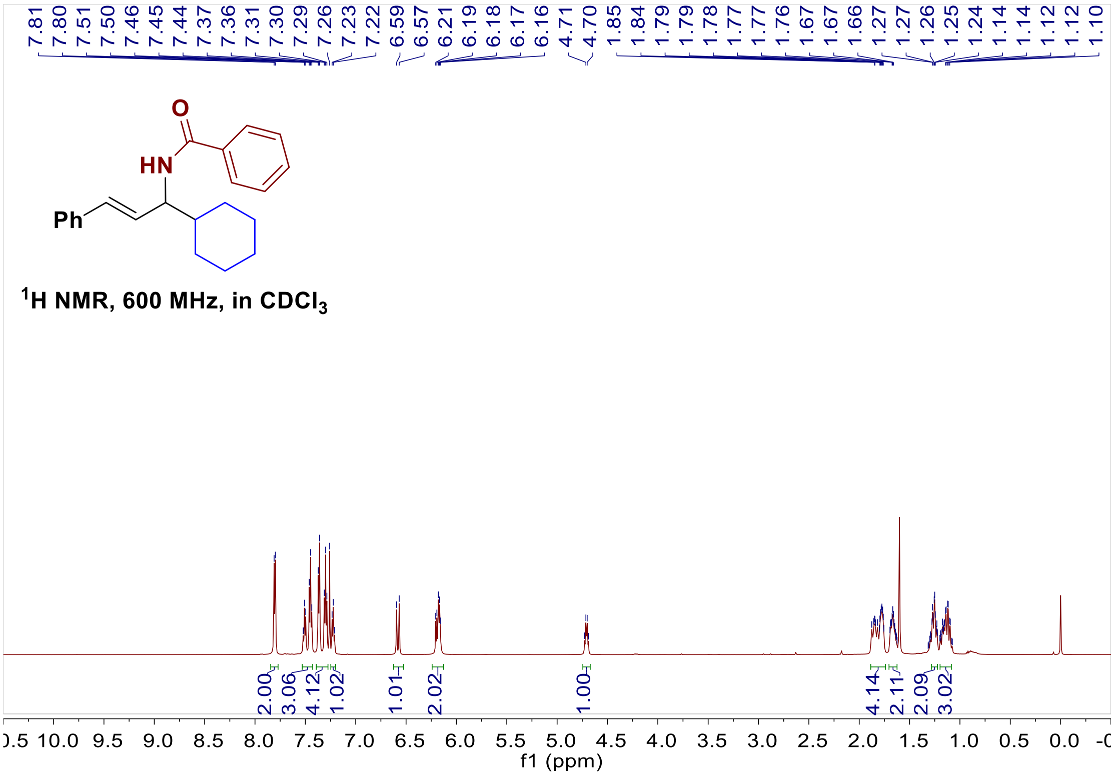

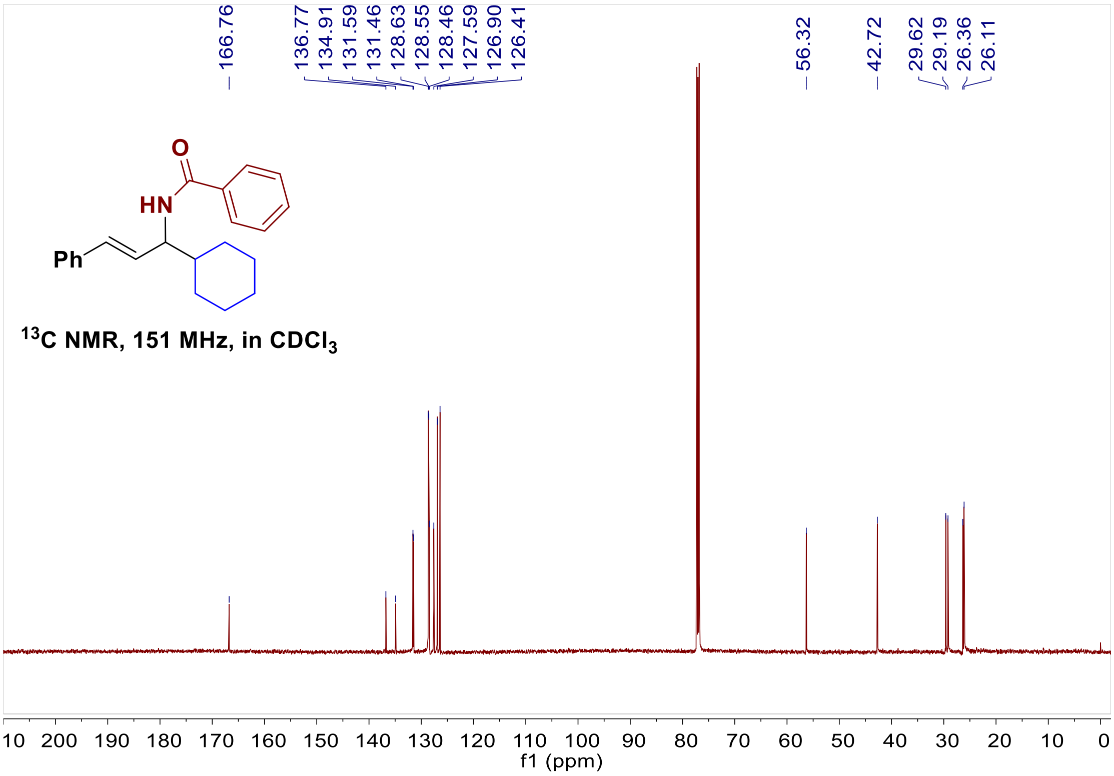

(*E*)-N-(1-cyclohexyl-3-phenylallyl)-4-(trifluoromethyl)-benzamide (14)

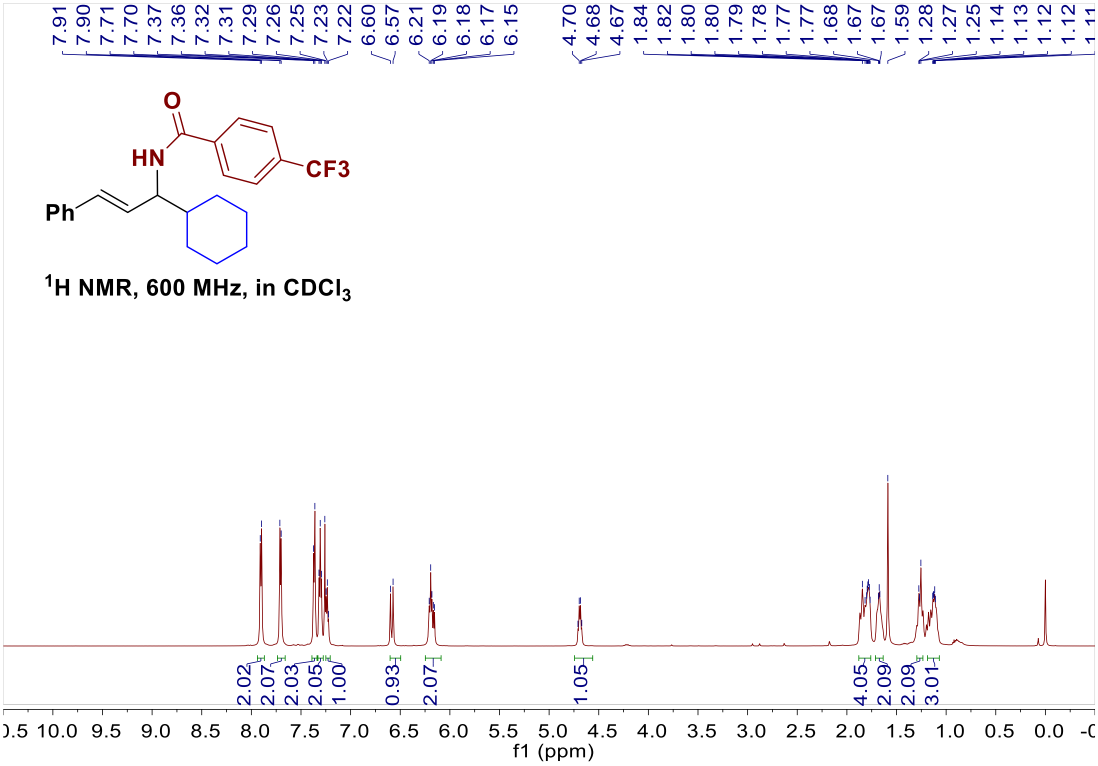

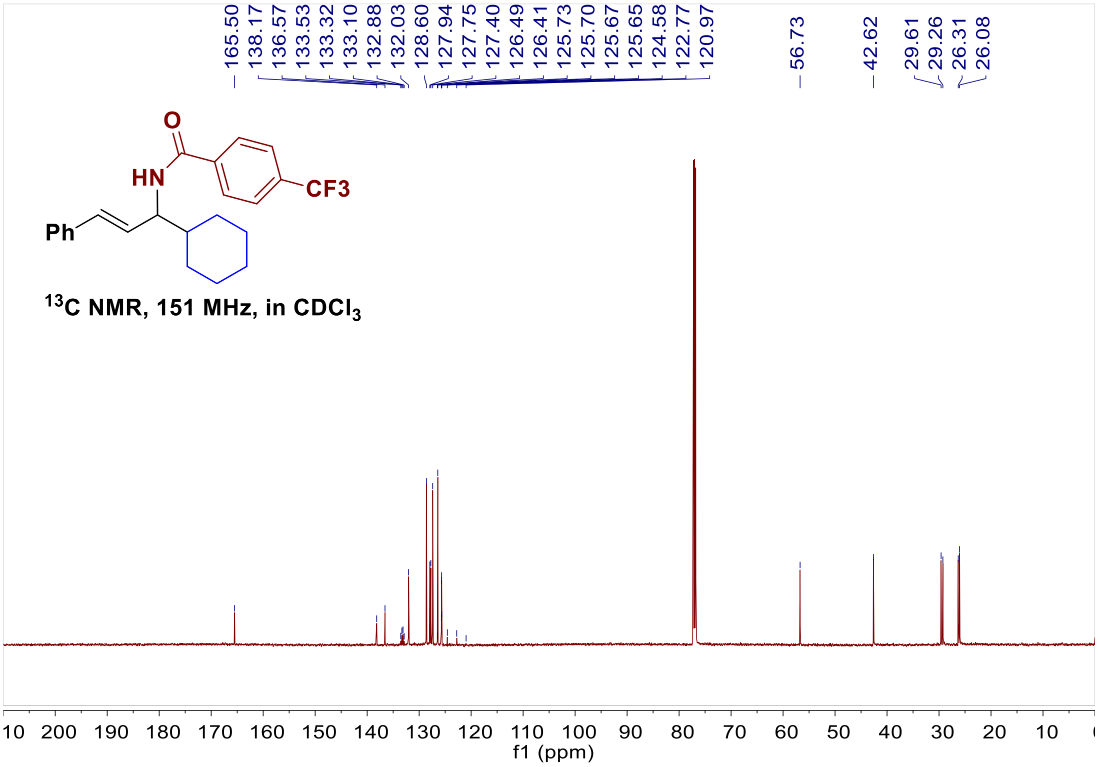

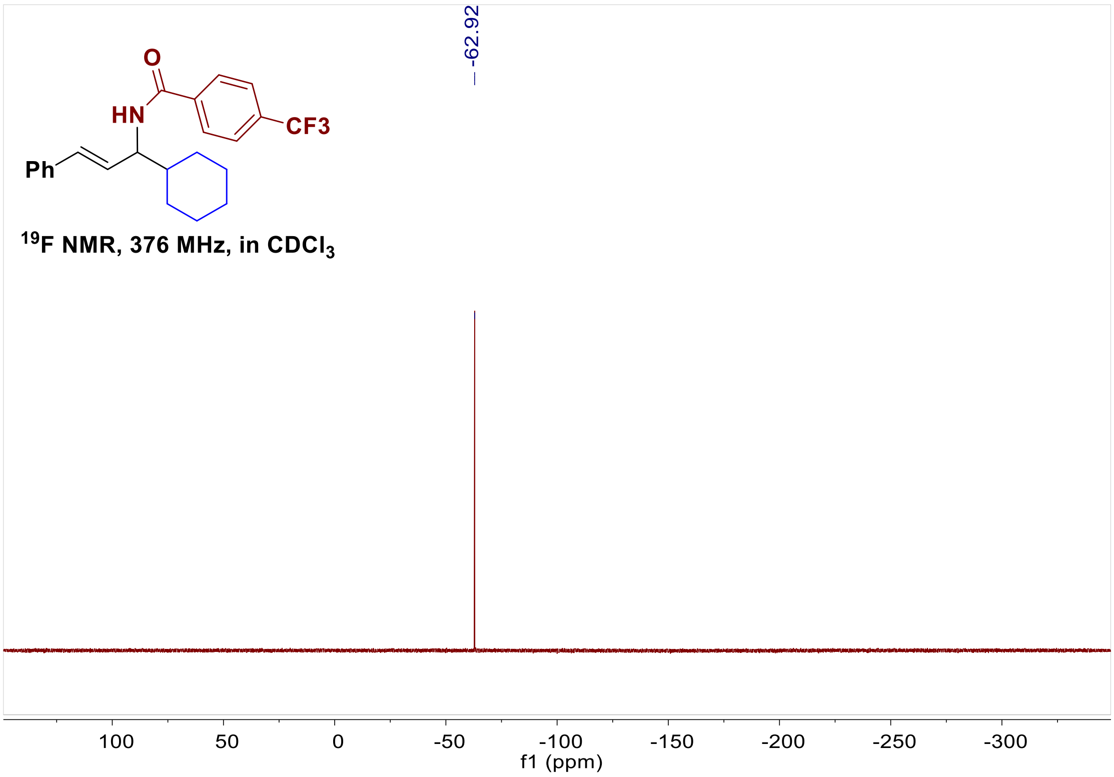
-62.92
O
HN
CF3
Ph
19F NMR, 376 MHz, in CDCl3
100
50
0
-50
-100
-150
-200
-250
-300
f1 (ppm)

(*E*)-N-(1-cyclohexyl-3-phenylallyl)cyclohexanecarboxamide (15)

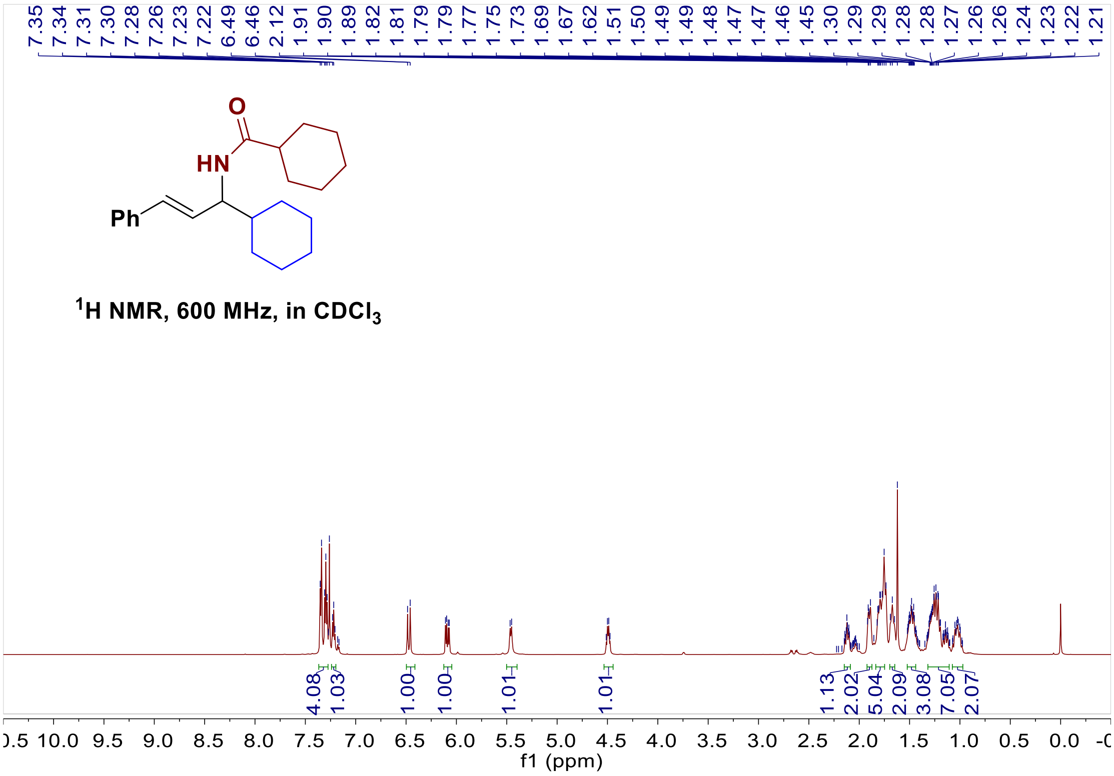

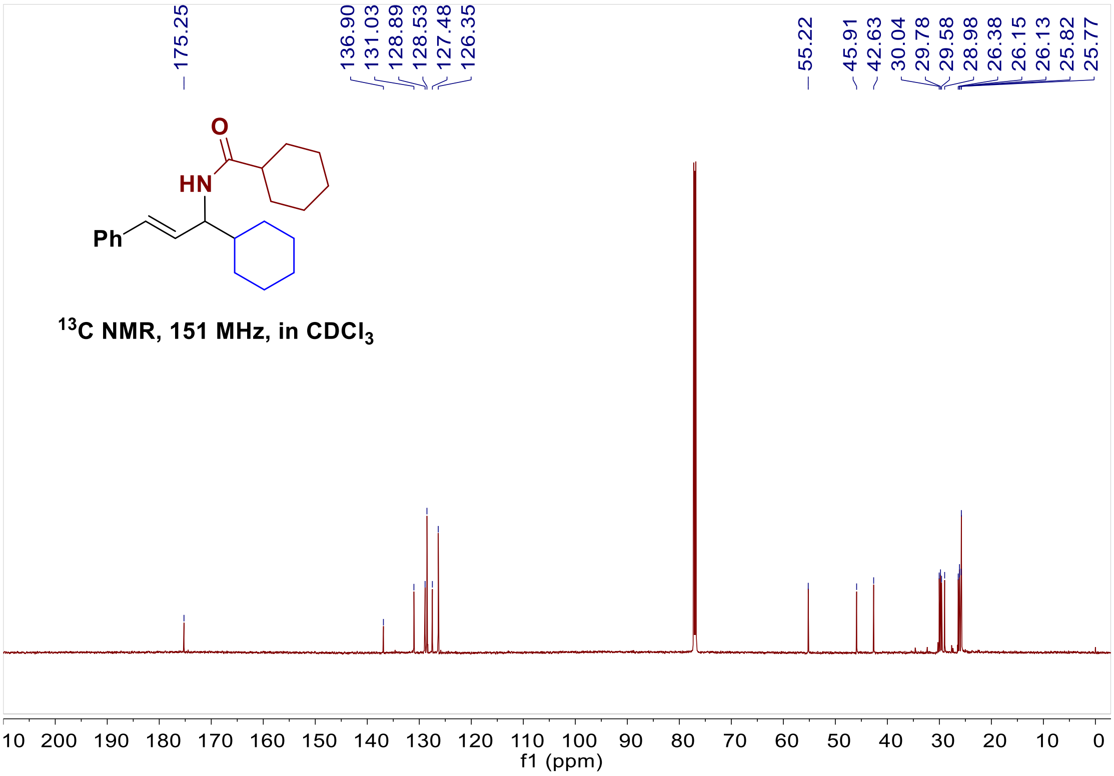

(*E*)-N-(1-cyclohexyl-3-phenylallyl)-2-phenylacetamide (16)

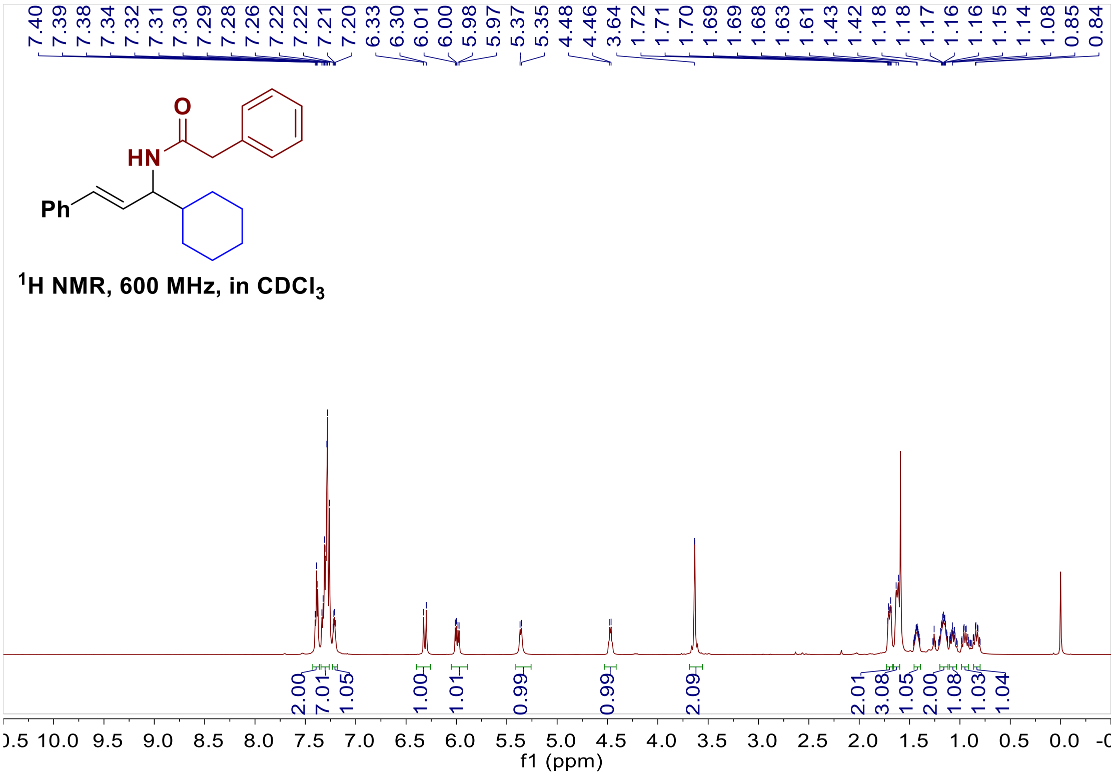

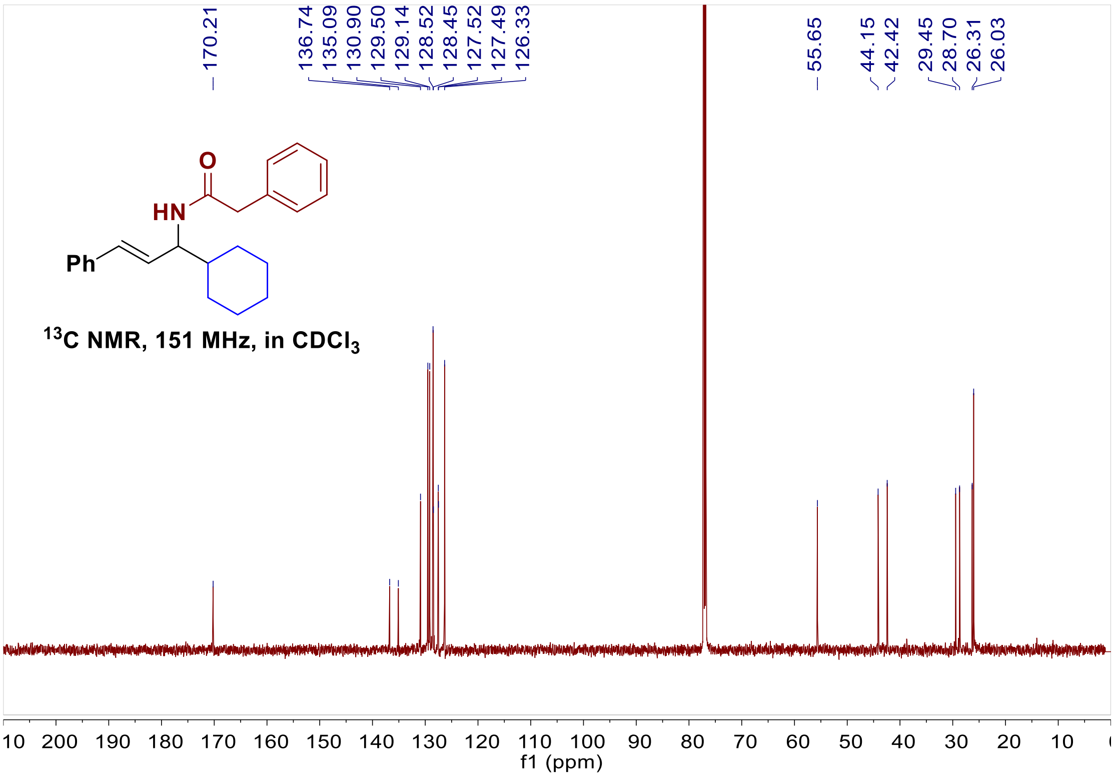

(*E*)-N-(1-(2,4-dimethoxyphenyl)-3-phenylallyl)-4-methylbenzenesulfonamide (18)

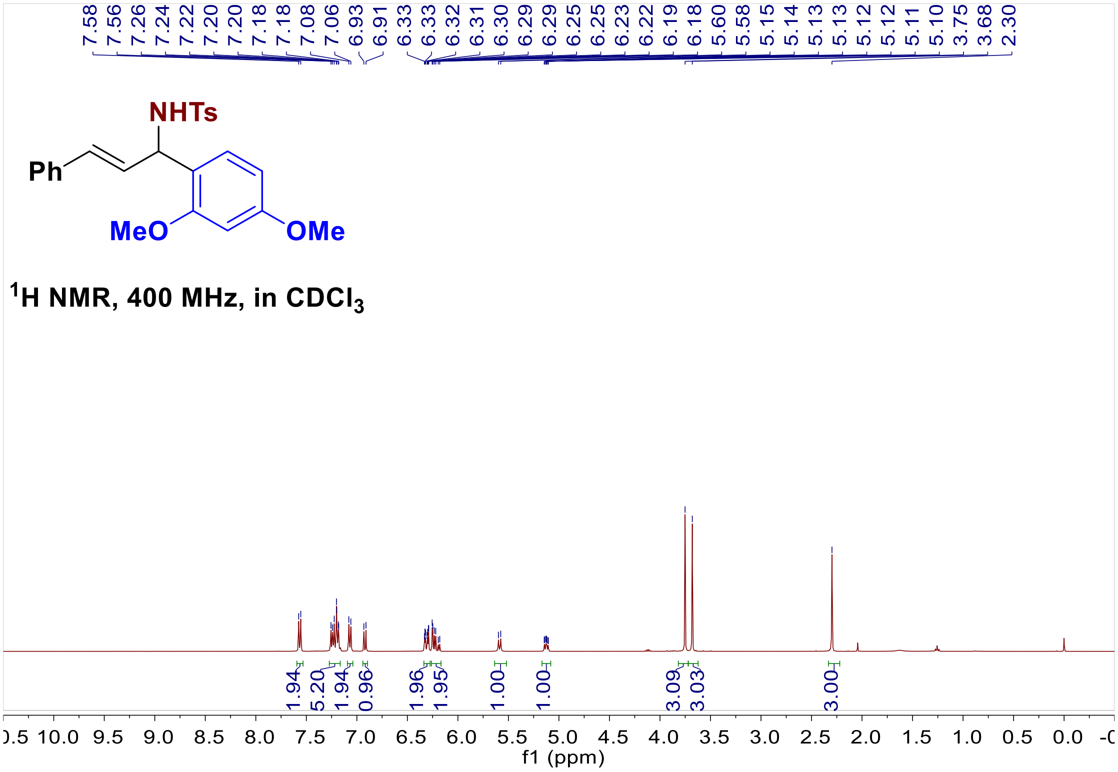

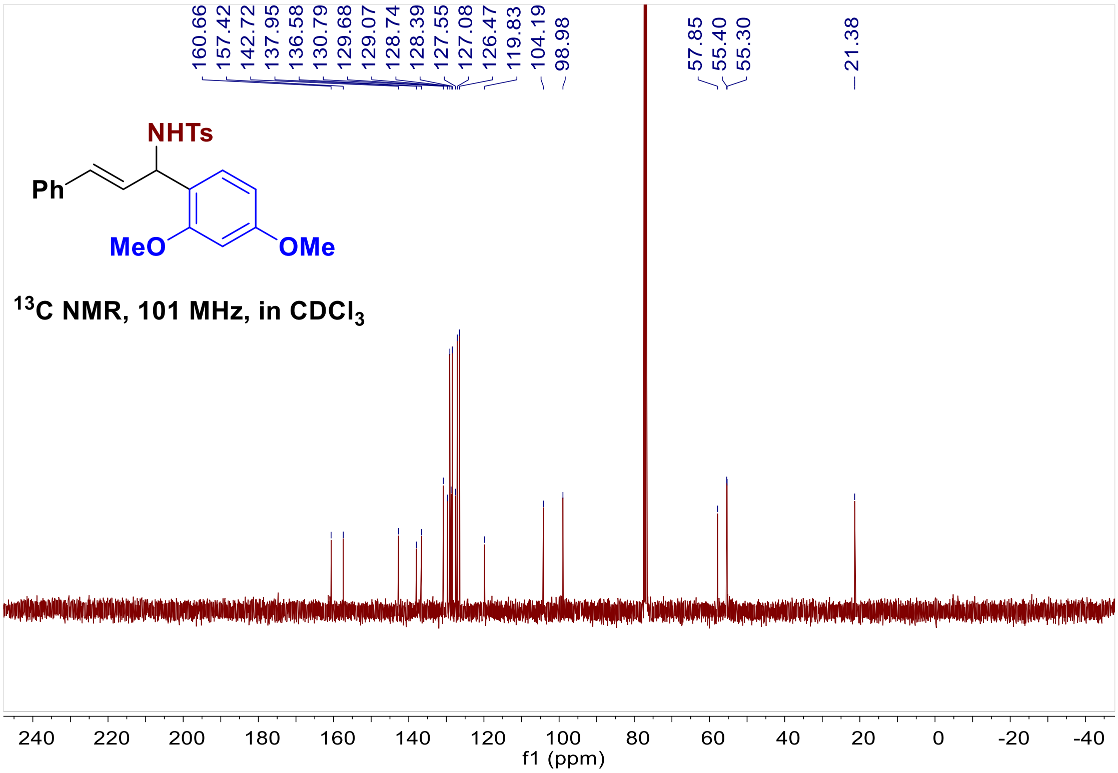

(*E*)-N-(1-(3-chloro-4-methylphenyl)-3-phenylallyl)-4-methylbenzenesulfonamide (19)

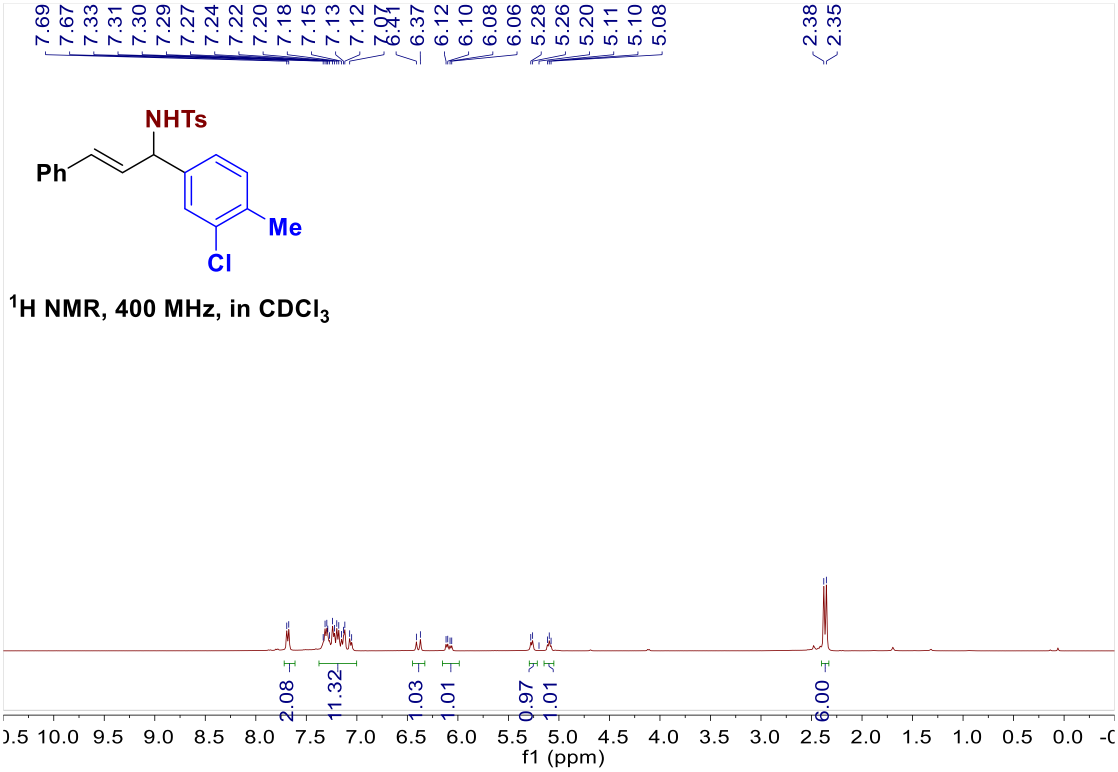

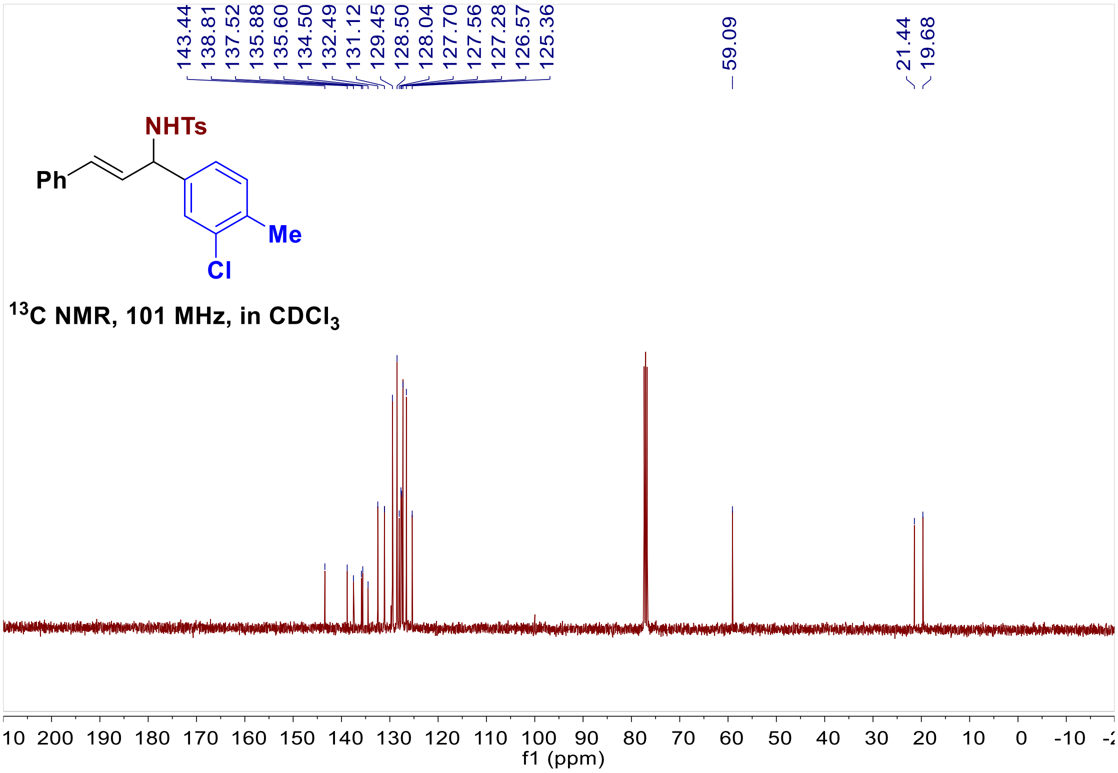

(*E*)-N-(1-(6-methoxypyridin-3-yl)-3-phenylallyl)-4-methylbenzenesulfonamide (21)

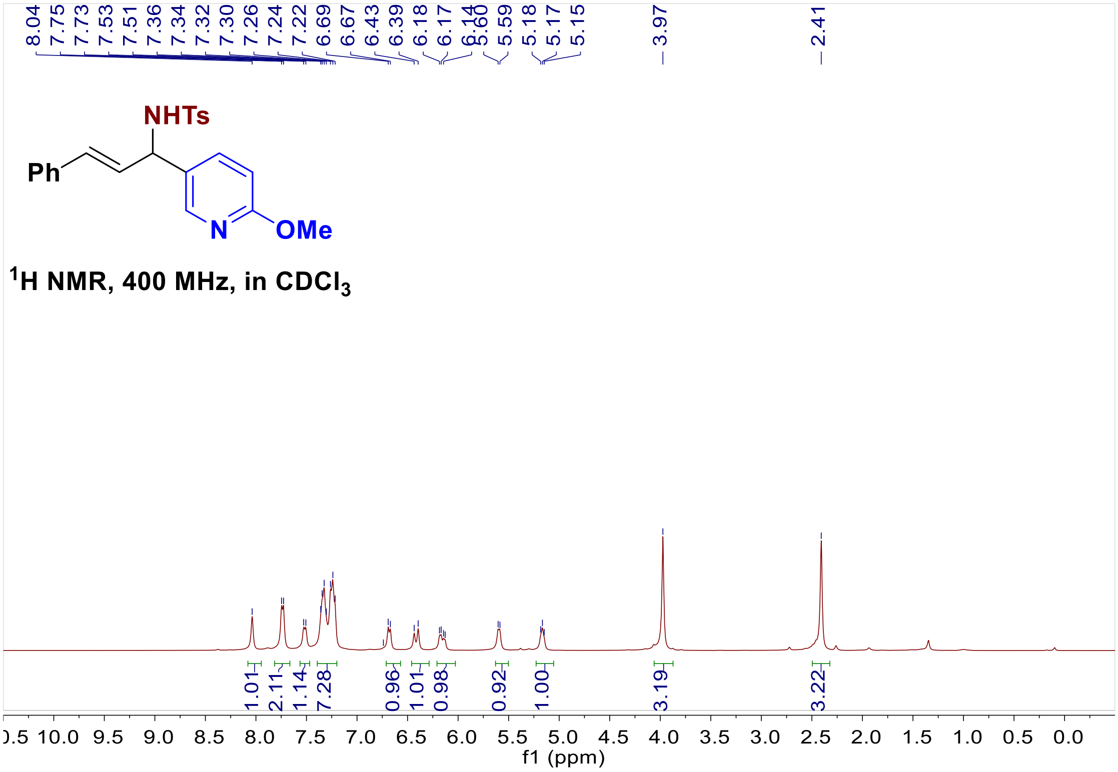

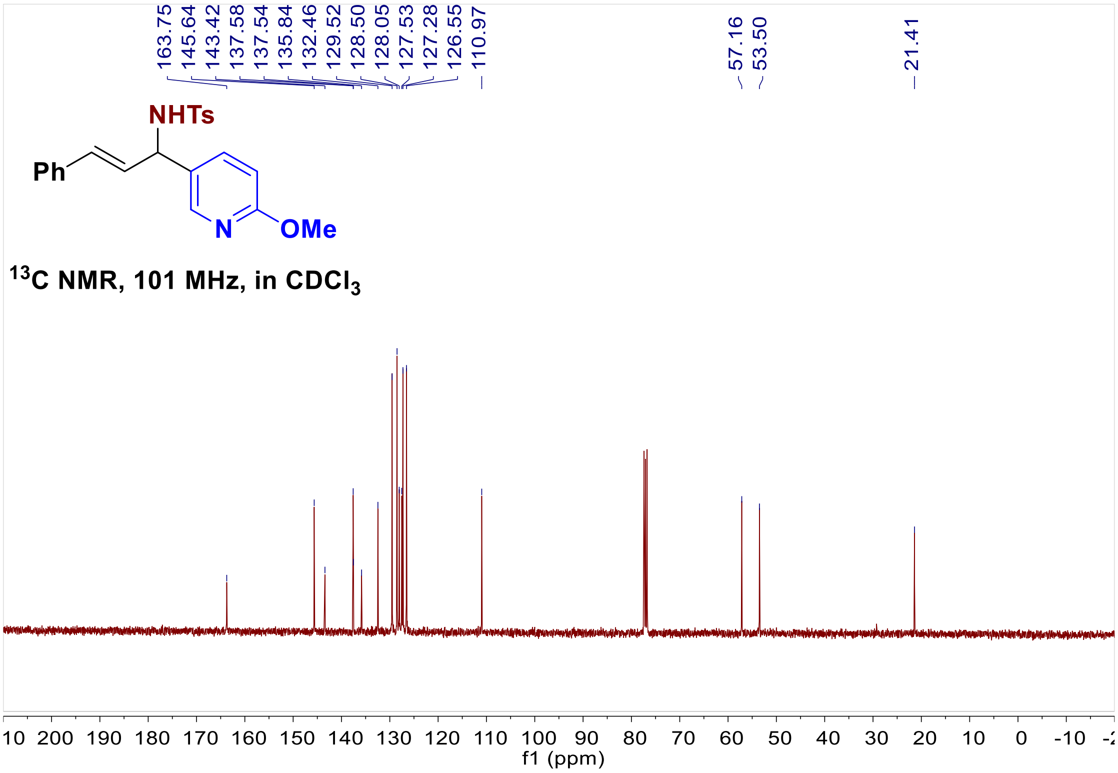

(*E*)-4-methyl-N-(3-phenyl-1-(1-tosyl-1H-indol-3-yl)allyl)benzenesulfonamide (22)

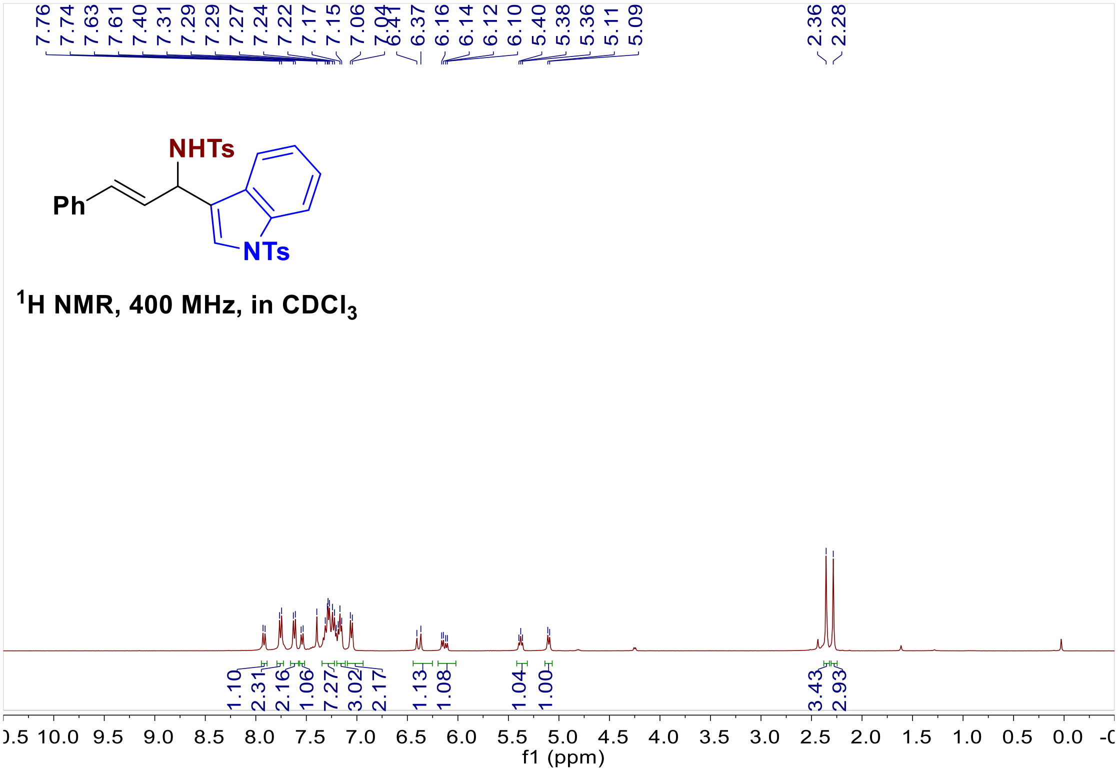

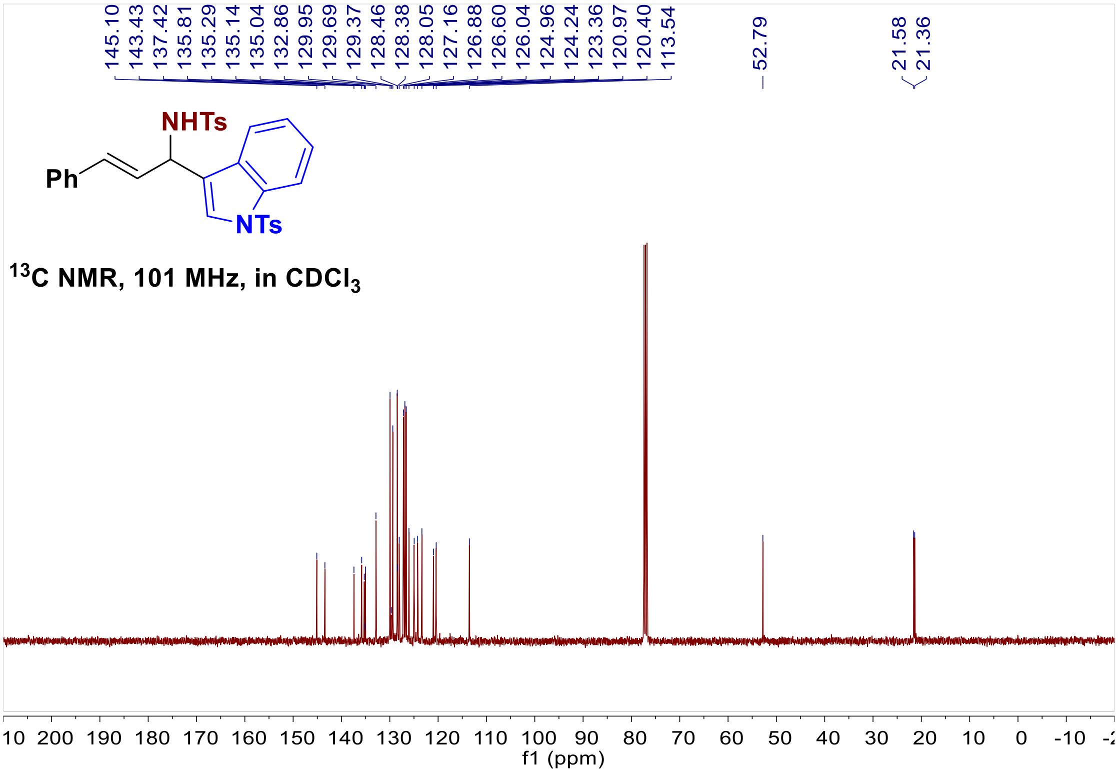

(*E*)-4-methyl-N-(1-(5-methylfuran-2-yl)-3-phenylallyl)benzenesulfonamide (23)

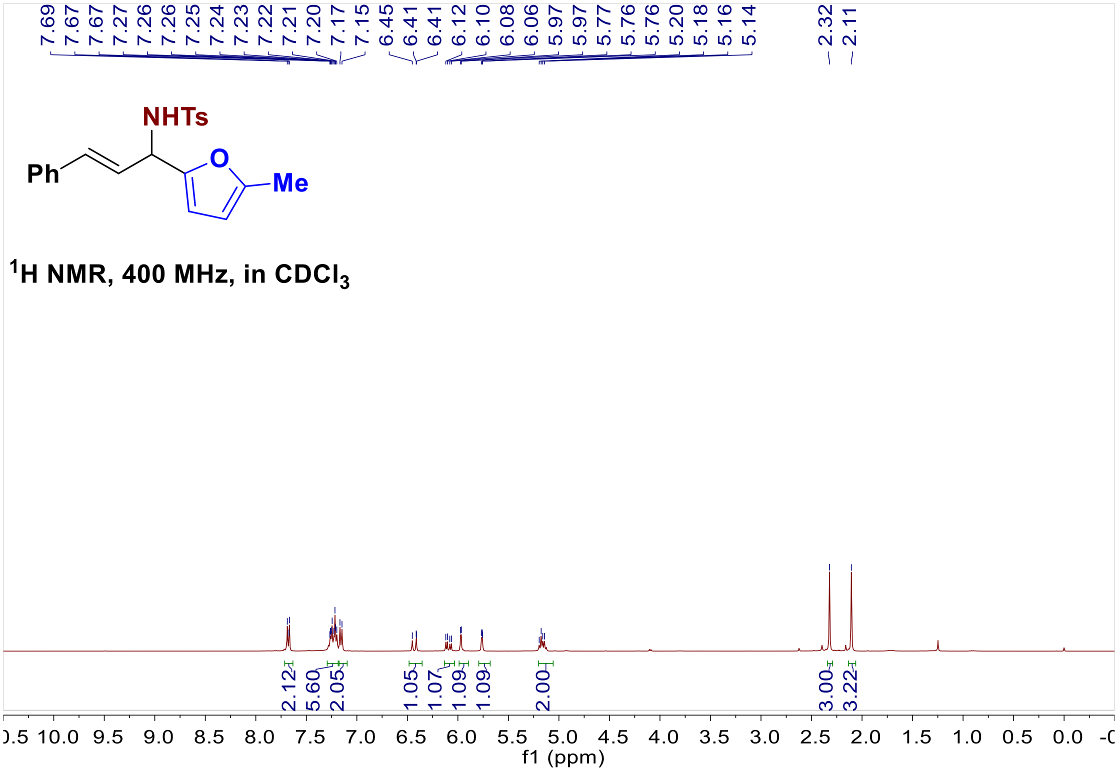

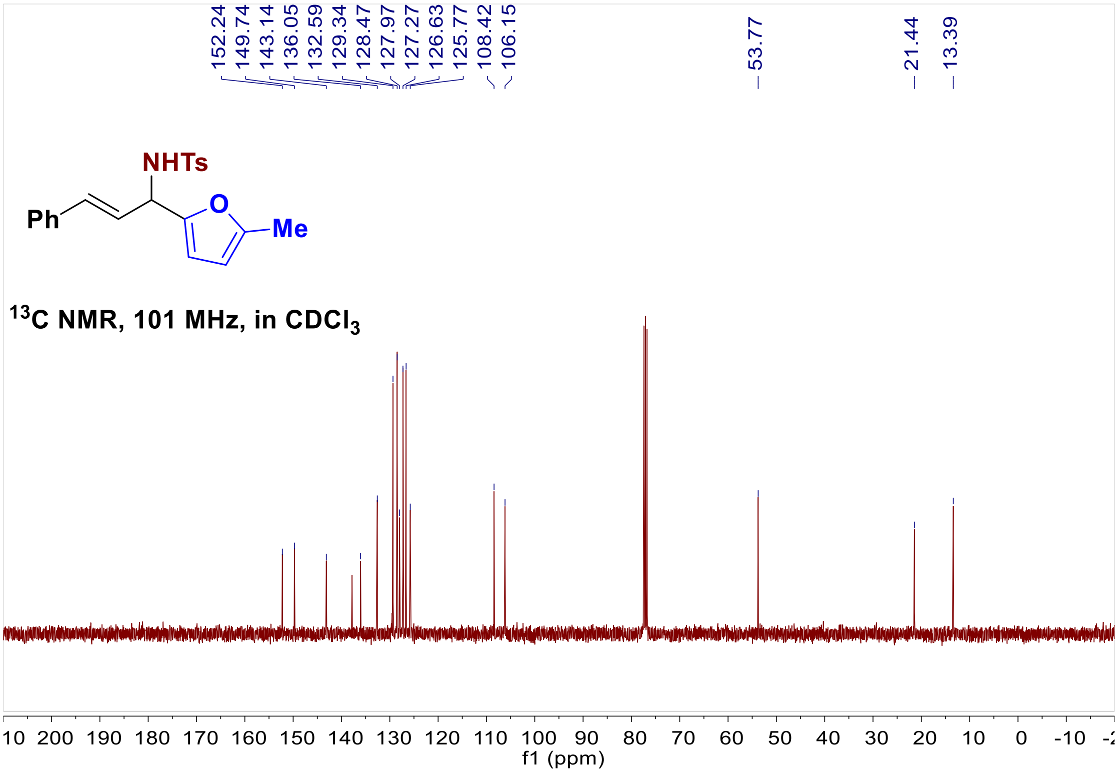

(*E*)-4-methyl-N-(1-(5-methylthiophen-2-yl)-3-phenylallyl)benzenesulfonamide (24)

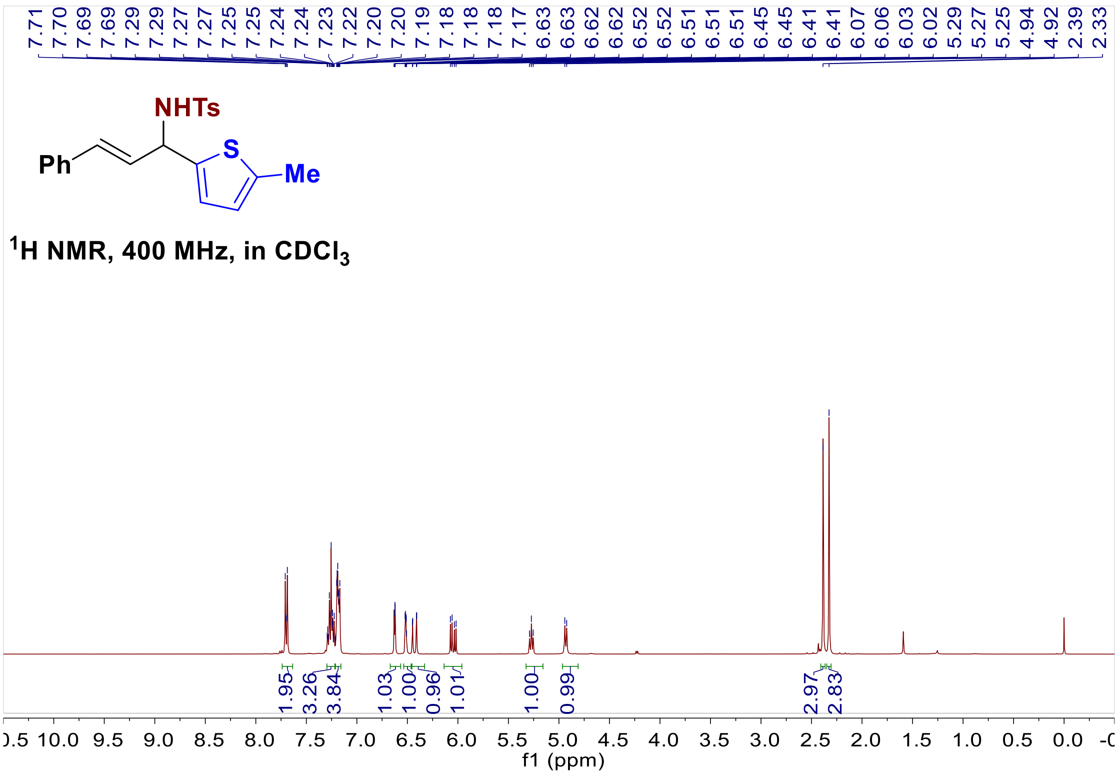

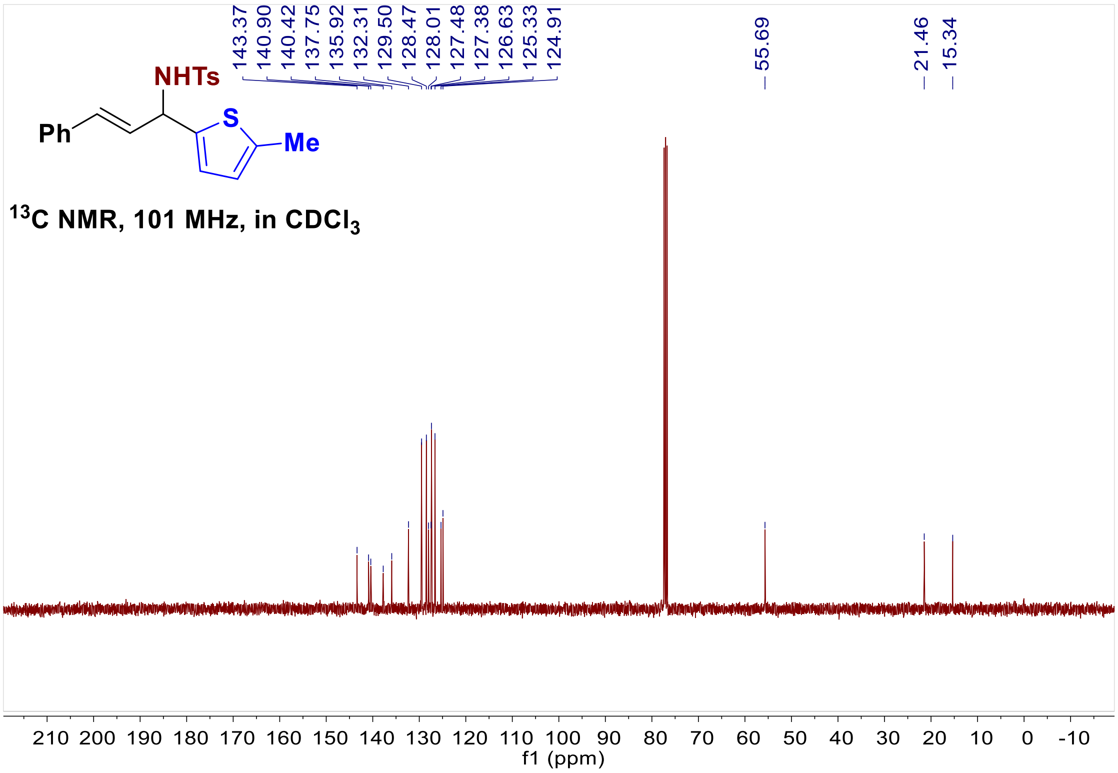

(*E*)-N-(1-(4,4-difluorocyclohexyl)-3-phenylallyl)-4-methylbenzenesulfonamide (25)

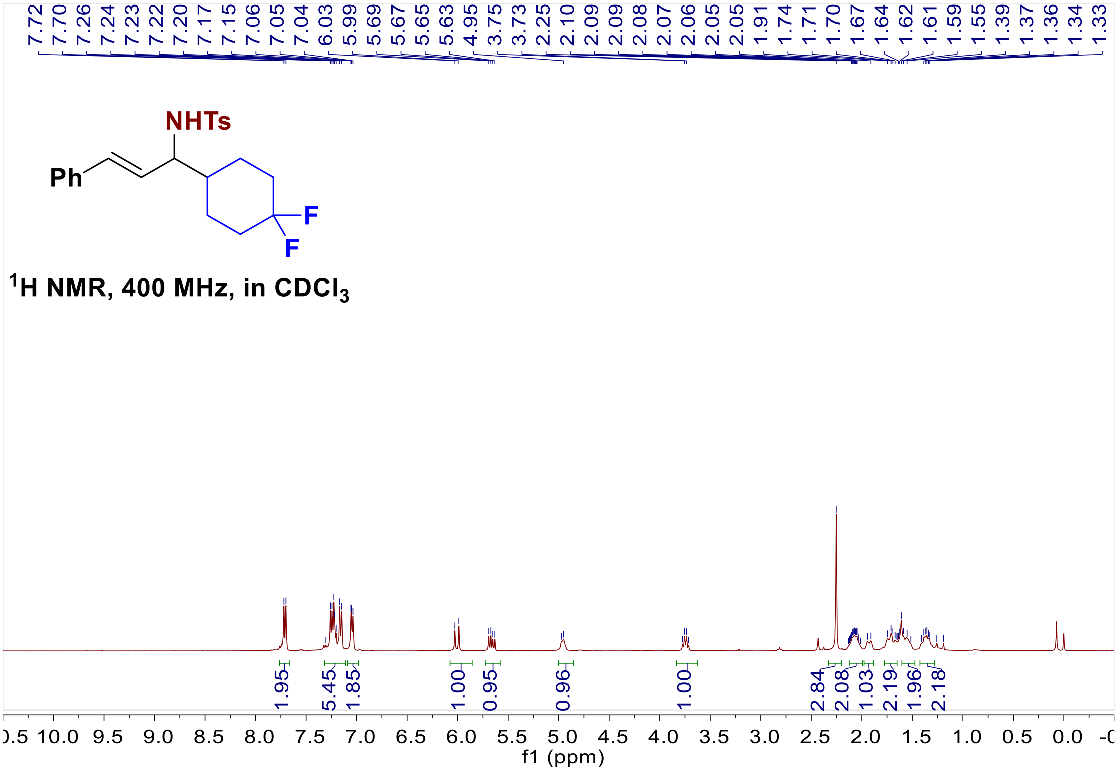

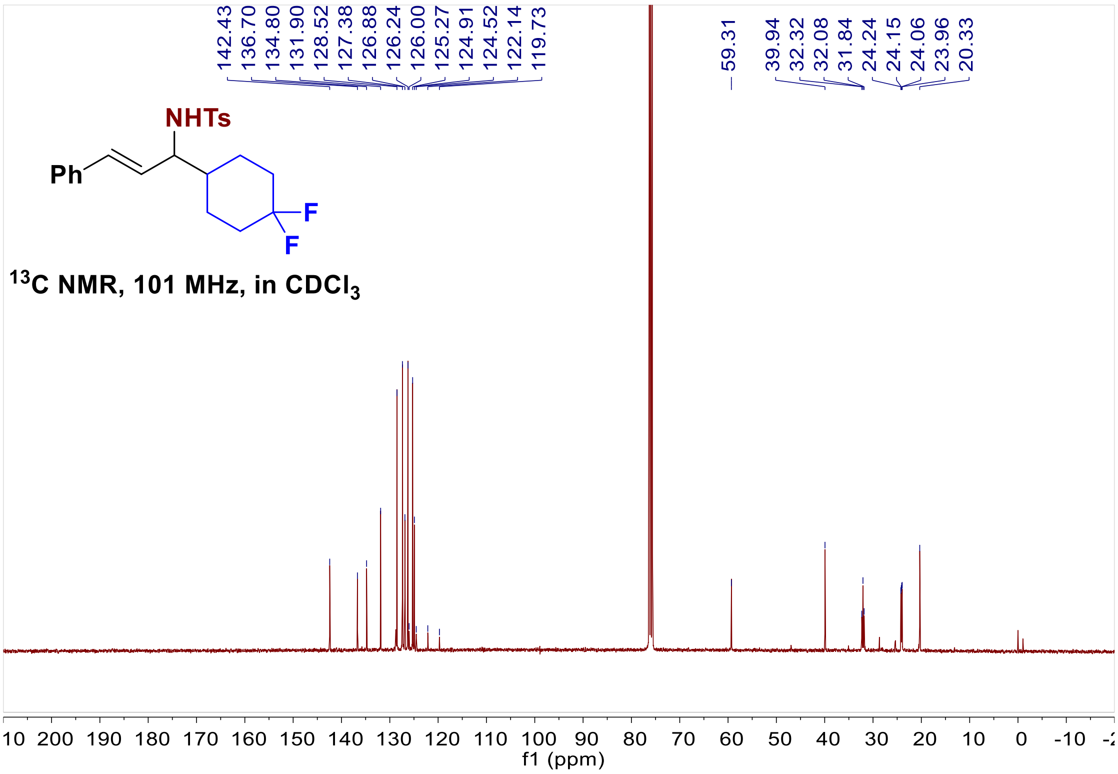

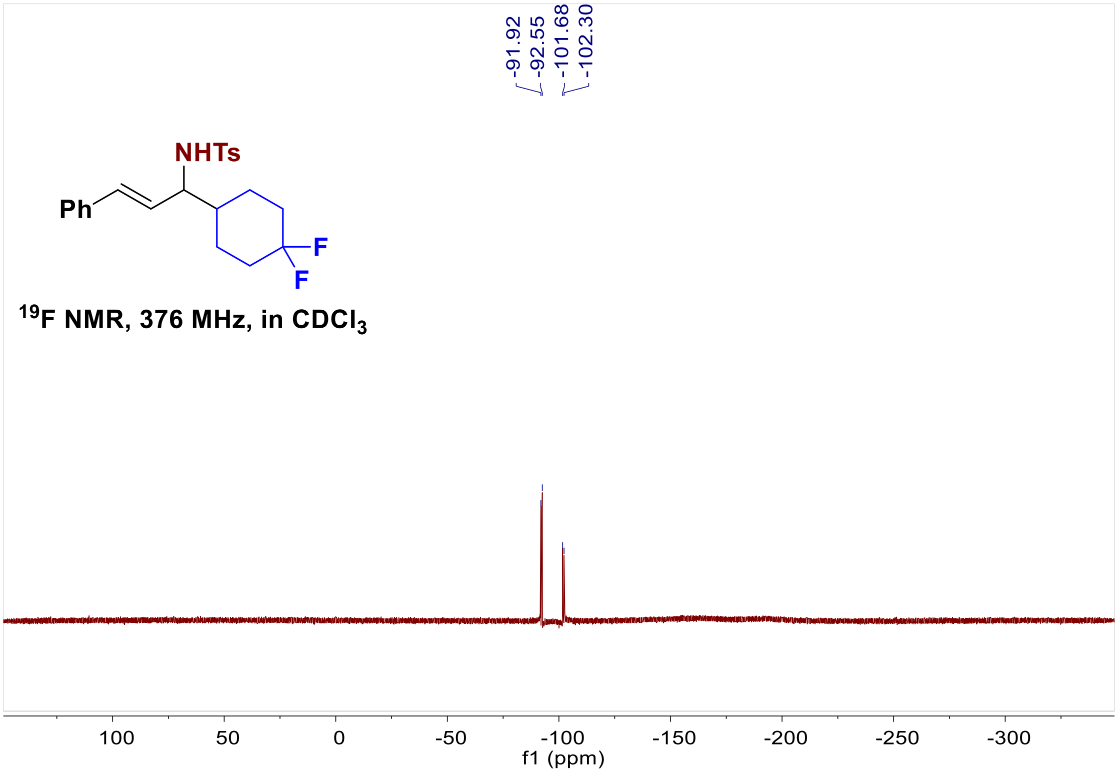

-91.92
-92.55
-101.68
-102.30
NHTs
Ph
F
F
19F NMR, 376 MHz, in CDCl3
100
50
0
-50
-100
-150
-200
-250
-300
f1 (ppm)

tert-butyl-(_E_)-4-(1-((4-methylphenyl)sulfonamido)-3-phenylallyl)piperidine-1-carboxylate (27)

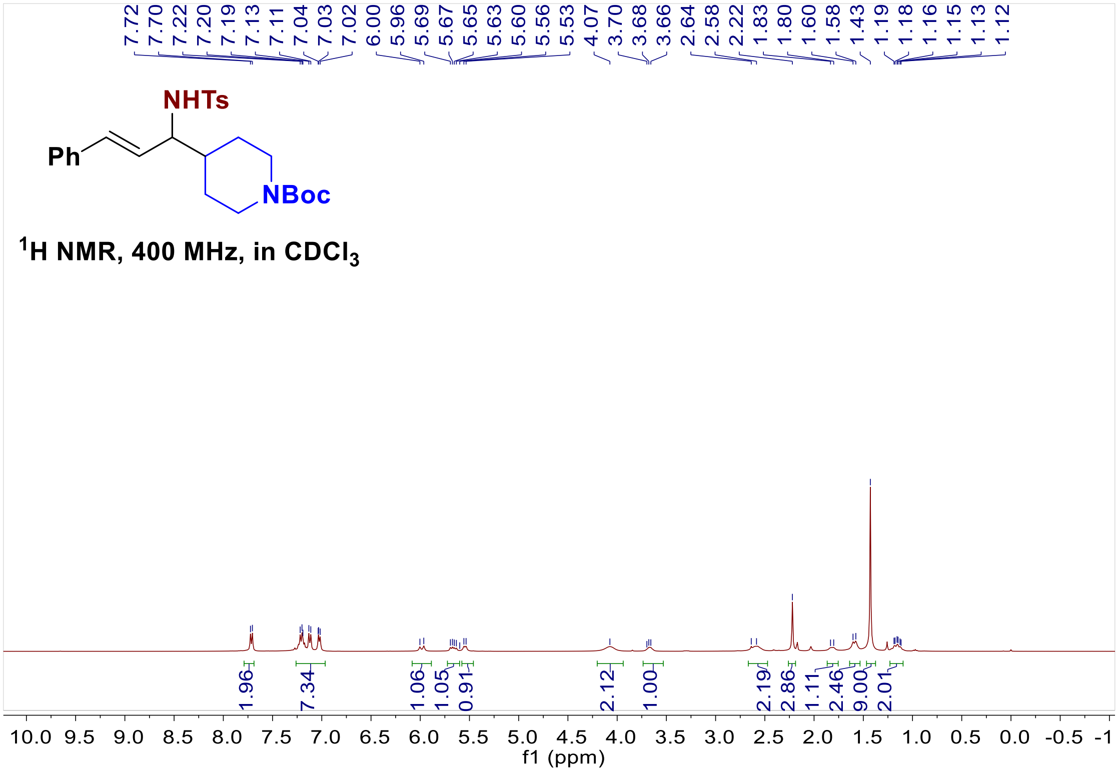

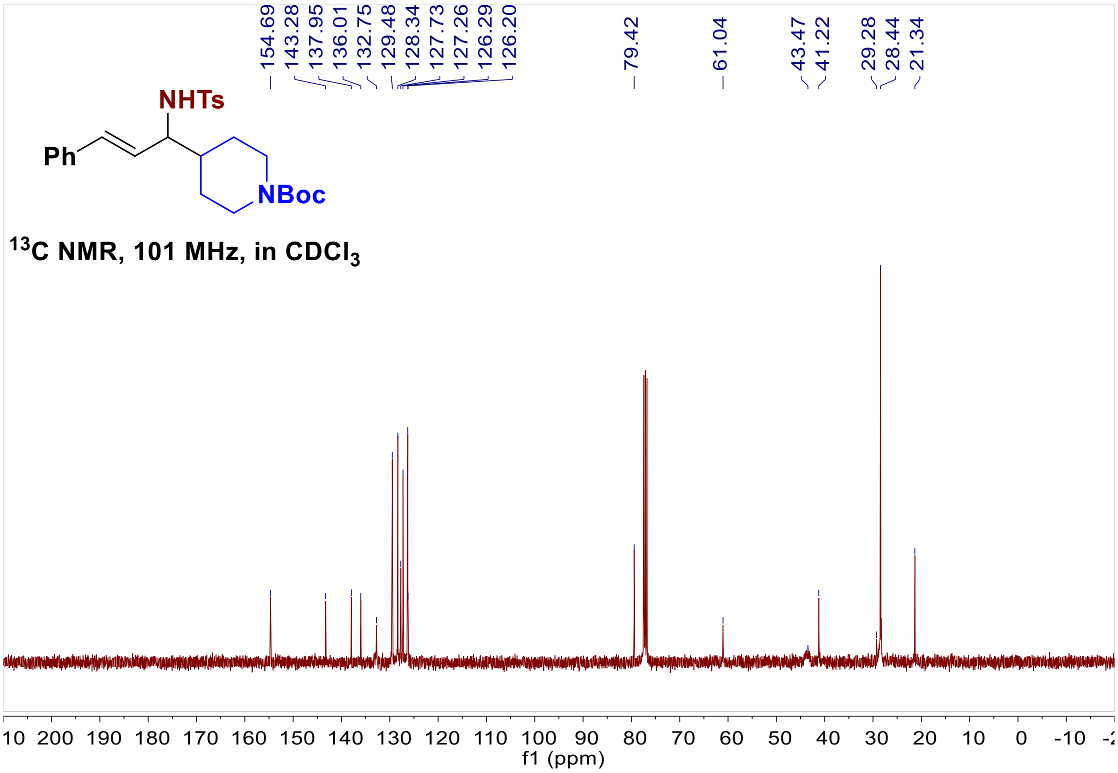

***tert*-butyl-(2*S*)-2-((*E*)-1-((4-methylphenyl)sulfonamido)-3-phenylallyl)pyrrolidine-1-carboxylate (28)**

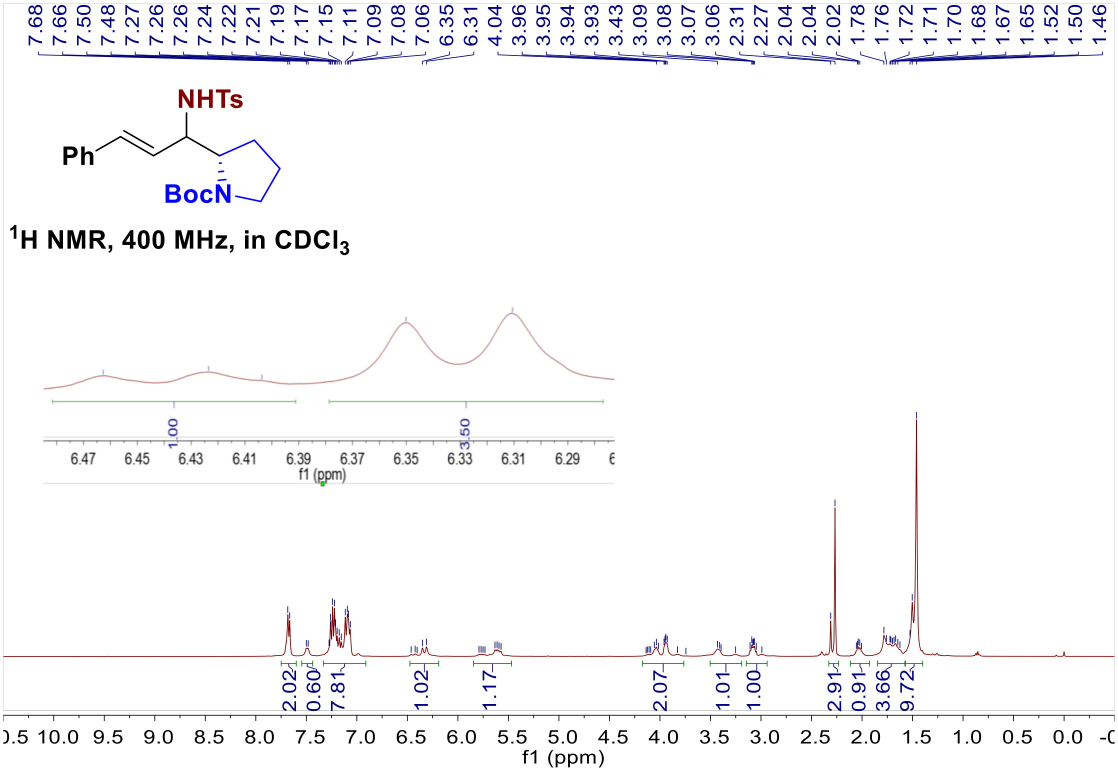

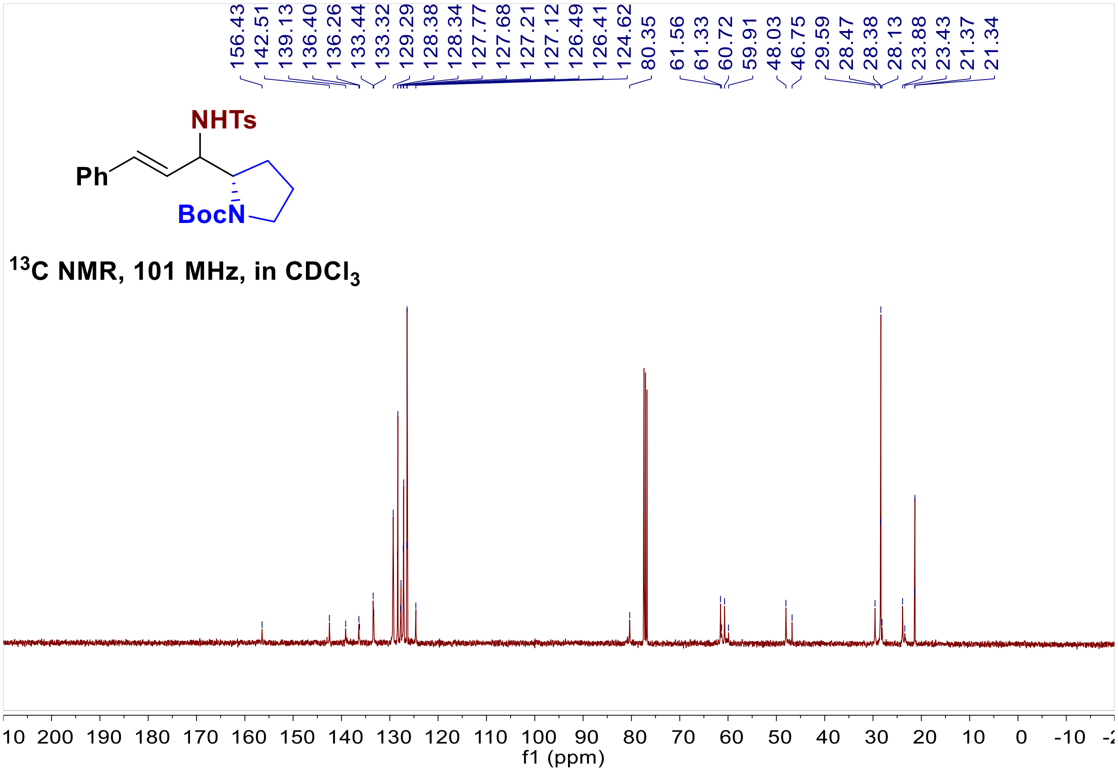

(*E*)-N-(1-cyclopentyl-3-phenylallyl)-4-methylbenzenesulfonamide (29)

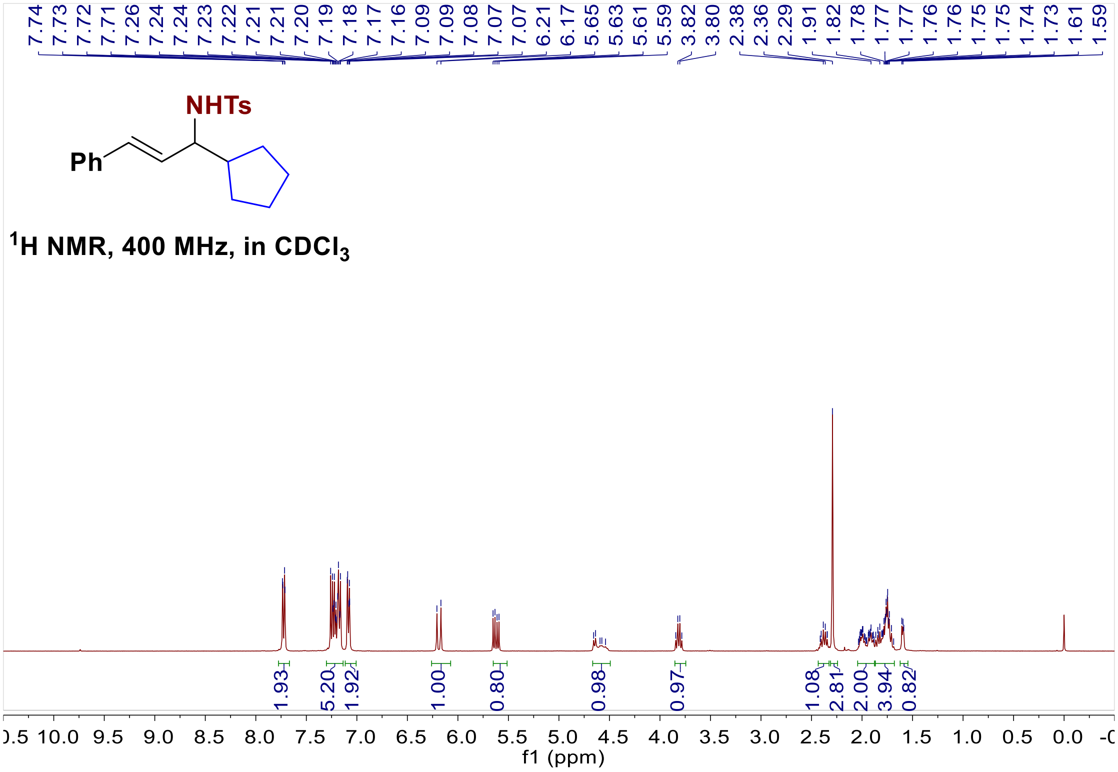

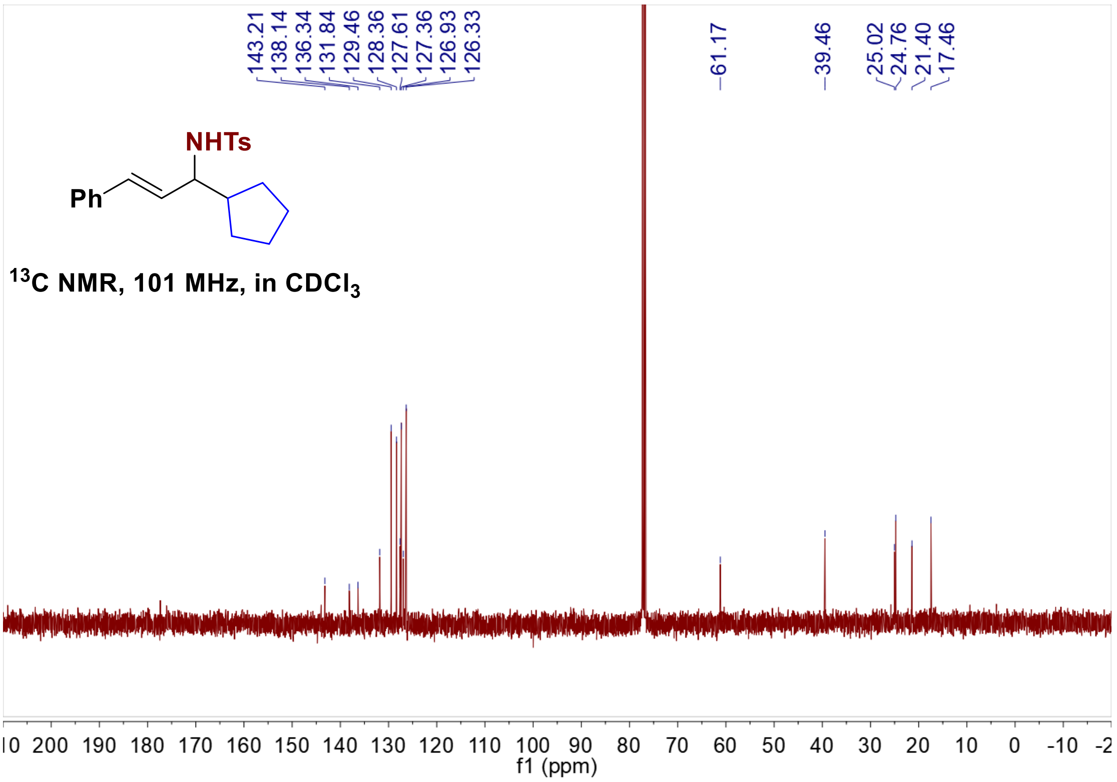

(*E*)-4-methyl-N-(3-phenyl-1-(tetrahydrofuran-2-yl)allyl)benzenesulfonamide (30)

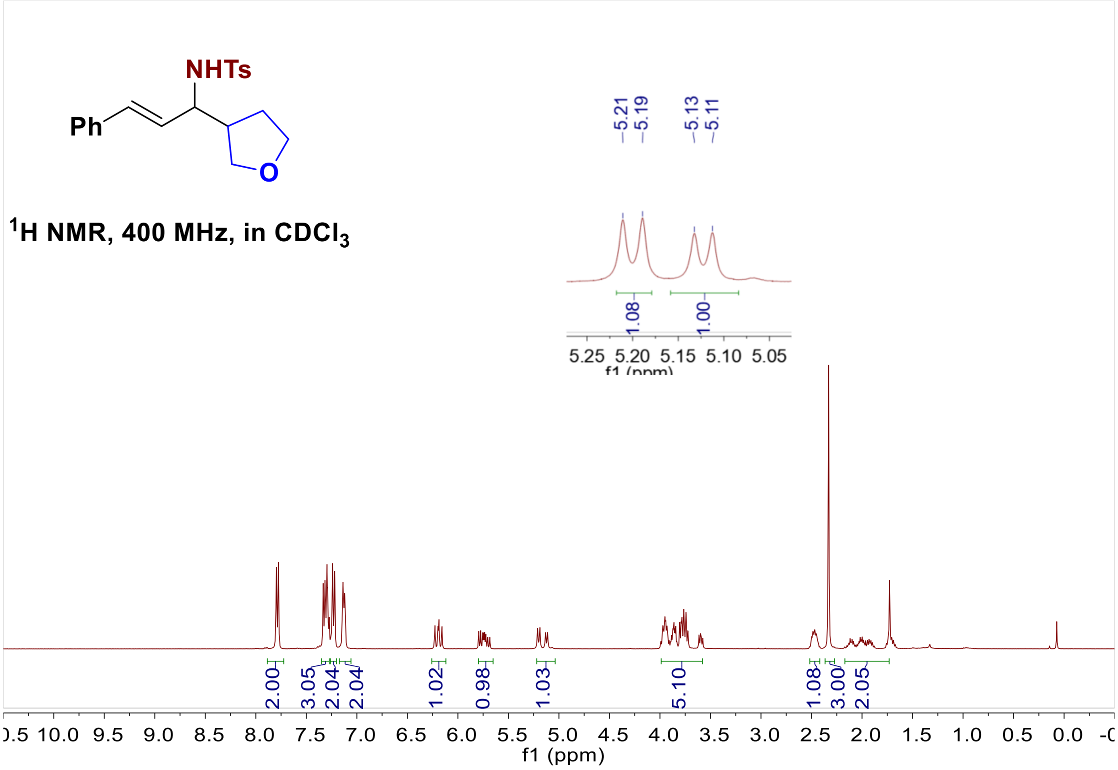

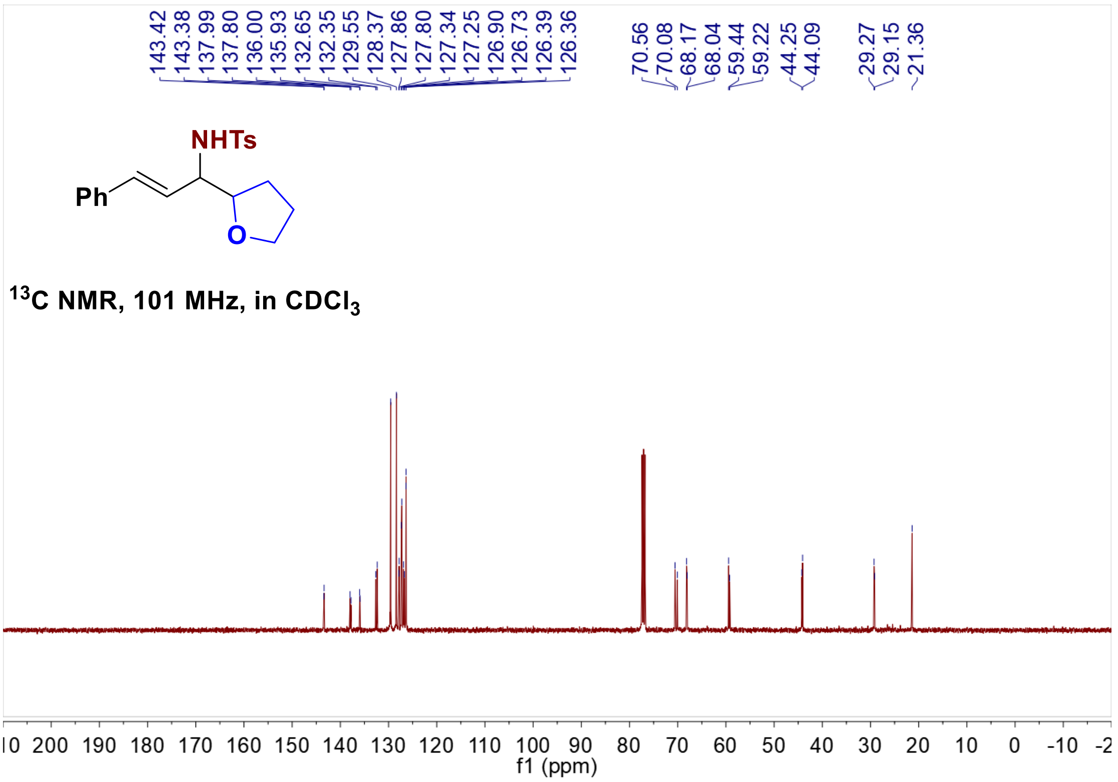

(*E*)-N-(1-cyclobutyl-3-phenylallyl)-4-methylbenzenesulfonamide (31)

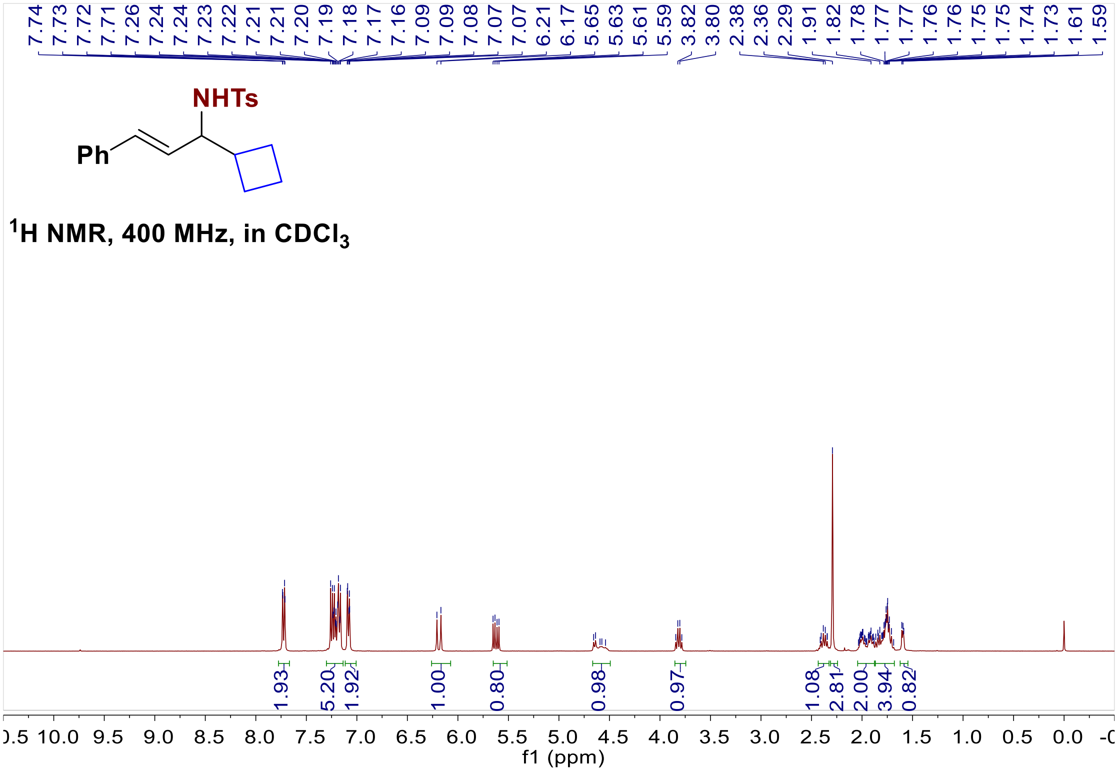

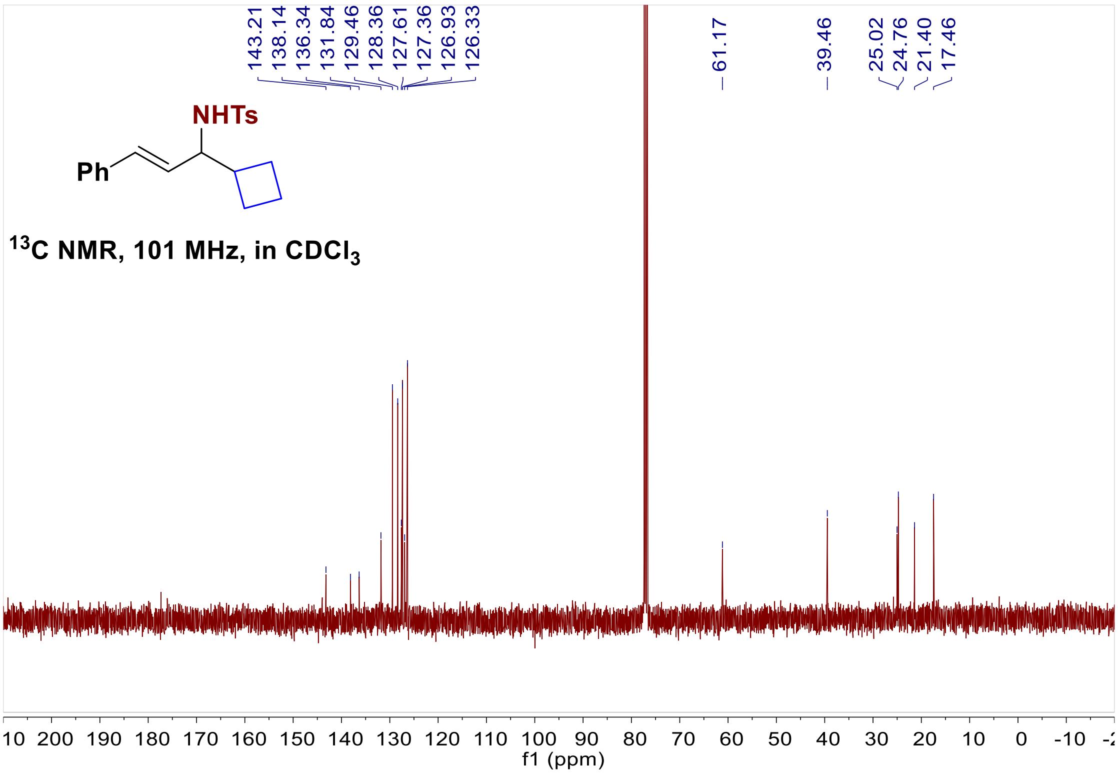

***tert*-butyl-(*E*)-3-(1-((4-methylphenyl)sulfonamido)-3-phenylallyl)azetidine-1-carboxylate (32)**

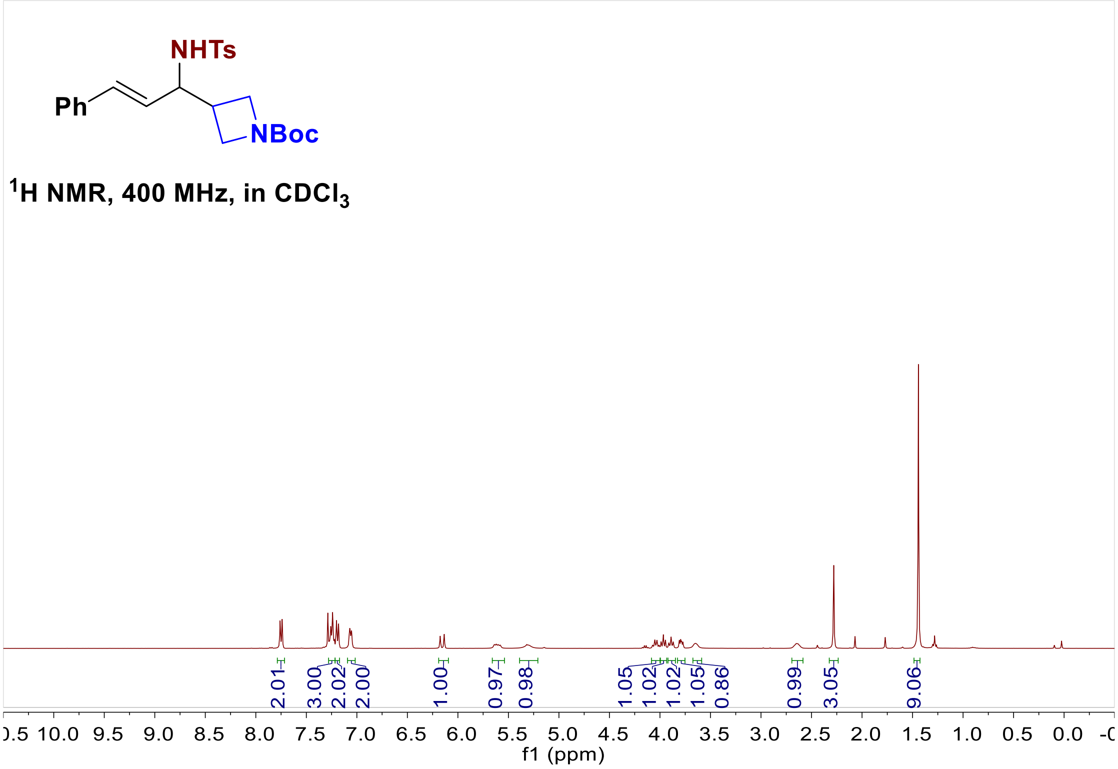

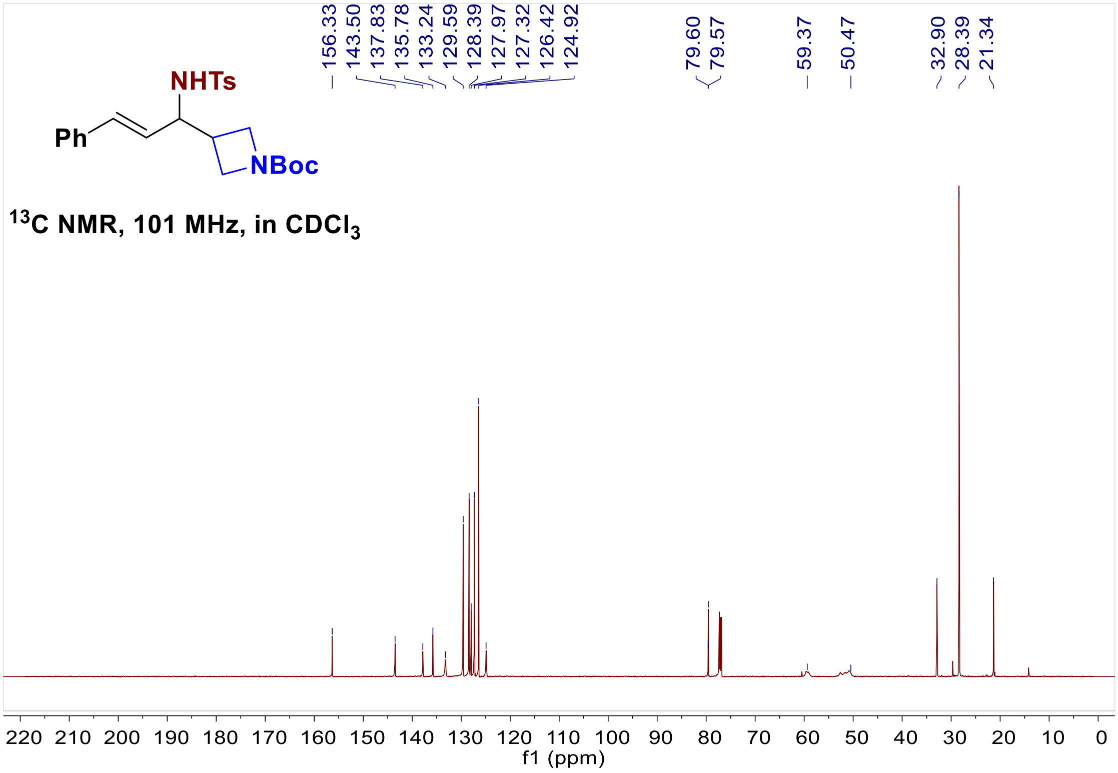

(*E*)-N-(7-hydroxy-1-phenylhept-1-en-3-yl)-4-methylbenzenesulfonamide (33)

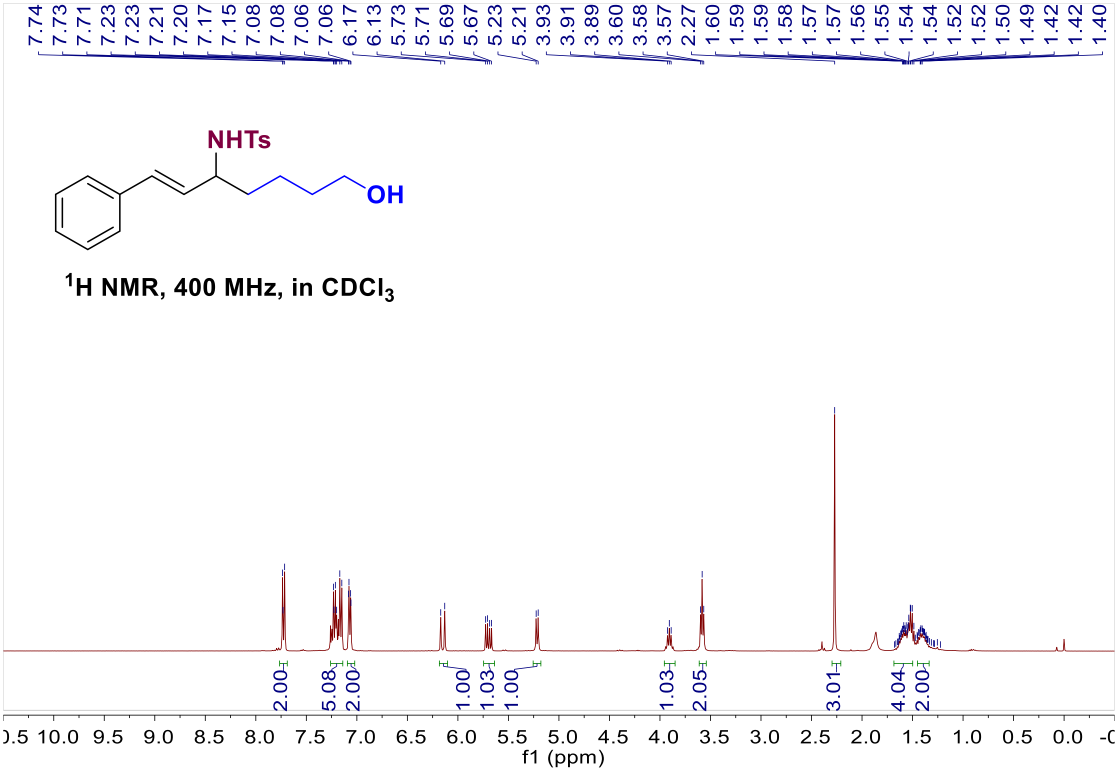

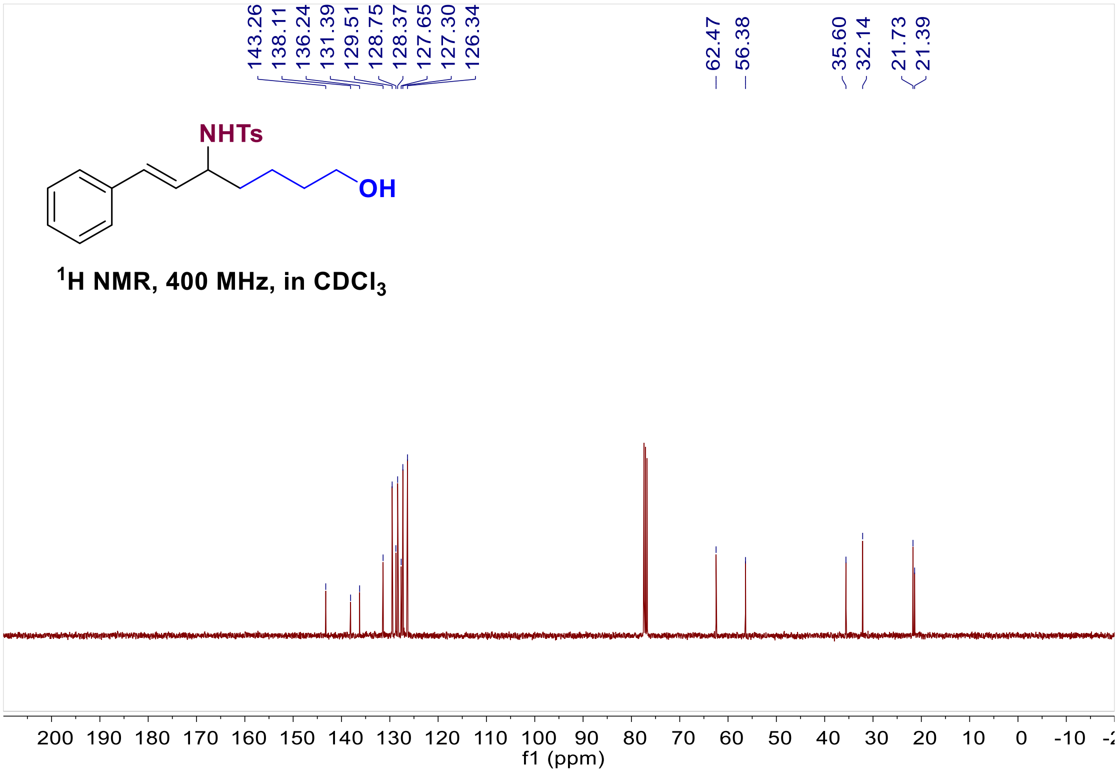

Isopropyl-(*E*)-3-(1-((4-methylphenyl)sulfonamido)-3-phenylallyl)bicyclo[1.1.1]pentane-1-carboxylate (35)

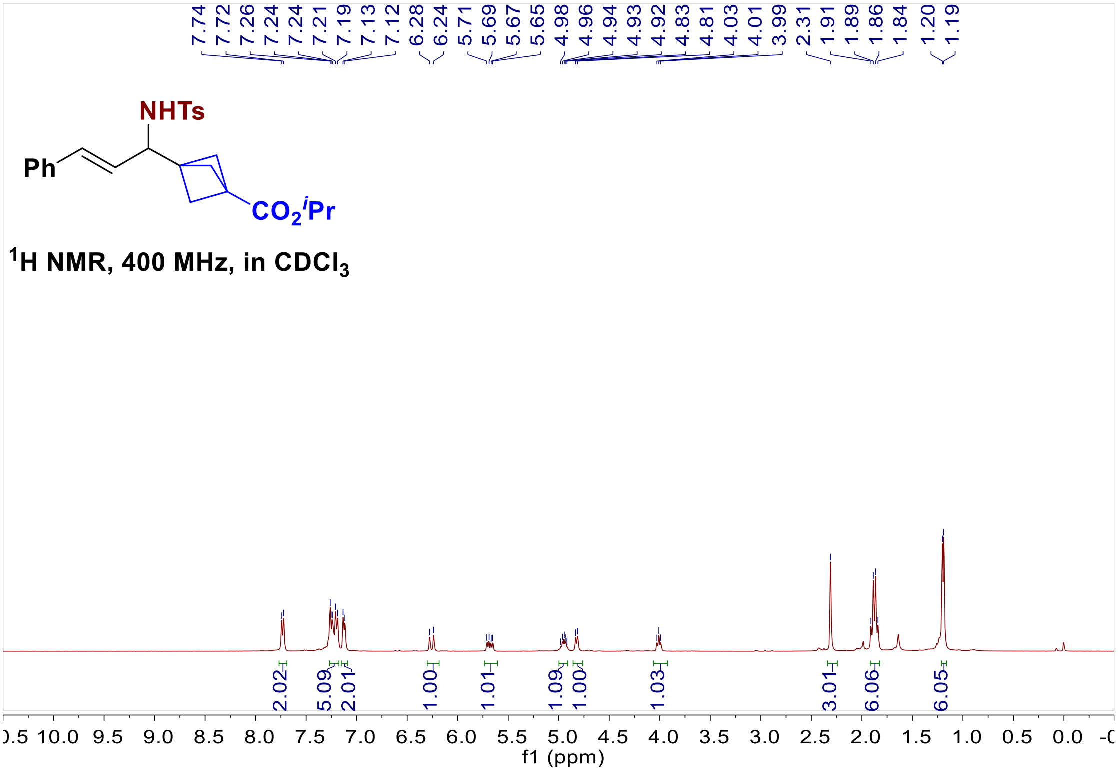

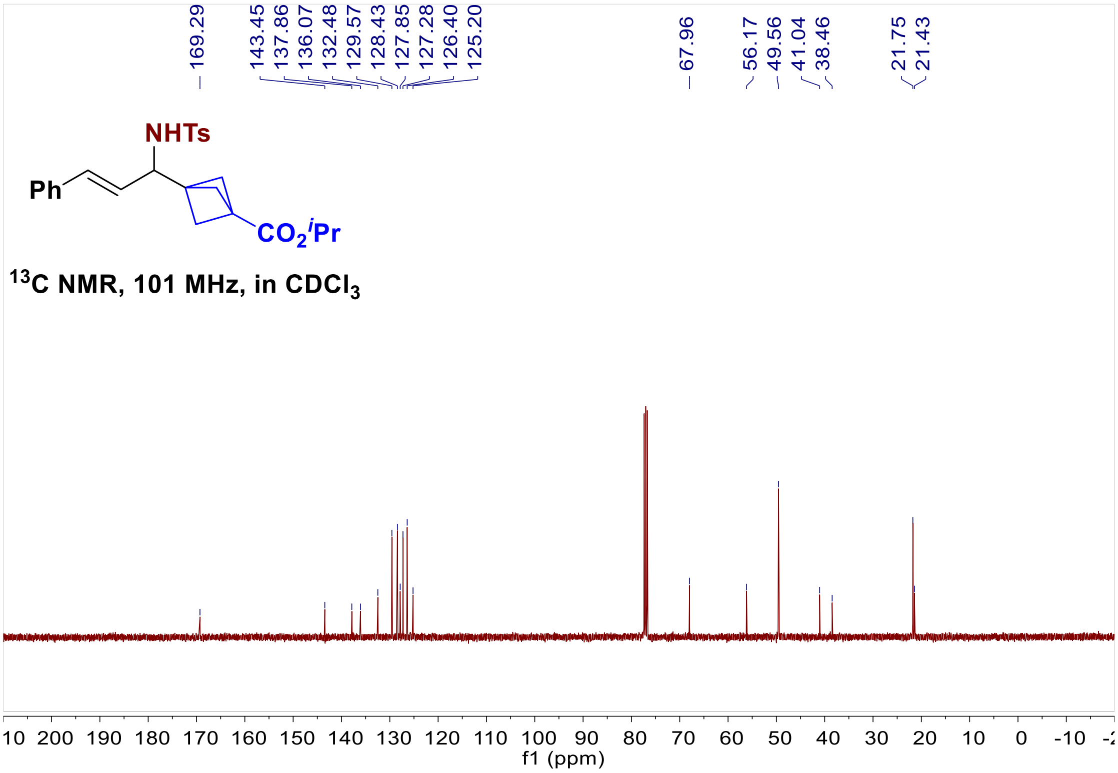

N-cinnamyl-4-methylbenzenesulfonamide (36)

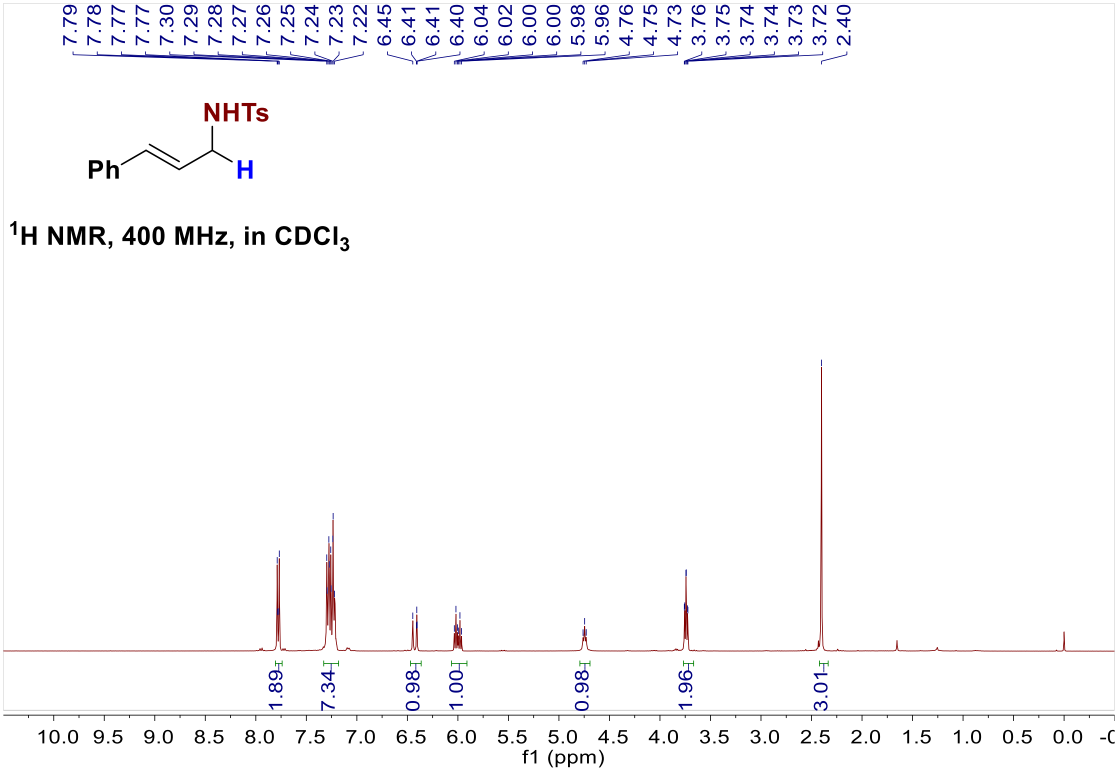

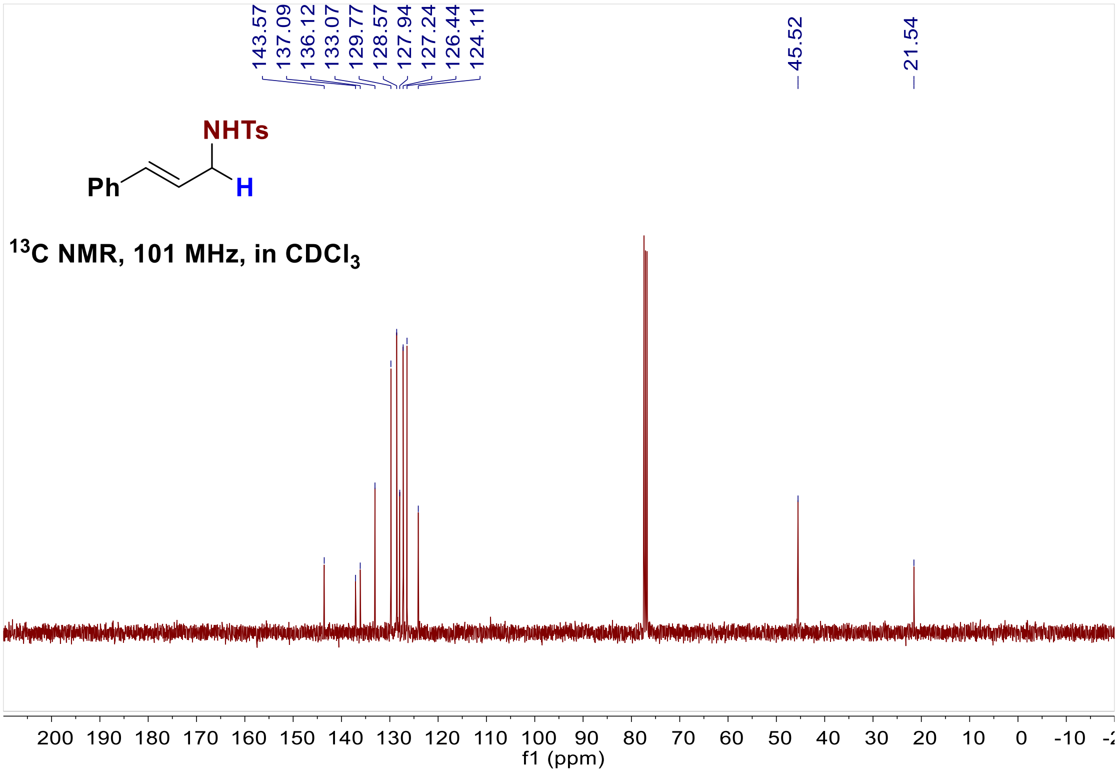

tert-butyl-(_E_)-4-(1-((4-methylphenyl)sulfonamido)-3-(3,4,5-trimethoxyphenyl)allyl)piperidine-1-carboxylate (37)

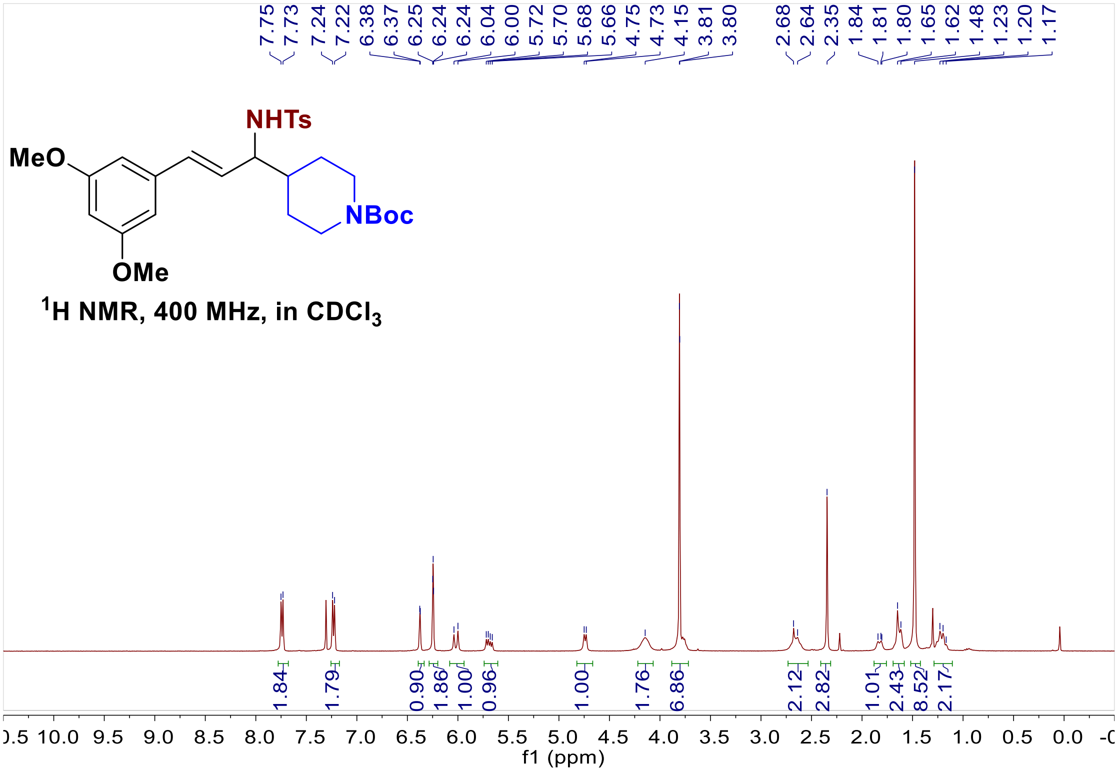

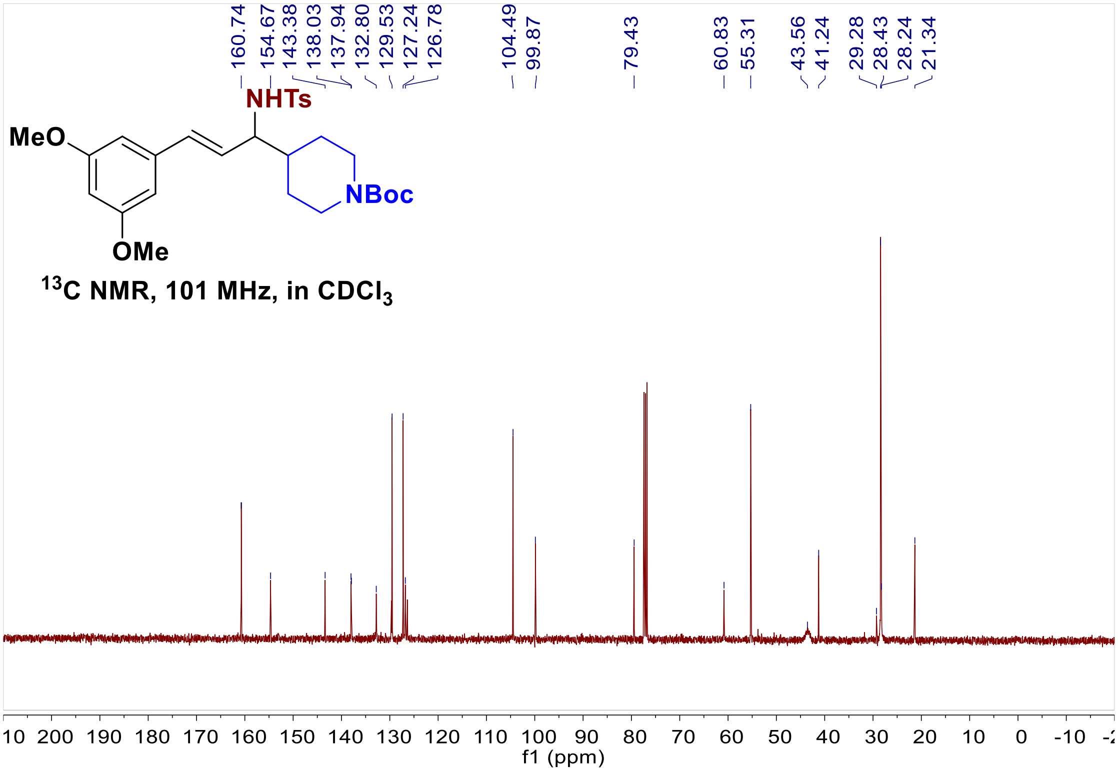

***tert*-butyl-(*E*)-4-(3-(3,5-dichlorophenyl)-1-((4-methylphenyl)sulfonamido)allyl)piperidine-1-carboxylate (38)**

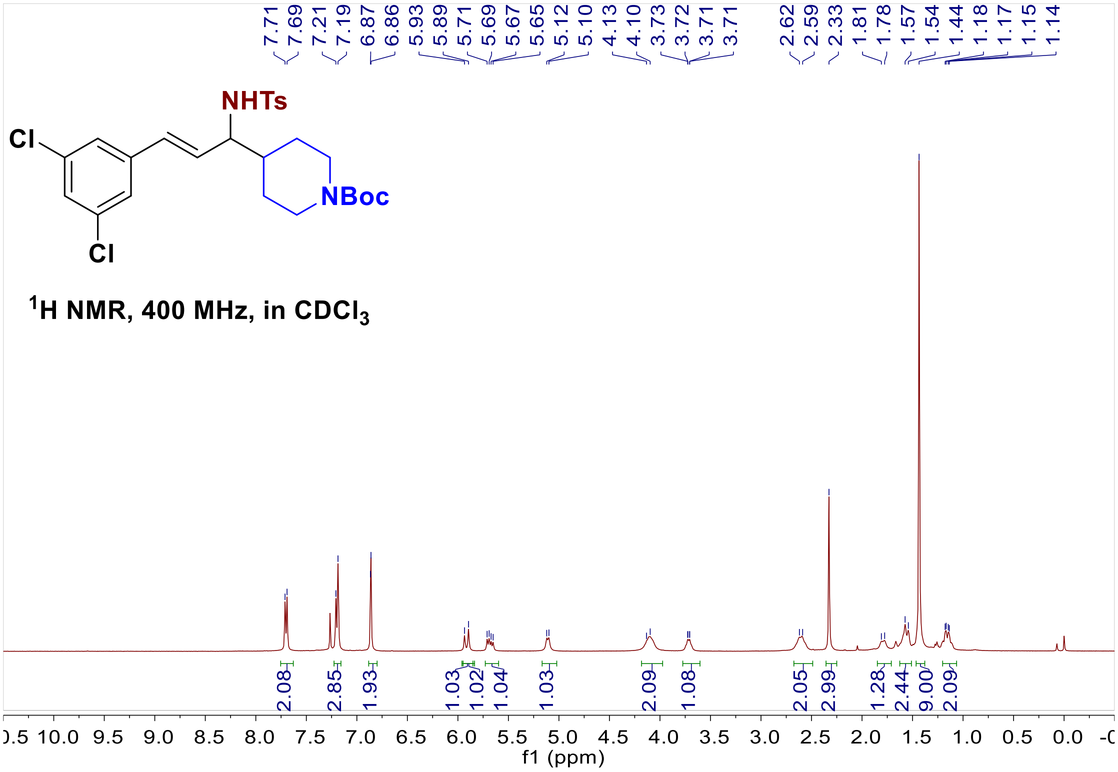

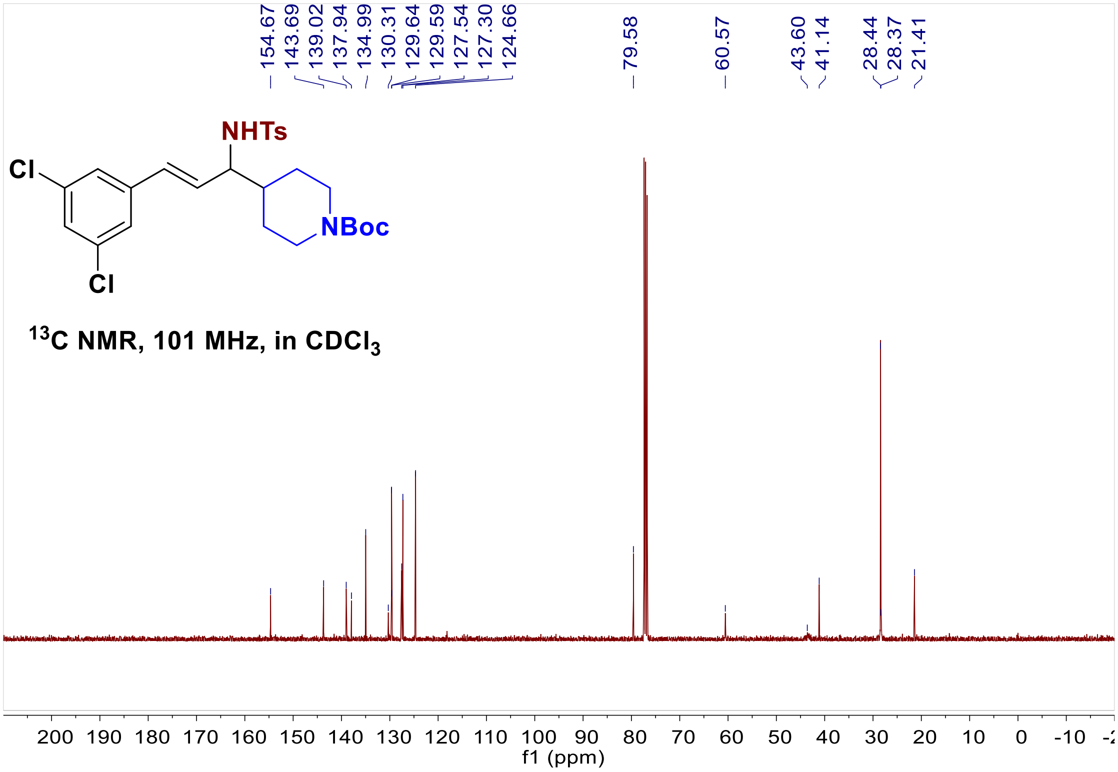

***tert*-butyl-(*E*)-4-(3-(4-fluorophenyl)-1-((4-methylphenyl)sulfonamido)allyl)piperidine-1-carboxylate (39)**

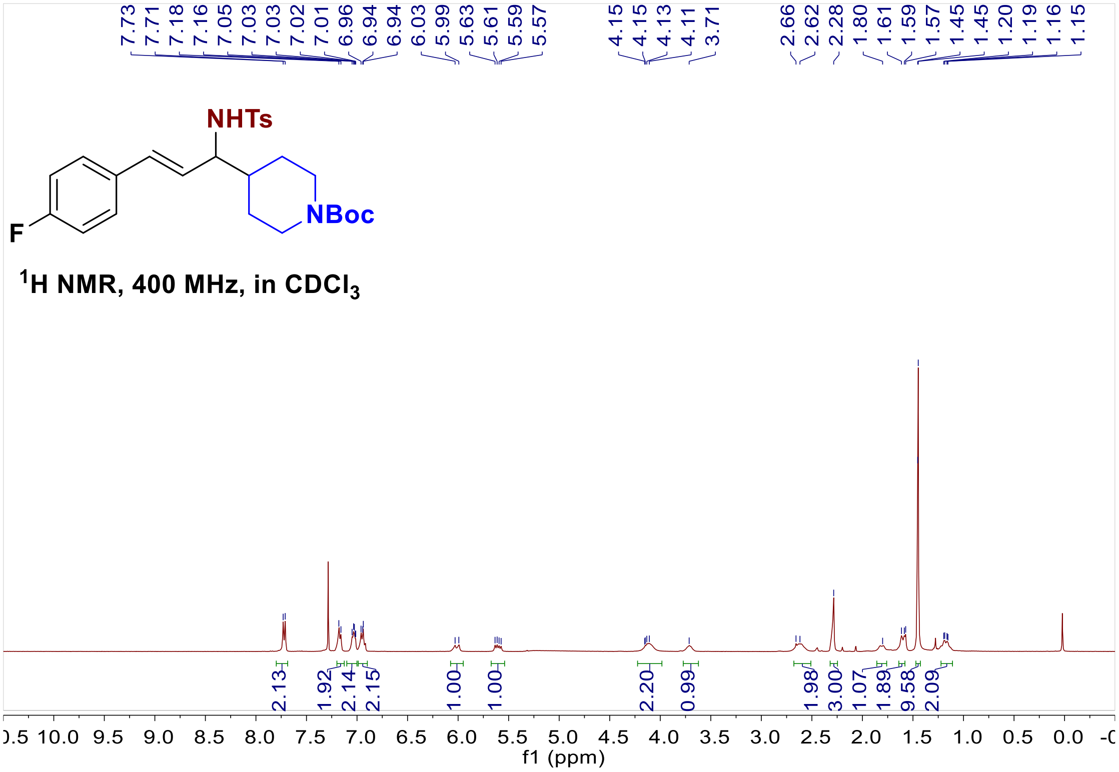

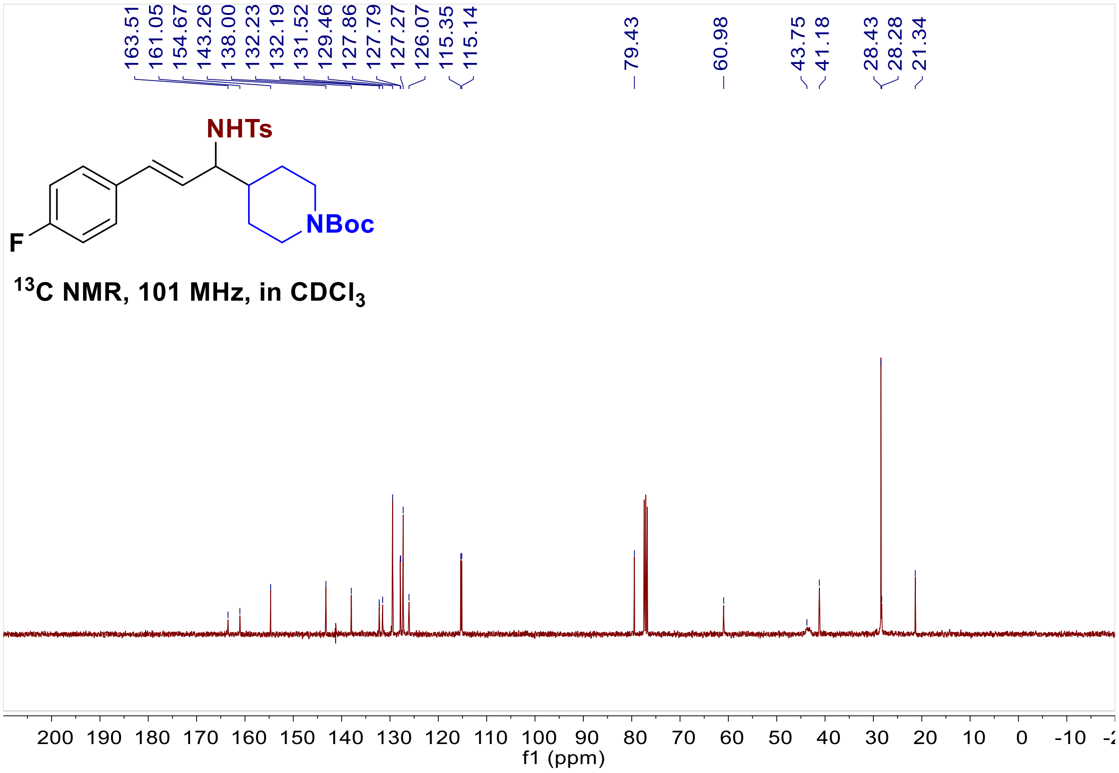

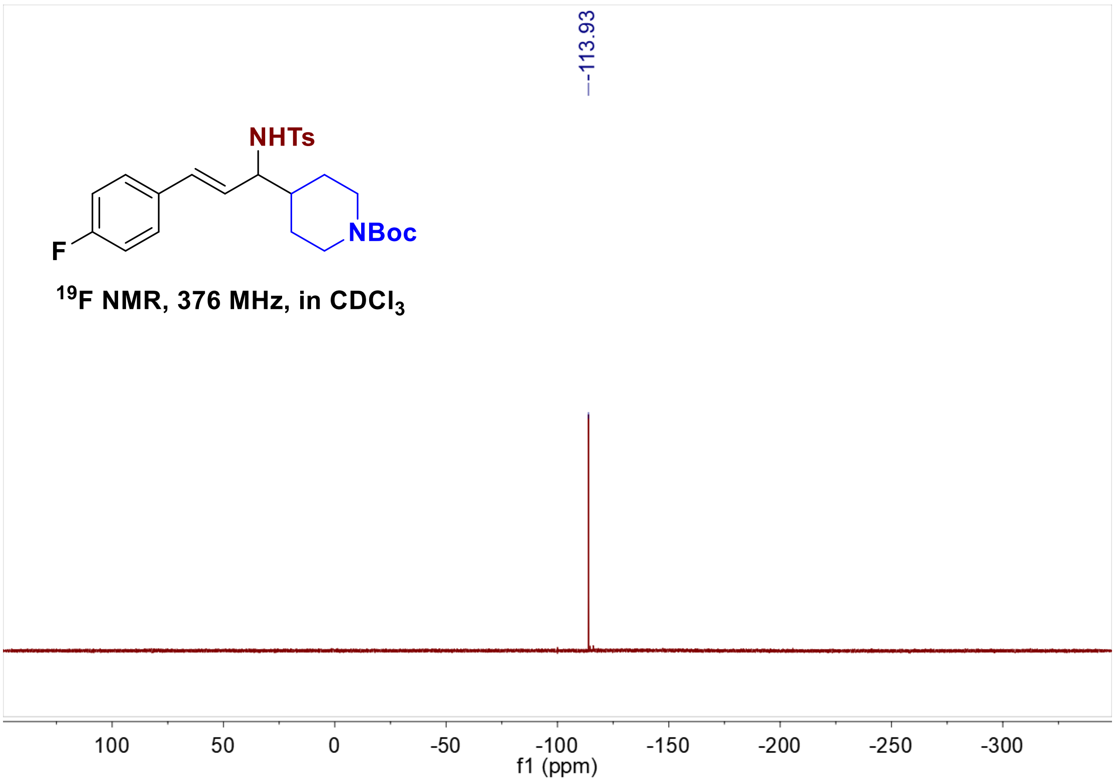

-113.93
NHTs
F
NBoc
19F NMR, 376 MHz, in CDCl3
100
50
0
-50
-100
-150
-200
-250
-300
f1 (ppm)

***tert*-butyl-(*E*)-4-(1-((4-methylphenyl)sulfonamido)-3-(4-(4,4,5,5-tetramethyl-1,3,2-dioxaborolan-2-yl)phenyl)allyl)piperidine-1-carboxylate (40)**

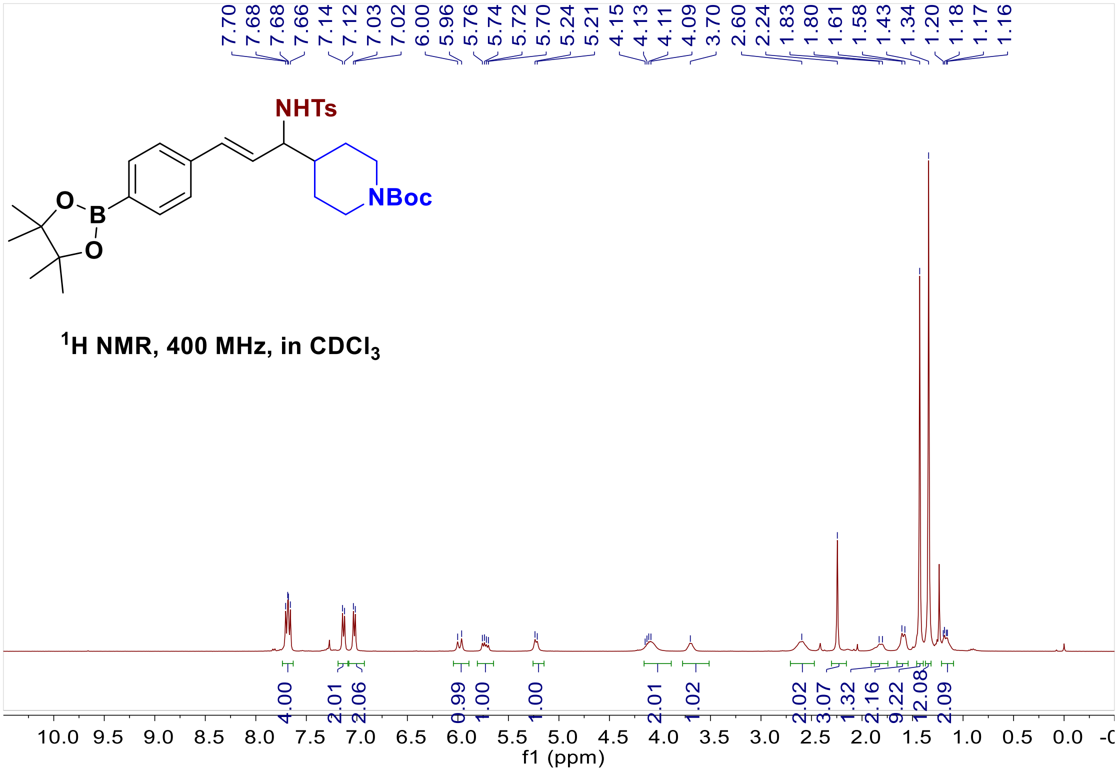

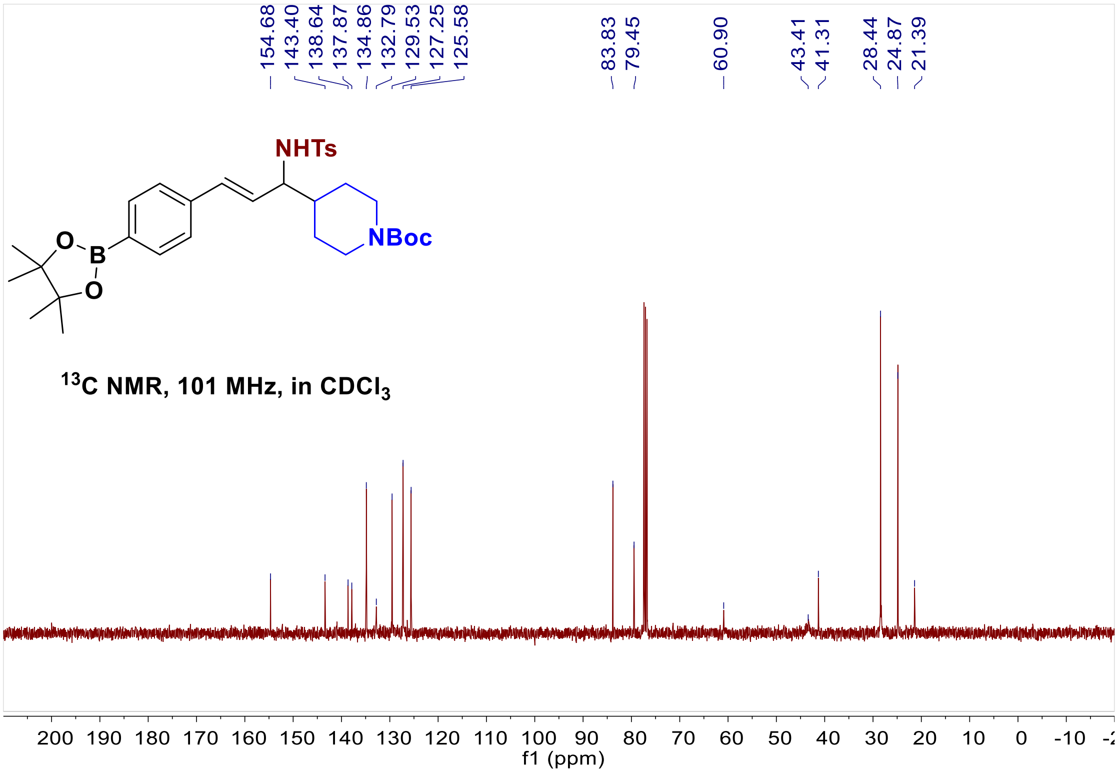

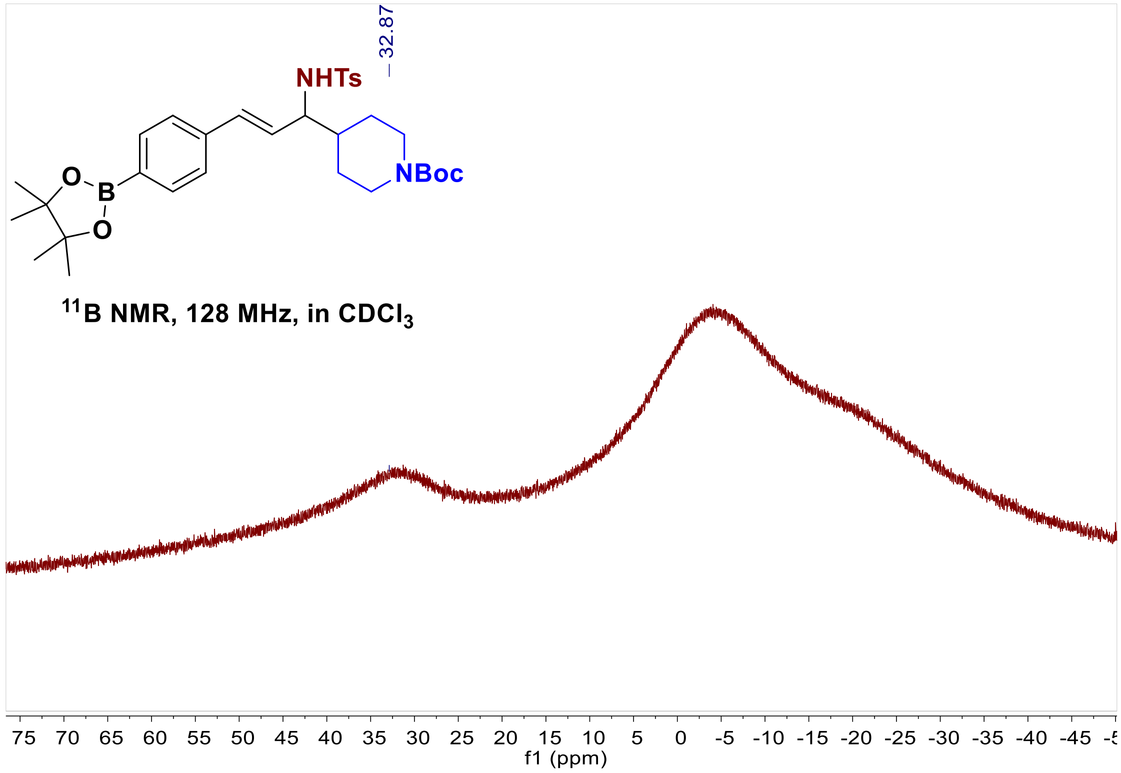

32.87
NHTs
NBoc
O
B
O
11B NMR, 128 MHz, in CDCl3
75 70 65 60 55 50 45 40 35 30 25 20 15 10 5 0 -5 -10 -15 -20 -25 -30 -35 -40 -45
f1 (ppm)

***tert*-butyl-(*E*)-4-(1-((4-methylphenyl)sulfonamido)-3-(naphthalen-2-yl)allyl)piperidine-1-carboxylate (41)**

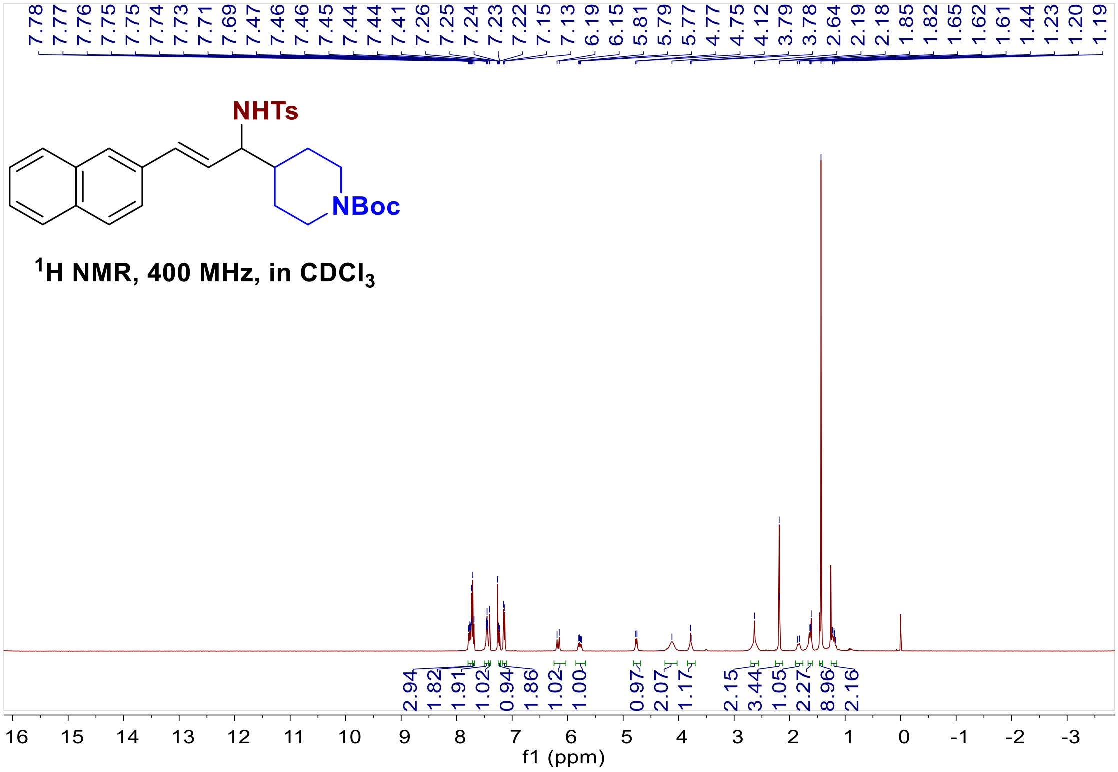

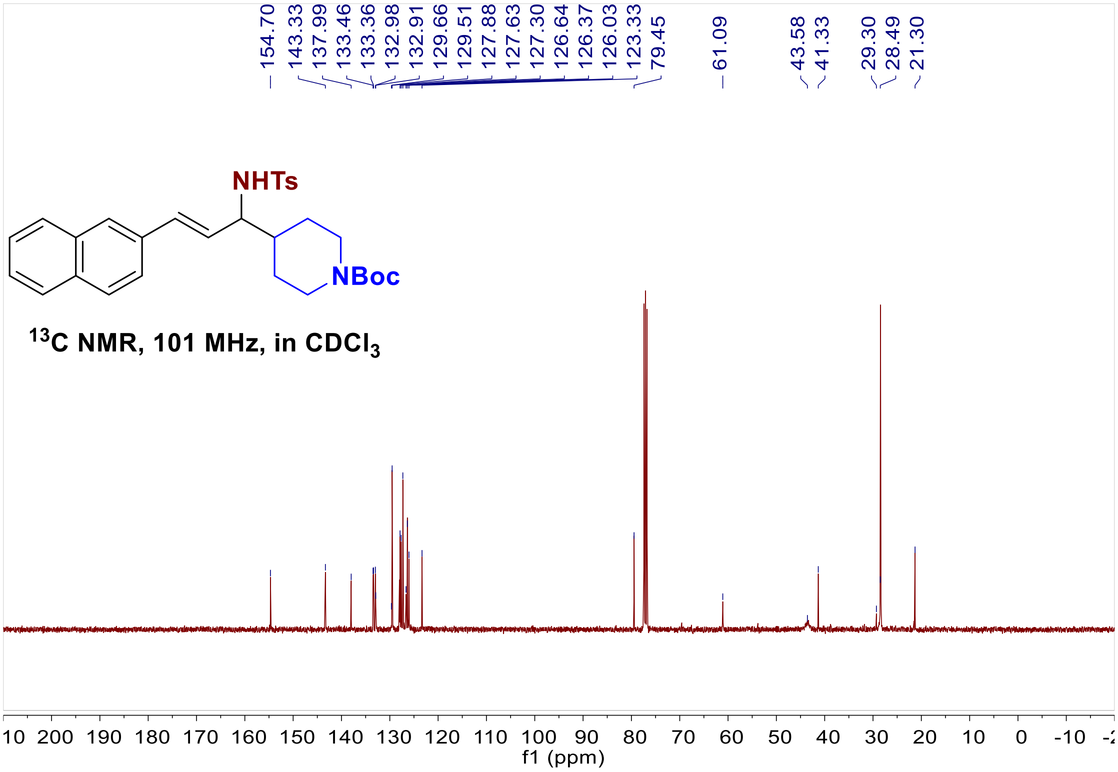

tert-butyl-(_E_)-4-(3-(5-methylfuran-2-yl)-1-((4-methylphenyl)sulfonamido)allyl)piperidine-1-carboxylate (42)

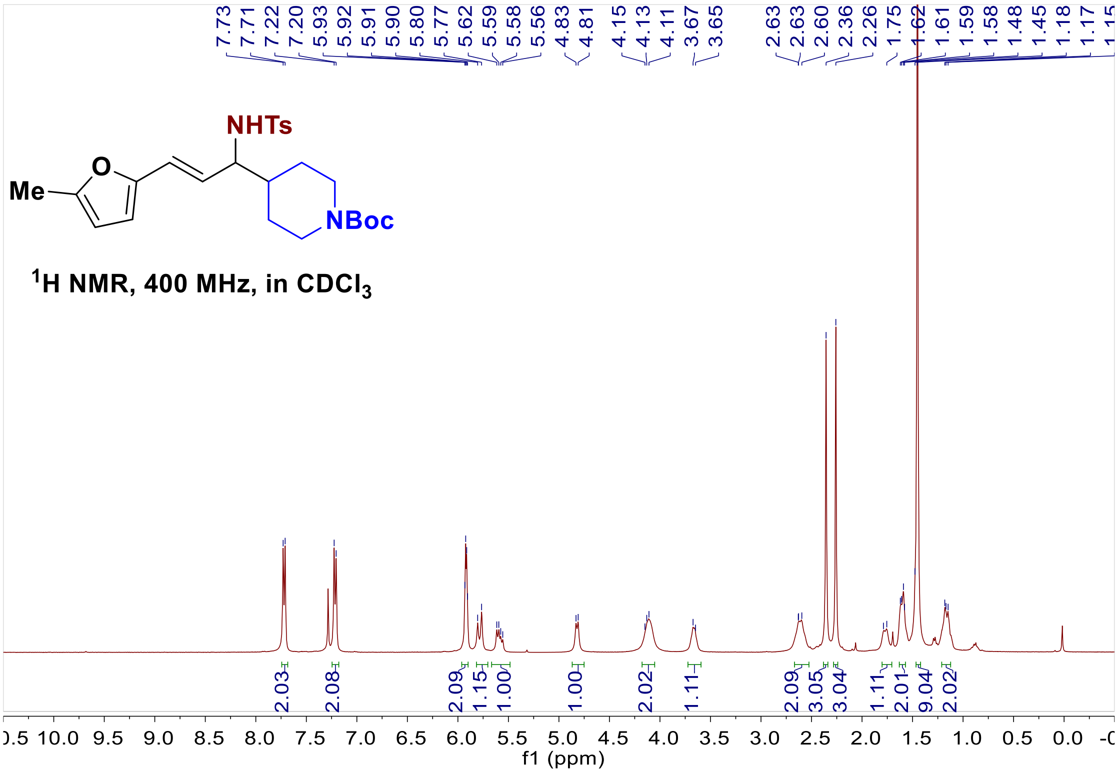

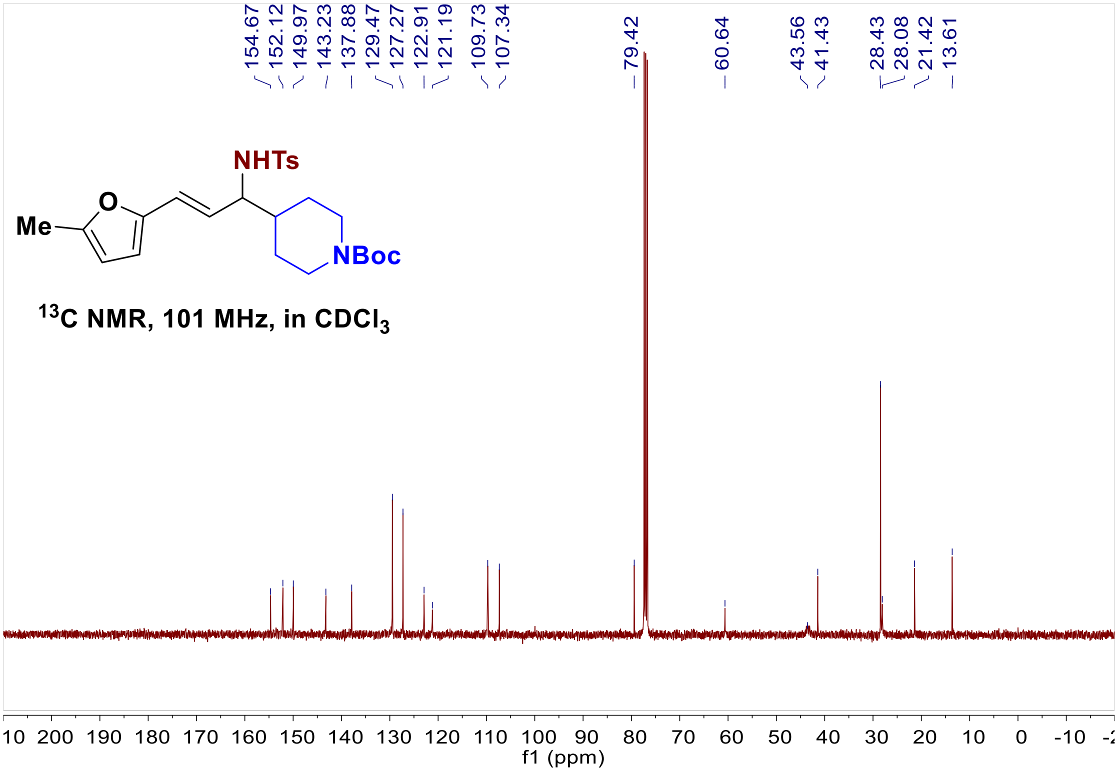

***tert*-butyl-(*E*)-4-(1-((4-methylphenyl)sulfonamido)-3-(thiophen-2-yl)allyl)piperidine-1-carboxylate (43)**

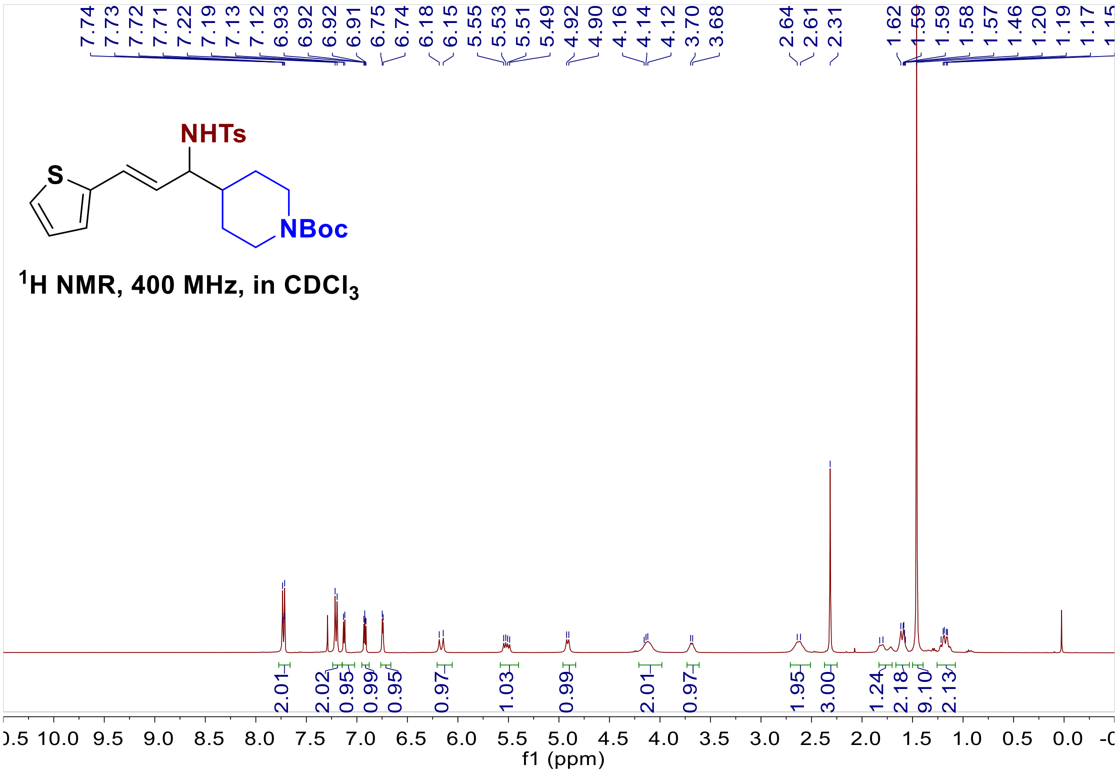

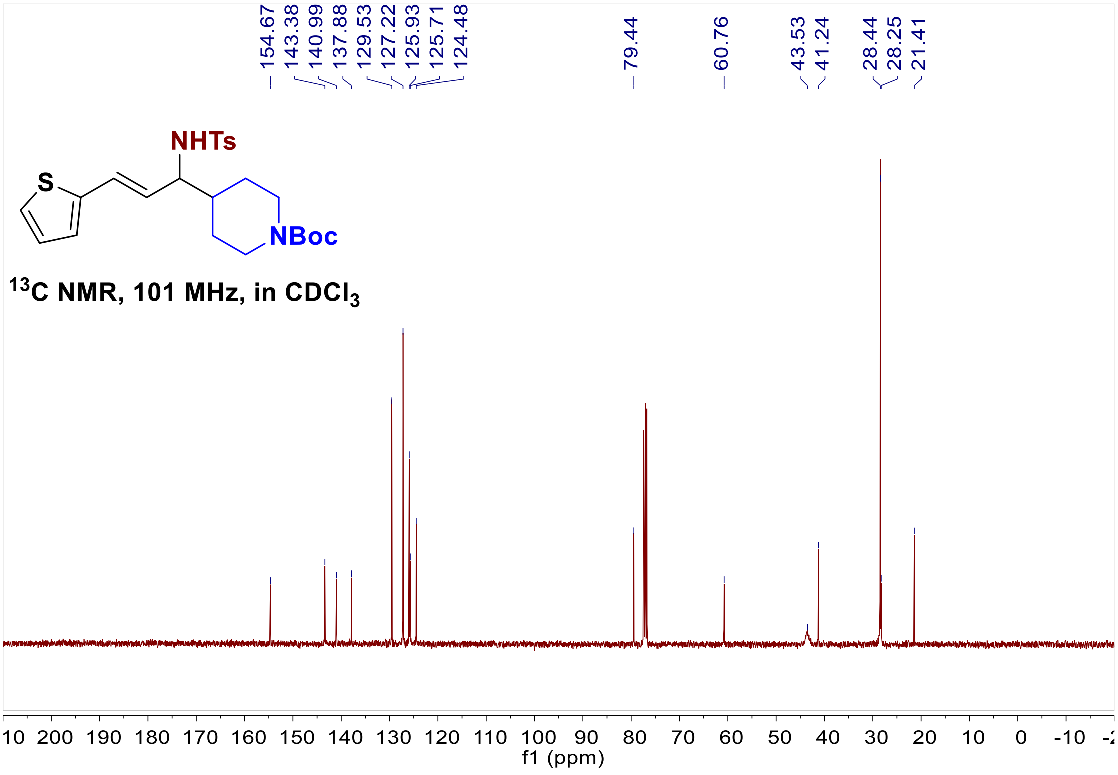

***tert*-butyl-(*E*)-4-(1-((4-methylphenyl)sulfonamido)-3-(thiophen-2-yl)allyl)piperidine-1-carboxylate (44)**

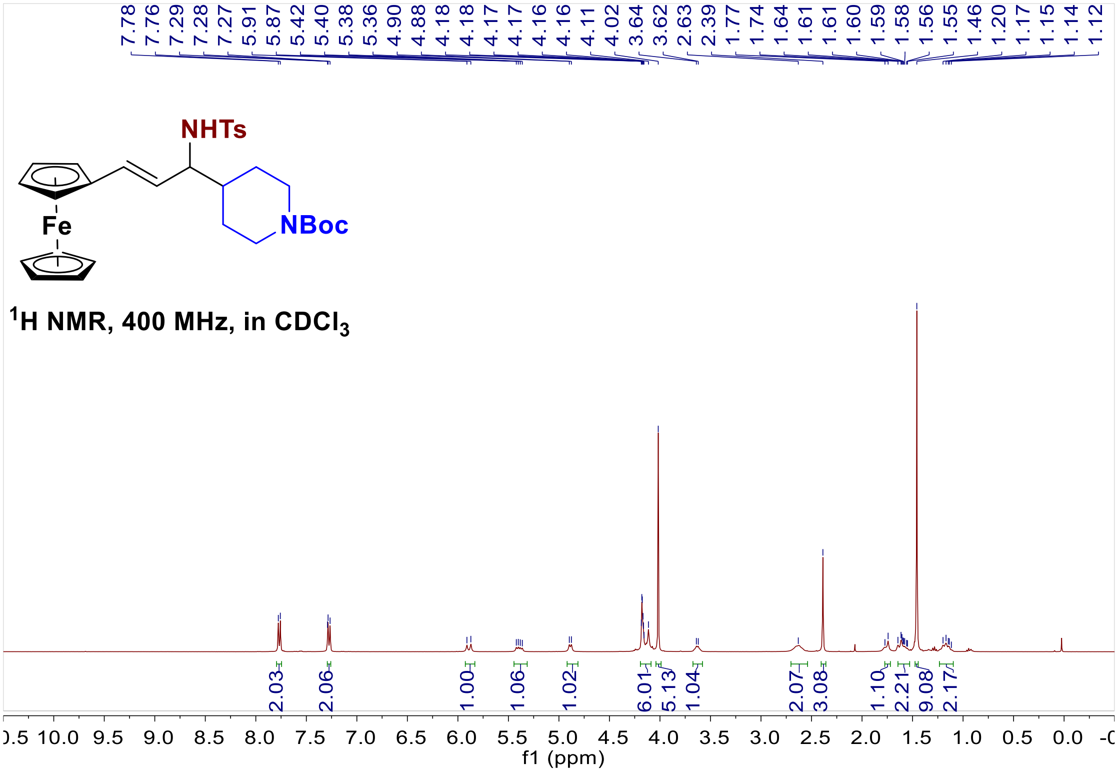

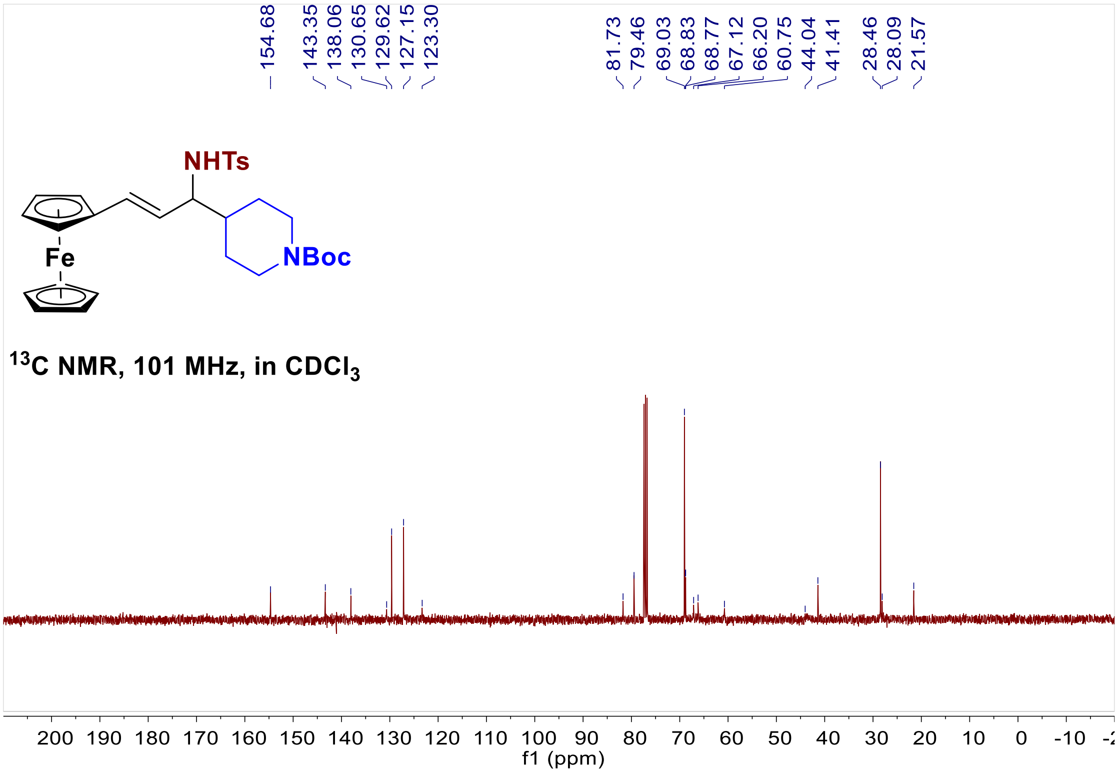

***tert*-butyl-4-((2*E*, 4*E*)-1-((4-methylphenyl)sulfonamido)-5-phenylpenta-2,4-dien-1-yl)piperidine-1-carboxylate (45)**

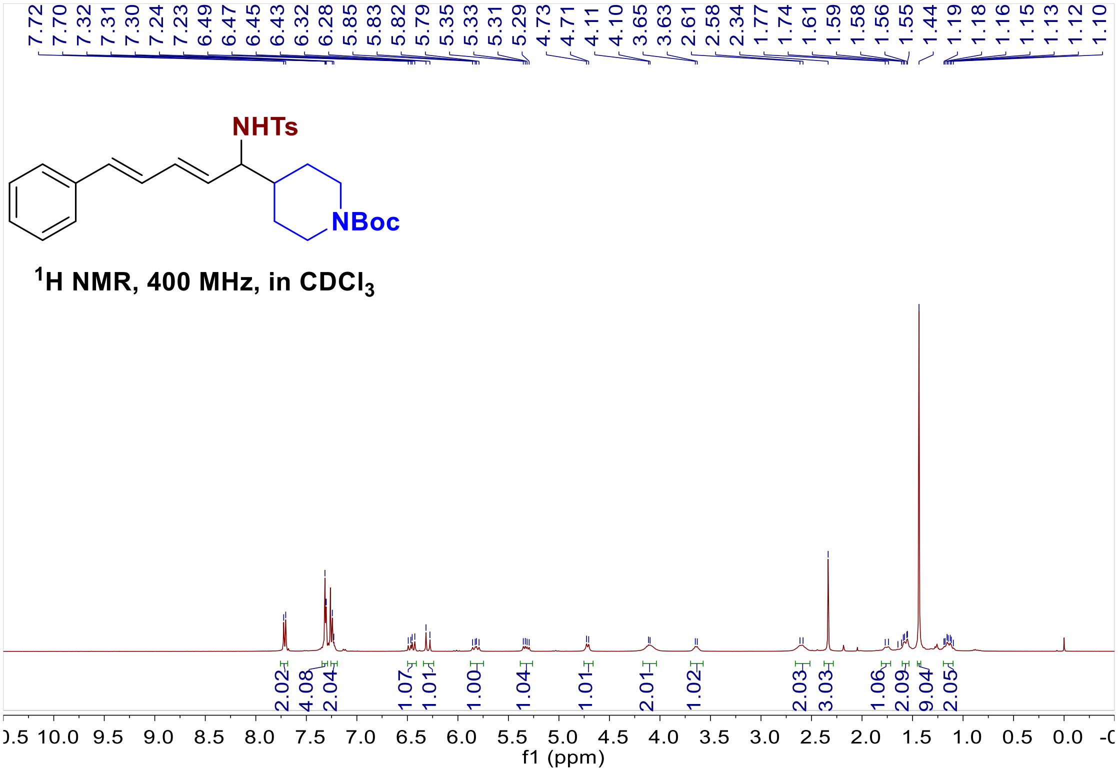

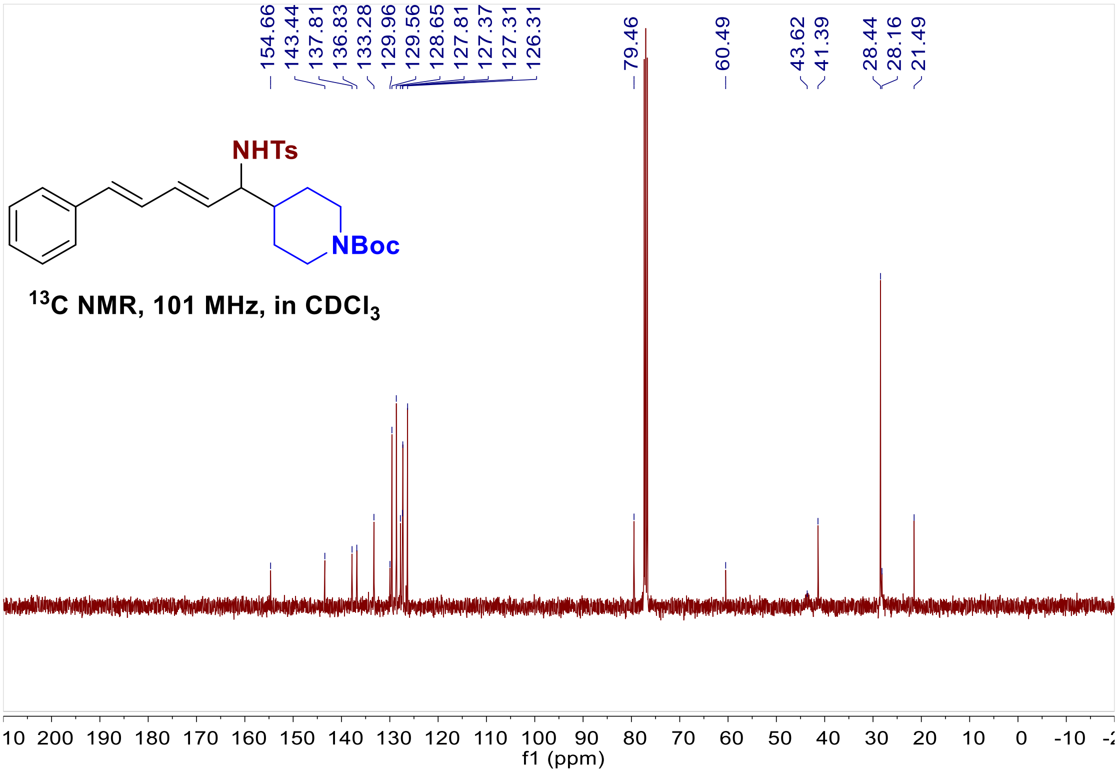

tert-butyl-(_E_)-4-(3-(9H-carbazol-9-yl)-1-((4-methylphenyl)sulfonamido)allyl)piperidine-1-carboxylate (46)

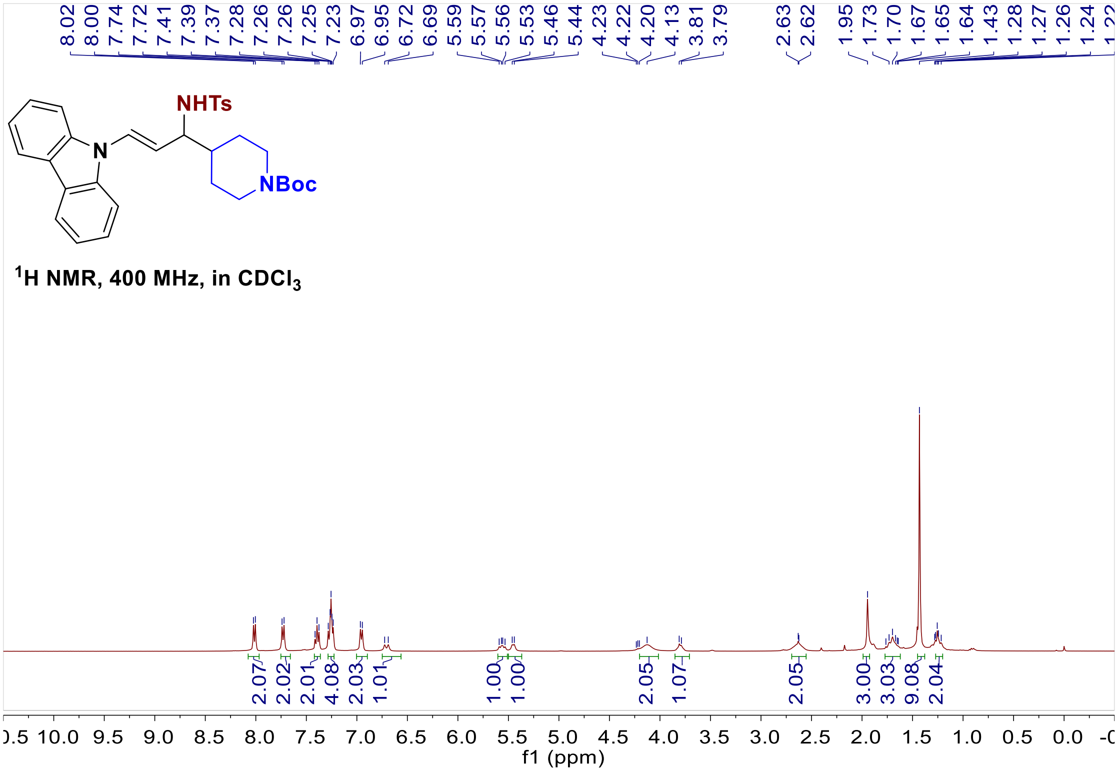

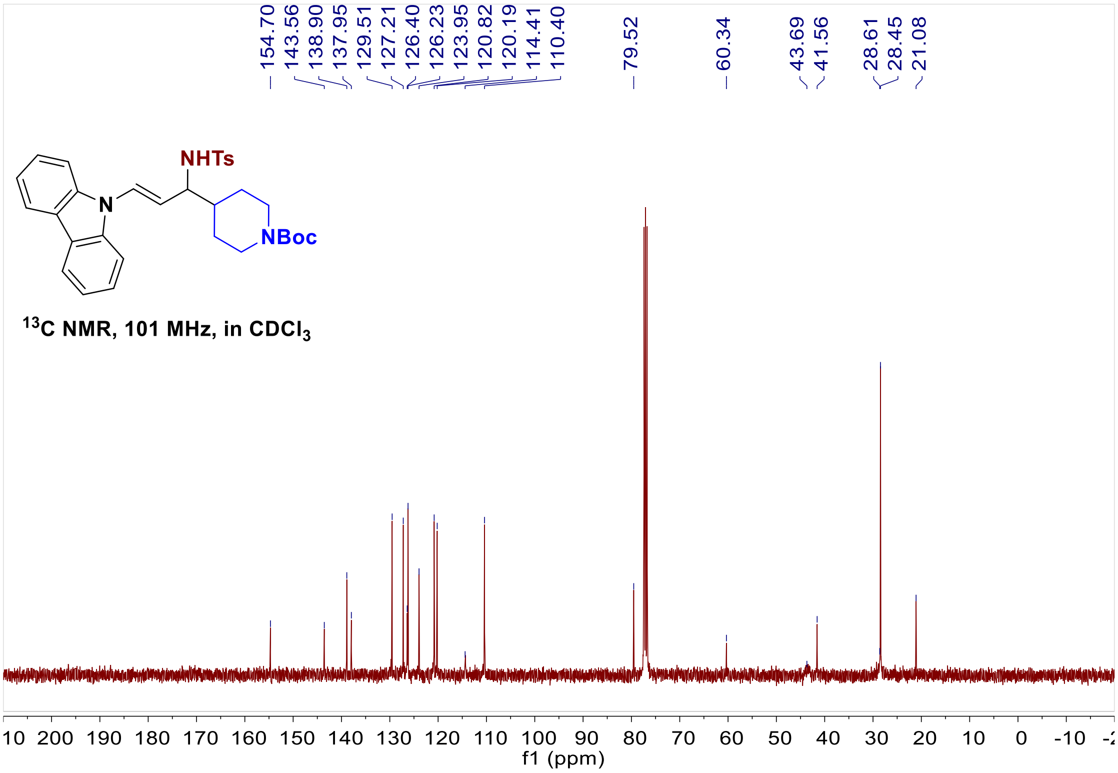

***tert*-butyl-(*E*)-4-(1-((4-methylphenyl)sulfonamido)-4-(trimethylsilyl)but-3-en-1-yl)piperidine-1-carboxylate (47)**

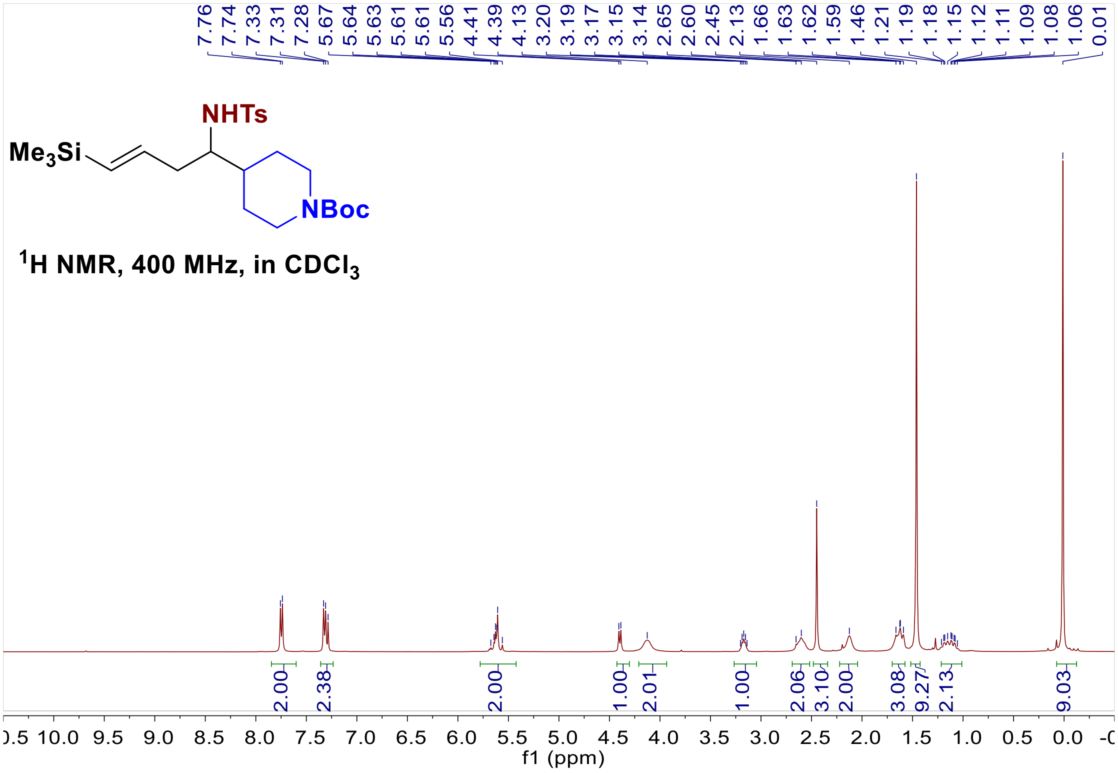

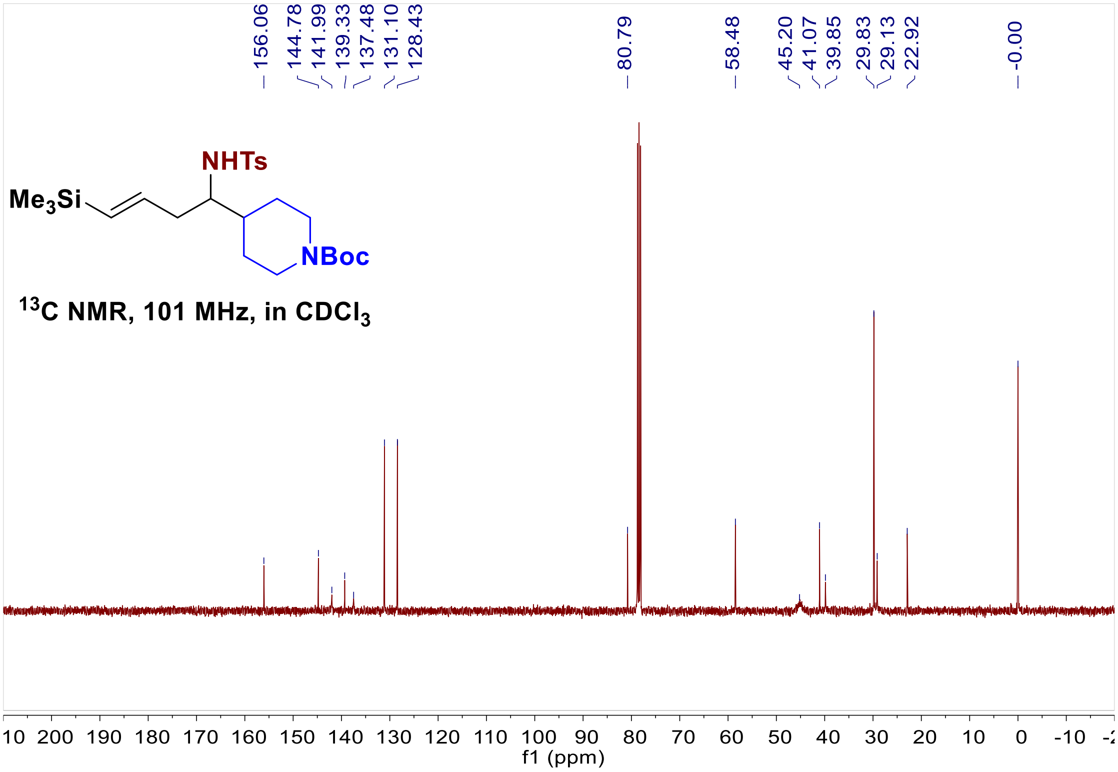

***tert*-butyl-(*E*)-4-(1-((4-methylphenyl)sulfonamido)-4-(trimethylsilyl)but-2-en-1-yl)piperidine-1-carboxylate (47′)**

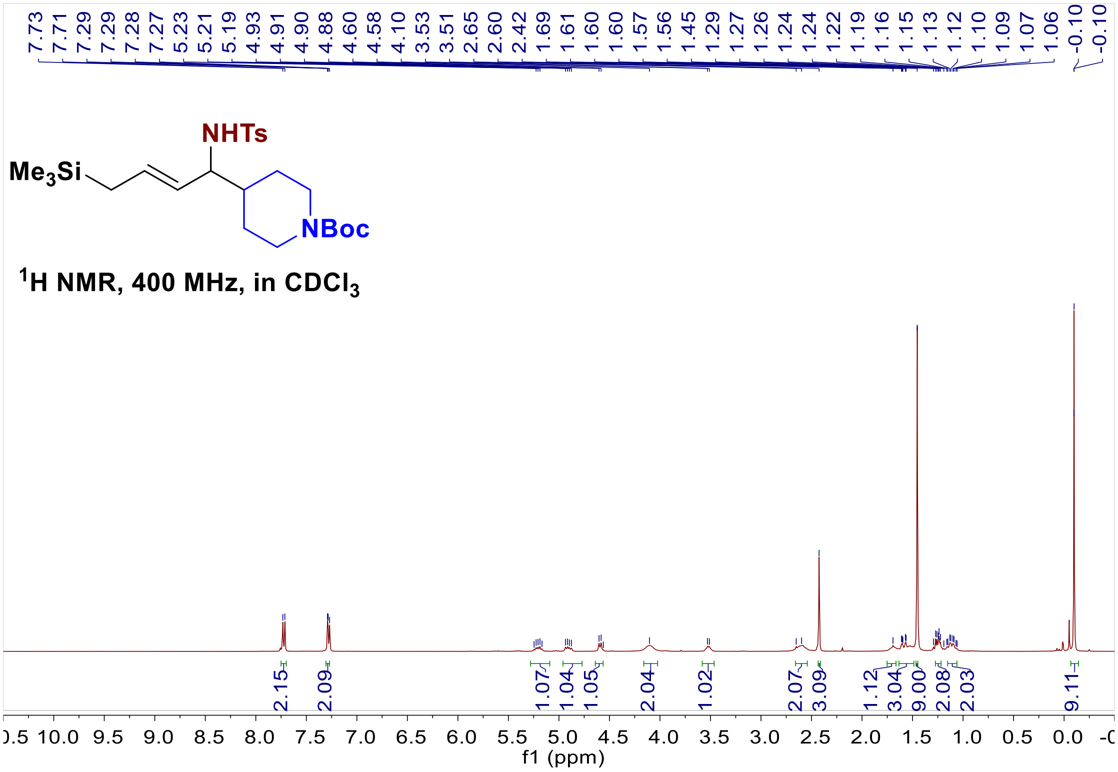

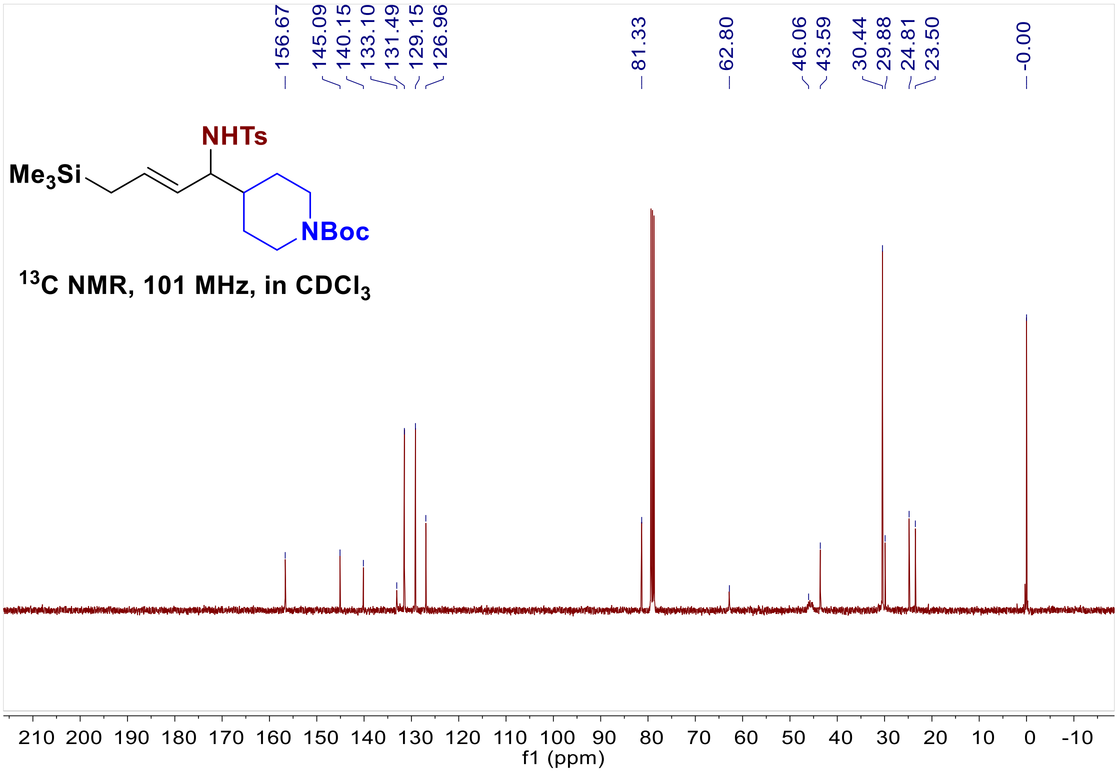

***tert*-butyl-(*E*)-4-(1-((4-methylphenyl)sulfonamido)-5-phenylpent-3-en-1-yl)piperidine-1-carboxylate (mixture A/H= 3.8:1) (48 and 48′)**

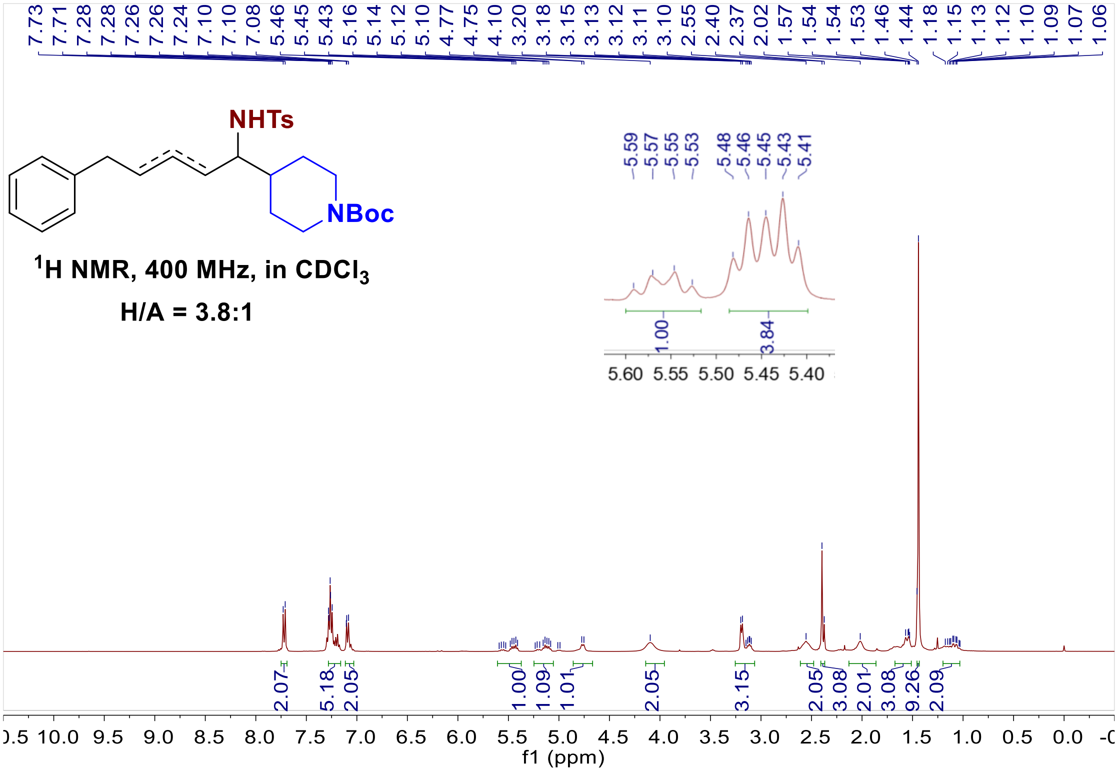

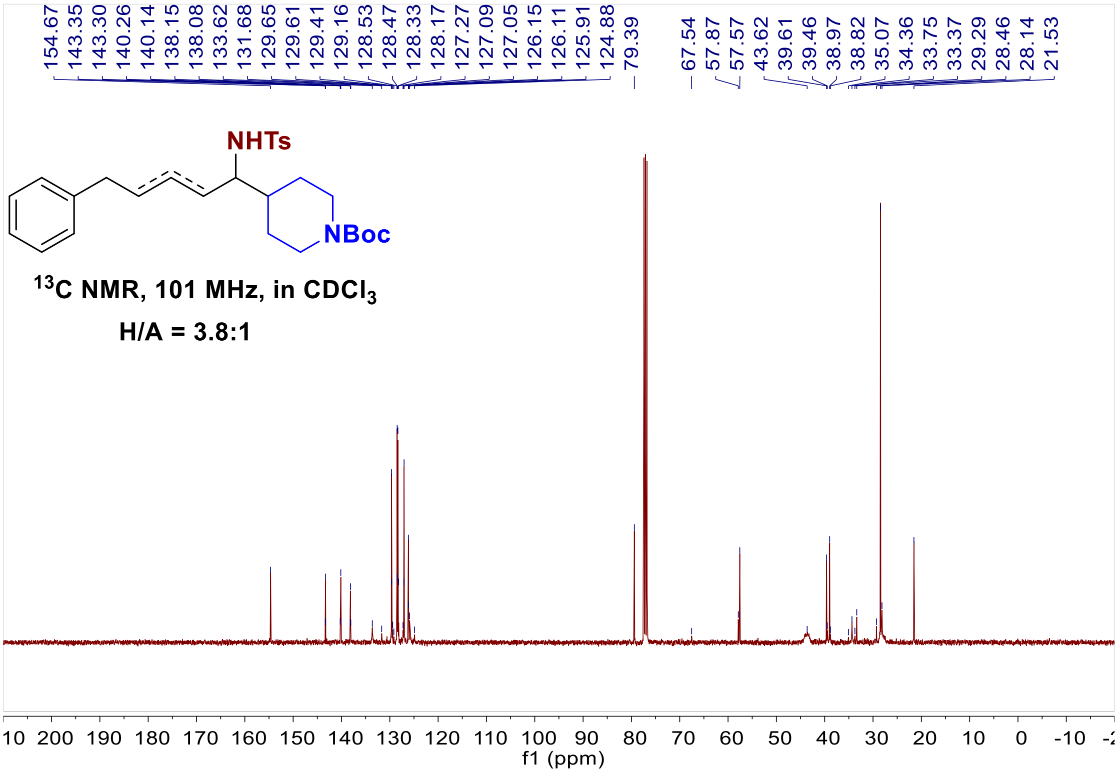

***tert*-butyl-(*E*)-4-(1-((4-methylphenyl)sulfonamido)oct-3-en-1-yl)piperidine-1-carboxylate (mixture A/H= 3:1) (49 and 49′)**

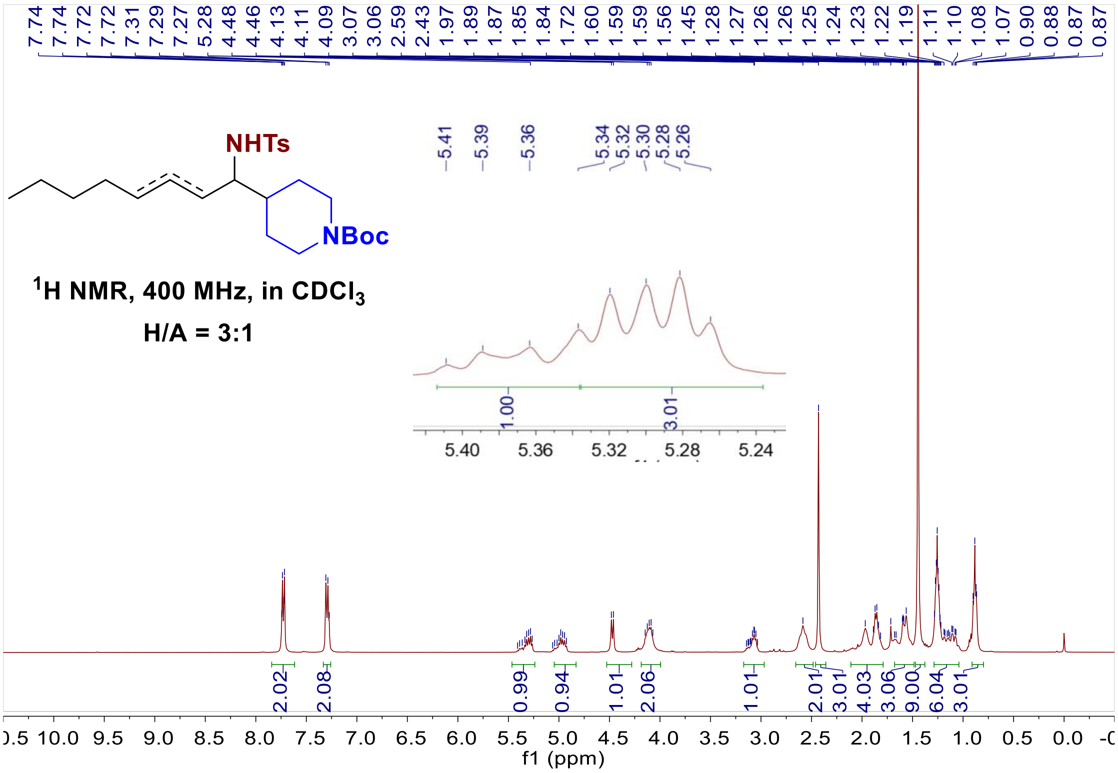

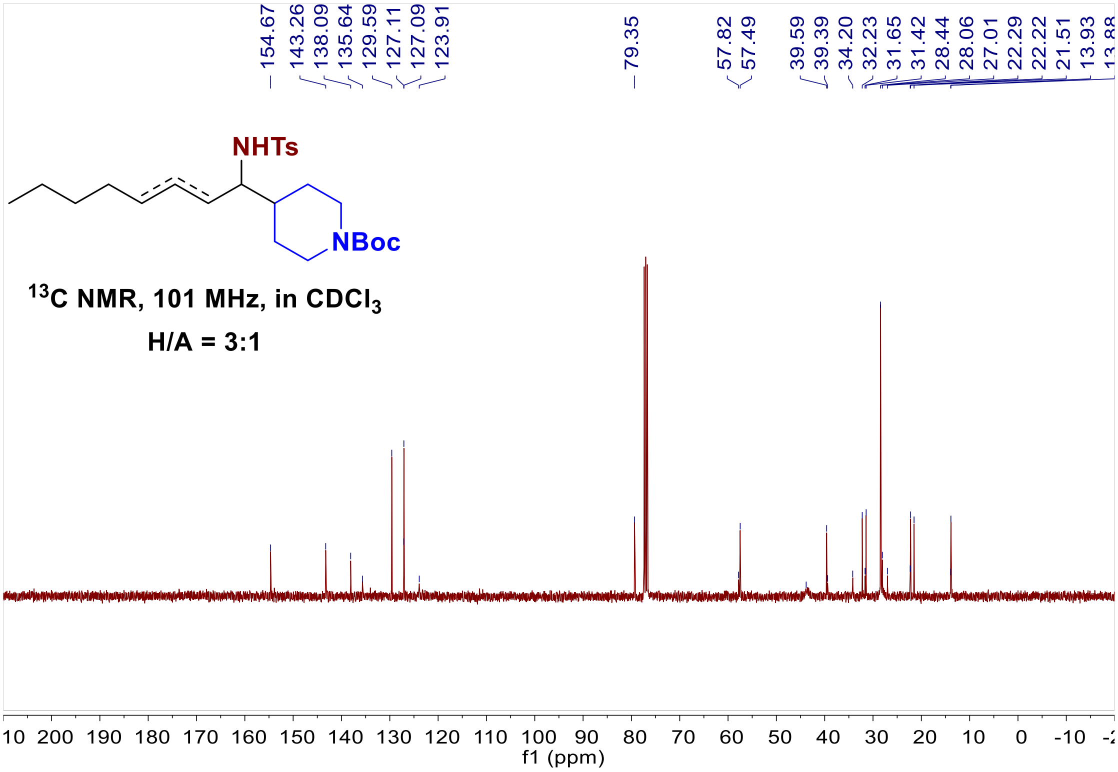

tert-butyl-4-(3-cyclohexylidene-1-((4-methylphenyl)sulfonamido)propyl)piperidine-1-carboxylate (50)

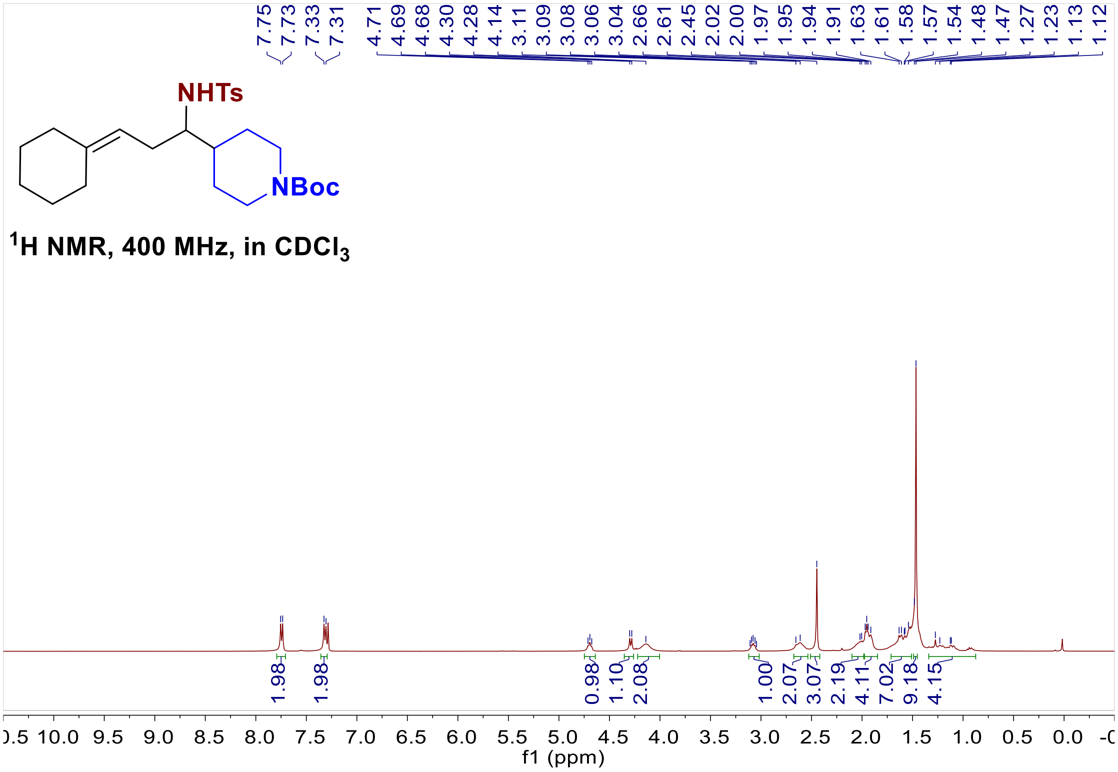

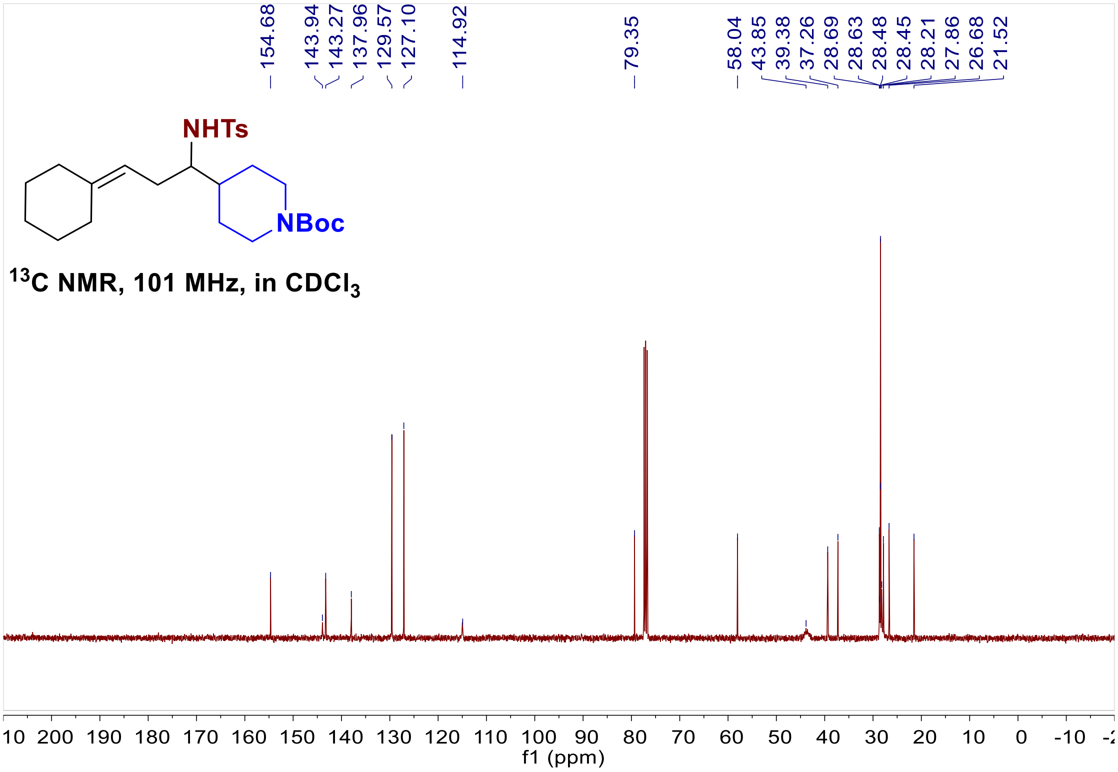

***tert*-butyl-(*E*)-4-(1-((4-methylphenyl)sulfonamido)-4-phenylbut-3-en-1-yl)piperidine-1-carboxylate (51)**

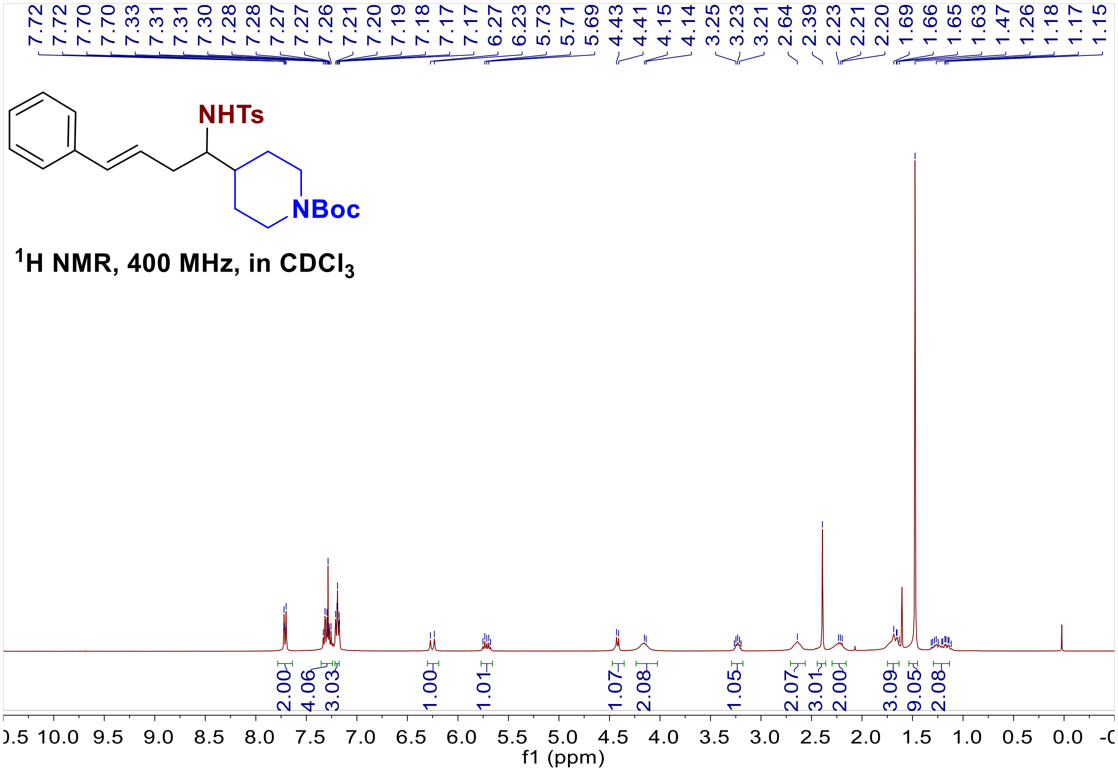

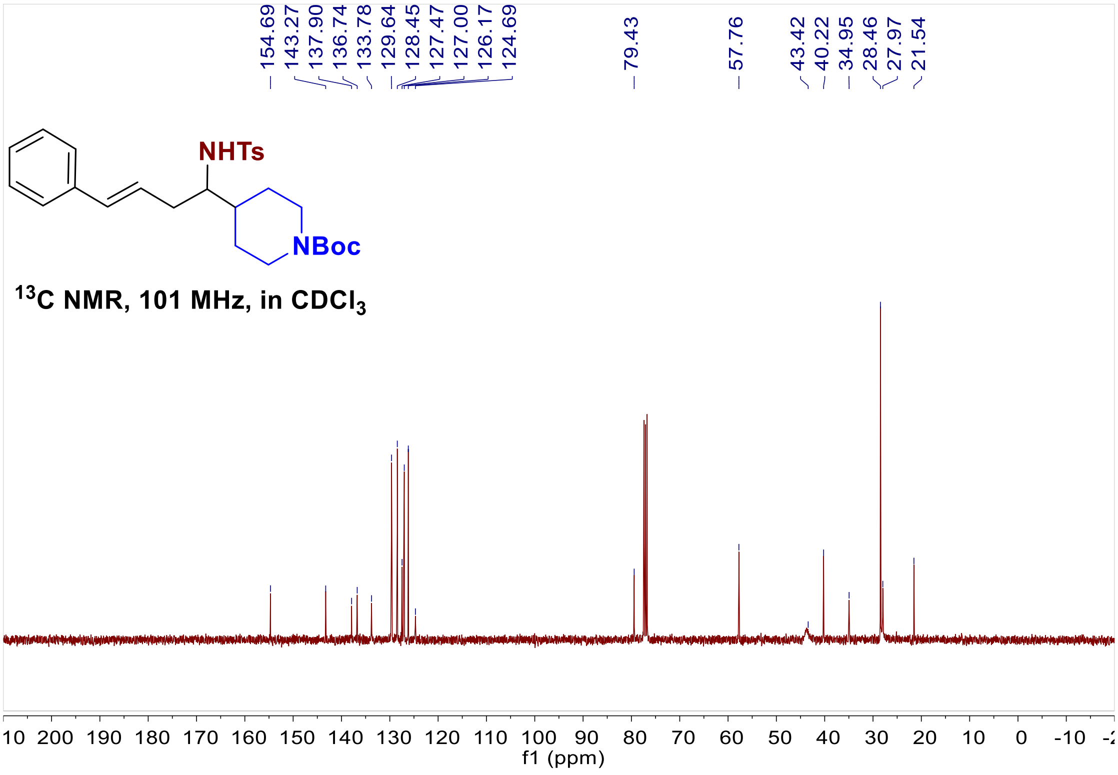

***tert*-butyl-(*E*)-4-(4-(4-methoxyphenyl)-1-((4-methylphenyl)sulfonamido)but-3-en-1-yl)piperidine-1-carboxylate (52)**

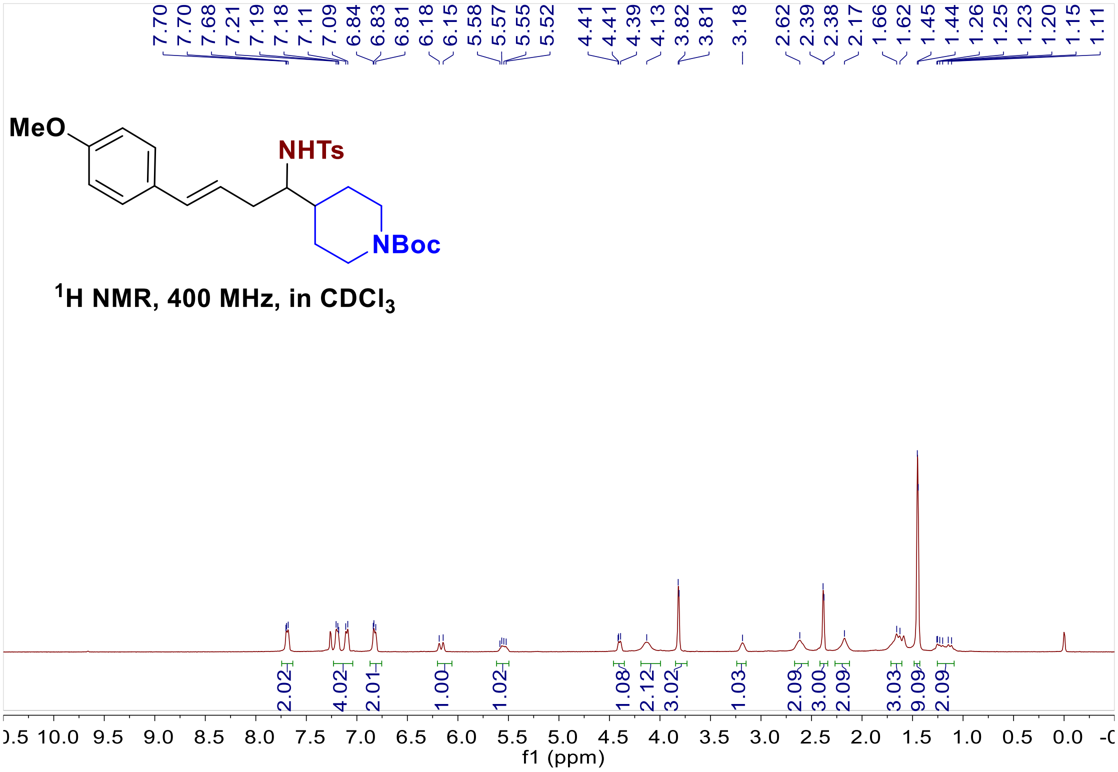

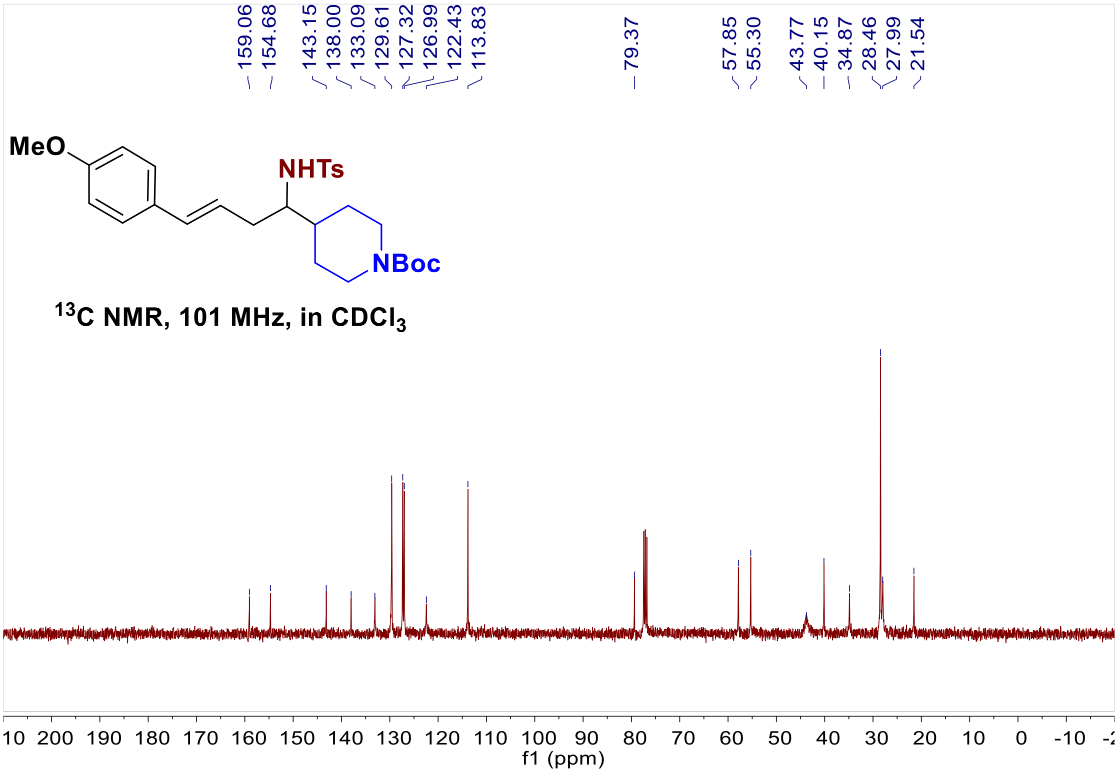

N-cinnamyl-4-(5-(*p*-tolyl)-3-(trifluoromethyl)-1H-pyrazol-1-yl)-benzenesulfonamide (53)

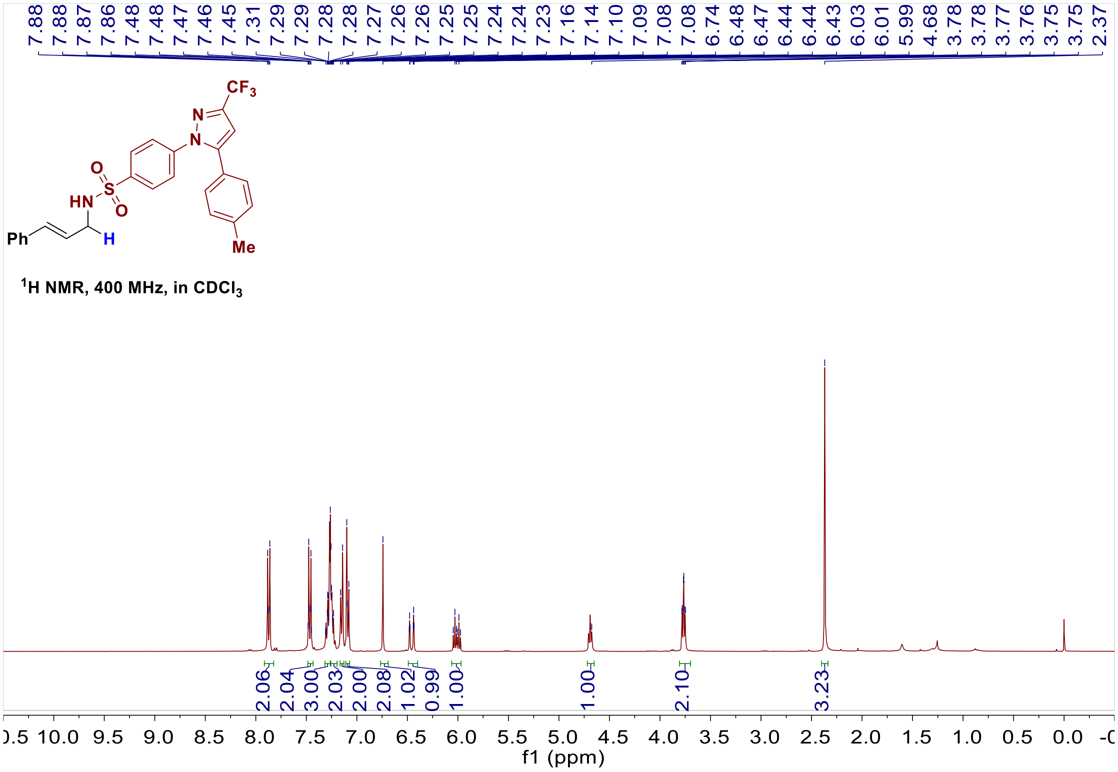

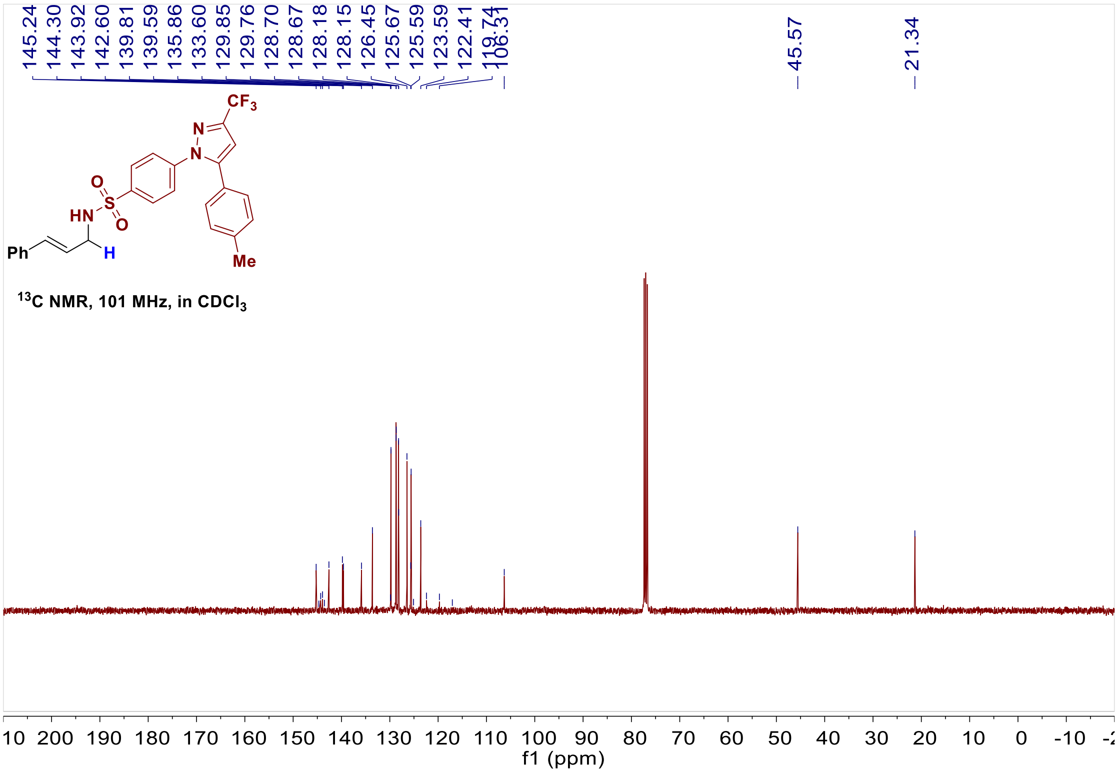

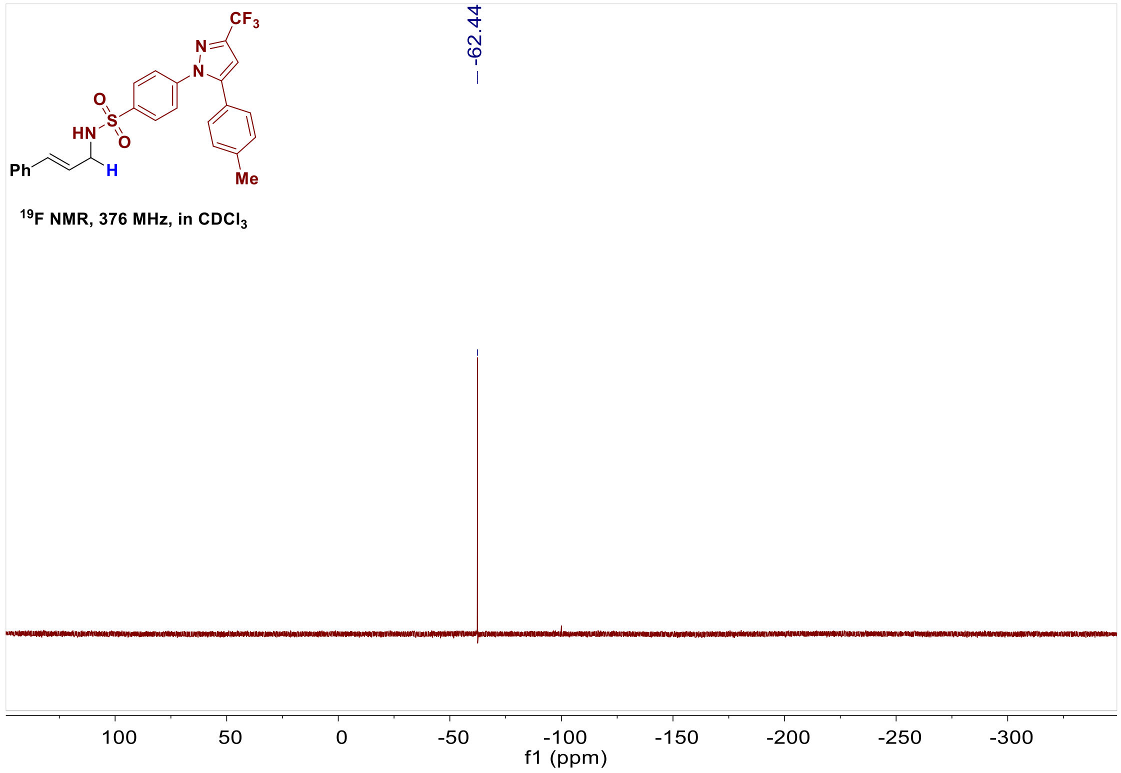

CF3
N
N
O
S
HN
O
Ph
H
Me
19F NMR, 376 MHz, in CDCl3
-62.44
100
50
0
-50
-100
-150
-200
-250
-300
f1 (ppm)

(*E*)-N-(1-cyclohexyl-3-phenylallyl)-4-(5-(*p*-tolyl)-3-(trifluoromethyl)-1H-pyrazol-1-yl)-benzenesulfonamide (54)

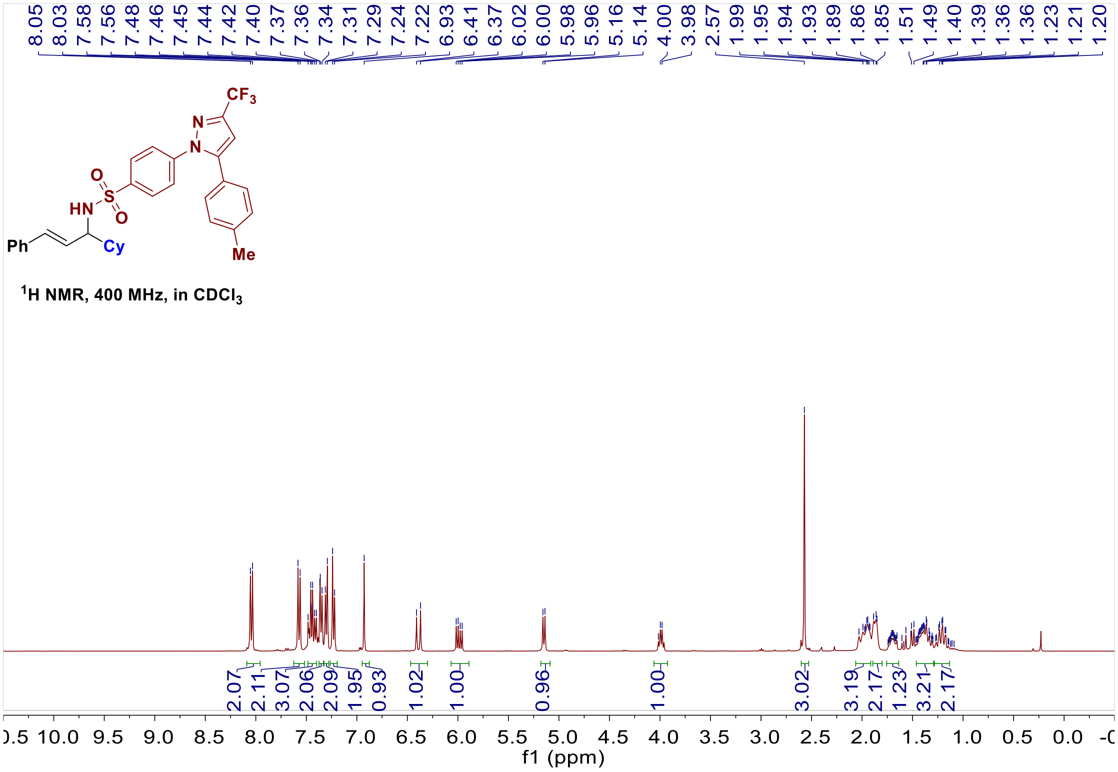

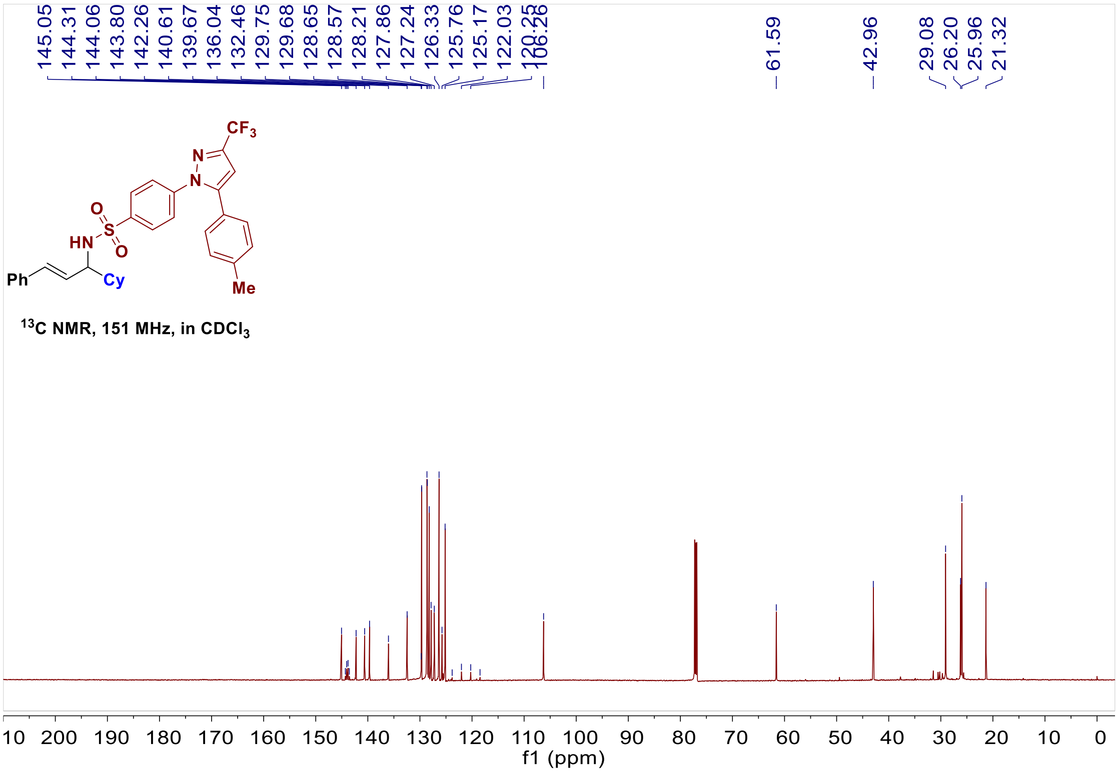

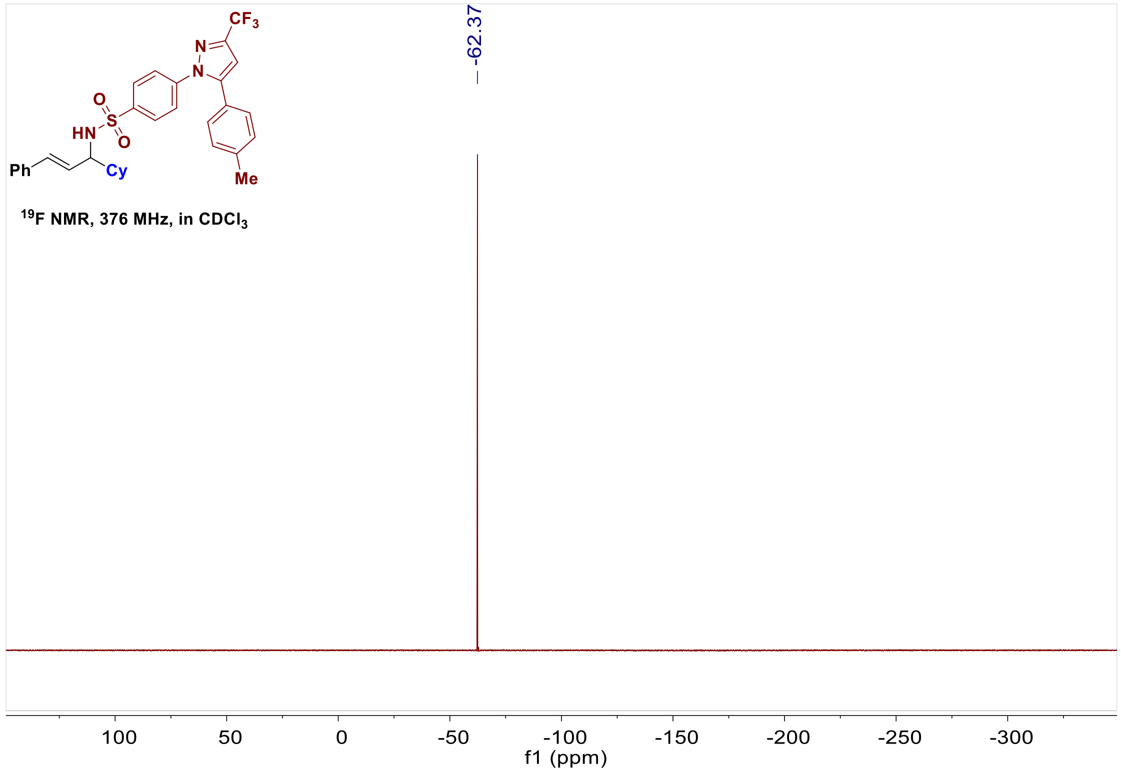

-62.37
CF3
N
N
O
S
HN
O
Ph
Cy
Me
19F NMR, 376 MHz, in CDCl3
100
50
0
-50
-100
-150
-200
-250
-300
f1 (ppm)

(*S*)-5-(N-cinnamylsulfamoyl)-N-((1-ethylpyrrolidin-2-yl)methyl)-2-methoxybenzamide (55)

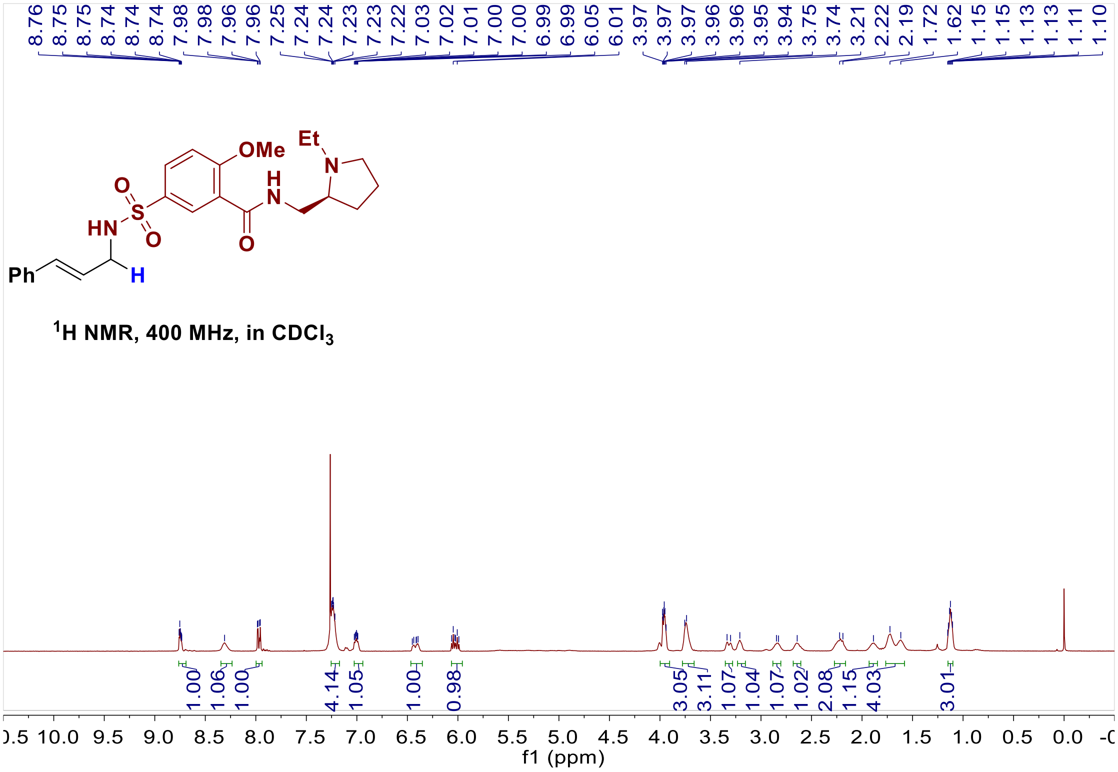

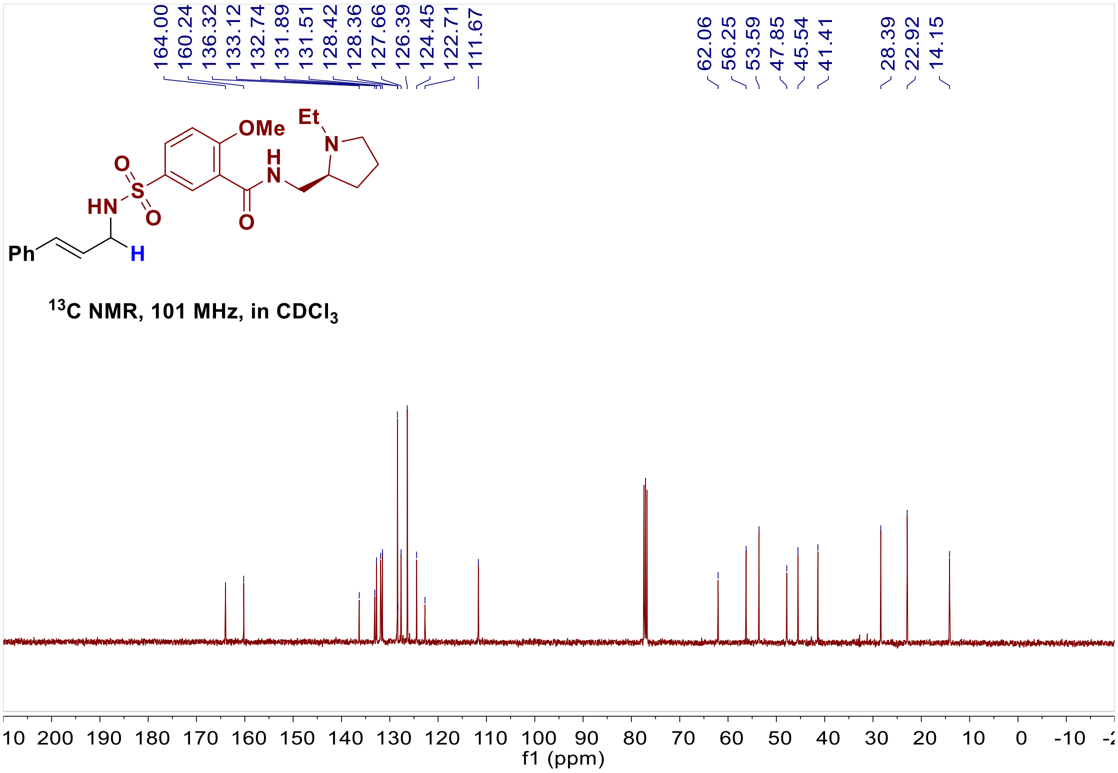

5-(N-((*E*)-1-cyclohexyl-3-phenylallyl)sulfamoyl)-N-(((*S*)-1-ethylpyrrolidin-2-yl)methyl)-2-methoxybenzamide (56)

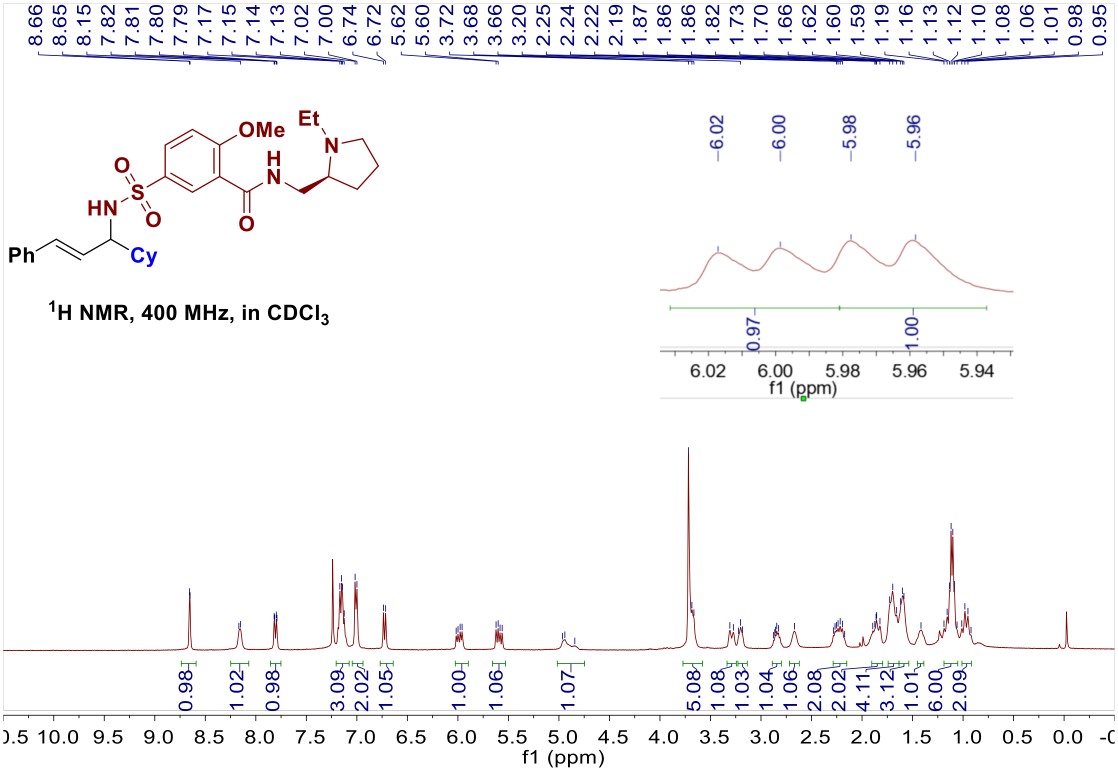

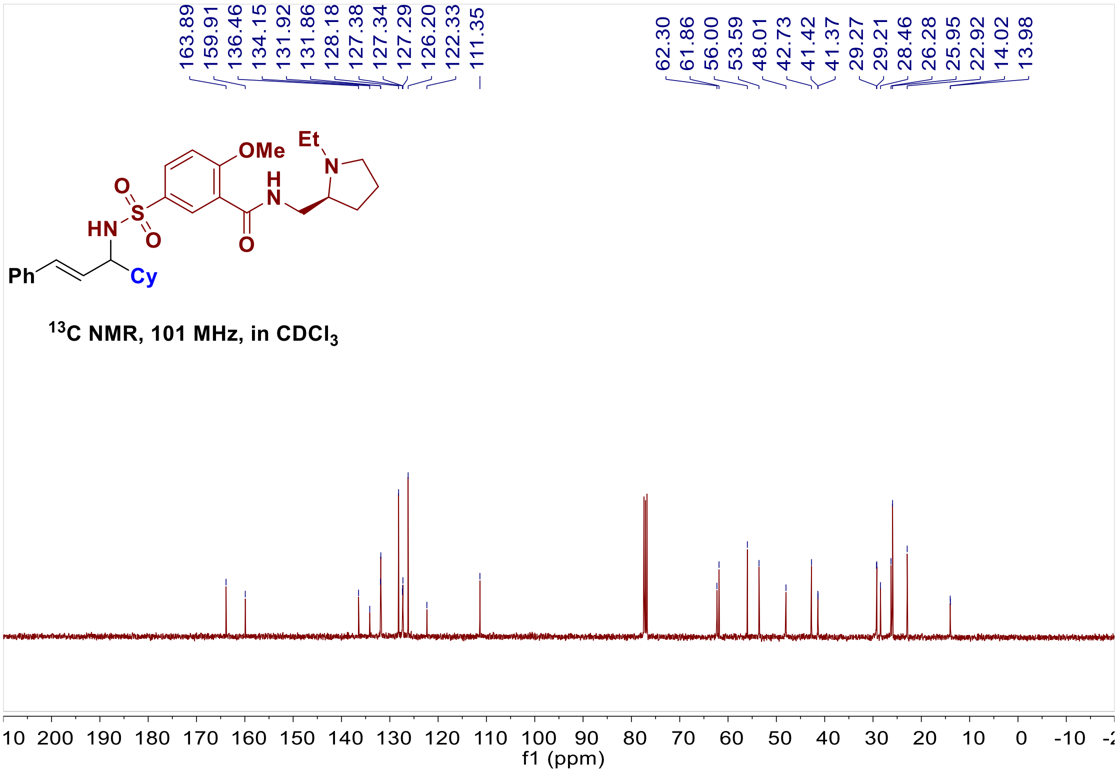

N-cinnamyl-1-((1*R*, 4*S*)-7,7-dimethyl-2-oxobicyclo[2.2.1]heptan-1-yl)methanesulfonamide (57)

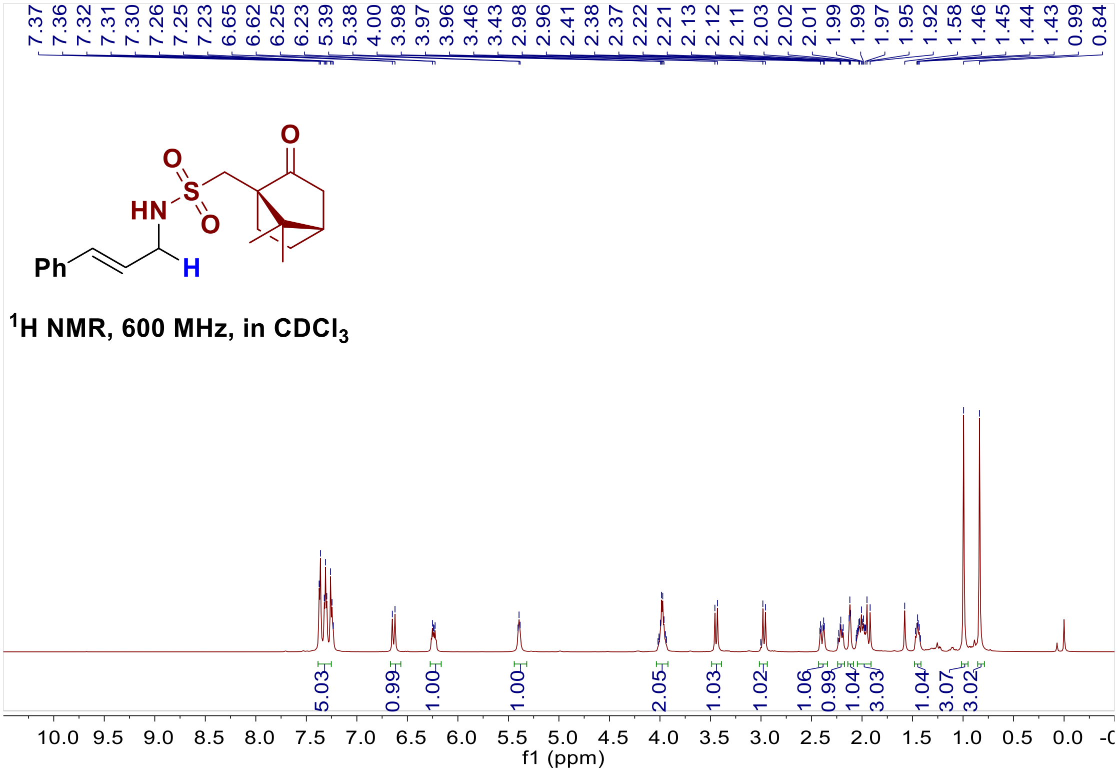

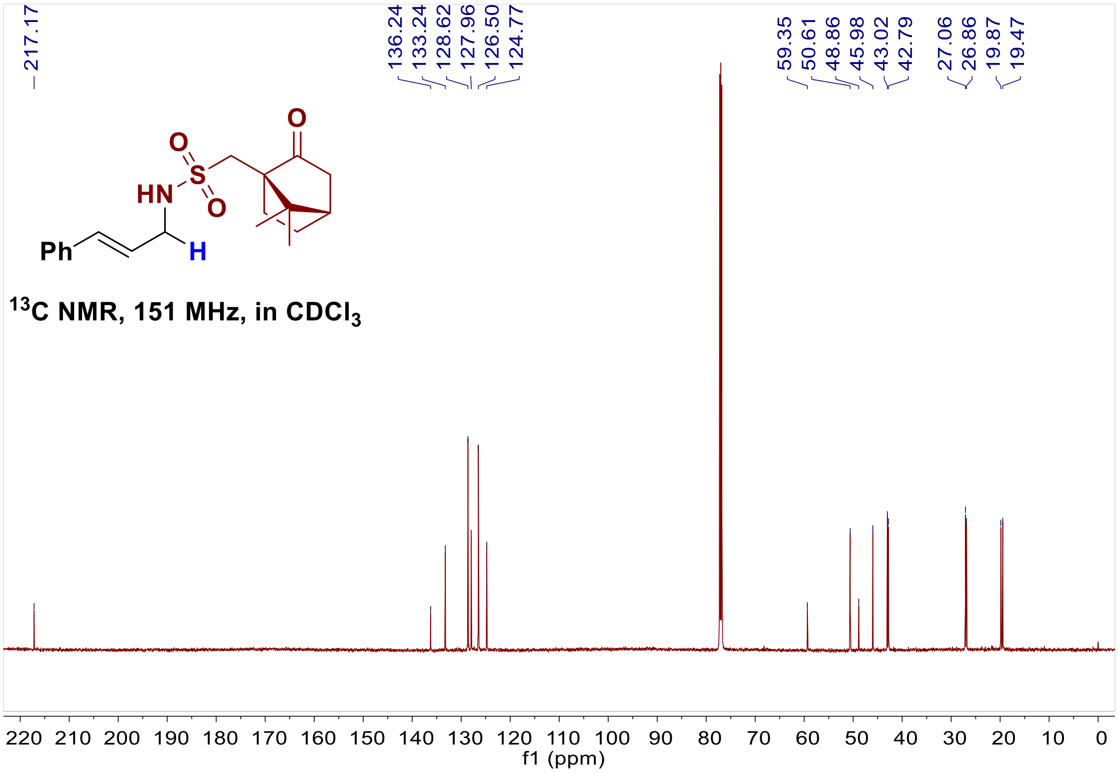

N-((*E*)-1-cyclohexyl-3-phenylallyl)-1-((1*R*, 4*S*)-7,7-dimethyl-2-oxobicyclo[2.2.1]heptan-1-yl)methanesulfonamide (58)

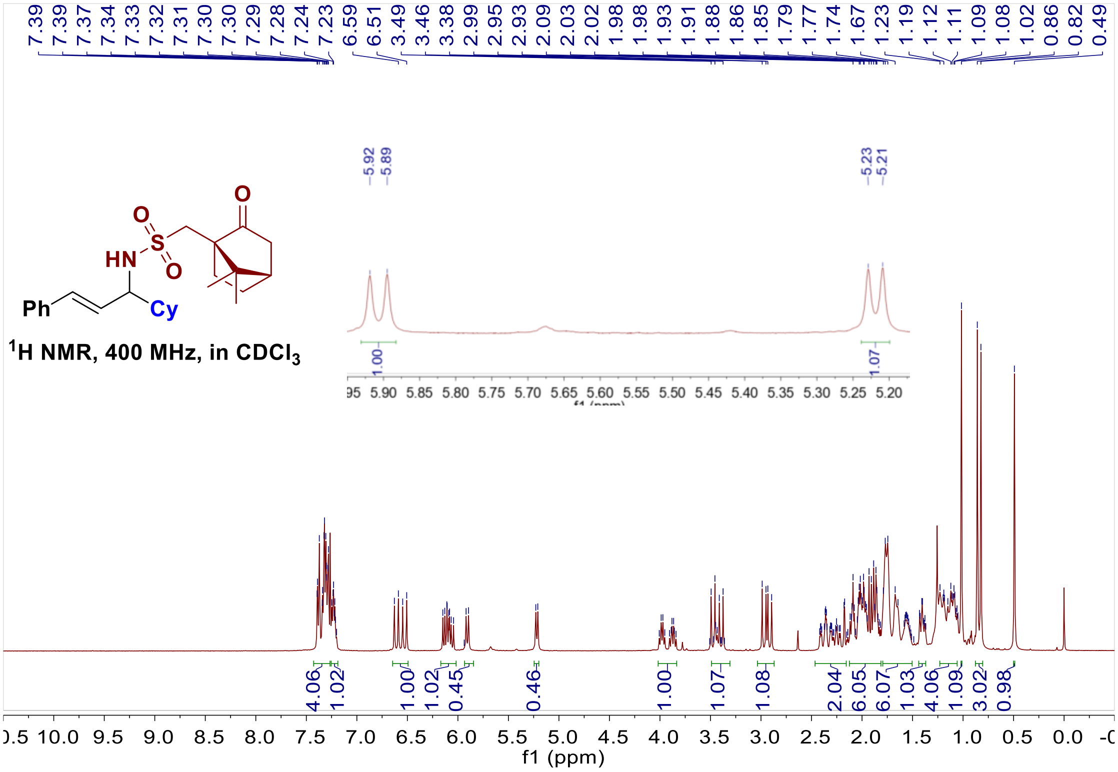

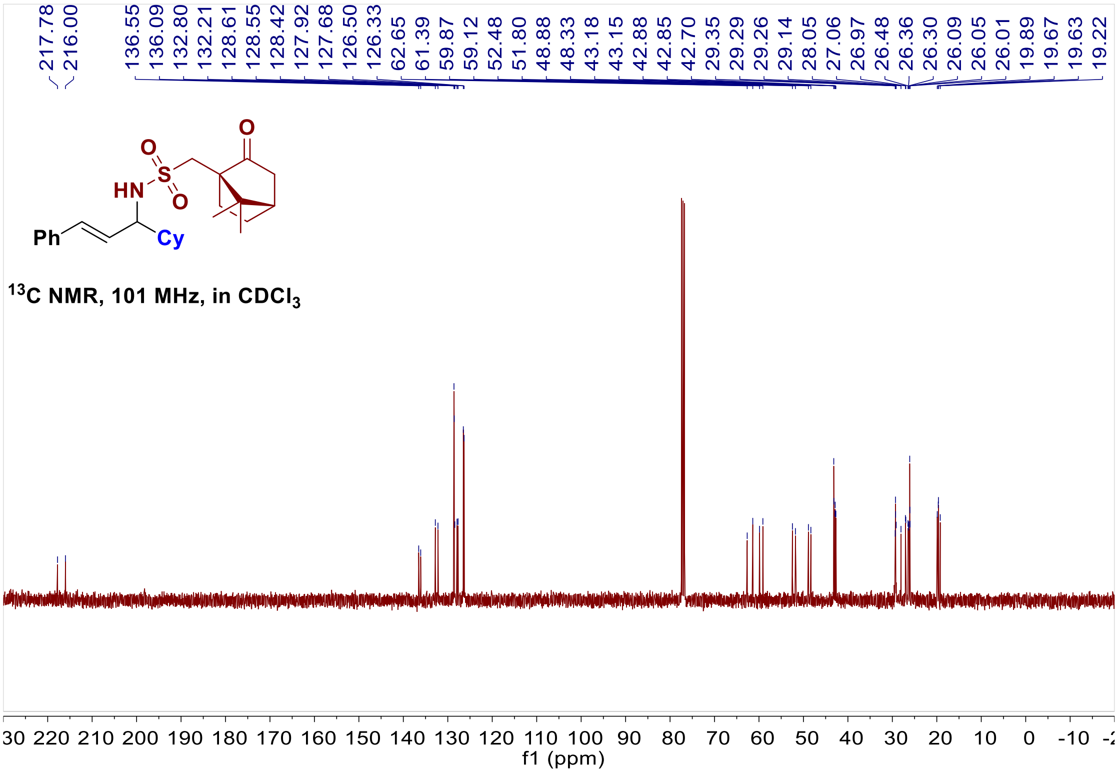

(*E*)-N-(7-(2,5-dimethylphenoxy)-4,4-dimethyl-1-phenylhept-1-en-3-yl)-4-methylbenzenesulfonamide (59)

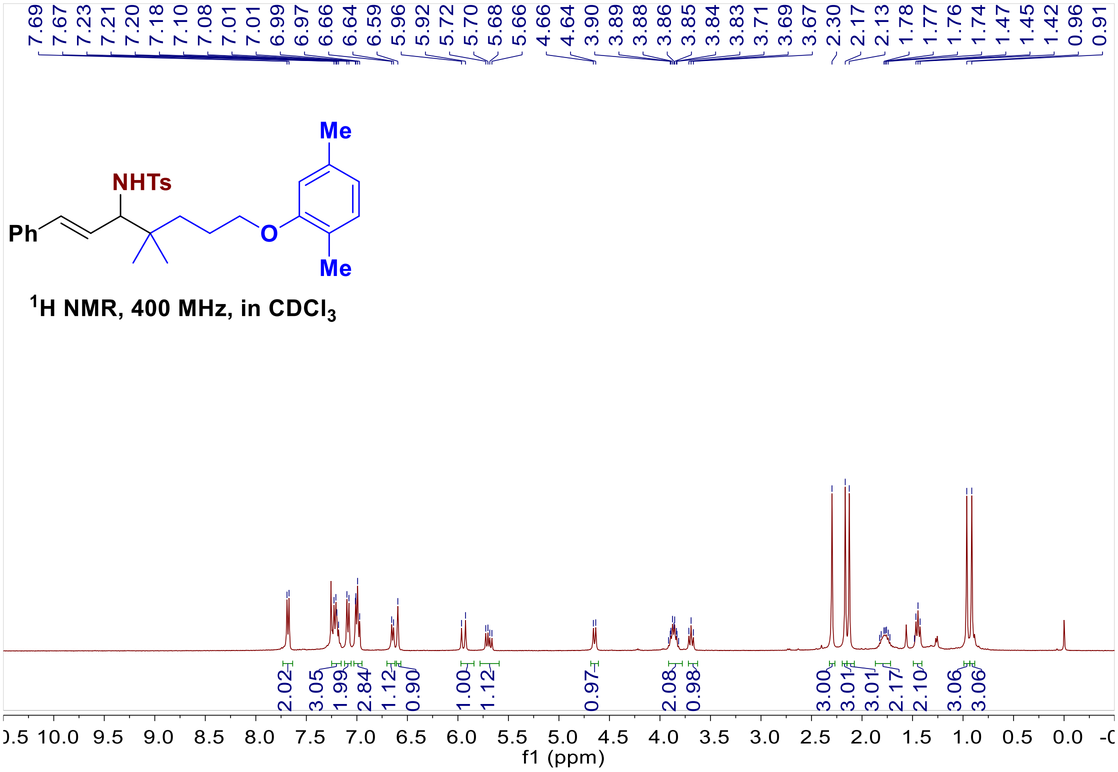

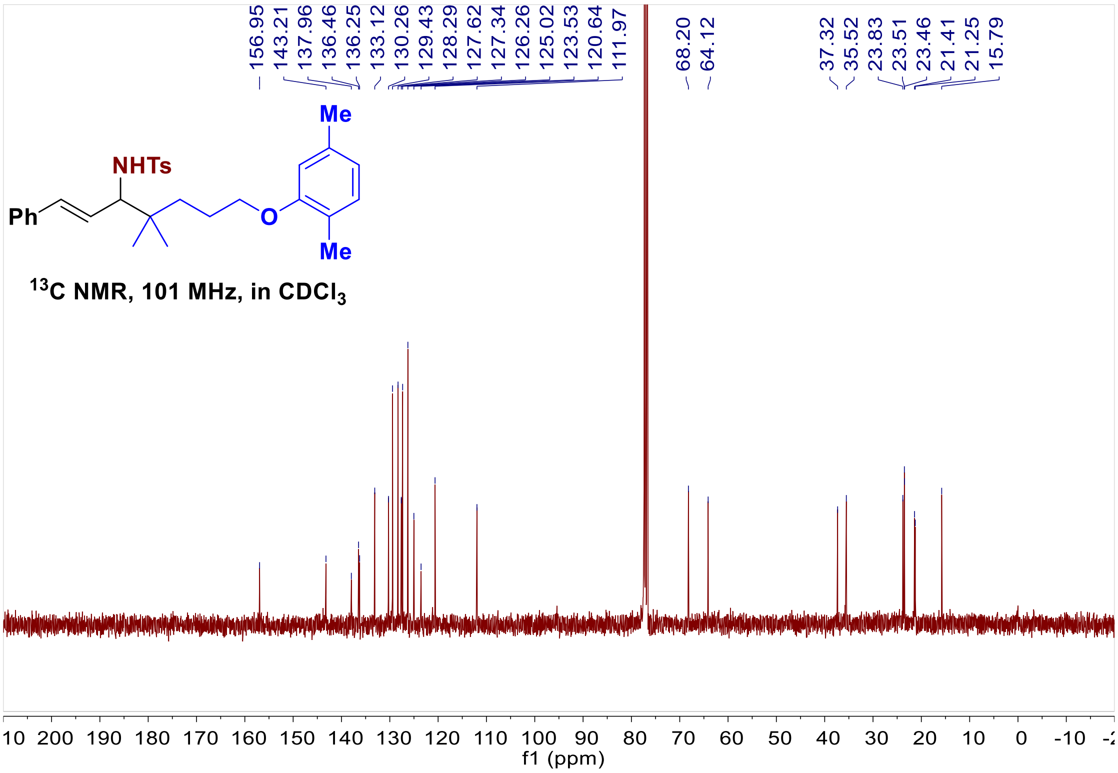

(*E*)-N-(5,9-dimethyl-1-phenyldeca-1,8-dien-3-yl)-4-methylbenzenesulfonamide (60)

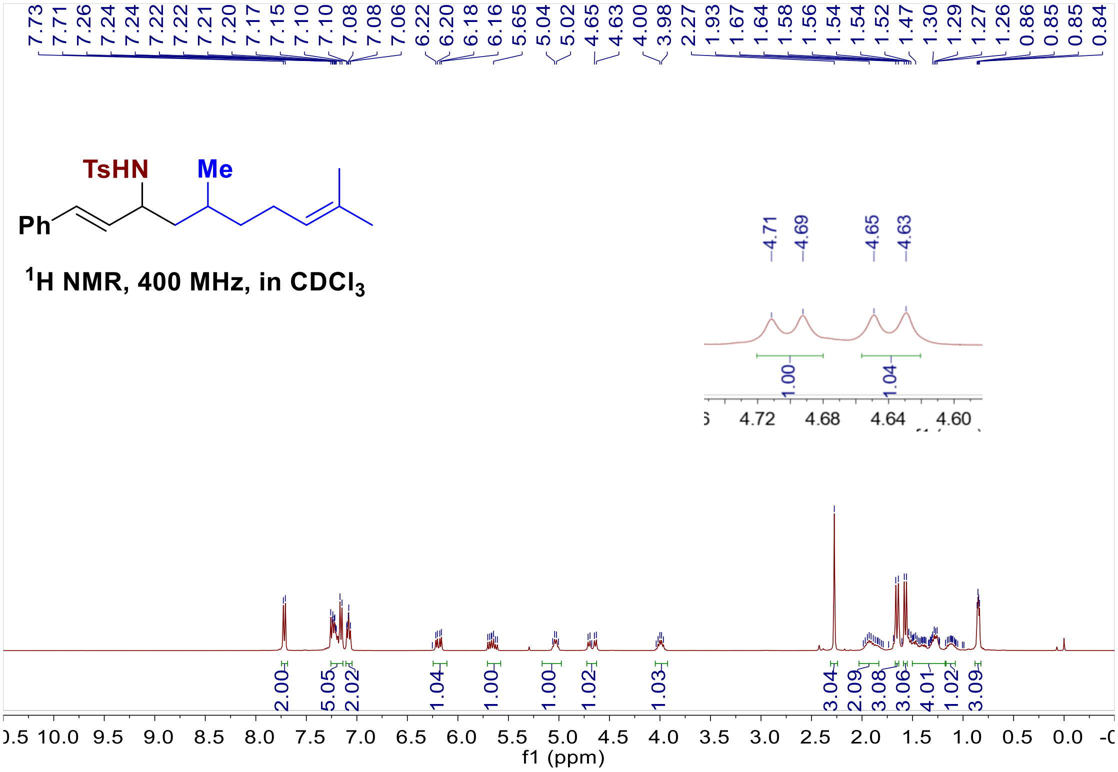

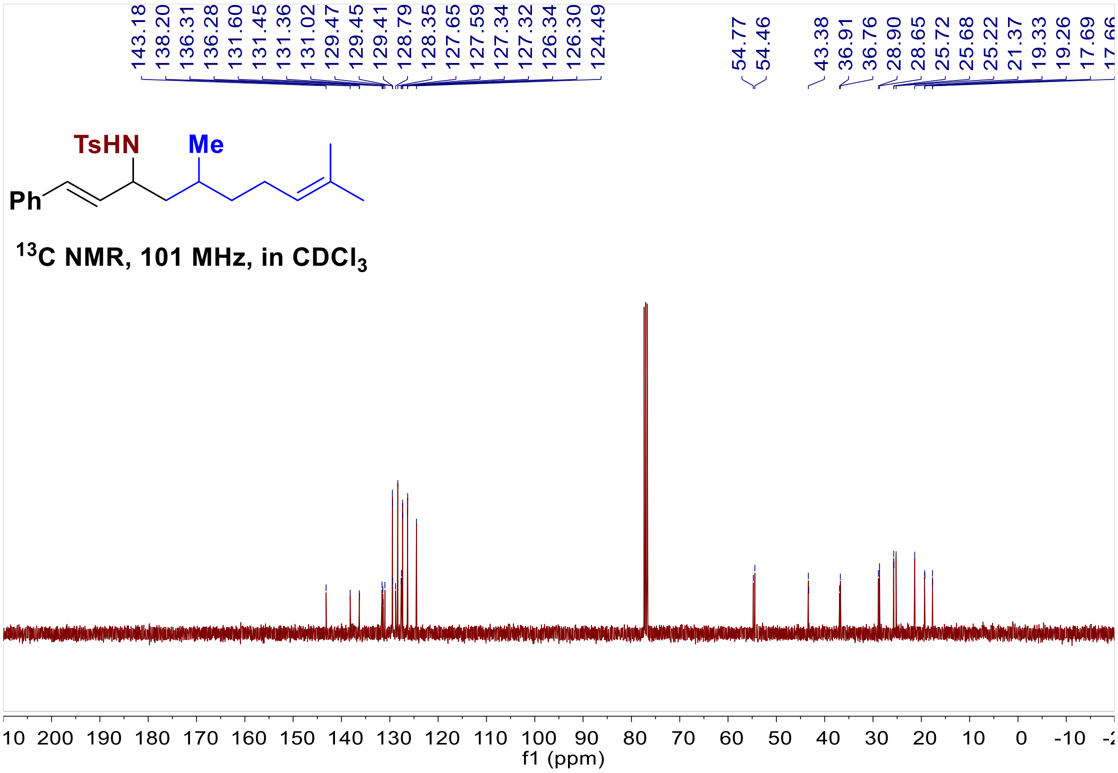

(*E*)-N-(1-(4-(4-hydroxy-4-methylpentyl)cyclohex-3-en-1-yl)-3-phenylallyl)-4-methylbenzenesulfonamide (61)

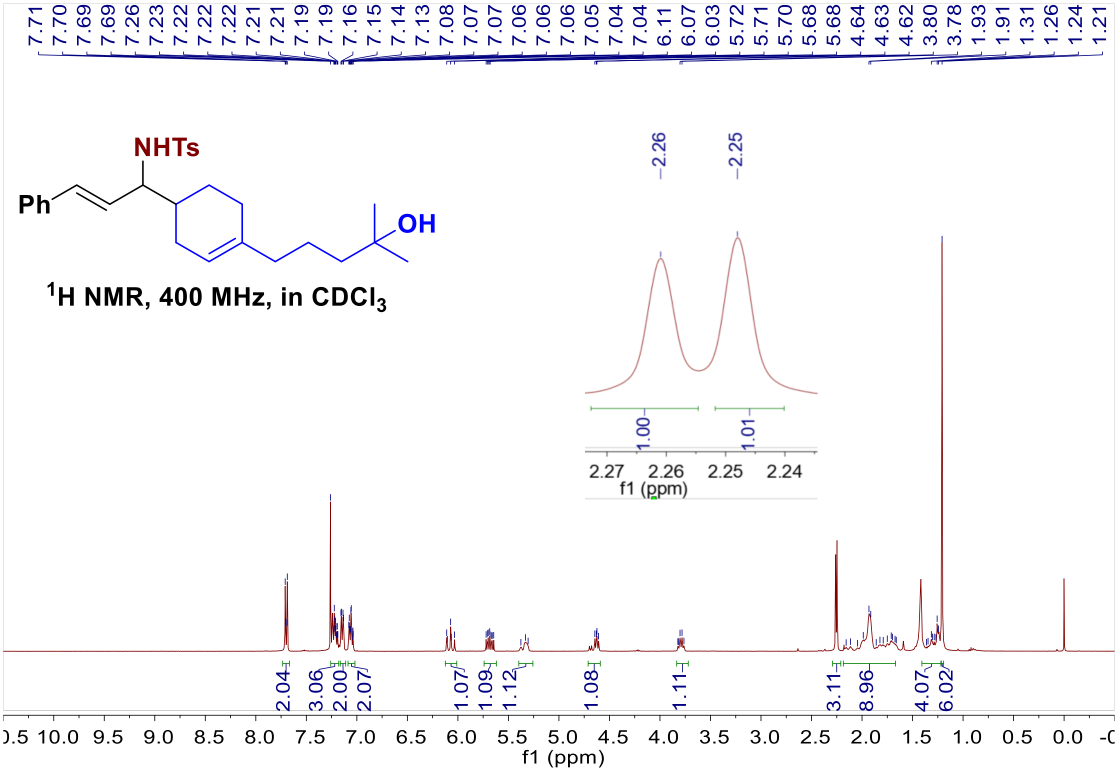

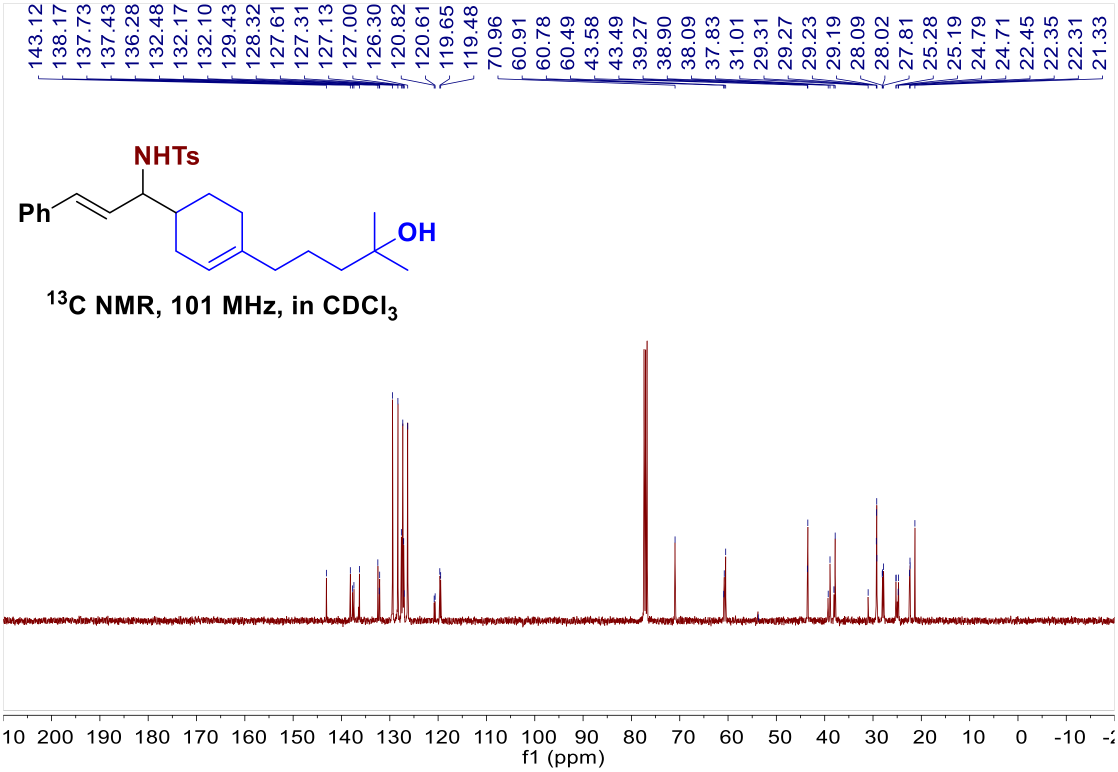

N-((6*S*, *E*)-6-((3*S*, 5*R*, 7*R*, 8*S*, 9*R*, 10*R*, 13*S*, 14*R*, 17*S*)-3,7-dihydroxy-10,13-dimethylhexadecahydro-1H-cyclopenta[a]phenanthren-17-yl)-1-phenylhept-1-en-3-yl)-4-methylbenzenesulfonamide (62)

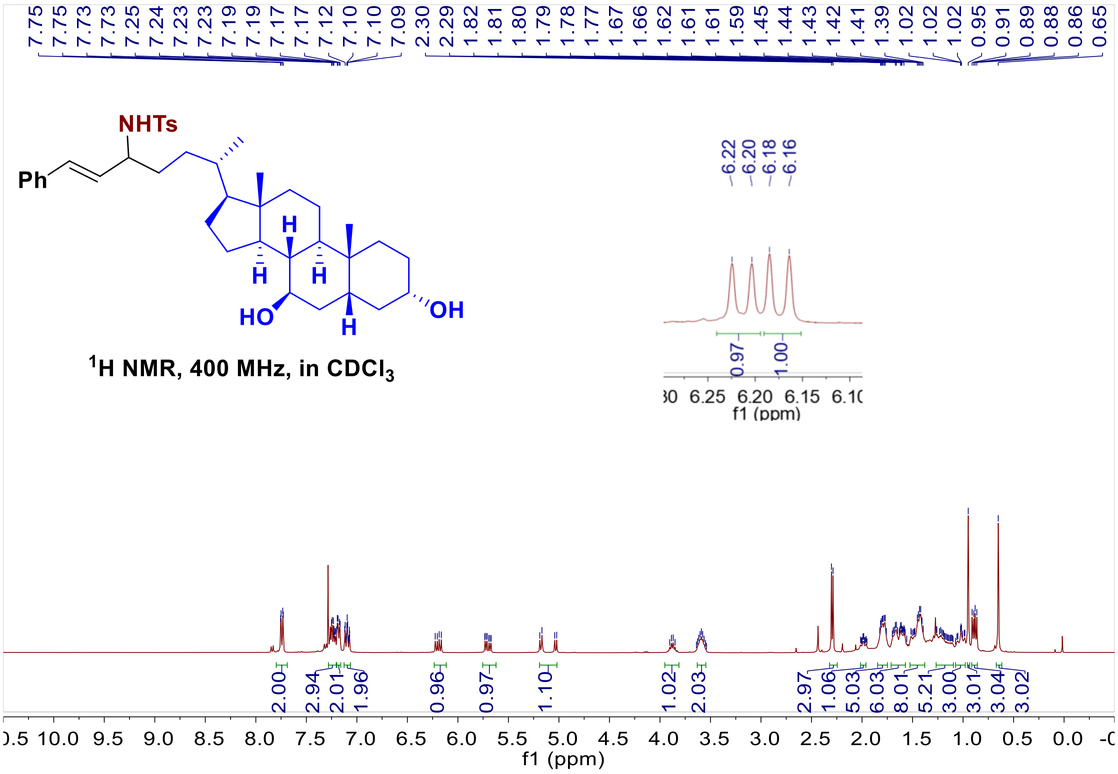

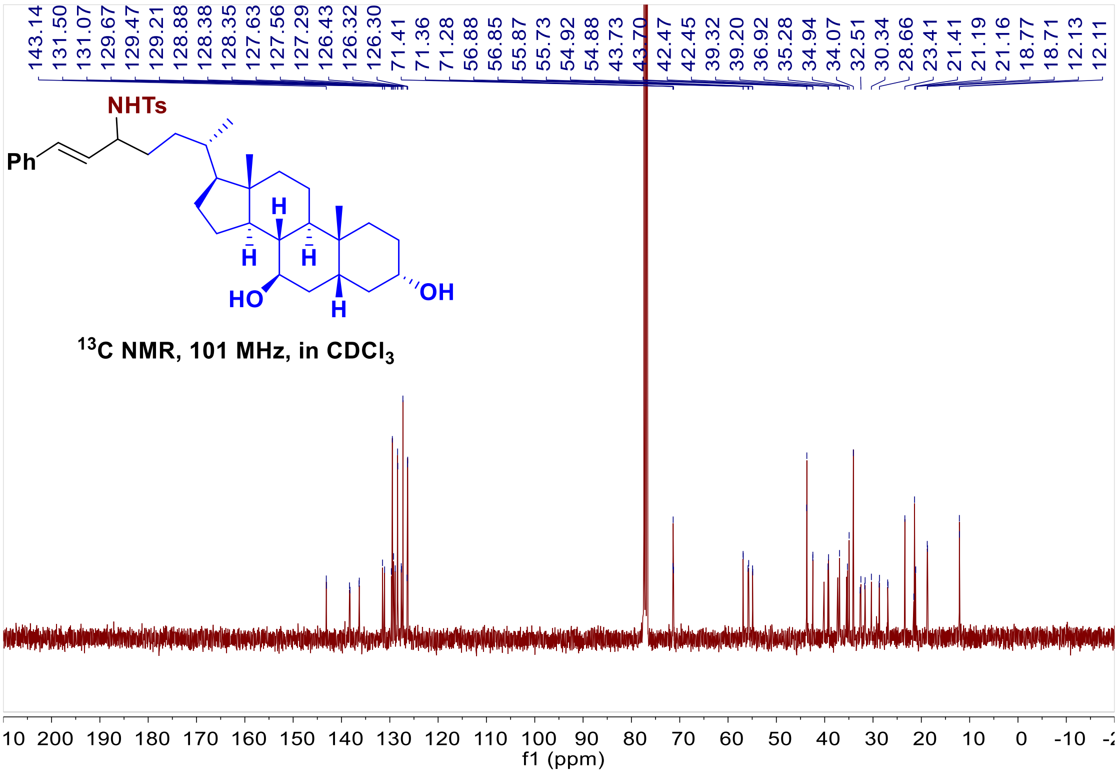

tert-butyl-(_E_)-4-(3-(11-(1-(ethoxycarbonyl)piperidin-4-ylidene)-6,11-dihydro-5H-benzo[5,6]cyclohepta[1,2-b]pyridin-8-yl)-1-((4-methylphenyl)sulfonamido)allyl)piperidine-1-carboxylate (63)

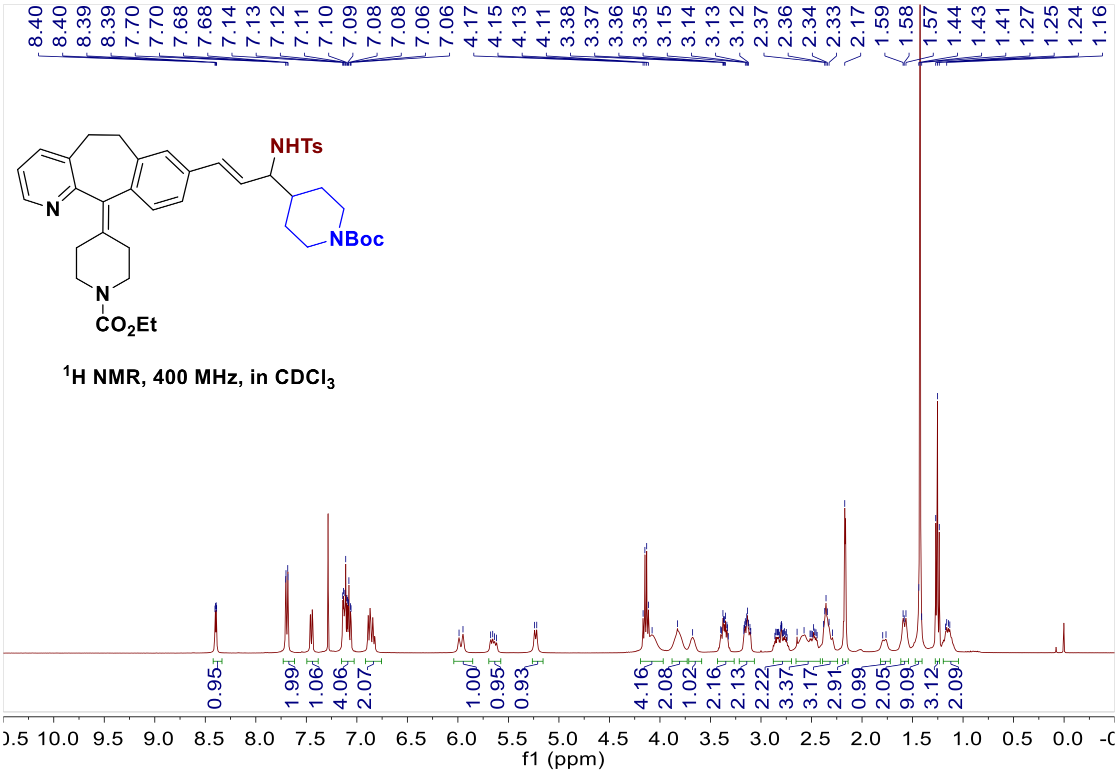

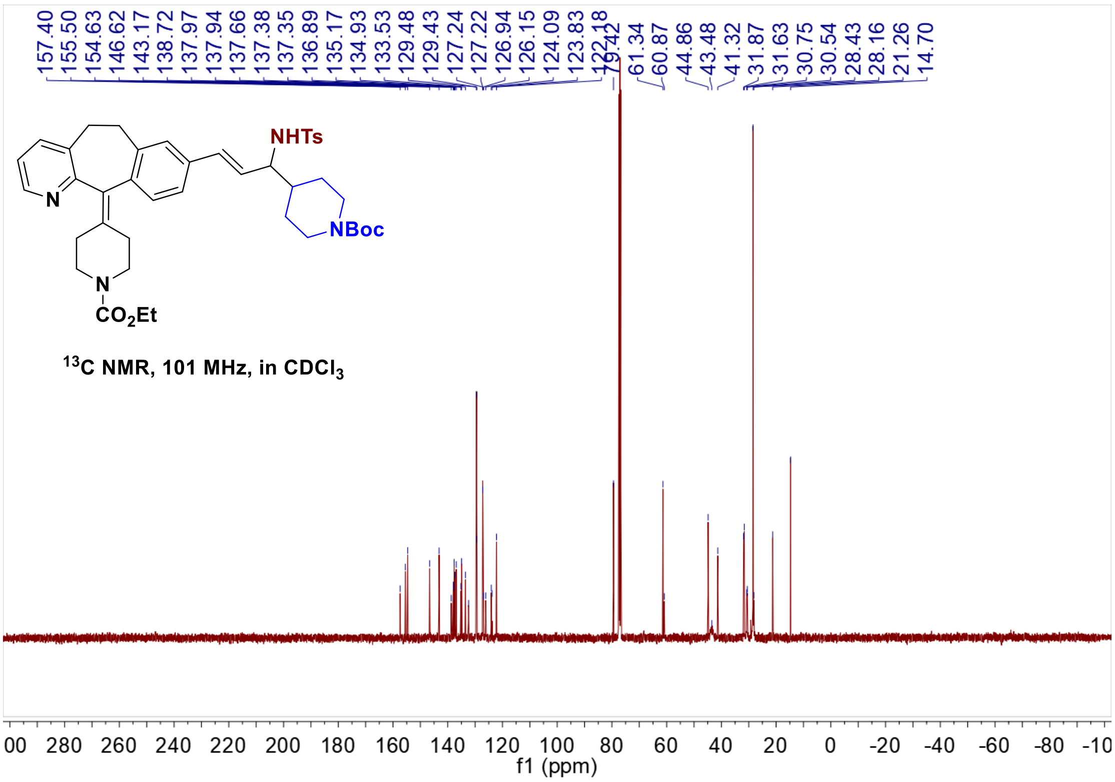

tert-butyl-4-((E)-3-(4-((((1R, 2S, 5R)-2-isopropyl-5-methylcyclohexyl)oxy)methyl)phenyl)-1-((4-methylphenyl)sulfonamido)allyl)piperidine-1-carboxylate (64)

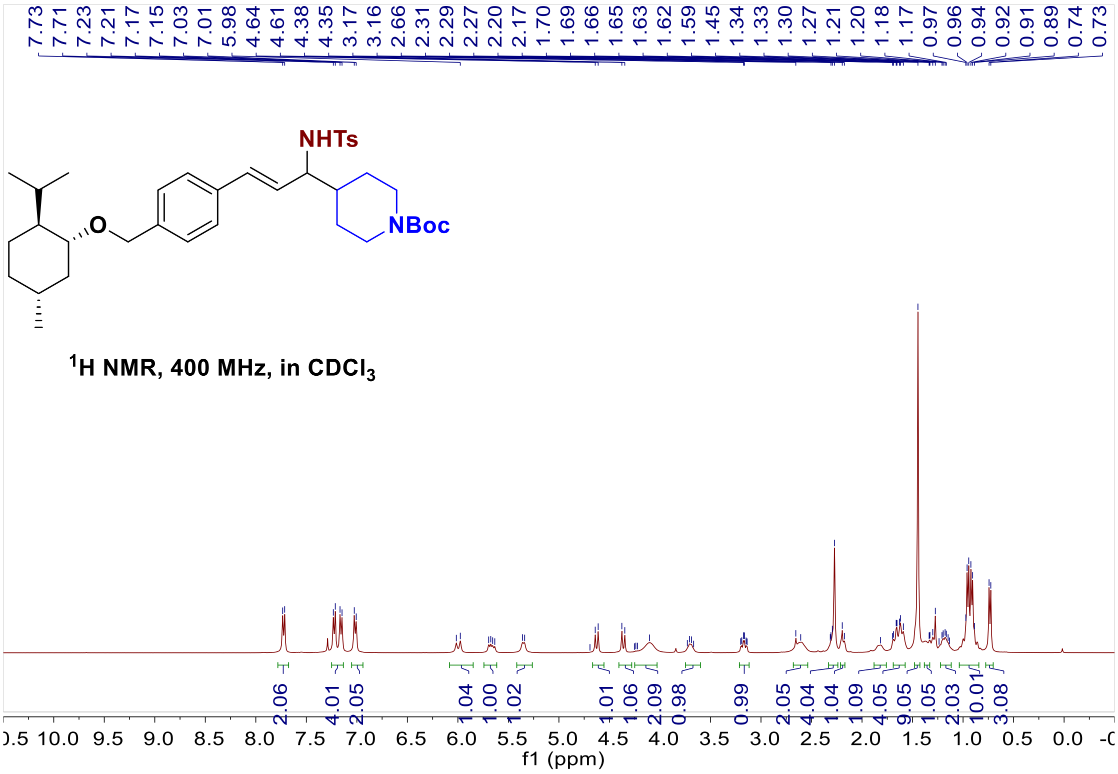

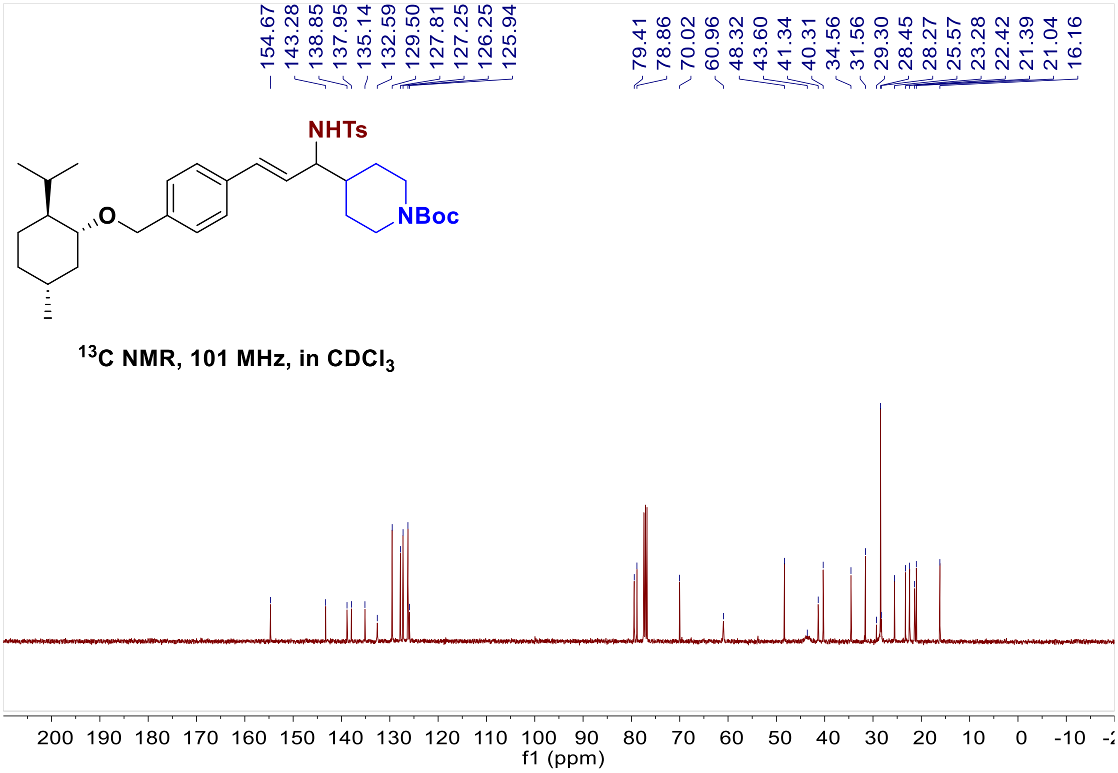

(*E*)-N-(3-(3,5-dichlorophenyl)allyl)methanesulfonamide (65)

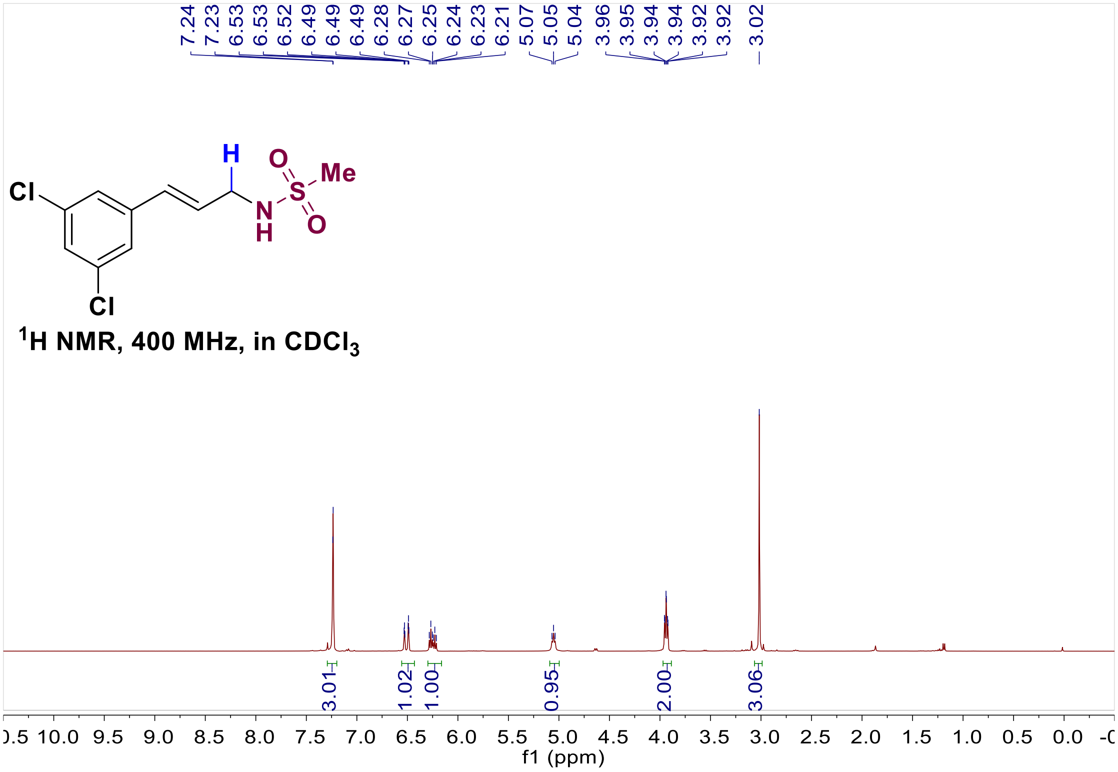

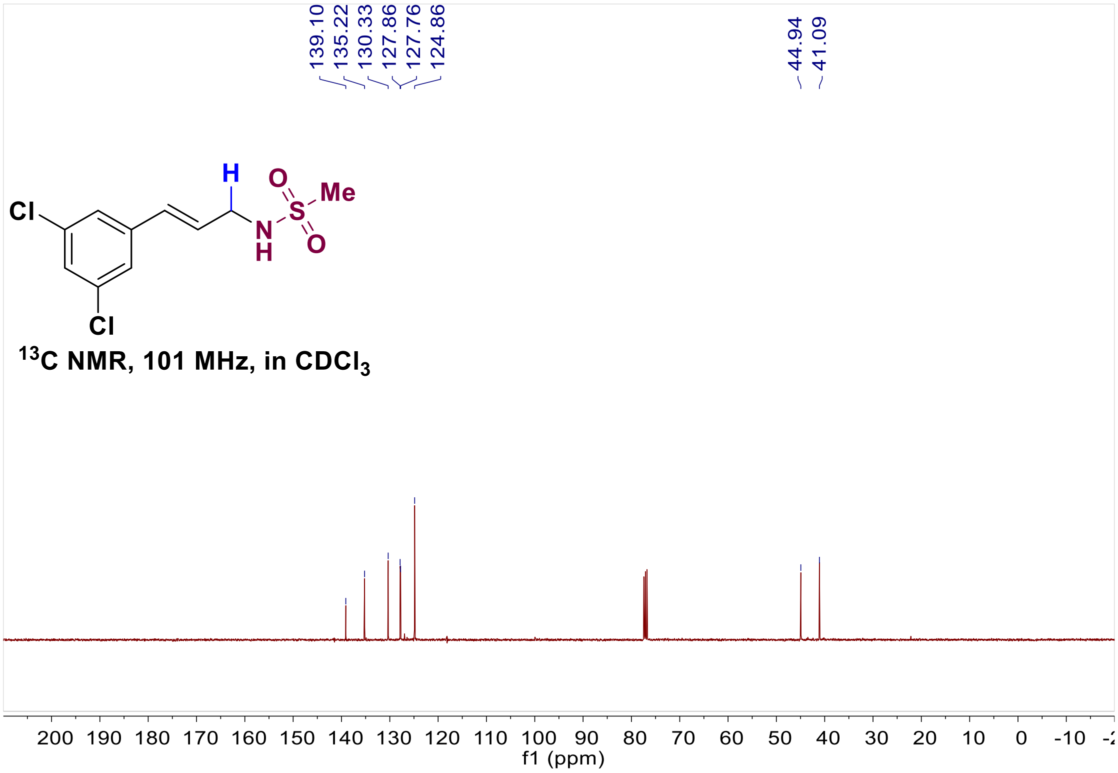

Methyl-(*E*)-2-(4-((N-(3-(3,5-dichlorophenyl)allyl)methylsulfonamido)methyl)phenyl)acetate (66)

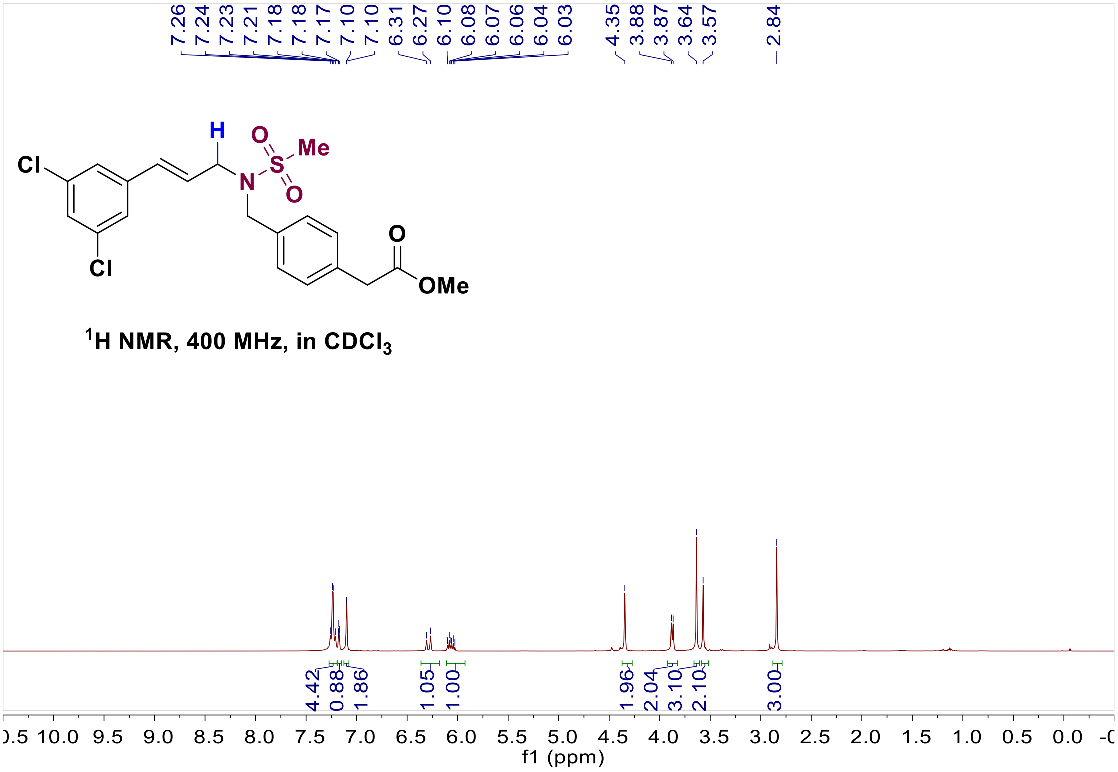

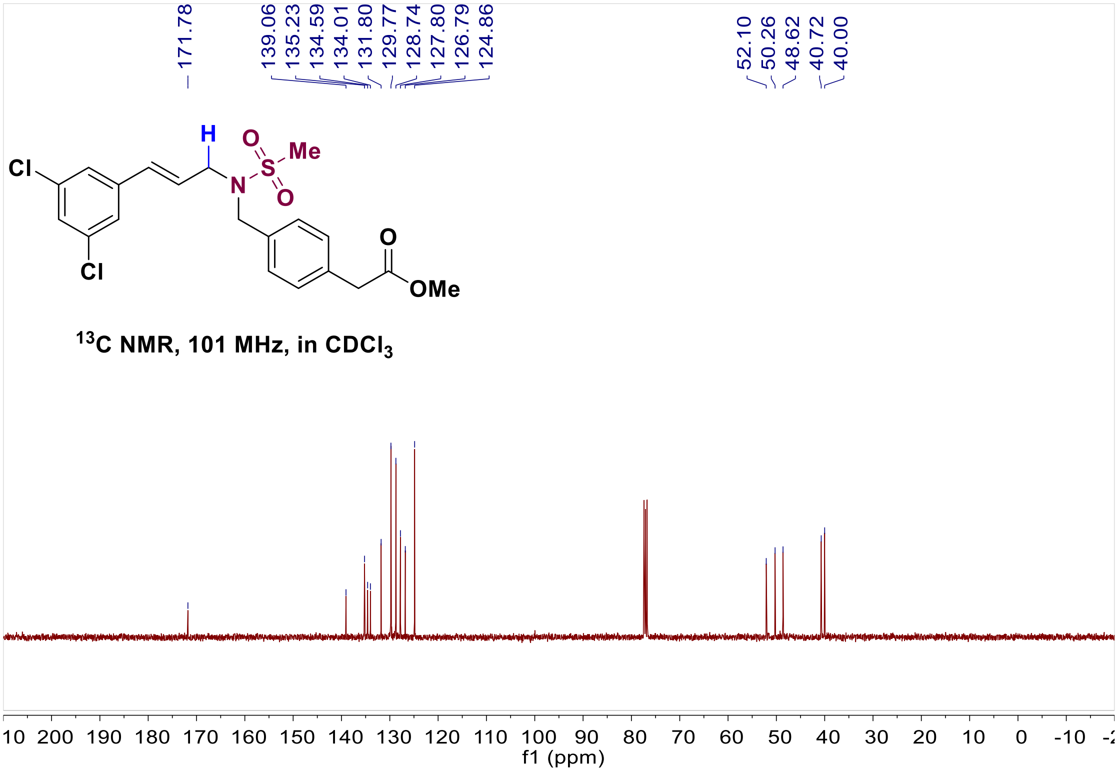

4-methyl-N-(phenyl(3-phenyloxiran-2-yl)methyl)benzenesulfonamide (69)

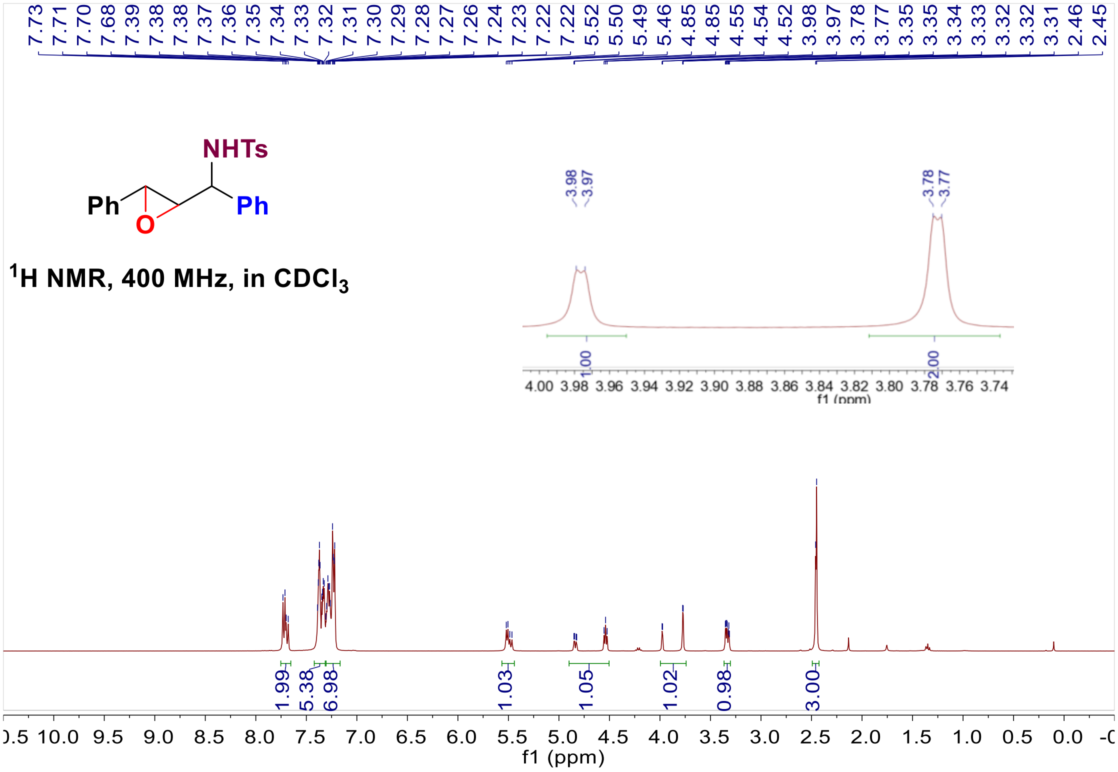

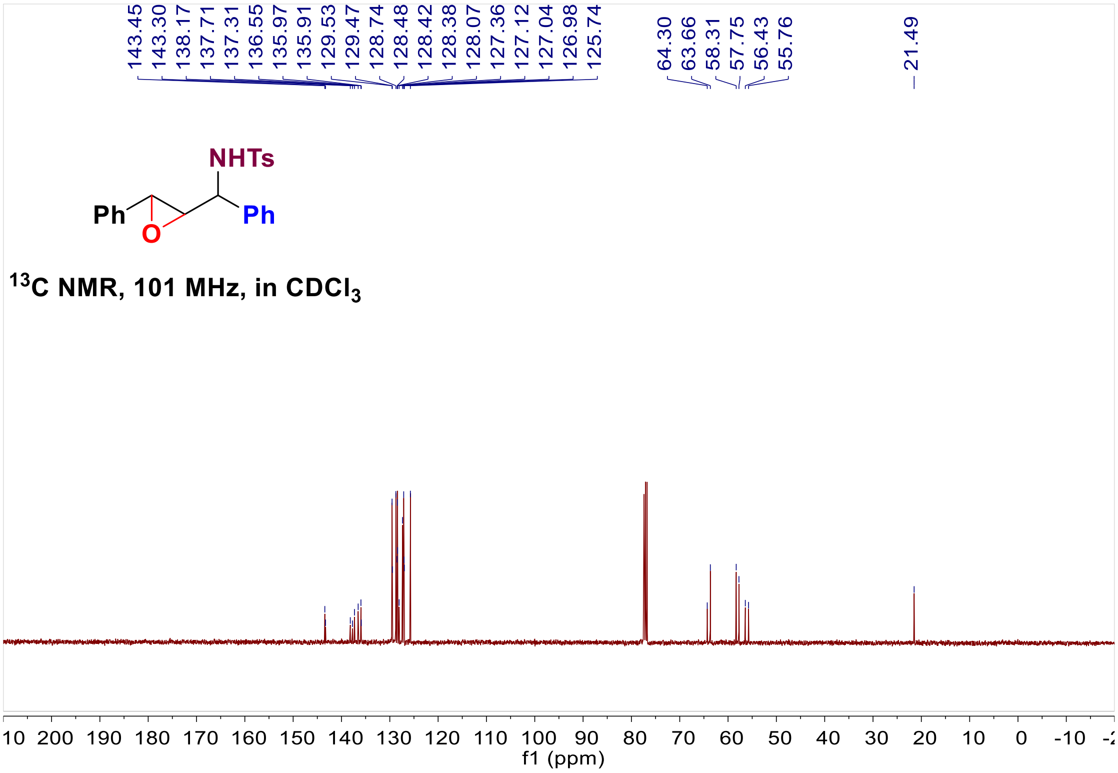

(*E*)-1-cyclohexyl-3-phenylprop-2-en-1-amine (71)

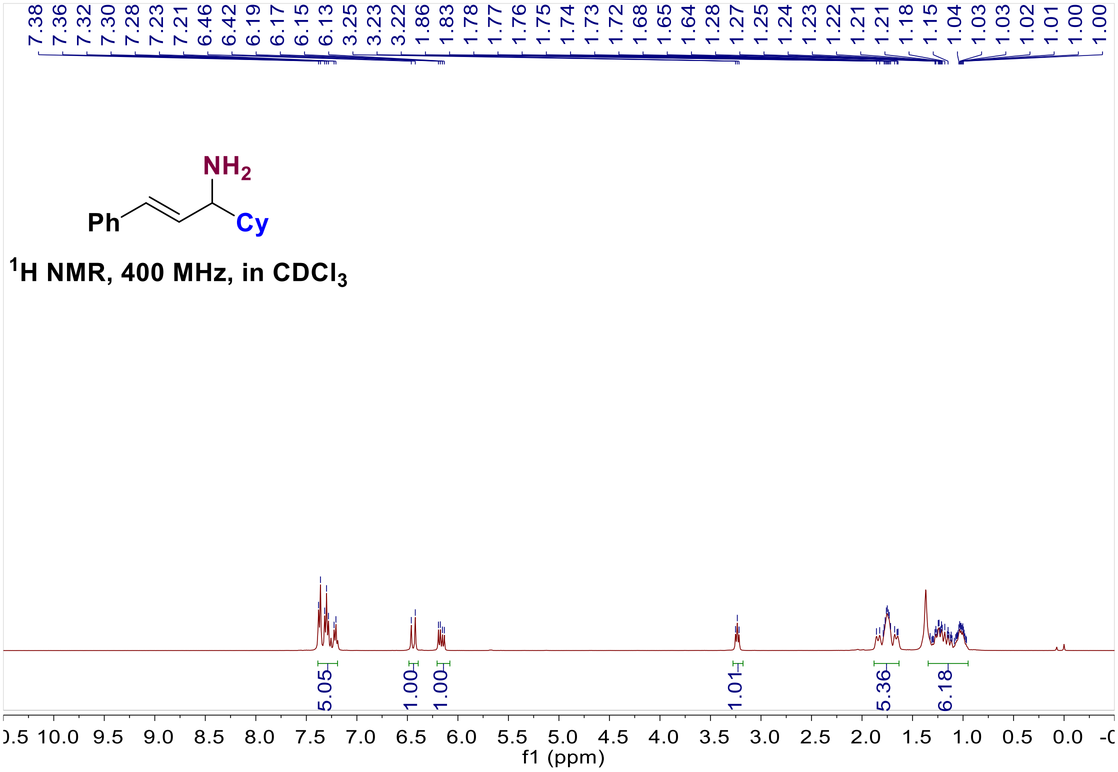

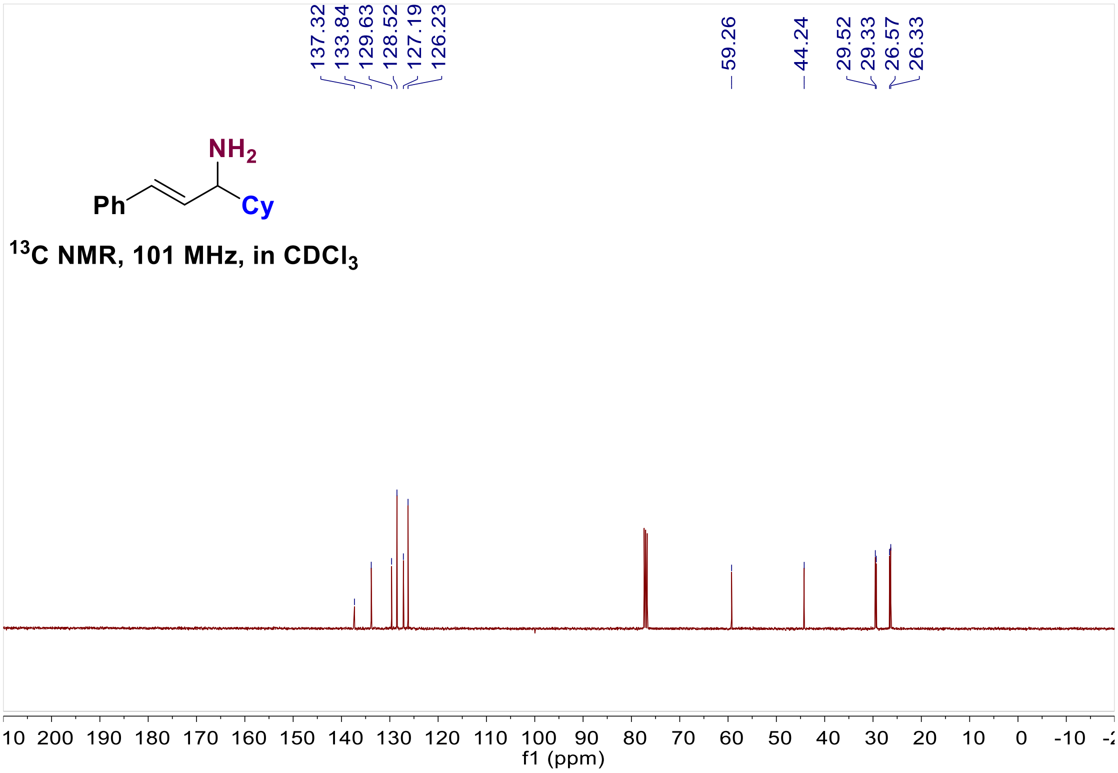

7. Control Experiments:

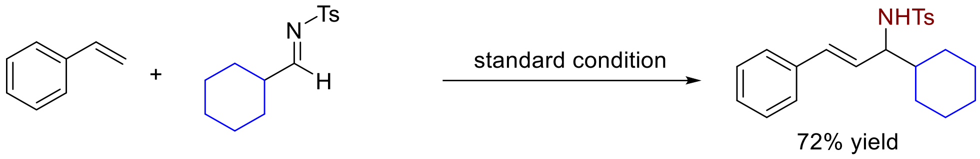

In an N_2-filled glove-box, an oven-dried sealed tube was charged with a stir bar, $Ni(COD)_2$ (5.5 mg, 0.02 mmol), PCy_3 (11.2 mg, 0.04 mmol), (*E*)-N-(cyclohexylmethylene)-4-methylbenzenesulfonamide (53.0 mg, 0.2 mmol, 1.0 equiv), anhydrous acetonitrile (0.5 mL), $Ti(O^iPr)_4$ (12 μL, 20 mol%), styrene (35 μL, 0.30 mmol, 1.5 equiv). The tube was then sealed with a Teflon-lined screw cap, removed from the glove box, and stirred at 100 °C for 12 h. The yields were determined by ^{1}H NMR analysis using dibromomethane as an internal standard.

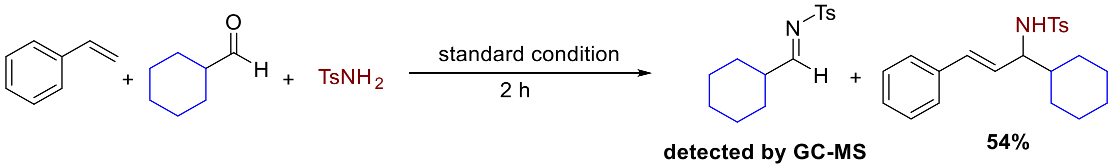

In an N_2-filled glove-box, an oven-dried sealed tube was charged with a stir bar, $Ni(COD)_2$ (5.5 mg, 0.02 mmol), PCy_3 (11.2 mg, 0.04 mmol), *p*-toluenesulfonamide (41.0 mg, 0.24 mmol, 1.2 equiv), anhydrous acetonitrile (0.5 mL) and cyclohexanecarboxaldehyde (24 μL, 0.2 mmol, 1.0 equiv), $Ti(O^iPr)_4$ (12 μL, 20 mol%), styrene (35 μL, 0.30 mmol, 1.5 equiv). The tube was then sealed with a Teflon-lined screw cap, removed from the glove box, and stirred at 100 °C for 2 h. After cooled to room temperature, t the reaction mixture was filtered through a membrane and the filtrate was used for identification. Qualitative analysis was performed by GC-MS. Results were shown in Figure S1. The yields were determined by ^{1}H NMR analysis using dibromomethane as an internal standard.

Figure S1. GC-Ms analysis of the standard reaction mixture

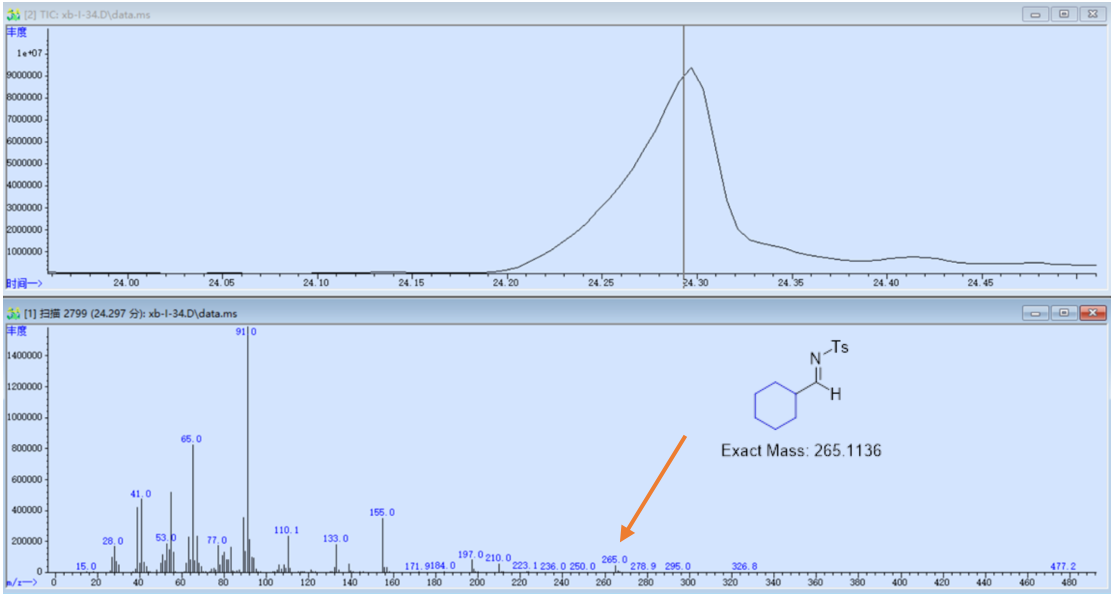

Reference:

1. L.-J. Xiao, C.-Y. Zhao, L. Cheng, B.-Y. Feng, W.-M. Feng, J.-H. Xie, X.-F. Xu and Q.-L. Zhou, *Angew. Chem. Int. Ed.,* 2018, **57**, 3396.
2. C. Fan, X.-Y. Lv, L.-J. Xiao, J.-H. Xie and Q.-L. Zhou, *J. Am. Chem. Soc*., 2019, **141**, 2889.
3. K. O. Cameron and B. A. Lefker (Pfizer Inc), US06344485B1, **2002**.
4. X.-B. Yan, L. Li, W.-Q. Wu, L. Xu, K. Li, Y.-C. Liu and H. Shi, *Nat. Commun*., 2021, **12**, 5881.
5. S. Robin, G. Rousseau, *Eur. J. Org. Chem*., 2000, **17**, 3007.
6. Y. Wang, Z. Lin, J. A. Oliveira and L. Ackermann, *J. Org. Chem*., 2021, **86**, 15935.
